# KHSRP ameliorates acute liver failure by regulating pre-mRNA splicing through its interaction with SF3B1

**DOI:** 10.1038/s41419-024-06886-1

**Published:** 2024-08-26

**Authors:** Mingxuan Li, Qian Fang, Pingping Xiao, Zhinang Yin, Guangbo Mei, Cheng Wang, Ying Xiang, Xuejun Zhao, Lihua Qu, Tian Xu, Jiaxi Zhang, Kejun Liu, Xiaoqing Li, Huifen Dong, Ruijing Xiao, Rui Zhou

**Affiliations:** 1 Hubei Province Key Laboratory of Allergy and Immunology, School of Basic Medical Sciences, Wuhan University, Wuhan, Hubei 430071 China; 2 Department of Medical Parasitology, School of Basic Medical Sciences, Wuhan University, Wuhan, Hubei 430071 China; 3 School of Basic Medical Sciences, Hubei University of Medicine, Shiyan, Hubei 442000 China; 4 Department of Pathophysiology, School of Basic Medical Sciences, Wuhan University, Wuhan, Hubei 430071 China; 5 School of Basic Medical Sciences, Xianning Medical College, Hubei University of Science and Technology, Xianning, Hubei 437000 China; 6 Center for Stem Cell Research and Application, Union Hospital, Tongji Medical School, Huazhong University of Science and Technology, Wuhan, Hubei 430022 China

**Keywords:** Alternative splicing, Liver diseases

## Abstract

Acute liver failure (ALF) is characterized by the rapidly progressive deterioration of hepatic function, which, without effective medical intervention, results in high mortality and morbidity. Here, using proteomic and transcriptomic analyses in murine ALF models, we found that the expression of multiple splicing factors was downregulated in ALF. Notably, we found that KH-type splicing regulatory protein (KHSRP) has a protective effect in ALF. Knockdown of *KHSRP* resulted in dramatic splicing defects, such as intron retention, and led to the exacerbation of liver injury in ALF. Moreover, we demonstrated that KHSRP directly interacts with splicing factor 3b subunit 1 (SF3B1) and enhances the binding of SF3B1 to the intronic branch sites, thereby promoting pre-mRNA splicing. Using splicing inhibitors, we found that Khsrp protects against ALF by regulating pre-mRNA splicing in vivo. Overall, our findings demonstrate that KHSRP is an important splicing activator and promotes the expression of genes associated with ALF progression by interacting with SF3B1; thus, KHSRP could be a possible target for therapeutic intervention in ALF.

## Introduction

Acute liver failure (ALF) is a type of acute hepatic emergency with a high mortality rate. In ALF, rapid deterioration of hepatocyte function can lead to hepatic encephalopathy, cerebral edema, coagulopathy, infection, and multiorgan dysfunction syndrome. The causes of ALF include viral hepatitis (hepatitis A, B, and E) and drug-induced hepatotoxicity due to drugs, such as acetaminophen (APAP) [1, 2]. The treatment of ALF is mainly limited to supportive care and urgent liver transplantation [3]; therefore, there remains a critical need to develop new therapeutic strategies. During ALF, liver cell death normally induces compensatory cell proliferation that can restore liver mass and prevent loss of organ function [4]; however, ALF is exacerbated when the extent of hepatocyte death exceeds the regenerative capacity of the liver [5]. To date, the molecular mechanisms underlying the pathogenesis of ALF remain unclear.

Pre-mRNA splicing is an essential event in the expression of genes with introns. It is performed by splicing machinery (the spliceosome) composed of five small nuclear RNAs (snRNAs: U1, U2, U4, U5, and U6) and >100 associated proteins [6]. The snRNAs associate with several proteins to form small nuclear ribonucleoprotein complexes (snRNPs) and dynamically assemble the spliceosome [7]. The U2 snRNP recognizes the branch site (BS) and interacts with U2AFs to define the 3′ splice site of each intron for spliceosome assembly at the early stage, and latterly functions as a key component in the splicing catalysis [8–10]. SF3b is the largest component of 17 S U2 snRNP and comprises SF3B1, SF3B2, SF3B3, SF3B4, SF3B5, SF3B6, and PHD finger protein 5 A (PHF5A). Moreover, SF3b stabilizes the U2 snRNA/BS interaction and plays a key role in BS recognition and selection during splicing [8, 9, 11]. SF3B1, the largest SF3b subunit, binds and crosslinks to pre-mRNA on both sides of the intron BS region [12, 13].

An accumulation of evidence now suggests that aberrant splicing is strongly associated with many diseases, including myelodysplastic syndrome and acute myeloid leukemia [14–17]. Pharmacological modulation of pre-mRNA splicing is emerging as a promising cancer therapy strategy [14]. In one study, epithelial splicing regulatory protein 2 (ESRP2) was found to coordinate the splicing of many cell proliferation-related genes, including the core components of the Hippo signaling pathway; thus, the study suggested the possible mechanism underlying the adaptation of hepatocytes to injury [18]. Another splicing regulator, SLU7 splicing factor homolog, has been shown to modify the pre-mRNA splicing of sirtuin 1, lipin 1, and serine and arginine-rich splicing factor 3, thereby increasing ethanol-induced inflammation and accelerating alcoholic liver injury in mice [19]. These findings imply that dysregulation of pre-mRNA splicing contributes to liver disease; however, the underlying molecular mechanisms and functional relevance of pre-mRNA splicing dysregulation in liver diseases, especially in ALF, remain unclear.

KH-type splicing regulatory protein (KHSRP) belongs to the RNA-binding protein family, which was first reported as a splicing factor in 1997. Over that last decade, most KHSRP-based studies have focused on its function as a key mediator of mRNA decay [20, 21]. KHSRP recognizes the AU-rich elements (AREs) within the 3′-UTRs of mRNAs and controls their stabilities in the cytoplasm [20, 22]. In addition, KHSRP participates in the promotion of miRNA biogenesis [23]. However, the functional significance of KHSRP in pre-mRNA splicing has received little attention. Wang et al. investigated the mechanism underlying the effects of a small-molecule drug (RG-7916) in the treatment of spinal muscular atrophy and reported that KHSRP could bind to survival motor neuron 2 (SMN2) pre-mRNA and enhance SMN2 splicing [24]. Recently, it has been reported that KHSRP bound to pre-mRNA intronic regions to modulate alternative splicing during monocytic differentiation [25]. Nevertheless, the regulatory mechanism underlying KHSRP-mediated splicing remains unclear.

In this study, we found that dysregulation of pre-mRNA splicing plays an important role in the pathogenesis of ALF. Specifically, we show that KHSRP is a critical determinant of pre-mRNA splicing and controls the expression of genes associated with ALF progression. Our results highlight the important role played by KHSRP in pre-mRNA splicing in ALF and suggest that the targeting of KHSRP may represent a new therapeutic strategy in treating ALF.

## Results

### Decreased expression of pre-mRNA splicing factors in ALF

**Fig. 1:**
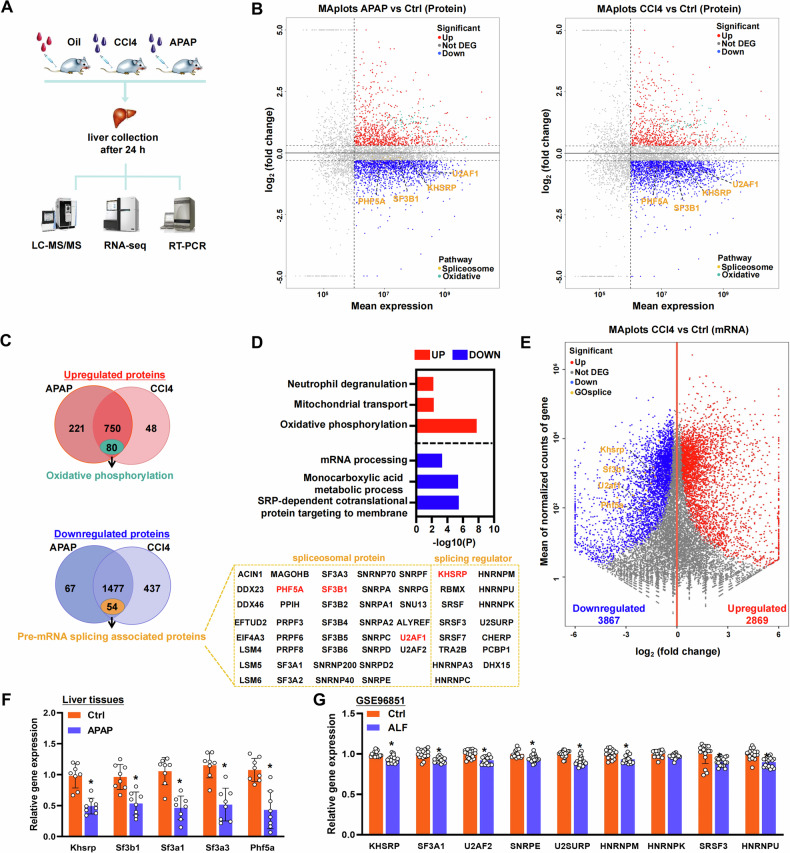
Decreased expression of pre-mRNA splicing factors in ALF. **A** Schematic diagram of the experimental setup. Mice (*n* = 3) were first injected with CCl4 or APAP for 24 h, after which their livers were collected for RNA-Seq, mass spectrometry (MS) analysis, and real-time PCR. **B** Volcano plots showing up- or downregulated proteins in APAP-treated, CCl4-challenged, or oil-treated groups according to MS. Splicing-associated and oxidative stress-related proteins are shown in orange and green, respectively. **C** Comparison of protein expression patterns in the livers of CCl4- and APAP-treated mice. Graphics indicate the proteins that showed increased or decreased expression in the livers. A total of 80 oxidative phosphorylation pathway-related proteins were decreased in both CCl4- and APAP-treated mice, and the 54 pre-mRNA splicing-related proteins downregulated in both CCl4- and APAP-treated mice are listed on the right. **D** GO analysis of MS data showing the proteins differentially expressed in both CCl4- and APAP-treated mice. **E** Volcano plots showing up- or downregulated genes in the control and CCl4-challenged groups according to RNA-Seq. Splicing-related factors are shown in orange. **F** Using real-time PCR, the expression of splicing-related genes was detected in mouse livers following APAP administration for 24 h. **G** Expression of splicing-related genes in ALF (*n* = 17) and control (*n* = 17) clinical samples from GSE96851.

To determine the changes in protein and gene expression in ALF, we performed proteomic analysis using a comprehensive nano-liquid chromatography–tandem mass spectrometry approach combined with RNA-Seq or real-time PCR. Murine ALF models were induced using CCl4 or APAP for 24 h and showed the highest levels of aspartate aminotransferase (AST) and alanine aminotransferase (ALT) after CCl4 or APAP treatment (Fig. 1A and S1A). A total of 41,248, 39,624, and 44,799 peptides were identified (peptide level FDR = 1%) in the CCl4-challenged, APAP-treated, and control mice, respectively, corresponding to 4711, 4608, and 4858 proteins (protein level FDR = 1%), respectively (Fig. 1B). After CCl4 and APAP treatment, the expression of 798 and 971 proteins, respectively, was significantly increased in the liver, whereas that of 1914 and 1544 proteins, respectively, was decreased in the liver (Fig. 1B, C). Interestingly, 750 of the proteins upregulated by CCl4 treatment also showed increased expression in APAP-treated mice (Fig. 1C). In addition, the expression levels of 1477 proteins were decreased in both CCl4-challenged and APAP-treated mice (Fig. 1C). Gene Ontology (GO) analyses of the differentially expressed proteins in CCl4-challenged and APAP-treated mice indicated that upregulated proteins were mainly involved in oxidation phosphorylation, mitochondrial transport, and neutrophil degranulation (Fig. 1D and S1B), whereas the downregulated proteins were enriched in functions such as mRNA processing, monocarboxylic acid metabolic process, and SRP-dependent cotranslational protein targeting to membrane (Fig. 1D and S1B). Pre-mRNA splicing factors were highly enriched in mRNA processing, among which 54 proteins were associated with splicing, including SF3B1, PHF5A, KHSRP, and U2AF1 (Fig. 1B, C). KHSRP has been previously reported as a splicing factor [21]. After CCl4 or APAP injection, Khsrp expression was downregulated in the liver samples (Fig. 1B, C). The expression of SF3B1 and KHSRP was validated using Western blotting (Fig. S1C).

We performed RNA-Seq using the livers of CCl4-challenged mice, and the biological replicates for the RNA-Seq dataset showed good concordance (Fig. S1D). We identified 3867 and 2869 downregulated and upregulated genes, respectively, after CCl4 treatment (adjusted *P* < 0.05; Fig. 1E). GO analyses revealed that the downregulated genes were highly enriched in the RNA catabolic process, consistent with our proteomic data (Fig. S1E). Among the downregulated genes, the expression of *Khsrp*, *Sf3b1*, *Sf3a1*, and *Phf5a* was also decreased at the mRNA level in CCl4-challenged mice (Fig. S1F). Real-time PCR confirmed the downregulation of *Khsrp*, *Sf3b1*, *Sf3a1*, and *Phf5a* in APAP-treated mice (Fig. 1F). Publicly available mice transcriptome datasets supported the downregulation of some pre-mRNA splicing-related genes in CCl4-challenged mice (Fig. S1G). In addition, previously published genome-wide expression profiling data from the livers of patients with ALF and control subjects confirmed that several splicing-related factors, including KHSRP, SF3A1, U2AF2, and HNRNPU, are downregulated in ALF relative to their expression in controls [26] (Fig. 1G). These results indicate that changes in pre-mRNA splicing factors are associated with ALF. Notably, we detected reduced expression of KHSRP, which has been reported as a splicing factor and shows altered expression levels in nonalcoholic fatty liver disease (NAFLD), hepatocellular carcinoma (HCC), and schistosome-induced hepatic fibrosis [27–29].

### Khsrp protects against ALF in vivo

**Fig. 2:**
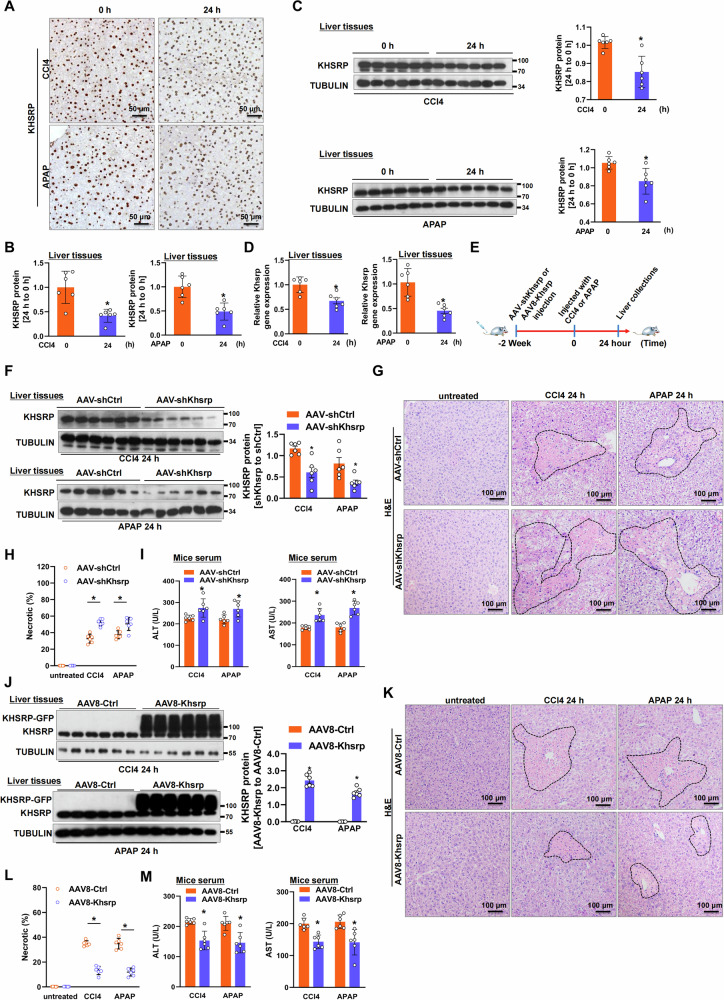
Khsrp protects against ALF in vivo. **A**–**C** Expression of KHSRP protein was detected using (**A**) immunohistochemistry and (**C**) Western blotting in the mouse livers (*n* = 6) following treatment with APAP or CCl4 for 24 h (scale bar: 50 μm). **B** The graph shows KHSRP protein levels in (A) as mean ± SEM. **D** Expression of Khsrp mRNA was detected in the livers induced by APAP or CCl4 for 24 h using real-time PCR. **E** Schematic diagram of the experimental setup. Mice (*n* = 6) were first infected with either AAV-shKhsrp or AAV8-Khsrp for 2 weeks using tail-vein injections. They were then intraperitoneally injected with CCl4 or APAP for an additional 24 h. **F–M** Expression of KHSRP protein was detected in the injured livers after (**F**) AAV-shKhsrp and (**J**) AAV8-Khsrp injection using Western blotting. The graph shows KHSRP protein levels as means ± SEM. H&E staining of liver sections from (**G**) Khsrp-knockdown and (**K**) Khsrp-overexpressing mice following treatment with CCl4 or APAP (scale bars: 100 μm). **H** and **L** Necrosis quantification is shown in the lower panels. (**I** and **M**) Plasma ALT and AST levels in mice. Data represent means ± SEM from three independent experiments. *p < 0.05.

Dysregulation of KHSRP in liver disease has been previously reported [27–30]; however, the role of KHSRP in ALF remains unclear. Thus, we examined the relevance of KHSRP in ALF pathogenesis. In murine ALF models induced using CCl4 or APAP, hepatic KHSRP staining was weaker throughout the cytoplasm and nuclei at 24 h than that in the untreated group (Fig. 2A, B). Furthermore, the downregulation of KHSRP was validated using Western blotting (Fig. 2C). Consistently, according to real-time PCR analysis, the mRNA expression of *Khsrp* was significantly decreased in the livers of mice treated with CCl4 or APAP (Fig. 2D).

To investigate the role of Khsrp in ALF in vivo, we constructed an AAV vector expressing a shRNA directed at *Khsrp* (AAV-shKhsrp) or a control hairpin (AAV-shCtrl) and explored the effect of *Khsrp*-knockdown in ALF (Fig. 2E). Compared to untreated mice, AAVs-injected mice showed high expression of GFP in the liver tissues (Fig S2A). Moreover, AAV-shKhsrp injection significantly decrease the expression of KHSRP by using Western blotting (Fig S2A). AAV-shKhsrp or AAV-shCtrl was injected into BALB/c mice for 2 weeks, and this was followed by CCl4 or APAP injections for 24 h (Fig. 2E). The expression of KHSRP was markedly reduced in the AAV-shKhsrp-treated group than in the controls, indicating that AAV-shKhsrp reduced KHSRP expression effectively in vivo (Fig. 2F and S2A). Hematoxylin and eosin (H&E) staining showed that the area of hepatic necrosis was significantly expanded in the *Khsrp*-knockdown animals following CCl4 or APAP treatment (Fig. 2G, H). Furthermore, serum levels of aminotransferases, such as AST and ALT, were increased in *Khsrp*-knockdown mice after CCl4 or APAP injection (Fig. 2I). Moreover, we examined whether ectopic Khsrp expression alleviates ALF in vivo. AAV8 vectors were used to deliver Khsrp to mice with APAP- or CCl4-induced ALF (Fig. 2E). The hepatic expression of KHSRP significantly increased in mice treated with AAV8-Khsrp (Fig. 2J and S2A). Decreased hepatic necrosis was detected in AAV8-Khsrp-infected mice following CCl4 or APAP injection (Fig. 2K, L). Moreover, ALT and AST levels in AAV8-Khsrp-infected mice were reduced compared with those in the controls after CCl4 or APAP treatment (Fig. 2M). In addition, according to the previous studies, hydrodynamic tail-vein injection with plasmids were used to overexpress the target gene in hepatocytes [31, 32]. Plasmid-mediated overexpression (pcDNA3.1-Khsrp injection) was used to increase KHSRP expression in hepatocytes. The results were similar in mice first injected with Khsrp-expressing vectors (pcDNA3.1-Khsrp) and then treated with CCl4 or APAP for 24 h (Fig S2B). Ectopic KHSRP expression in the Khsrp-expressing vector-treated group was confirmed compared with that in the control group (Fig S2C–E). H&E staining showed that the area of hepatic necrosis observed in the control was diminished in the Khsrp vector-treated group injected with CCl4 or APAP (Fig S2D, E). Notably, ALT and AST levels in Khsrp vector-treated mice were reduced compared with those in the controls after CCl4 or APAP treatment for 24 or 48 h (Fig S2F, G).

### *Khsrp* depletion induces intron retention

**Fig. 3:**
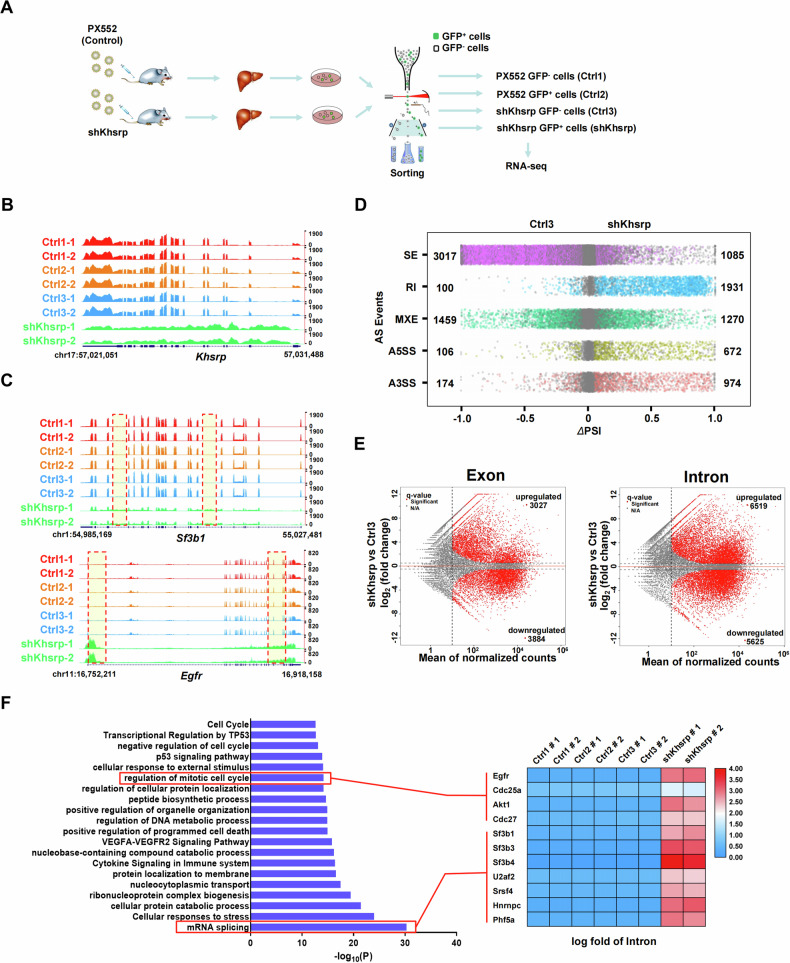
*Khsrp* depletion induces intron retention. **A** Schematic diagram of the experimental setup. Mice (*n* = 2) were first infected with either AAV-shKhsrp or PX552 (control) for 2 weeks via tail-vein injection. Primary hepatocytes were then isolated and sorted using flow cytometry. Cells were divided into four groups, namely, PX552 GFP^−^ (Ctrl1), PX552 GFP^+^ (Ctrl2), shKhsrp GFP^−^ (Ctrl3), and shKhsrp GFP^+^ (shKhsrp), and prepared for RNA-Seq analysis. **B**, **C** Genome browser tracks of RNA-Seq signals at (**B**) *Khsrp* and at (**C**) *Egfr* and *Sf3b1* in the Ctrl1, Ctrl2, Ctrl3, and shKhsrp groups. Tracks of RNA-Seq signals at the intron-retained regions are shown by dotted boxes. **D** Scatter plots show changes in splicing events between Ctrl3 and shKhsrp groups. Using rMATS, five types of AS events were analyzed: Retained introns (RIs), skipped exons, alternative 5′ and 3′ splice sites (A5SS and A3SS, respectively), and mutually exclusive exons. Significantly changed events (|ΔPSI| > 0.05, FDR < 0.05, and supporting reads ≥ 5) are shown by color dots. **E** Volcano plots showing up- or downregulated exons or introns in Ctrl3 or shKhsrp groups according to RNA-Seq analysis. **F** GO analysis based on RNA-Seq results showing mRNA expression from intron-retained genes after Khsrp-knockdown in primary hepatocytes (left). Heatmaps of mRNA splicing factors and cell cycle-related genes are also shown (right).

To investigate the regulatory network of KHSRP, we performed RNA-Seq using primary hepatocytes isolated from AAV-shKhsrp-injected mice. Specifically, mice were treated with AAV-shKhsrp or the control construct (PX552) for 2 weeks via tail-vein injection. AAV-infected GFP-positive hepatocytes were purified using fluorescence-activated cell sorting (Fig. 3A and S3A), and the effect of *Khsrp*-knockdown in these cells was confirmed using RNA-Seq and real-time PCR (Fig. 3B and S3B). The biological replicates for RNA-Seq datasets showed good concordance (Fig. S3C). Given that KHSRP functions as a splicing factor as previously reported [21], we aimed to determine the contribution of KHSRP to pre-mRNA splicing. Interestingly, *Khsrp*-knockdown resulted in increased introns at the intron regions of Sf3b1, Egfr, Cdc25a, and Phf5a RNA transcripts (Fig. 3C and S3D). Using the common differential alternative splicing (AS) analysis tool rMATS [13], we identified *Khsrp*-knockdown induced the alteration of 10,788 AS events in primary hepatocytes (Fig. 3D). Whereas in control cells, relatively less changes in AS events were observed (Fig. S3E). Splicing analysis revealed that Khsrp regulated various types of AS events, including skipped exons (SEs), alternative 5′ splice sites, alternative 3′ splice sites, retained introns (RIs), and mutually exclusive exons (Fig. 3D). Notably, we identified a strong defect in splicing with 1931 introns displaying intron retention (RIs in Fig. 3D, right panel) in the shKhsrp group, which was close to 20-fold higher than the level of RIs in control cells. In addition, SEs were the main splicing change upon silencing of *Khsrp* in primary hepatocytes (Fig. 3D). These dramatic changes in RIs and SEs in *Khsrp*-knockdown primary hepatocytes suggested that the depletion of *Khsrp* significantly inhibits pre-mRNA splicing, implying KHSRP is an activator of splicing. We analyzed differentially expressed exons and introns (adjusted P < 0.05), and 6911 exons (3027 upregulated) and 12,144 introns (6519 upregulated) were differentially expressed in *Khsrp*-depleted primary hepatocytes compared with the expression in the controls (Fig. 3E). Notably, most of the Khsrp-responsive introns were retained, and the number of upregulated and downregulated exons was almost equal, indicating that *Khsrp* depletion induces most of the intron retention (Fig. 3E). Given that the production of RI RNAs is usually associated with gene repression [33], we performed GO analysis of genes with intron retention in *Khsrp*-knockdown cells. GO analysis of 638 genes (log2Foldchange > 1, baseMean > 5000, and q-value < 0.05) revealed that Khsrp-regulated genes with intron retention were highly enriched in mRNA splicing, cellular response to stress, and regulation of the mitotic cell cycle (Fig. 3F and Supplementary Table 3). Intriguingly, *Khsrp*-knockdown increased the intron levels of some pre-mRNA splicing-related genes, including *Sf3b1* and *Phf5a* (Fig. 3C and S3D), thereby implicating KHSRP as an important regulator of pre-mRNA splicing.

To confirm whether KHSRP regulates pre-mRNA splicing in HL7702 cells, we designed two shRNAs to target different regions of *KHSRP* (KHSRP shRNA1 and shRNA2, respectively) and confirmed that both effectively silenced the expression of KHSRP (Fig. S4A). After the stable knockdown of *KHSRP* with shRNAs, RNA-Seq was used to determine the effect of *KHSRP* depletion on global transcription and splicing in HL7702 cells. The biological replicates for RNA-Seq datasets showed good concordance (Fig. S4B). The *KHSRP*-depleted transcript in HL7702 cells was shown using genome browser tracks (Fig. S4C). In agreement with our primary mouse hepatocyte data, *KHSRP*-knockdown increased the intron levels of EGFR, SF3B1, CDC25A, SF3B4, SF3B6, and PHF5A transcripts in HL7702 cells (Fig. S4D). Furthermore, RIs were the main splicing change following the silencing of *KHSRP* in HL7702 cells (Fig. S4E, F). GO analysis of 764 genes (log2Foldchange > 1, baseMean > 5000, and q-value < 0.05) in which intron retention was induced by *KHSRP* depletion revealed that the genes were significantly enriched with various functions, including RNA metabolism, mRNA splicing, regulation of mRNA processing, and the cell cycle (Fig. S4G and Supplementary Table 4). Overall, these data indicate that KHSRP plays an important role in promoting pre-mRNA splicing.

### KHSRP interacts with SF3B1

**Fig. 4:**
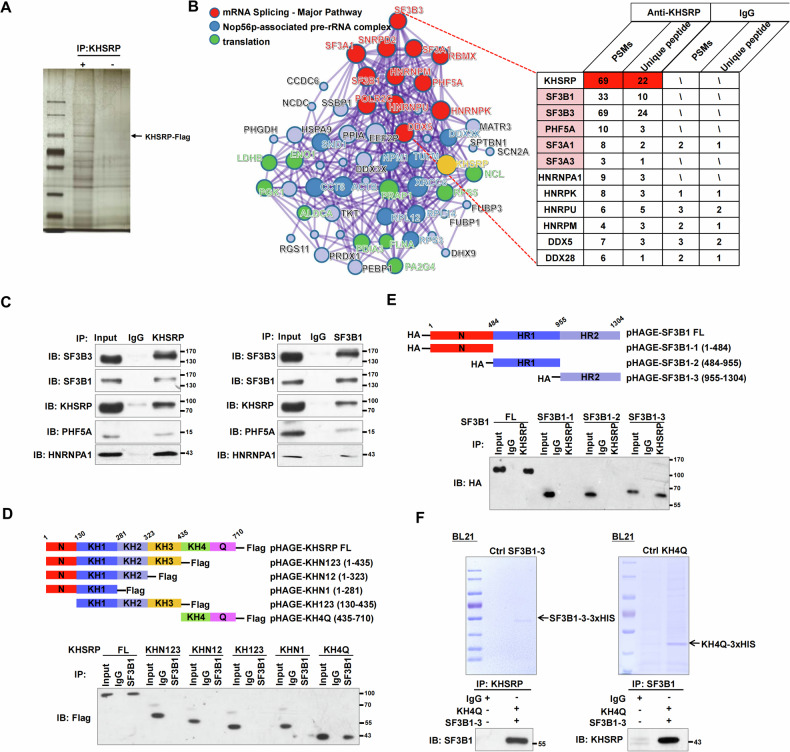
KHSRP interacts with SF3B1. **A** The Flag-KHSRP protein was enriched with KHSRP antibody and detected using silver staining. Arrowhead represents the predicted size of KHSRP. **B** STRING network analysis of the KHSRP protein interactome. Red circles, mRNA splicing; blue circles, Nop56p-associated pre-rRNA complex; green circles, translation (left). KHSRP-interacting splicing complex enriched over IgG. Data from mass spectrometry analysis are shown in the table (right). Peptide-spectrum match counts (#PSM). U2 snRNPs are labeled in pink. **C** Coimmunoprecipitation of KHSRP and pre-mRNA splicing factors. Protein extracts from HL7702 cells were immunoprecipitated using the antibodies for KHSRP or SF3B1 and immunoblotted with the antibodies for the indicated proteins. HNRNPA1 was used as a positive control. **D** Schematic diagram of KHSRP truncations. The numbers shown indicate the amino acid position (top). Detection of the binding domain of KHSRP that interacts with SF3B1 in a coimmunoprecipitation assay using HL7702 cells that overexpressed different Flag-tagged KHSRP truncations (bottom). **E** Schematic diagram of SF3B1 truncations. The numbers shown indicate the amino acid position (top). Detection of the binding domain of SF3B1 that interacts with KHSRP in a coimmunoprecipitation assay using HL7702 cells that overexpressed different constructs with HA-tagged SF3B1 truncations (bottom). **F** The interaction between KHSRP-KH4Q and SF3B1-3 was tested in a cell-free system. The inputs and recombinant proteins that were pulled down were detected using Coomassie staining and Western blotting.

Given that *KHSRP*-knockdown disrupts pre-mRNA splicing, we hypothesized that the physical interactions between KHSRP and the core splicing factors are required for the regulation of pre-mRNA splicing. To test this hypothesis, we performed immunoprecipitation using anti-KHSRP antibody in HL7702 cells and then used mass spectrometry to identify the potential binding partners of KHSRP (Fig. 4A and S5A). We identified 173 protein candidates with at least a twofold enrichment over IgG (Supplementary Table 5). Proteomic network analysis indicated that most of these candidates, including SF3B1, SF3B3, PHF5A, and SF3A1, were involved in pre-mRNA splicing (Fig. 4B). Interestingly, the set of proteins that interacts with KHSRP belongs to 17 S U2 snRNP (Fig. 4B). The interactions between KHSRP and the proteins of the SF3b complex, e.g., SF3B1, SF3B3, and PHF5A, were confirmed using coimmunoprecipitation (Fig. 4C). HNRNPA1 was used as a positive control for interacting with KHSRP, as previously reported [34]. Meanwhile,we also validated the interactions between KHSRP and SF3b complex in HEK293T cells (Fig. S5B). Given that KHSRP interacts with some of the proteins of 17 S U2 snRNPs from our mass spectrometry data, we investigated whether intracellular KHSRP binds to U2 snRNA in HL7702 cells using formaldehyde crosslinking RNA-binding protein immunoprecipitation. We detected highly enriched U2 snRNA but not U4 and U6 snRNA with KHSRP (Fig. S5C).

Given that SF3B1 functions as a core component of U2 snRNP, we considered the interaction between KHSRP and SF3B1. Thus, we generated a series of KHSRP-expressing constructs with truncated coding regions (amino acids 1–435 in which the KH4 and Q-rich domains were deleted, amino acids 1–323 containing the KH1 and KH2 domains, and amino acids 1–281 containing the KH1 domain, etc.; Fig. 4D). We found that the KH4 and Q-rich domain (435–710) of KHSRP was required for the interaction with SF3B1 (Fig. 4D). Moreover, we constructed a series of HA-SF3B1 mutants (amino acids 1–484 containing the coiled-coil region; amino acids 484–955 containing the HEAT 1–10 domain, which binds to U2-U6 snRNAs; and amino acids 955–1304 containing the HEAT 11–22 domain, which is required for binding to SF3B3 and PHF5A). We used these truncated constructs to identify the essential motifs in SF3B1 for the interaction with KHSRP, finding that the HEAT 11–22 domain of SF3B1 (amino acids 955–1304) was crucial for the binding of SF3B1 with KHSRP (Fig. 4E). Finally, we generated pET15b-KHSRP-KH4Q (amino acids 435–710) and pET15b-SF3B1-3 (amino acids 955–1304) constructs and confirmed the interaction between the KH4–Q-rich domain of KHSRP and the HEAT 11–22 domain of SF3B1 in vitro (Fig. 4F). Overall, the results of these in vitro binding assays indicate that KHSRP directly interacts with SF3B1.

### KHSRP promotes pre-mRNA splicing through the interaction with SF3B1

**Fig. 5:**
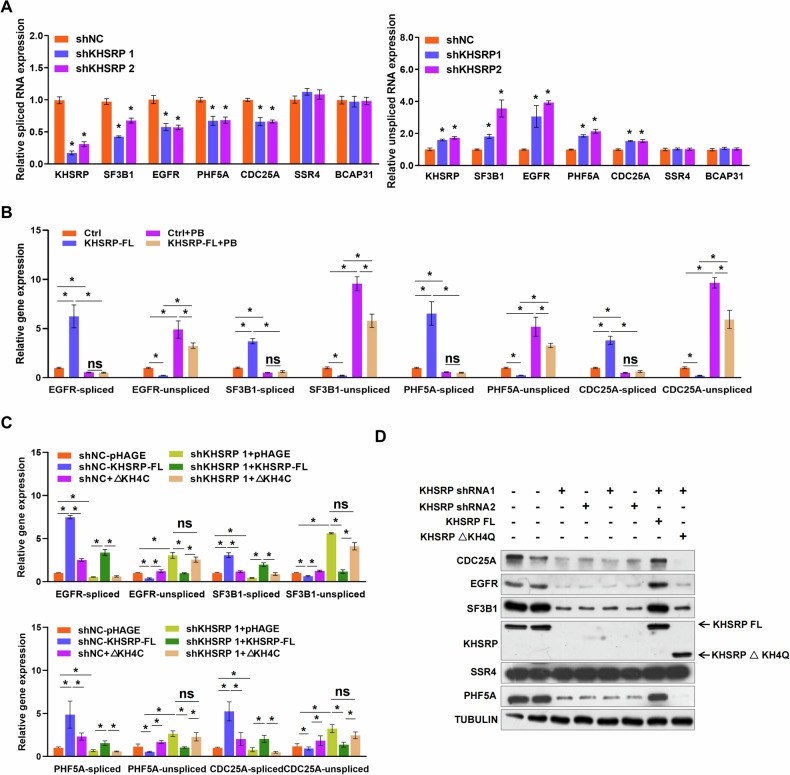
KHSRP promotes pre-mRNA splicing through the interaction with SF3B1. **A** Spliced or unspliced mRNAs of KHSRP, SF3B1, EGFR, PHF5A, CDC25A, SSR4, and BCAP31 were measured using real-time PCR in stable KHSRP-knockdown HL7702 cells. **B**, **C** Spliced or unspliced EGFR, SF3B1, PHF5A, and CDC25A mRNAs were detected in (**B**) HL7702 cells with stable overexpression of KHSRP in the presence or absence of PB or (**C**) KHSRP-knockdown cells transfected with pHAGE-KHSRP-FL or pHAGE-KHSRP-ΔKH4Q. **D** Protein levels of KHSRP, EGFR, PHF5A, CDC25A, SF3B1, and SSR4 were detected in KHSRP-knockdown cells transfected with pHAGE-KHSRP-FL or pHAGE-KHSRP-ΔKH4Q using Western blotting. Data represent means ± SEM from three independent experiments. *p < 0.05; NS, no significant difference compared with the control.

To investigate the mechanism underlying intron retention following *KHSRP* depletion, we verified our RNA-Seq data using real-time PCR with primers that could detect the spliced and unspliced RNAs of KHSRP-responsive genes [[35], Supplementary Table 2]. Consistent with our RNA-Seq data, *KHSRP*-knockdown reduced the levels of the spliced forms of several KHSRP-responsive genes and increased the levels of the unspliced mRNAs (Fig. 5A). *SSR4* and *BCAP31*, two genes not related to KHSRP, were used as negative controls (Fig. 5A and S5D). We also confirmed the downregulation of EGFR and SF3B1 at the protein level in *KHSRP*-knockdown cells using Western blotting (Fig. S5E). KHSRP overexpression restored the protein levels of EGFR and SF3B1 that were downregulated due to *KHSRP* depletion (Fig. S5E). Moreover, the expression of EGFR and SF3B1 was significantly reduced in the liver tissues of *Khsrp*-knockdown mice (Fig. S5F, G).

To investigate the signaling cascade of KHSRP in pre-mRNA splicing, HL7702 cells were treated with pladienolide B (PB), an inhibitor of a splicing modulator of the SF3b complex. As expected, PB suppressed the expression of SF3B1 significantly at the mRNA and protein levels (Fig. S5H). We also measured the levels of spliced and unspliced mRNAs in HL7702 cells with stable overexpression of KHSRP in the presence or absence of PB, finding that overexpression of KHSRP increased the expression of the spliced mRNAs of EGFR, SF3B1, PHF5A, and CDC25A but decreased the expression of unspliced mRNAs; however, PB blocked the upregulation of spliced mRNAs induced by KHSRP overexpression (Fig. 5B). In this case, *SSR4* and *BCAP31* were used as negative controls (Fig. S5I).

Our data indicate that the KH4 and Q-rich domain (435–710) of KHSRP is required for the interaction with SF3B1; thus, we examined the possibility that this domain is responsible for the regulation of pre-mRNA splicing. Stable *KHSRP*-knockdown HL7702 cells were transfected with pHAGE-KHSRP-FL (complete *KHSRP* CDS) or pHAGE-KHSRP-ΔKH4Q (without the KH4 and Q-rich domain of KHSRP) (Fig. S5J). The expression of spliced EGFR, SF3B1, PHF5A, and CDC25A mRNAs markedly decreased in pHAGE-KHSRP-ΔKH4Q-transfected cells than in pHAGE-KHSRP-FL-transfected cells (Fig. 5C). In contrast, pHAGE-KHSRP-ΔKH4Q transfection increased the expression level of unspliced mRNAs compared with that observed with pHAGE-KHSRP-FL transfection (Fig. 5C). Moreover, pHAGE-KHSRP-FL transfection led to a significant increase in the expression of spliced EGFR, SF3B1, PHF5A, and CDC25A mRNAs and a substantial decrease in unspliced mRNAs in *KHSRP*-knockdown cells (Fig. 5C). pHAGE-KHSRP-ΔKH4Q transfection failed to rescue the *KHSRP* depletion-induced downregulation of spliced or upregulation of unspliced EGFR, SF3B1, PHF5A, and CDC25A mRNA expression (Fig. 5C). In addition, pHAGE-KHSRP-ΔKH4Q did not rescue the *KHSRP* depletion-induced downregulation of EGFR, SF3B1, PHF5A, and CDC25A at the protein level (Fig. 5D). In this case, SSR4 was used as a negative control (Fig. 5D). These results show that the KH4 and Q-rich domain of KHSRP is specifically involved in the regulation of pre-mRNA splicing.

**Fig. 6:**
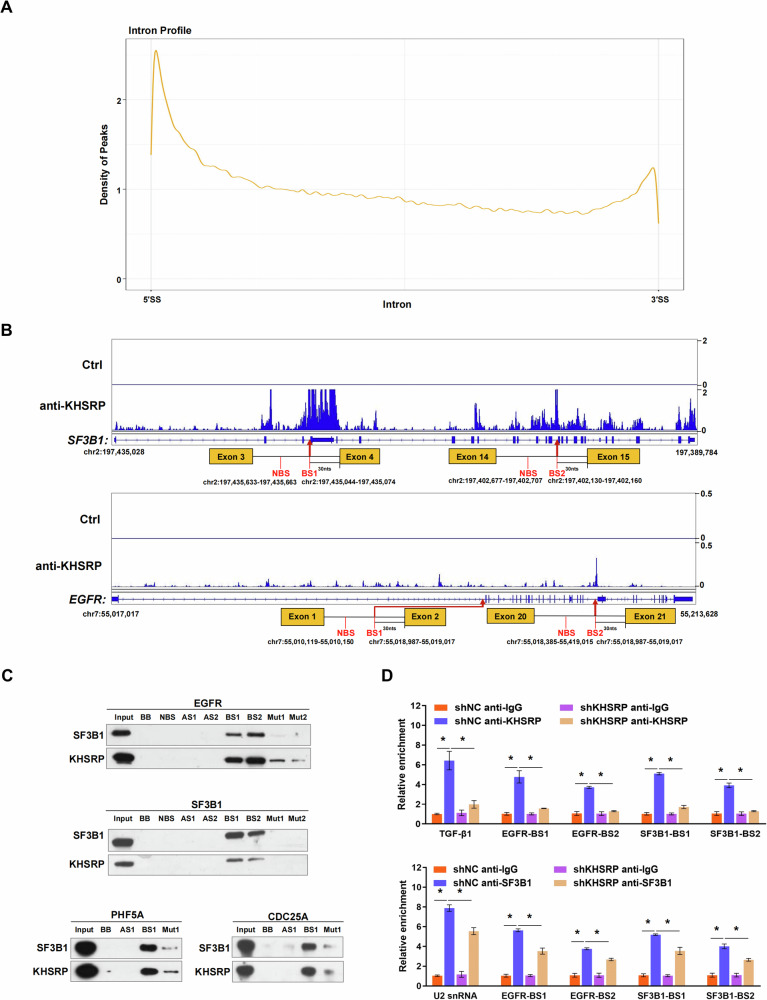
KHSRP is responsible for recruiting U2 snRNP to the BS regions. The distribution of KHSRP CLIP peak centers in the intronic regions according to eCLIP data (GSE177285). **A** The binding regions of KHSRP are enriched in introns, within which KHSRP preferentially binds at the regions close to the 5′ and 3′ splice sites, the latter of which is the typical location of the BS region. **B** Enriched peaks of KHSRP were observed at BS regions on introns 1 and 20 of EGFR and introns 3 and 14 of SF3B1 according to eCLIP data (GSE177285). NBS represents non-BS region on the introns. **C** Western blot analysis of SF3B1, EGFR, PHF5A, and CDC25A pulled down using biotinylated BS RNA probes in HL7702 cells. Antisense RNAs (AS), the non-BS region on the introns (NBS), blank beads, and BS mutated probes (Mut) were used as negative controls. **D** Associations of KHSRP or SF3B1 with RNAs containing BS regions were detected in KHSRP-knockdown cells using RIP. RNAs enriched with IgG were set as 1. The ARE regions in the TGF-β1 3′-UTR and U2 snRNA were used as positive controls. Data represent means ± SEM from three independent experiments. *p < 0.05.

Given that KHSRP promotes pre-mRNA splicing, we characterized the RNA binding sites of KHSRP by analyzing the eCLIP data of KHSRP in HepG2 cells from the GEO database. The binding regions of KHSRP are enriched in introns, within which KHSRP preferentially binds at the regions close to the 5′ and 3′ splice sites, the latter of which is the typical location of the BS region (Fig. 6A). Based on analysis of the eCLIP data, we observed enriched peaks of KHSRP at BS regions on introns 1 and 20 of EGFR and introns 3 and 14 of SF3B1 (Fig. 6B).

We conducted a RNA pull-down assay using biotinylated probes to determine whether KHSRP could bind with the BS region at the retained intronic regions of EGFR, SF3B1, CDC25A, and PHF5A (Fig. 6B, C and S6A). Antisense transcripts, probes detecting the non-BS region on the introns, and blank beads were used as negative controls (Fig. 6B). RNA probes that detect mutated BS were also included. Consistent with the previous study [36], SF3B1 made contact with the BS region of pre-mRNA, and KHSRP was able to bind to the BS region on the introns of pre-EGFR, pre-SF3B1, pre-PHF5A, and pre-CDC25A in vitro (Fig. 6C). Notably, the protein bands of KHSRP and SF3B1 were more enriched with the RNA probes containing the BS region than the mutated RNA probes, suggesting that the affinities of KHSRP and SF3B1 are decreased when the mutation is present (Fig. 6C). To confirm these interactions, we conducted a RIP assay using primers covering the BS regions of pre-EGFR, pre-SF3B1, pre-PHF5A, and pre-CDC25A (Fig. S6A). The resultant data showed that KHSRP and SF3B1 bound to the BS regions of pre-EGFR, pre-SF3B1, pre-PHF5A, and pre-CDC25A (Fig. 6D and S6B). Notably, the binding of SF3B1 to the BS regions of pre-EGFR and pre-SF3B1 was significantly attenuated in *KHSRP*-knockdown cells (Fig. 6D). Moreover, *KHSRP*-knockdown decreased the binding of SF3B1 protein with U2 snRNA (Fig. S6C). Taken together, these data suggest that KHSRP facilitates the interaction between SF3B1 and U2 snRNA and is responsible for recruiting U2 snRNP to the BS regions.

### Khsrp protects against ALF partially through the regulation of pre-mRNA splicing

**Fig. 7:**
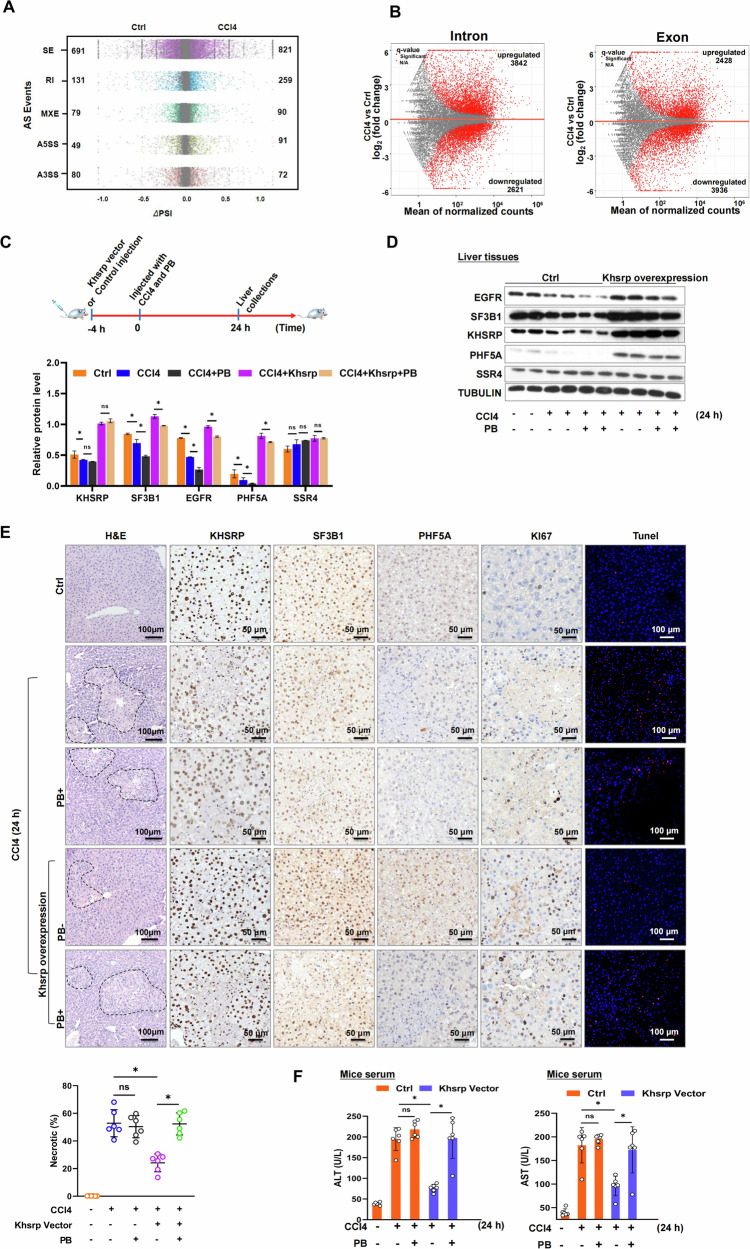
Khsrp protects against ALF partially by regulating pre-mRNA splicing. **A** Scatter plots show changes in splicing events between Ctrl and CCl4-treated groups. Using rMATS, five types of AS events were analyzed: Retained introns (RIs), skipped exons, alternative 5′and 3′splice sites (A5SS and A3SS, respectively), and mutually exclusive exons. Significantly changed events (|ΔPSI| > 0.05, FDR < 0.05, and supporting reads ≥ 5) are shown by color dots. **B** Volcano plots showing up- or downregulated exons and introns in control or CCl4-treated groups according to RNA-Seq analysis. **C** Schematic diagram of the experimental setup. Mice (*n* = 6) were first injected with Khsrp vectors or controls for 4 h via hydrodynamic tail-vein injections, after which they were intraperitoneally injected with CCl4 and/or PB for an additional 24 h. **D** Protein levels of KHSRP, EGFR, SF3B1, PHF5A, and SSR4 in mouse livers treated with CCl4 for 24 h in the presence or absence of PB according to Western blotting. Graph shows protein levels as means ± SEM. **E** H&E staining of liver sections from Khsrp-overexpressing mice treated with CCl4 in the presence or absence of PB (scale bar: 100 μm). Necrosis quantification is shown in the below panel. Expression of KHSRP, SF3B1, PHF5A, and KI67 proteins was detected using immunohistochemistry (scale bar: 50 μm). Immunohistochemistry analysis and quantification of a TUNEL assay (red) in the liver sections (scale bar: 100 μm). **F** Plasma ALT and AST levels in mice. *p < 0.05.

Given that the expression of Khsrp was decreased in murine ALF models and that *Khsrp*-knockdown induced intron retention through the interaction with SF3B1, we hypothesized that intron retention was involved in the pathogenesis of ALF. To test this hypothesis, we analyzed RNA-Seq data from the livers of CCl4-challenged and control mice. As expected, CCl4 injection induced intron retention relative to that detected in the controls (Fig. 7A). Notably, splicing analysis revealed that a strong defect in splicing with 259 introns displaying intron retention (Fig. 7A, left panel) in the CCl4-challenged group, which was close to 2-fold higher than the level of RIs in control group. Moreover, 3842 introns and 2428 exons were upregulated in CCl4-challenged mice than in the controls (Fig. 7B). We also used PB as a splicing inhibitor to investigate whether Khsrp protects against ALF through the regulation of pre-mRNA splicing in vivo. Mice were randomized to receive either vehicle (DMSO) or PB (3.00, 1.50, or 0.75 mg/kg) intraperitoneally, and liver samples were collected after 24 h (Fig. S7A). We measured SF3B1 and KHSRP expression using real-time PCR and Western blotting. The expression of SF3B1 was markedly reduced in the mouse livers treated with PB (3.00, 1.50, and 0.75 mg/kg) (Fig. S7B, C). However, there was no significant difference in the KHSRP level in mice after PB injection at 0.75 mg/kg (Fig. S7B). Furthermore, there were no significant changes of Khsrp in the primary hepatocytes isolated from mice after PB injection (0.75 mg/kg) by real-time PCR and Western blot (Fig. S7C). ALT and AST levels markedly increased in PB-treated mice (3.00 and 1.50 mg/kg) than in the controls (Fig. S7D). Furthermore, H&E staining showed that the area of hepatic necrosis was significantly larger in the PB-treated group (3.00 and 1.50 mg/kg) (Fig. S7E). Contrastingly, PB treatment at 0.75 mg/kg did not lead to evident hepatic damage (Fig. S7D, E). Intriguingly, we noted marked differences in the behavior and feeding habits of vehicle- and high-dose (3.00 and 1.50 mg/kg) PB-treated mice (data not shown), which has not been previously reported [37, 38]. Furthermore, we performed RNA-seq using primary hepatocytes isolated from PB-injected mice (0.75 mg/kg). Splicing analysis showed that PB treatment at 0.75 mg/kg induced splicing defect with alteration of 498 AS events in primary hepatocytes, especially 74 introns displaying intron retention (Fig. S7F). These data suggested that PB treatment at 0.75 mg/kg effectively disturbs RNA splicing in vivo. Based on these results, we used 0.75 mg/kg PB in our in vivo studies. PB treatment at 0.75 mg/kg reduced the expression of SF3B1, as shown in immunohistochemistry analysis (Fig. S7G). In mice treated with CCl4 with or without PB for 24 h and then tail-vein-injected hydrodynamically with Khsrp-expressing vectors (pcDNA3.1-Khsrp) for 4 h (Fig. 7C), PB treatment induced a decrease in EGFR, SF3B1, and PHF5A protein expression levels (Fig. 7D). The expression of KHSRP, EGFR, SF3B1, and PHF5A was, as expected, significantly increased in the Khsrp-expressing vector-treated group (Fig. 7D). In this case, SSR4 was used as a negative control. These data were confirmed using immunohistochemistry (Fig. 7E). H&E staining and AST/ALT assays revealed that the combined stimulation of CCl4 and PB did not result in more serious liver injury as compare with only CCL4 treatment (Fig. 7E, F). Importantly, PB treatment reversed the hepatic protection conferred by Khsrp overexpression in CCl4-challenged mice (Fig. 7E). Moreover, the levels of ALT and AST significantly increased in the Khsrp vector-treated group following PB injection in CCl4-challenged mice (Fig. 7F). Consequently, these results suggest that PB treatment partially reverses the hepatic protection conferred by Khsrp overexpression in CCl4-challenged mice.

To determine how PB reverses Khsrp-mediated protection against hepatic injury, we examined the proliferation (KI67) and apoptosis (TUNEL assay) of hepatocytes in the Khsrp-expressing vector-treated group in the presence and absence of PB. CCl4 treatment increased the expression of KI67 and the number of apoptotic cells (Fig. 7E, S7H), consistent with the results in previous studies [39, 40]. Khsrp overexpression further increased KI67 expression and inhibited the apoptosis of hepatocytes, suggesting that it alleviated liver damage (Fig. 7E). However, PB injection decreased the expression level of KI67 and increased the number of apoptotic cells in Khsrp-overexpressing mice (Fig. 7E). These data indicate that PB exacerbates liver damage in Khsrp-overexpressing mice and nullifies the protective effects of Khsrp.

Our results indicate that KHSRP promotes EGFR splicing. EGFR signaling has been shown to protect the liver from cholestatic injury and fibrosis [41]. Thus, we used Egfr to investigate whether Khsrp overexpression alleviates liver injury in mice through pre-mRNA splicing. To manipulate EGFR signaling, we used the cancer therapy drug erlotinib hydrochloride, an inhibitor of EGFR-associated tyrosine kinase. The expression levels of EGFR signaling-related genes, including *EGFR*, *EGF*, *ERBB2*, and *ERBB4*, were downregulated in HL7702 cells following erlotinib treatment (Fig. S8A). We treated mice with erlotinib orally for 3 days and then injected the mice intraperitoneally with CCl4, after which they received a hydrodynamic tail-vein injection with Khsrp-expressing vectors (Fig. S8B). Khsrp overexpression increased the expression of EGFR, whereas erlotinib injection significantly decreased EGFR expression levels, as confirmed using immunohistochemistry (Fig. S8C). Oral treatment with erlotinib in the Khsrp overexpression group enlarged the area of hepatic necrosis and increased the serum activity of ALT and AST (Fig. S8C, D). Erlotinib treatment also reduced KI67 expression and increased the number of apoptotic hepatocytes in Khsrp-overexpressing mice after CCl4 injection (Fig. S8C).

## Discussion

**Fig. 8:**
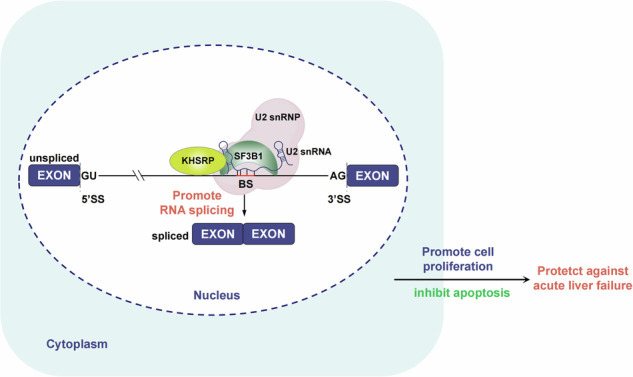
KHSRP protects against ALF by regulating pre-mRNA splicing through its interaction with SF3B1. KHSRP interacts with SF3B1 to enhance the binding of SF3B1 to the BS regions and promotes pre-mRNA splicing of some splicing- and cell proliferation-related genes; thus, it plays an important role in the pathogenesis of ALF.

This study revealed the critical role played by dysregulation of pre-mRNA splicing in the pathogenesis of ALF. Specifically, we found that KHSRP, an important regulator of pre-mRNA splicing, is downregulated aberrantly in ALF. KHSRP interacts with SF3B1 to enhance the binding of SF3B1 to the BS regions and promotes the pre-mRNA splicing of several splicing- and cell proliferation-related genes to protect against ALF (Fig. 8). Our results highlight the protective role of KHSRP in liver disease and illuminate the links between KHSRP, pre-mRNA splicing, and liver injury.

Aberrant splicing is involved in the pathogenesis of liver diseases, such as NAFLD and HCC [42–44]. The expression of several splicing factors, including SRSF10, SRSF7, and SF3A1, was decreased in either mild or advanced NAFLD [43, 45]. In a study on HCC, ~45,000 AS events from 377 liver samples were discovered by analyzing RNA-Seq data in TCGA database [46]. Using proteomic and transcriptomic analyses, we characterized 54 splicing factors, including Khsrp, Sf3a1, Phf5a, and Sf3b1, which were downregulated in chemically induced ALF models. Downregulated splicing factors, including KHSRP, SF3A1, and U2AF2, were also observed in ALF patients. Intriguingly, our results suggest that there is a high degree of intron retention in murine ALF models. Moreover, our findings indicate that dysregulation of pre-mRNA splicing is an important event in ALF, which is a new insight into the pathogenesis of ALF.

Previous studies have revealed that intron retention is a central component of gene expression programs during normal development and in response to stress and disease [47]. Several RNA-binding proteins (RBPs), such as PTBP1, hnRNPLL, and XAB2, have been identified as coregulators in various sets of intron retention [48–50]. More recently, it has been reported that far upstream bingding protein 1 (FUBP1) function as a general splicing at 3’ splice site, with a crucial role in promoting efficient splicing of long intron [51]. Our mechanistic analysis revealed that KHSRP modulates pre-mRNA splicing, especially intron retention. Consistent with previous findings that KHSRP regulates splicing via a number of intronic splicing enhancer sequences [21, 25], our transcriptome analysis adds an extra layer of complexity to intron retention induced by *KHSRP* depletion. We explored the molecular mechanisms underlying the specific regulation of pre-mRNA splicing by KHSRP, finding that KHSRP directly interacts with SF3B1 through the KH4 and C-terminal domain of KHSRP. Our data are consistent with a previous study, which showed that RBPs containing the K homology domain can regulate AS [52]. Moreover, we found that KHSRP could bind to the BS regions of several responsive genes, and the presence of KHSRP enabled a more efficient association between SF3B1 and U2 snRNA, suggesting that a common mechanism potentially underlies KHSRP-regulated splicing. The level of intron retention is reportedly higher in the nuclei than in the cytoplasm [47], which is consistent with our finding that KHSRP is more likely to be localized in the nuclei. KHSRP has been found to localize in nuclear speckles that serve as splicing factor storage sites, which further supports our results [22]. Notably, we found that KHSRP promoted the splicing of a set of splicing-related genes, including *SF3B1*, *PHF5A*, and *SF3B4*, suggesting the existence of a positive feedback loop that promotes pre-mRNA splicing. These data further strengthen the evidence that links KHSRP with pre-mRNA splicing. Collectively, these findings emphasize the important role played by KHSRP in modulating pre-mRNA splicing and expand our knowledge of KHSRP’s regulatory roles. The involvement of KHSRP in pre-mRNA splicing-mediated regulation provides a new means by which KHSRP could positively regulate many genes, and such regulation may play a critical role in the coordinated and dynamic expression of genes under the physiological and pathological conditions of multiple diseases.

Liver cell death and proliferation are considered central to the pathogenesis of ALF [4, 5]. In our study, KHSRP positively regulated cell proliferation- and apoptosis-related genes, including *EGFR* and *CDC25A*, through the promotion of pre-mRNA splicing. EGFR has been reported to prevent hepatocyte apoptosis and liver failure [41]. Downregulated KHSRP was observed not only in murine ALF models but also in ALF patients, implying that KHSRP plays a consistent role in the pathogeneses of ALF. Overexpression of Khsrp ameliorated ALF in vivo, whereas inhibition of EGFR signaling partially nullified the protective effects of Khsrp. These findings suggest that KHSRP protects against ALF partially by promoting cell proliferation and preventing apoptosis. Most previous studies, including our own, have found that KHSRP negatively regulates the expression of some inflammatory factors by promoting mRNA degradation [20, 27, 30, 53], suggesting that KHSRP protects against ALF through distinct mechanisms. We can speculate plausibly that the two levels of KHSRP-mediated regulation function dynamically to coordinate gene expression under physiological and pathological conditions in ALF. Based on these findings, we suggest that inhibiting KHSRP degradation or activating KHSRP could be potential new avenues in ALF treatment.

Molecule inhibitors targeting the spliceosome machinery are becoming promising therapeutic agents for treating cancer [14, 16]. For example, PB, an inhibitor of SF3B1, has been shown to exert antitumor activity in both cancer cell lines and mouse xenograft models [37, 38]. However, little is known about whether such splicing inhibitors are toxic to normal tissues. Our data suggest that high concentrations of PB are apparently toxic to the liver. Furthermore, we found that Khsrp protects against liver injury by regulating pre-mRNA splicing in vivo, suggesting that a complex mechanism underlies pre-mRNA splicing in liver injury. Since KHSRP plays a critical role in protecting the liver from damage, activation of KHSRP may represent an alternative approach to alleviating the toxicity of splice inhibitors in healthy tissues.

In this study, we provide compelling in vitro and in vivo evidence indicating that KHSRP plays a vital role in ALF through the regulation of pre-mRNA splicing. Our data suggest the existence of a previously unreported mechanism underlying the regulation of pre-mRNA splicing by KHSRP through its interaction with SF3B1; thus, we revealed a novel means by which liver cells potentially provide protection against acute liver disease. Furthermore, our findings imply that KHSRP-mediated regulation of pre-mRNA splicing may represent a new mechanism in the regulatory network that is likely relevant to the development of new therapeutic strategies for ALF.

## Materials and Methods

### Animals and models of ALF

Mice aged 8–11 weeks and weighing 22–25 g, were purchased from Hubei Provincial Center for Disease Control and Prevention (Wuhan, China). ALF models induced using carbon tetrachloride (CCl4) and APAP administration were established in male BALB/c mice. The mice were randomly divided into four groups (*n* = 6 each) that are treated with vehicle (olive oil or saline solution), APAP, or CCl4. APAP at 500 mg/kg (Sigma) or CCl4 at 1 ml/kg [Sigma; diluted with olive oil (1:4) before administration and filtered using a 0.22 μm filter] were intraperitoneally injected into the mice. The investigators were blinded to the group allocation during the experiment. All animal experiments were conducted in accordance with the guidelines of the animal protection and use committee of Wuhan University and the rules and regulations of Wuhan University.

### Mass spectrometry assays for liver tissues

Nine mice were randomly divided into three groups (i.e., oil-, CCl4-, and APAP-treated groups). Liver samples were collected at 24 h after injection and subjected to acetone precipitation, mass spectroscopy-grade trypsin digestion, and desalination (the latter using C18 stage tips; Thermo Fisher Scientific). The processed samples were analyzed using an Orbitrap Exploris 480 mass spectrometer equipped with the FAIMS Pro interface and an EASY-nanoLC system (Medical Research Center for Structural Biology, Wuhan University). EASY-nanoLC was configured using a Hypersil GOLD C18 Selectivity HPLC column. The HPLC gradient was prepared as follows: 2% to 8% solvent B (A = 0.1% formic acid in water; B = 80% acetonitrile, 0.1% formic acid) in 2 min, from 8% to 32% solvent B over 168 min, from 32% to 100% solvent B in 6 min, and 100% solvent B for 4 min at a flow rate of 300 nl/min. The full scan mass range was 350–1500 m/z and the Obitrap resolution was 60,000. Data were obtained using a data-dependent acquisition method and analyzed using Protein Discovery (Thermo Fisher Scientific) with a four-stage searching program. The strict target false discovery rate (FDR) for peptide-spectrum match counts or peptides was set at 0.01. Peptides with a confidence of <0.01 were excluded. For protein assembling, all proteins with a q-value of >0.01 received high confidence, whereas those with a q-value of >0.05 received medium confidence. The mass spectrometry proteomics data collected in this study have been deposited to the ProteomeXchange Consortium via the PRIDE partner repository with the dataset identifier PXD029204.

### Construction and stereotactic injection of vectors for *Khsrp*-knockdown or overexpression

Recombinant adeno-associated virus (AAV) vectors were gifts from Dr. Y.C. Xia (Wuhan University). AAV-CMV (Addgene; catalog no. 105530) was constructed to express the complete coding sequence (CDS) of *Khsrp* (AAV8-Khsrp; overexpression of KHSRP). Empty vectors were used as negative controls. The recombinant plasmids were treated using a triple-transfection helper-free method and purified according to a method published previously [54]. Briefly, HEK293T cells (ATCC) were transfected with AAV-CMV, AAV8 (Addgene; catalog no. 112864), and AAV-helper (Addgene; catalog no. 81070) plasmids in 150-mm dishes at 80% confluence. For hepatic *Khsrp*-knockdown, PX552 (Addgene; catalog no. 60958) vectors expressing a short hairpin RNA (shRNA) directed at *Khsrp* mRNA (AAV-shKhsrp; knockdown of *Khsrp*) were constructed and then verified using sequencing. PX552 vectors were used as negative controls. HEK293T cells were transfected with PX552, AAV-DJ (Addgene; catalog no. 130878), and AAV-helper (Addgene; catalog no. 81070) plasmids. GFP was used as a reporter. Virus titers were determined using quantitative PCR. The final virus in phosphate-buffered saline (PBS) had a titer of 10 × 10^10^ viral particles/ml. A previous study reported that mice were tail-vein-injected with the prepared viruses for 14 days and then subjected to CCl4 treatment for 24 h [32]. Information on the plasmids used in this study is listed in Supplementary Table 1.

### Immunoprecipitation mass spectrometry assay

Stable HL7702 cell lines expressing KHSRP (pHAGE-KHSRP-FL containing the complete CDS of *KHSRP*) were cultured for 48 h, and the cells were dissolved in cell suspension [10-mM HEPES (pH 7.9), 10-mM KCl, 1.5-mM MgCl_2_ 340-mM sucrase (~12% w/v), 10% (v/v) glycerol, 0.5-mM DTT, protease inhibitor PI (1:100), and 10-mM sodium butyrate (HDAC inhibitor from a 1-M stock solution)]. The samples were treated with an equal volume of 0.2% Triton with a final concentration of 0.1% and a final concentration of 1-mM CaCl_2_. After adding anti-KHSRP (A302-021A, BETHYL) and maintaining the samples overnight at 4 °C, A/G agarose beads were added for 4 h. After washing the beads with PBST twice, 100 μl of elusion buffer mixed with SDS loading buffer was added to each sample. Subsequently, 15 μl of supernatant was collected for gel running and silver staining was used for detection, whereas detection was conducted in the remaining samples using mass spectrometry. All acquired raw data were processed using pFind (V3.1.6) software. The peak lists were searched for in the UniProt human protein database (release 20190308). Four missed cleavages were allowed for trypsin. The precursor and fragment ion mass tolerances were both 20 ppm. The open-search algorithm in pFind was used with acetylation (Lys) set as a variable modification. The minimum peptide length was set at 6, and the estimated FDR threshold for peptides and proteins was set to a maximum of 1%. The algorithm defaults were used for all other parameters in pFind.

### RNA immunoprecipitation assay

Formaldehyde crosslinking RNA immunoprecipitation (RIP) was performed as previously described [55]. Briefly, HL7702 cells were washed twice with 10 ml of PBS and suspended in 10 ml of PBS. Formaldehyde (AR grade; Mallinckrodt; from a 37% HCHO/10% methanol stock solution) was added to a final concentration of 1% (v/v, 0.36 M) and incubated at room temperature for 10 min with slow mixing. Crosslinking reactions were quenched with the addition of glycine (pH 7.0) to a final concentration of 0.25 M, after which the cells were incubated at room temperature for 5 min. The cells were then harvested and lysed using RIPA buffer (100-mM KCl, 5-mM MgCl_2_, 10-mM HEPES-NaOH, and 0.5% NP-40) with RNase inhibitor (Promega) and protease inhibitor cocktail (Roche). The crosslinked RNA samples were fragmented using three rounds of sonication (20 s per round) in an ultrasonic homogenizer with a microprobe at an amplitude setting of 7 (output: 8–9 W). The 50-ml protein A/G beads (Sigma) were incubated with 1 mg of antibody at room temperature for 1 h in a washing buffer [50-mM Tris (pH 7.4), 150-mM NaCl, 1-mM MgCl_2_, and 0.05% NP-40]. The resulting supernatant was then incubated with an antibody conjugated with immobilized beads at 4 °C for 3 h. The same amount of immunoglobulin G (IgG) was used as a control. The protein-captured beads were washed with the washing buffer three times. RNA extraction and immunoblotting from the beads were conducted using TRIzol (Invitrogen) and loading buffer, respectively, for the subsequent detection of coimmunoprecipitated RNA and protein. PCR primers for RIP are listed in Supplementary Table 2. The antibodies used for RIP analysis were anti-KHSRP (A302-021A; BETHYL), anti-SF3B1 (sc-514655; Santa Cruz), and IgG (B900610; Proteintech).

### RNA-Seq and data analysis

Total RNA was isolated using an RNeasy Kit (Qiagen), and RNA quality was checked using an Agilent Bioanalyzer 2100. The mRNA was purified using KAPA mRNA Capture Kits (Roche), and cDNA libraries were prepared using KAPA RNA HyperPrep Kits (Roche) at Kindstar Global (China). Equal amounts of cDNA library from each sample were pooled for sequencing on an Illumina HiSeq X platform (150-bp paired-end sequencing). Samples were sequenced with a median read of 113.913107 M (range: 85.567278–130.156120 M), and the raw reads were deposited to the Gene Expression Omnibus with the accession numbers GSE179164, GSE157581, GSE171317, and GSE268435. Reads were mapped to the GRCh38/GRCm38 genome assemblies using Hisat2 v2.1.0 with the default settings. The aligned reads were converted to bigwig coverage files using reads per million. Genome annotations were extracted from ensemble GRCh38/GRCm38 Ens_96 and used to count reads with htseq-count v0.13.5. R version 3.6.0 and Deseq2 were used for differentially expressed gene analysis. Differentially spliced events were analyzed using rMATS, and significant differentially spliced events were screened using the following conditions: |ΔPSI| > 0.05 and FDR < 0.05 [13].

### Real-time PCR for detection of spliced and unspliced mRNAs

Total RNA was isolated from transfected cells using TRIzol reagent (Invitrogen), according to the manufacturer’s instructions. Reverse transcription PCR was performed using a cDNA Reverse Transcription Kit (Takara). Primers for detecting spliced and unspliced mRNAs were designed according to methods published previously [35] and are listed in Supplementary Table 2. Spliced and unspliced mRNA levels were detected using a SYBR Green Master Mix Kit (Vazyme), and fold changes were calculated using the 2^-ΔΔCt^ method. β-actin (mouse) or GAPDH (human) were used as endogenous controls.

### Fluorescence-activated cell sorting

Primary hepatocytes were isolated from mice injected with AAV-shKhsrp or PX552 (control) for 2 weeks according to methods published previously [56]. Both the AAV-shKhsrp and PX552 constructs contained GFP tags. Cell suspensions were filtered using a strainer and analyzed using an LSRFortessa flow cytometer (BD Biosciences). GFP intensity was detected in the PE channel, and data were analyzed using FlowJo V10 software. For sorting, the cells were resuspended using flow cytometry buffer (PBS with 2% fetal bovine serum) and then sorted using a FACSAria Cell Sorter (BD Biosciences) to separate GFP-positive and GFP-negative mouse primary hepatocytes.

### RNA pull-down assay

We confirmed the sequences of the BS regions in the retained introns of EGFR, SF3B1, PHF5A, and CDC25A by analyzing the eCLIP data of KHSRP in HepG2 cells [57], and we designed RNA probes for detecting these BS regions accordingly. In addition, the DEEPCLIP website (deepclip.compbio.sdu.dk/) was used to design the mutated probes for detecting the mutated BS regions [58]. The sequences of these probes are listed in Supplementary Table 2. RNAs were transcribed using T7 RNA Polymerase (Invitrogen). Biotin-labeled probes were obtained using a Pierce TM RNA 30 End Desthiobiotinylation Kit (Thermo Fisher Scientific). The 15-pM biotinylated RNA probes were incubated with 50-μl streptavidin magnetic beads for 30 min in the washing buffer. HL7702 cells were lysed using IP lysis buffer (Beyotime) with RNase inhibitor and protease inhibitor cocktail. After centrifugation at 12,000 *g* and 4 °C for 10 min, the supernatant was collected. Part of the supernatant was saved to use in immunoblotting as an input, whereas the remaining supernatant was added to the probe beads and incubated at 4 °C overnight. To harvest the proteins, the remaining beads were eluted with elution buffer (95% formamide with 5% ethylenediaminetetraacetic acid) at 95 °C for 5 min. The RNA-bound proteins were analyzed using immunoblotting.

### Statistical analysis

All experiments were performed in triplicate (at the least). The investigators were blinded to the group allocation during when assessing the outcome. The sample size was chosen to ensure adequate power to detect a prespecified effect size. All data are presented as the means ± standard error of the mean (SEM) of three experiments. Analysis was performed using Student’s t-test or ANOVA when appropriate. *P* values of <0.05 were considered significant.

### Supplementary information

Supplementary figures and tables

supplemental method

original Western blot

Supplementary tables

## Data Availability

The RNA-Seq FASTQ files were deposited in NCBI’s Gene Expression Omnibus (GEO) (https://www.ncbi.nlm.nih.gov/geo/query/acc.cgi?acc=GSE166060). (https://www.ncbi.nlm.nih.gov/geo/query/acc.cgi?acc=GSE171317), accessible password is **epehkukejjixhcl**. (https://www.ncbi.nlm.nih.gov/geo/query/acc.cgi?acc=GSE179164). The mass spectrometry proteomics data have been deposited to the ProteomeXchange Consortium via the PRIDE partner repository with the dataset identifier PXD029204. The RNA-Seq FASTQ files were deposited in NCBI’s Gene Expression Omnibus (GEO) (https://www.ncbi.nlm.nih.gov/geo/query/acc.cgi?acc=GSE268435).
