# Supplementary material for: KHSRP ameliorates acute liver failure by regulating pre-mRNA splicing through its interaction with SF3B1
Source: Cell Death Dis. 2024 Aug 26;15(8):618. doi: 10.1038/s41419-024-06886-1 (PMC11347664; doi:10.1038/s41419-024-06886-1)
Supplement: Supplementary file 1 — Supplementary figures and tables [file 41419_2024_6886_MOESM1_ESM.pdf]

Figure S1

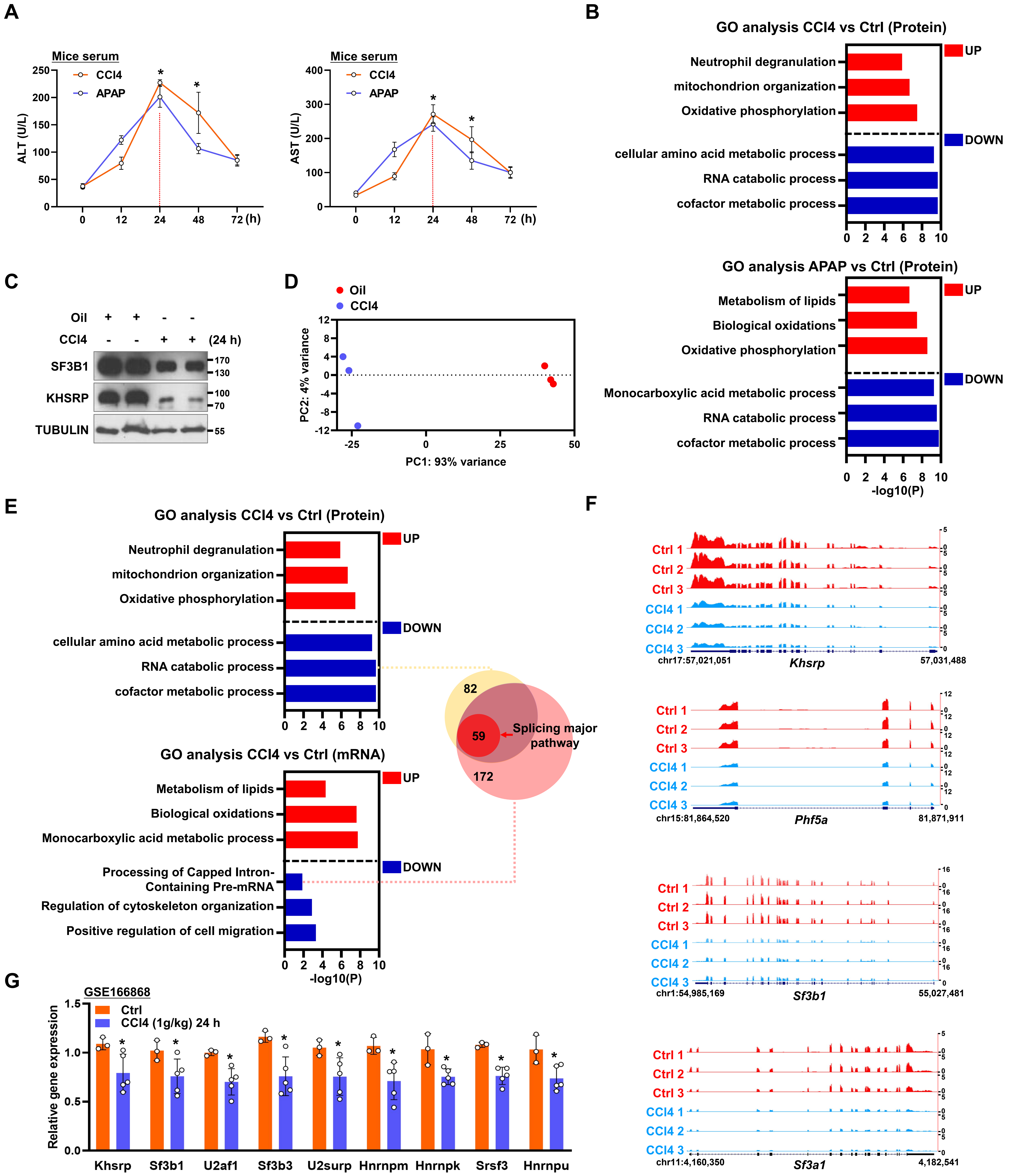

Figure S1. The downregulated pre-mRNA splicing factors in murine ALF models. (A) Plasma ALT and AST levels in mice injected with CCl4 or APAP at different time points. (B) GO analysis of MS data showing up- or downregulated proteins in CCl4- and APAP-treated mice. (C) Protein levels of Khsrp and Sf3b1 was measured in mouse livers injected with CCl4 for 24 h using western blotting. (D) Principal component analysis (PCA) was used to compare the intragroup and intergroup differences of gene in oil- and CCl4-treated mouse livers. (E) Venn diagram of downregulated proteins associated with RNA catabolic process and downregulated mRNAs related with processing of capped intron-containing pre-mRNA in the livers from CCl4-treated mice. A total of 59 splicing-related factors were downregulated in the livers from CCl4-treated mice. (F) Genome browser tracks of RNA-Seq signals at *Khsrp*, *Phf5a*, *Sf3b1*, and *Sf3a1* in oil- and CCl4-treated mouse livers. (G) The expression of splicing factors in ALF (n = 5) and controls (n = 3) in mouse samples from GSE166868. Data represent means ± SEM from three independent experiments. *$p < 0.05$.

Figure S2

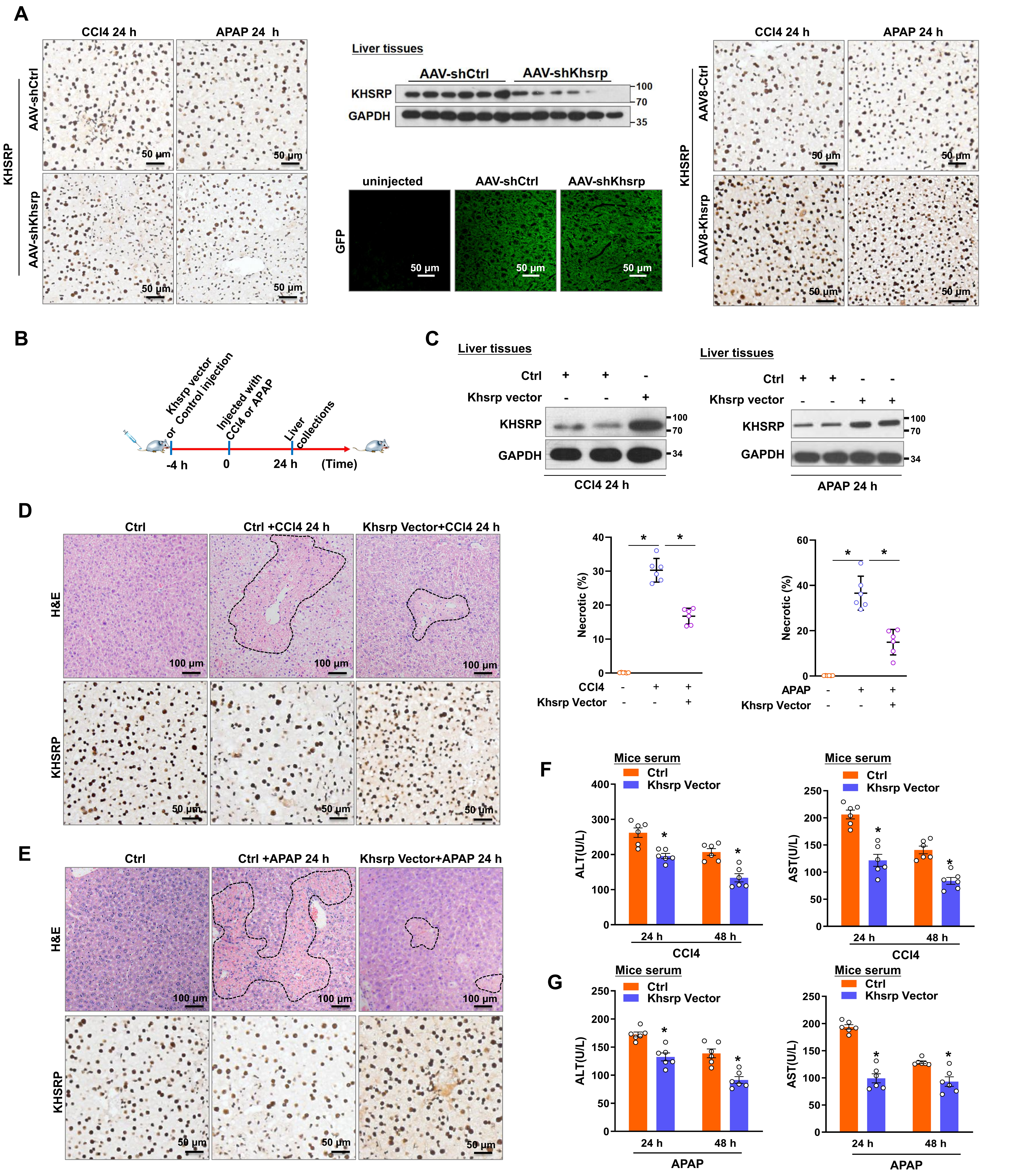

Figure S2. Khsrp protects against ALF in APAP- or CCl4-induced mice. (A) Expression of KHSRP protein was detected using immunohistochemistry in the mouse livers (n=6) following treatment with APAP or CCl4 for 24 h (scale bar: 50 μm). Western blot showing KHSRP protein levels in the liver tissues of AAV-shKhsrp or AAV-shCtrl-injected mice. Representative confocal microscopy image of GFP signal (green) in the liver tissues (middle panel) from mice infected with AAV (scale bar: 50 μm). (B) Schematic overview of the experimental setup. Mice (n = 6) were first infected with either Khsrp or control vectors for 4 h via hydrodynamic tail-vein injection. They were then intraperitoneally injected with CCl4 or APAP for additional 24 h. (C-E) Expression of KHSRP protein was detected in the injured livers after Khsrp or control vectors injection using (C) western blotting and (D, E) immunohistochemistry (scale bar: 50 μm). H&E staining of liver sections from KHSRP or control mice following treatment with (D) CCl4 or (E) APAP (scale bar: 100 μm). Necrosis quantification is shown in the right panels. (F, G) Plasma ALT and AST levels in mice. Data represent means ± SEM from three independent experiments. *$p < 0.05$.

Figure S3

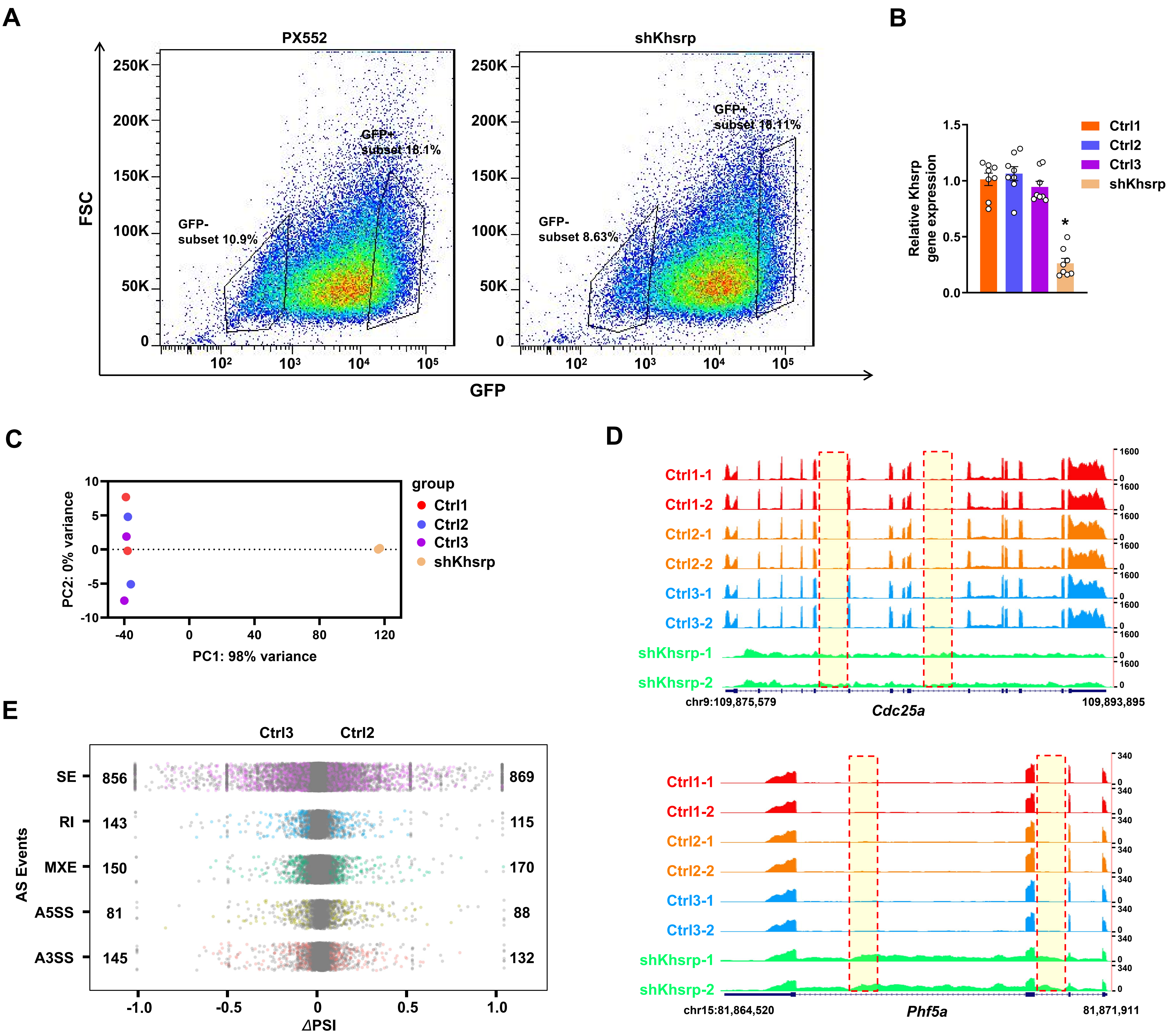

Figure S3. Khsrp regulates pre-mRNA splicing in primary hepatocytes. (A) Representative flow-cytometry plots showing the percentage of GFP (x-axis) positive and GFP negative primary hepatocytes isolated from AAV-shKhsrp- or PX552 (control)-treated mice. Hepatocytes sorted for RNA-Seq were highlighted by the boxes. (B) Expression of *Khsrp* mRNA was detected in primary hepatocytes isolated from AAV-shKhsrp- or PX552 (control)-treated mice using real-time PCR. (C) Principal component analysis (PCA) was used to compare the intragroup and intergroup differences of gene in Ctrl1, Ctrl2, Ctrl3, and shKhsrp groups. (D) Genome browser tracks of RNA-Seq signals at *Cdc25a* and *Phf5a* in the Ctrl1, Ctrl2, Ctrl3, and shKhsrp groups. Tracks of RNA-Seq signals at the intron-retained regions are shown by dotted boxes. (E) Scatter plots show changes in splicing events between Ctrl3 and Ctrl2 groups. Using rMATS, five types of AS events were analyzed: Retained introns (RIs), skipped exons, alternative 5′ and 3′ splice sites (A5SS and A3SS, respectively), and mutually exclusive exons. Significantly changed events (|ΔPSI| > 0.05, FDR < 0.05, and supporting reads ≥ 5) are shown by color dots. Data represent means ± SEM from three independent experiments. *$p < 0.05$.

Figure S4

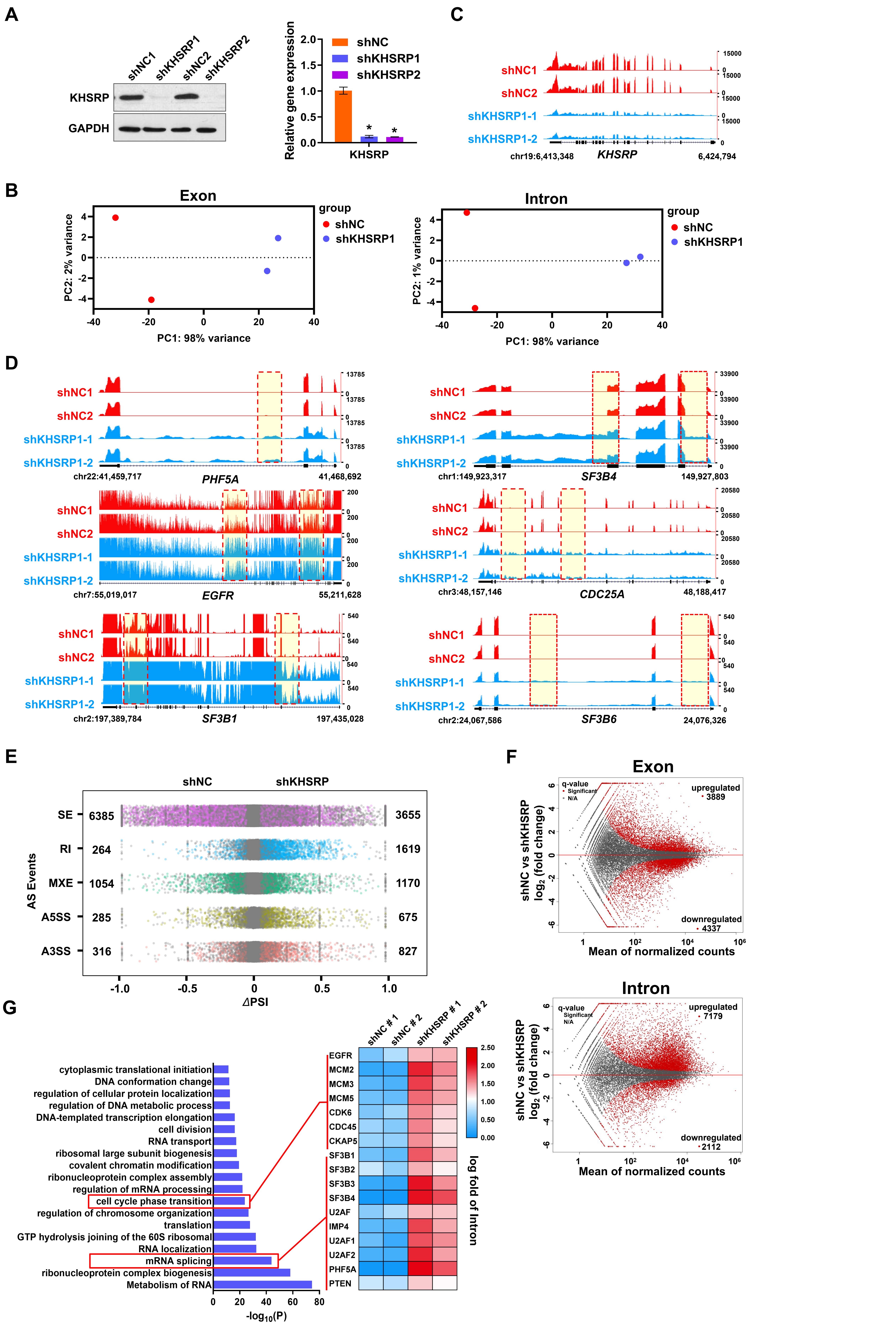
A
shNC1
shKHSRP1
shNC2
shKHSRP2
KHSRP
GAPDH
Relative gene expression
shNC
shKHSRP1
shKHSRP2
KHSRP
C
shNC1
shNC2
shKHSRP1-1
shKHSRP1-2
chr19:6,413,348
KHSRP
6,424,794
B
Exon
group
shNC
shKHSRP1
PC2: 2% variance
PC1: 98% variance
Intron
PC2: 1% variance
D
PHF5A
chr22:41,459,717
41,468,692
SF3B4
chr1:149,923,317
149,927,803
EGFR
chr7:55,019,017
55,211,628
CDC25A
chr3:48,157,146
48,188,417
SF3B1
chr2:197,389,784
197,435,028
SF3B6
chr2:24,067,586
24,076,326
E
shNC
shKHSRP
AS Events
SE
6385
3655
RI
264
1619
MXE
1054
1170
A5SS
285
675
A3SS
316
827
ΔPSI
F
Exon
q-value
Significant
N/A
upregulated
3889
downregulated
4337
shNC vs shKHSRP
log2 (fold change)
Mean of normalized counts
Intron
upregulated
7179
downregulated
2112
G
cytoplasmic translational initiation
DNA conformation change
regulation of cellular protein localization
regulation of DNA metabolic process
DNA-templated transcription elongation
cell division
RNA transport
ribosomal large subunit biogenesis
covalent chromatin modification
ribonucleoprotein complex assembly
regulation of mRNA processing
cell cycle phase transition
regulation of chromosome organization
translation
GTP hydrolysis joining of the 60S ribosomal
RNA localization
mRNA splicing
ribonucleoprotein complex biogenesis
Metabolism of RNA
-log10(P)
shNC # 1
shNC # 2
shKHSRP # 1
shKHSRP # 2
EGFR
MCM2
MCM3
MCM5
CDK6
CDC45
CKAP5
SF3B1
SF3B2
SF3B3
SF3B4
U2AF
IMP4
U2AF1
U2AF2
PHF5A
PTEN
log fold of Intron

Figure S4. KHSRP regulates pre-mRNA splicing in HL7702 cells. (A) The mRNA and protein levels of KHSRP was measured in HL7702 cells transfected with KHSRP shRNA1 or shRNA2 using real-time PCR and western blotting. (B) Principal component analysis (PCA) was used to compare the intragroup and intergroup differences of intron or exon in shNC and shKHSRP groups. (C, D) Genome browser tracks of RNA-Seq signals at (C) *KHSRP* and at (D) *PHF5A*, *SF3B1*, *EGFR*, *SF3B4*, *CDC25A* and *SF3B6* in control and *KHSRP*-knockdown HL7702 cells. Tracks of RNA-Seq signals at the intron-retained regions are shown by dotted boxes. (E) Scatter plots show changes in splicing events between shKHSRP and shNC cells. Using rMATS, five types of AS events were analyzed: Retained introns (RIs), skipped exons, alternative 5′ and 3′ splice sites (A5SS and A3SS, respectively), and mutually exclusive exons. Significantly changed events ($|\Delta PSI| > 0.05$, $FDR < 0.05$, and supporting reads ≥ 5) are shown by color dots. (F) Volcano plots showing up- or downregulated exons or introns in shKHSRP or control groups according to RNA-Seq analysis. (G) GO analysis based on RNA-Seq results showing mRNA expression from intron-retained genes in *KHSRP*-knockdown HL7702 cells (left). Heatmaps of mRNA splicing factors and cell cycle-related genes are also shown (right). Data represent means $\pm$ SEM from three independent experiments. $^*p < 0.05$.

Figure S5

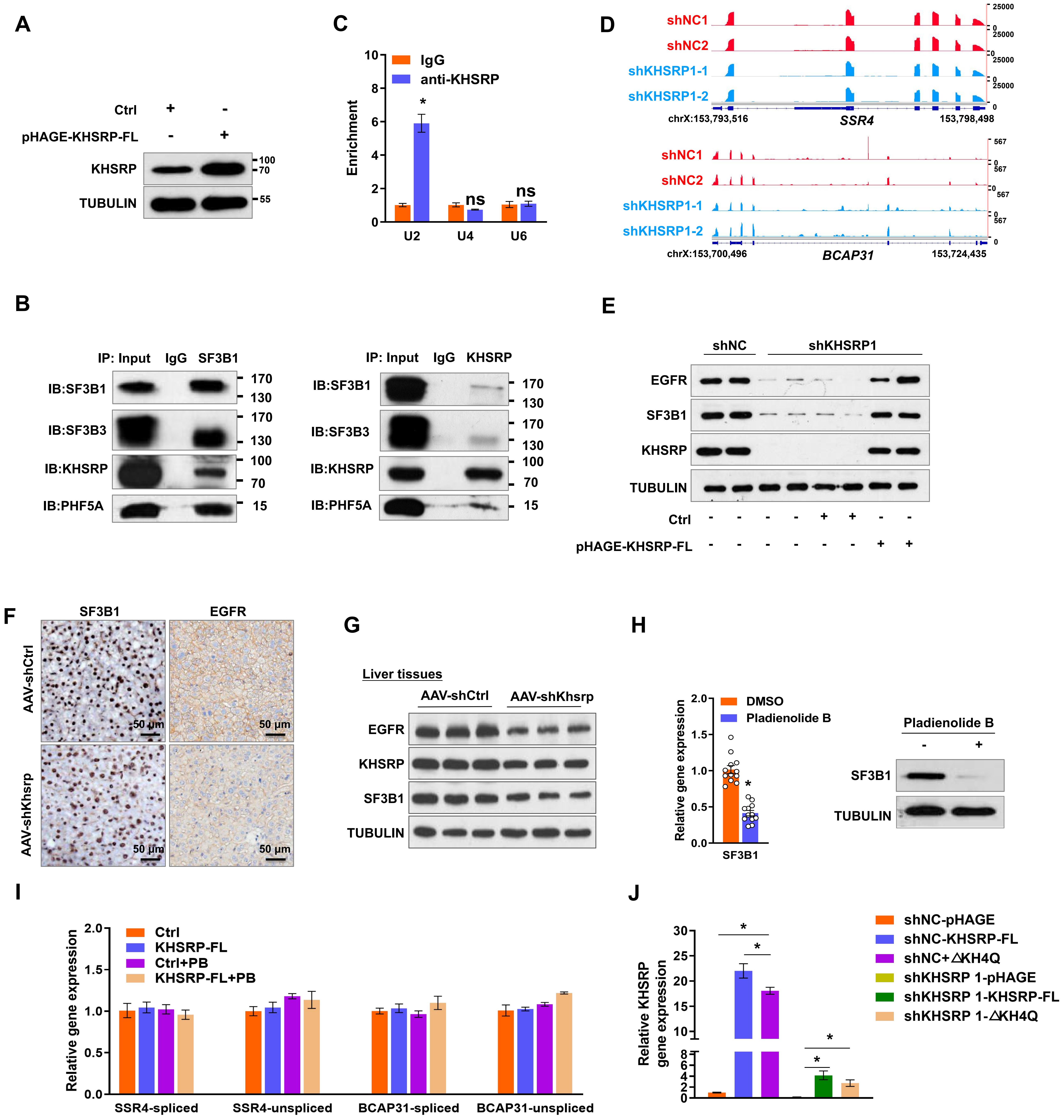

Figure S5. KHSRP promotes gene expression through pre-mRNA splicing. (A) Protein level of KHSRP was detected in HL7702 cells transfected with pHAGE-KHSRP-FL for 48 h using western blotting. (B)Coimmunoprecipitation of KHSRP and pre-mRNA splicing factors. Protein extracts from HEK293T cells were immunoprecipitated using the antibodies for KHSRP or SF3B1 and immunoblotted with the antibodies for the indicated proteins. (C) Associations of KHSRP to U2, U4, and U6 snRNAs were detected in HL7702 cells using RIP. RNAs enriched with IgG were set as 1. (D) Genome browser tracks of RNA-Seq signals at *SSR4* and *BCAP31* in control and *KHSRP* knockdown HL7702 cells. (E) Protein levels of KHSRP, SF3B1, and EGFR was detected in shNC or shKHSRP cells transfected with pHAGE-KHSRP-FL for 48 h using western blotting. (F) Expression of SF3B1 and EGFR proteins was detected in the mouse livers following AAV-shKhsrp injection using immunohistochemistry (scale bar: 50 μm). (G) Expression of EGFR, SF3B1, and KHSRP protein was detected in the injured liver after AAV-shKhsrp injection using western blotting. (H) The mRNA and protein levels of SF3B1 in HL7702 cells with or without PB. (I) The spliced or unspliced mRNAs of SSR4 and BCAP31 were detected in HL7702 cells with stable overexpression of KHSRP with or without PB. (J) pHAGE-KHSRP-FL or pHAGE-KHSRP-ΔKH4Q were transfected in shNC or shKHSRP cells. KHSRP mRNA expression was measured using real-time PCR. Data represent means ± SEM from three independent experiments. *$p < 0.05$; NS, no significant difference compared with the control.

Figure S6

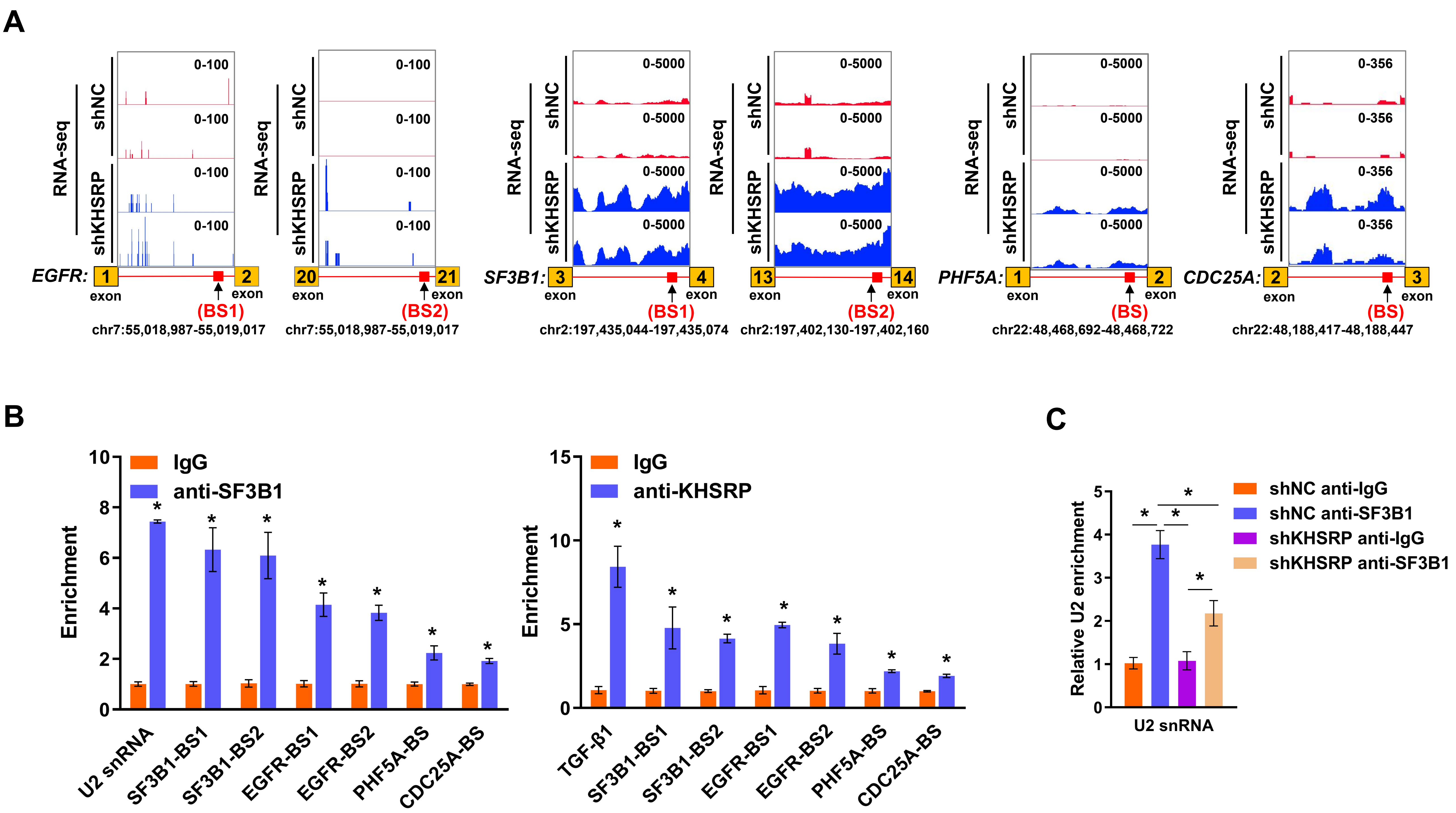

Figure S6. KHSRP promotes pre-mRNA splicing through the interaction with SF3B1. (A) The positions of BS regions on the introns of EGFR, SF3B1, PHF5A, and CDC25A. (B) Associations of KHSRP or SF3B1 with RNAs containing BS regions were detected in HL7702 cells using RIP. RNAs enriched with IgG were set as 1. The ARE regions in the TGF-β1 3′-UTR and U2 snRNA were used as positive controls. (C) Associations of SF3B1 to U2 snRNA were detected in HL7702 cells using RIP. RNAs enriched with IgG were set as 1. Data represent means ± SEM from three independent experiments. *$p < 0.05$.

Figure S7

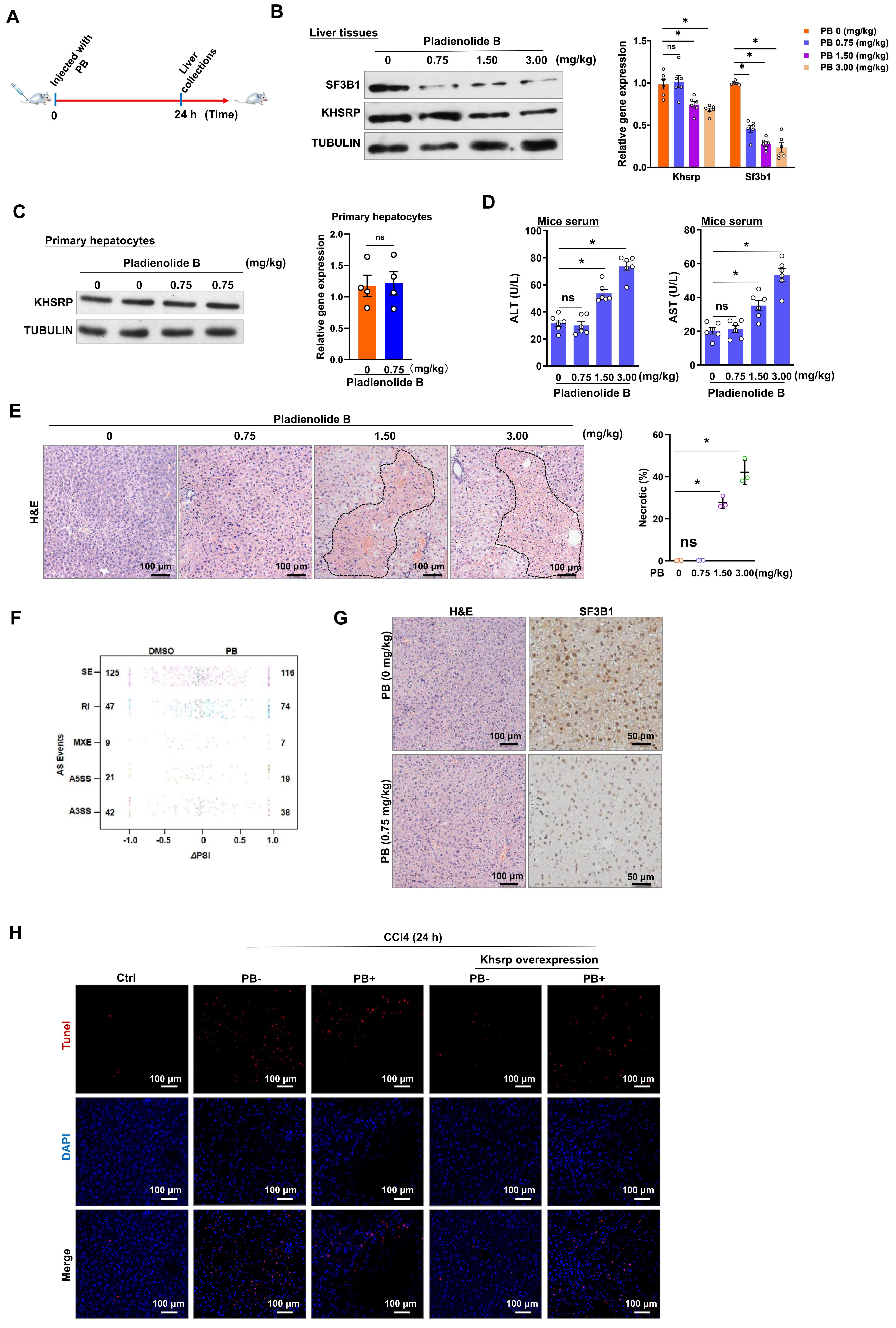

A
Injected with PB
Liver collections
0
24 h (Time)
B
Liver tissues
Pladienolide B
0 0.75 1.50 3.00 (mg/kg)
SF3B1
KHSRP
TUBULIN
Relative gene expression
Khsrp
Sf3b1
PB 0 (mg/kg)
PB 0.75 (mg/kg)
PB 1.50 (mg/kg)
PB 3.00 (mg/kg)
C
Primary hepatocytes
Pladienolide B (mg/kg)
0 0 0.75 0.75
KHSRP
TUBULIN
Primary hepatocytes
Relative gene expression
0 0.75 (mg/kg)
Pladienolide B
D
Mice serum
ALT (U/L)
0 0.75 1.50 3.00 (mg/kg)
Pladienolide B
Mice serum
AST (U/L)
0 0.75 1.50 3.00 (mg/kg)
Pladienolide B
E
Pladienolide B
0 0.75 1.50 3.00 (mg/kg)
H&E
100 μm
Necrotic (%)
PB 0 0.75 1.50 3.00(mg/kg)
F
DMSO PB
SE 125 116
RI 47 74
MXE 9 7
A5SS 21 19
A3SS 42 38
AS Events
ΔPSI
G
H&E SF3B1
PB (0 mg/kg)
PB (0.75 mg/kg)
100 μm
50 μm
H
CCl4 (24 h)
Khsrp overexpression
Ctrl PB- PB+ PB- PB+
Tunel
DAPI
Merge
100 μm

Figure S7. The effect of PB treatment on mouse livers. (A) Schematic diagram of the experimental setup. Mice (n = 3) were intraperitoneally injected with PB for 24 h (B) Protein and mRNA levels of KHSRP and SF3B1 in mouse livers treated with PB for different dose according to Western blotting and real-time PCR. (C) Protein and mRNA expression of KHSRP in primary hepatocytes isolated from mouse livers treated with PB (0.75 mg/kg). (D) Plasma ALT and AST levels in mice. (E) H&E staining of liver sections from mice treated with PB (scale bar: 50 μm). Necrosis quantification is shown in the lower panels. (F) Scatter plots show changes in splicing events between DMSO and PB-treated groups.Using rMATS, five types of AS events were analyzed: Retained introns (RIs), skipped exons, alternative 5′ and 3′ splice sites (A5SS and A3SS, respectively), and mutually exclusive exons. Significantly changed events ($|\Delta PSI| > 0.05$, FDR < 0.05, and supporting reads ≥ 5) are shown by color dots. (G) H&E staining of liver sections from mice induced by PB (0.75 mg/kg) for 24 h (scale bar: 100 μm). Expression of SF3B1 proteins was detected using immunohistochemistry (scale bar: 50 μm). (H) Apoptosis of hepatocytes in the mouse liver sections was determined by TUNEL staining (red signal). DAPI was used to stain muclei (blue signal) (scale bar: 100 μm). Data represent means ± SEM from three independent experiments. *$p < 0.05$; NS, no significant difference compared with the control.

Figure S8

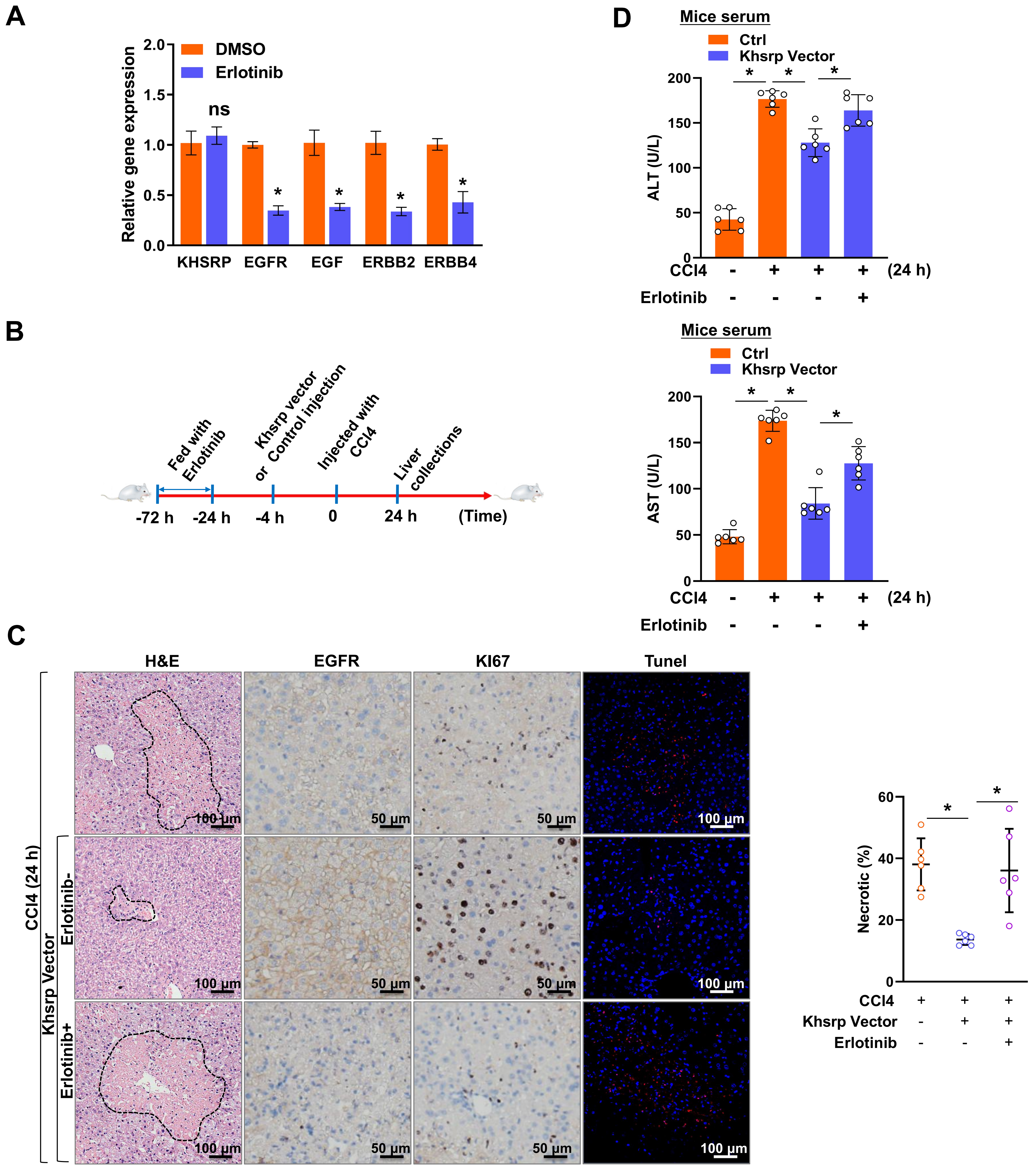

Figure S8. Khsrp protects against ALF partially through regulating Egfr. (A) mRNA levels of *EGF*, *KHSRP*, *EGFR*, *ERBB2*, and *ERBB4* transcripts were detected in HL7702 cells with or without erlotinib using real-time PCR. (B) Schematic diagram of the experimental setup. Mice (n = 6) were first treated with erlotinib orally for 3 days. They were then infected with Khsrp vectors or controls for 4 h via hydrodynamic tail-vein, after which they were intraperitoneally injected with CCl4 for an additional 24 h. (C) H&E staining of liver sections from Khsrp overexpression mice induced with CCl4 with or without erlotinib (scale bar: 100 μm). Necrosis quantification is shown in the right panels. Expression of EGFR and KI67 proteins was detected using immunohistochemistry (scale bar: 50 μm). Immunohistochemistry analysis and quantification of a TUNEL assay (red) in the liver sections (scale bar: 100 μm). (D) Plasma ALT and AST levels in mice. Data represent means ± SEM from three independent experiments. *p < 0.05 ; NS, no significant difference compared with the control.

Supplementary Table 1. Detailed information of the plasmids

**Plasmids**	**Inserted gene fragment**	**Vector**	**TAG**	Sense primer (5′-3′)	Antisense primer (5′-3′)
pHAGE-KHSRP-FL	KHSRP CDS full length	pHAGE	3xFlag	TTTgctagcATGTCGGACTACAGCACGGGAGGAC	TTTctcgagTTGAGCCTGCTGCTGTCCCTGCTGC
pHAGE-KHN123/pHAGE-ΔKH4Q	KHSRP CDS (1-435)	pHAGE	3xFlag	TTTgctagcATGTCGGACTACAGCACGGGAGGAC	TTTctcgagCTTGTGAGTGGGGATGGAGAAGGTC
pHAGE-KHN12	KHSRP CDS (1-323)	pHAGE	3xFlag	TTTgctagcATGTCGGACTACAGCACGGGAGGAC	TCTctcgagTCCGCCAATCCGAGATCCGTACTCA
pHAGE-KHN1	KHSRP CDS (1-281)	pHAGE	3xFlag	TTTgctagcATGTCGGACTACAGCACGGGAGGAC	TTTctcgagTTTGTCCACATTCGTATTCTGAGAT
pHAGE-KH123	KHSRP CDS (130-435)	pHAGE	3xFlag	TTTgctagcAGTTCTCAACTTGGACCCATCCATC	TTTctcgagCTTGTGAGTGGGGATGGAGAAGGTC
pHAGE-KH4Q	KHSRP CDS (435-710)	pHAGE	3xFlag	TTTgctagcTGTGGGCTGGTCATCGGCCGAGGTG	TCTctcgagTTGAGCCTGCTGCTGTCCCTGCTGC
pHAGE-SF3B1-FL	SF3B1 CDS full length	pHAGE	HA	TTTgctagcATGGCGAAGATCGCCAAGACTCACG	TTTctcgagTTATAAGATATAGTCAAGTTCATAA
pHAGE-SF3B1-1	SF3B1 CDS (1-484)	pHAGE	HA	TTTgctagcATGGCGAAGATCGCCAAGACTCACG	TTTctcgagTTCATCAACATCAACCAATAGTTTA
pHAGE-SF3B1-2	SF3B1 CDS (484-955)	pHAGE	HA	TTTgctagcTCAACACTTAGTCCAGAAGAGCAAA	TTTctcgagAATCAAGTCAGCTGCCTGTTGCCTA
pHAGE-SF3B1-3	SF3B1 CDS (955-1304)	pHAGE	HA	TTTgctagcTCTCGAACTGCTGTTGTCATGAAGA	TTTctcgagTTATAAGATATAGTCAAGTTCATAA
KHSRP-KH4Q	KHSRP CDS (435-710)	PET-15B	His	TTTcatatgTGTGGGCTGGTCATCGGCCGAGGTG	TCTggatccTTGAGCCTGCTGCTGTCCCTGCTGC
SF3B1-SF3B1-3	SF3B1 CDS (955-1304)	PET-15B	His	TTTcatatgTCTCGAACTGCTGTTGTCATGAAGA	TCTggatccTTATAAGATATAGTCAAGTTCATAA
AAV8-Khsrp	m-Khsrp CDS full length	AAV-CMV	GFP	TTCTaagcttATGTCGGACTACAACACCGGAGGTC	TTTgaattcAGGGCACACCCCGCAGGGGAAAGG G
AAV-shKhsrp	m-Khsrp shRNA	PX552	GFP	CCGGCCCTGAGAAGATTGCTCACATCTCGAGATGTGAGCAATCTTCTCAGGGTTTTTG	
Khsrp Vector	m-Khsrp CDS full length	pcDNA3.1+	N/A	TTTaagcttATGTCGGACTACAACACCGGAGGTC	TTTgaattcAGGGCACACCCCGCAGGGGAAAGG G

Restriction enzyme sites were indicated by lowercase letters.

Supplementary Table 2. Primers and shRNA sequences

Gene symbol	Sense primer (5′-3′)	Antisense primer (5′-3′)
**primers for real-time PCR**		
KHSRP	CTCCTGATTTTGGTTTTG	TCCTGTTGGATTTTGTTA
spliced-EGFR	ACCTGCTCAACTGGTGTGT	TCCTTCTGCATGGTATTCTT
spliced-SF3B1	GAGTGGGCCTCGATTCTAC	TGATATCCTGGCTTCTTCTGA
spliced-SF3B3	AAGAAACCTTTGGCAAGAG	GATGAAATGGTAAGTCGGG
spliced-CDC25A	CTTTGTCTGATGAGGATGATG	CTACACAGGGAAGGGGAGT
spliced-PHF5A	ACTCCTATGTGCGTCCCTG	TTGAAGCCGTATTTTTTGC
spliced-SSR4	TGAGACCGTCTTCATTGTG	ATTCCTCTGAGCCTTCCTG
spliced-BCAP31	CGGAGGTCTTTGTTGTGTT	GGATTGTTCTGGAGGTTCA
unspliced-EGFR	GCATCTGCCTCACCTCCAC	TCCCTTCCCTGATTACCTTTG
unspliced-SF3B1	TCATTTTGAATCTTTGCAT	CTGTTCTGTTGACTGTGGTA
unspliced-SF3B3	GATCTTTATTTTTTGGTGATC	ACAATGTAGTCTTTGGTGC
unspliced-CDC25A	TCTGAAAAGCAGAAAGTGT	CAAAGGAGAAAAAAGGAGT
unspliced-PHF5A	GTGCGCATATGTGATGAGTG	CAGTGGATGGCAAAGTGTT
unspliced-SSR4	TTTCCTTGTCTGCCCATACGA	CTAACTCGCCCCGCTCTTG
unspliced-BCAP31	GTCGGGAATGCTGAGGTGA	CGCAGGGTGCTATTGGTCT
m-Khsrp	TGGTGGGCTTGATTATTGG	TTGGACAGACTCAGGTGCT
GAPDH	CTCATGCGCTGTGTGGAA	GAAAATGGGAAACTGGCT
U6	ATGCTTGCCTCAGTAGCACACA	AATATGGAATGCTTCACAAAT

**Sequences for shRNA**		
NC	TTCTCCGAACGTGTCACGTT	
KHSRP 1	CCGGCCCGAGAAGATTGCTCATATACTCGAGTATATGAGCAATCTTCTCGGGTTTTTG	
KHSRP 2	CCGGCTGAGGATAAAGCAATTCATTCTCGAGAATGAATTGCTTTATCCTCAGTTTTTG	
m-Khsrp 1	CCGGCCCTGAGAAGATTGCTCACATCTCGAGATGTGAGCAATCTTCTCAGGGTTTTTG	
m-Khsrp 2	CCGGGCTTGGGAAGAGTATTACAAACTCGAGTTTGTAATACTCTTCCCAAGCTTTTTG	
**Sequences for RIP-PCR**		
EGFR-BS1	CTCATGCGCTGTGTGGAA	GAAAATGGGAAACTGGCT
EGFR-BS2	CCCCTCTCACACCAAATG	TCCCTCAAAACACAGTCC
SF3B1-BS1	ACACTCACTCCACAACCCAAG	GTGGTTCAAGAGGAAAAGCAAT
SF3B1-BS2	TATGACTCCCGGCTGAACAAG	AGCAAGCAGAAACAGGCAAAT
PHF5A-BS	GCAAGTCATTGGATAGAG	TGGGTAACAAGAGAGAAA
U2 snRNA	ATCGCTTCTCGGCCTTTTGG	TAAGGGACTCTTGAAAATAT
U4 snRNA	AGTTTTGTGCAGTGGCAGTA	CCAGAGTGCTGGGATTATAGG
U6 snRNA	ATGCTTGCCTCAGTAGCACACA	AATATGGAATGCTTCACAAAT
**Sequences of probes for RNA Pulldown**	**Sense (Mutation sites to BSs were indicated by underline)**	**Anti-sense**
EGFR-BS1	UGCAUUUCUCAGUAUUUCAUGUGAUAUCUGU	UGUCUAUAGUGUACUUUAUGACUCUUUACGU
EGFR-BS2	AUCUGUCCCUCACAGCAGGGUCUUCUCUGUU	UUGUCUCUUCUGGGACGACACUCCCUGUCUA
EGFR-Mut1	UGCAUUUCUCAGUAUUUUCGGUGAUAUCUGU	/
EGFR-Mut2	AUCUGUCCCUCGACGCAGGGUCUUCUCUGUU	/
EGFR-NBS	GGCGCGCACCCGAGGGGCGGGCGCUGCCCACCCGCCG	/
SF3B1-BS1	UGGAUAUUAAGUGAGACAUACCUCUUUUCAG	GACUUUUCUCCAUACAGAGUGAAUUAUAGGU
SF3B1-BS2	GGGCAUAGUUAAAACCUGUGUUUGGUUUUG	GUUUUGGUUUGUGUCCAAAAUUGAUACGGG
SF3B1-Mut1	UGGAUAUUAAGUGAGGACUACCUCUUUUCAG	/
SF3B1-Mut2	GGGCAUAGUUAAGCACUGUGUUUGGUUUUG	/
SF3B1-NBS	AAGGUUUUGCUGUCUUGCUCAGGCUGAUCCCAA	/
PHF5A-BS	UAAUUGUACUUUUUACCAUCUUUCAUCUUG	GUUCUACUUUCUACCAUUUUUCAUGUUAAU
PHF5A-Mut1	UAAUUGUACUUUUUGAUAUCUUUCAUCUUG	/
CDC25A-BS	UGUAUUAUUUCUUGAGAGGUUUUUUUUUUCC	CCUUUUUUUUUUGGAGAGUUCUUUAUUAUGU
CDC25A-Mut1	UGUAUUAUUUCUUGGACGGUUUUUUUUUUCC	/
