# Supplementary material for: KHSRP ameliorates acute liver failure by regulating pre-mRNA splicing through its interaction with SF3B1
Source: Cell Death Dis. 2024 Aug 26;15(8):618. doi: 10.1038/s41419-024-06886-1 (PMC11347664; doi:10.1038/s41419-024-06886-1)
Supplement: Supplementary file 2 — supplemental method [file 41419_2024_6886_MOESM2_ESM.pdf]

Supplemental Experimental Methods

Cell lines and reagents

HL7702 (human hepatocyte cell line) and 293T cells were obtained from ATCC. All cell lines were maintained in Dulbecco's modified Eagle's medium (DMEM) supplemented with 10% fetal bovine serum (FBS) and cultured in a humidified incubator at 37°C with 5% CO_2. Pladienolide B (TOCRIS bioscience) was used at a final concentration of 1 uM for cells or 0.75 mg/kg for mice. Erlotinib Hydrochloride (Absin, Shanghai) was used at a final concentration of 5 μM for cells or 100 mg/kg for mice. These reagents at the working concentration showed no cytotoxic effects on cells in this study.

Hydrodynamic tail-vein injection

Hydrodynamic injection was performed as previously described (1,2). Briefly, constructed pcDNA3.1-Khsrp (contain complete mouse *Khsrp* CDS) and pcDNA3.1 were suspended in saline solution and subsequently injected into the lateral tail veins of male or female mice (0.1 ml/g body weight) in less than 7 seconds. The information of pcDNA3.1-Khsrp was listed in Supplemental Table 2.

Western blot

Proteins were extracted from cells or tissues with RIPA lysis buffer (Servicebio) and quantified by the Bradford method (Sangon). Cell lysates were then loaded in SDS-PAGE gel to separate proteins and transferred to nitrocellulose membrane.

Antibodies to KHSRP (Bethyl, A302-021A), SF3B1 (Santa cruz Biotechnology, 618426), TUBULIN (Proteintech, 66031-1-Ig), EGFR (ZENBIO, 510673), PHF5A (Proteintech, 15554-1-AP), CDC25A (Invitrogen, DCS-120), SF3B3 (Proteintech, 14577-1-AP), FLAG (Proteintech, 66008-2-Ig), HA (Proteintech, 66006-1-Ig), SSR4 (Proteintech, 11655-2-AP) and GAPDH (Proteintech, 60004) were used for blotting.

Serum measurements

The levels of alanine aminotransferase (ALT) and aspartate aminotransferase (AST) in serum were measured by using ALT Assay Kit and AST Assay Kit (Nanjing Jiancheng Bioengineering Institute).

Plasmids and short hairpin RNAs

All the plasmids used in this paper were generated using the Gibson assembly cloning method. The cDNA sequences of KHSRP CDS full length, KHSRP CDS (1-435), KHSRP CDS (1-323), KHSRP CDS (1-281), KHSRP CDS (130-435), and KHSRP CDS (435-710) were cloned into pHAGE-3 × Flag-puro vectors. The cDNA sequences of SF3B1 CDS full length, SF3B1 CDS (1-484), SF3B1 CDS (484-955), and SF3B1 CDS (955-1374) were cloned into pHAGE-1 × HA-puro vectors. The KHSRP CDS (435-710) and SF3B1 CDS (955-1374) were cloned into pET-15b-3xHIS for HIS pulldown assay. The cDNA sequences of mouse Khsrp CDS were delivered into pCDNA3.1 (+) vector. The 3'UTR of TGF-β1, SF3B1, EGFR, PHF5A and CDC25A sequences were cloned into pMIR-GLO reporter vectors. All constructs were

verified by full-length sequencing. Lentiviral vectors encoding human *KHSRP* and mouse *Khsrp*, and scrambled control shRNA were obtained from Era Biotech. The information of plasmids and shRNA sequences were shown in Supplementary Table 1-2.

Generation of KHSRP knockdown and overexpression cells

For lentiviral stock preparation, 293T cells were cotransfected with pHAGE-KHSRP-FL or shKHSRP plasmid and three packaging plasmids (pGag-Pol, pRev, and pVSVG). Supernatants containing packaged lentivirus were collected after 48 h, passed through a 0.45 μm filter and added to HL7702 cells along with 1 mg/ml polybrene (Sigma, H9268). Cells were selected using purinomycin (1μg/ml).

Luciferase assay

Transient transfections of stable KHSRP overexpression cells or control cells were accomplished by plating cells at a density of 1×10^5 cells per well of a 24-well culture plate 24 h before transfection. The pMIR-GLO reporter vectors containing the 3′ UTRs of TGF-β1, SF3B1, EGFR, PHF5A, and CDC25A were transfected using 1 μl of Lipofectamine 2000 transfection reagent (Invitrogen) following instructions. After 24 h transfection, cells were harvested in Reporter lysis buffer (Progema), and the luciferase activity was determined by Luciferase assay system (Progema). The firefly luciferase activity was normalized to the Renilla luciferase activity.

Histology, Immunohistochemistry, and Apoptosis assay

Liver samples were fixed overnight in 4% formalin at room temperature, continuously dehydrated in ethanol, embedding with paraffin. Paraffin sections were stained with Meyer hematoxylin and stained with eosin. Representative areas of each stained tissue section were imaged at ×40 magnification. For immunohistochemistry, tissue sections were incubated with the antibodies against EGFR (510673, ZENBIO), SF3B3 (14577-1-AP, Proteintech), SF3B1 (sc-5a4655, Santa Cruz), Ki-67 (511390, ZENBIO) and PHF5A (15554-1-AP, Proteintech) for 1 h at room temperature. These slides were then subjected to horseradish peroxidase-linked secondary antibodies for 1 h at room temperature. The sections were visualized by using the DAB substrate kit (Vector Labs) and representative areas of each stained tissue section were imaged at ×100 magnification. ImageJ software was used to quantify the staining results.

Apoptosis was detected by TUNEL staining in liver tissues. TUNEL staining was performed using the TUNEL Assay kit (Elabscience) according to the manufacturer's instructions, with apoptotic cells exhibiting red nuclear fluorescence. Liver sections were counterstained with propidium iodide (DAPI) for 5-10 min to stain cell nuclei. Finally, the fluorescent images were captured by using the Zeiss LSM 880 Confocal Microscope (Medical Research Center for Structural Biology, Wuhan University).

Co-immunoprecipitation (Co-IP)

A total of 1×10^6 cells for each immunoprecipitation were lysed with IP lysis buffer (Beyotime Biotechnology, Shanghai) on ice for 30 min and then sonicated in an

ice-water bath. Cell lysates were incubated with the antibody overnight at 4°C. Before incubated with the cell lysate, the A/G agarose beads were treated with 5% BSA. The immunoprecipitates (IP) were analyzed for the presence of SF3B1 (sc-5a4655, Santa Cruz) and KHSRP (A302-021A, BETHYL) by Western blot.

His pulldown assays

Expression of recombinant His-tagged SF3B1-SF3 and His-tagged KHSRP-KH4Q proteins were induced in BL21 cells at 28°C for 24 h by 0.1 mM isopropyl β-D-1-thiogalactopyranoside (IPTG). Bacterial cells were lysed using a lysis buffer containing 50 mM Tris-Cl (pH 7.4), 150 mM NaCl, and 0.5% Triton X-100. Recombinant proteins were then purified using HisPur™ Ni-NTA Resin (88221, ThermoFisher). Protein concentrations were determined by measuring their optical density absorbance at 280 nm. The 100 μl His-tagged SF3B1-SF3 was mixed with 100 μl His-tagged KHSRP-KH4Q in 0.5 ml binding buffer (50 mM Tris-Cl, pH 7.4, 150 mM NaCl, and 0.5% Triton X-100). The binding reaction was performed with specific antibody for KHSRP (55409-1-AP, Proteintech) or SF3B1 (27684-1-AP, Proteintech) overnight at 4 °C, and the beads were subsequently washed three times with the binding buffer. Proteins were analyzed by SDS-PAGE and identified by antibody for KHSRP (55409-1-AP, Proteintech) and SF3B1 (27684-1-AP, Proteintech).

REFERENCES

1. Xiao, P., Li, M., Zhou, M., Zhao, X., Wang, C., Qiu, J., Fang, Q., Jiang, H., Dong, H. and Zhou, R. (2021) TTP protects against acute liver failure by regulating CCL2 and CCL5 through m6A RNA methylation. *JCI insight*, **6(23)**, e149276.
2. Yu, S. and Vernia, S. (2020) A Transposon-Based Mouse Model of Hepatocellular Carcinoma via Hydrodynamic Tail Vein Injection. *Methods in molecular biology (Clifton, N.J.)*, **2164**, 129-143.
