# Supplementary material for: KHSRP ameliorates acute liver failure by regulating pre-mRNA splicing through its interaction with SF3B1
Source: Cell Death Dis. 2024 Aug 26;15(8):618. doi: 10.1038/s41419-024-06886-1 (PMC11347664; doi:10.1038/s41419-024-06886-1)
Supplement: Supplementary file 3 — original Western blot [file 41419_2024_6886_MOESM3_ESM.pdf]

Figure 2

C

Liver tissues

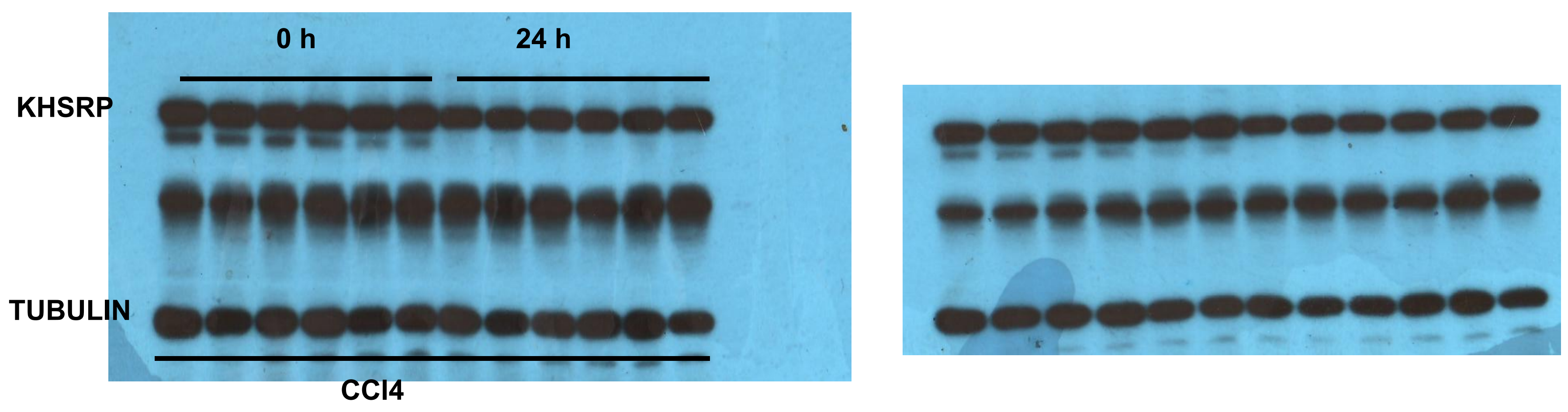

Antibody messages

Khsrp (A302-021A, BETHYL)

αTubulin (66031-1-Ig, Proteintech)

Liver tissues

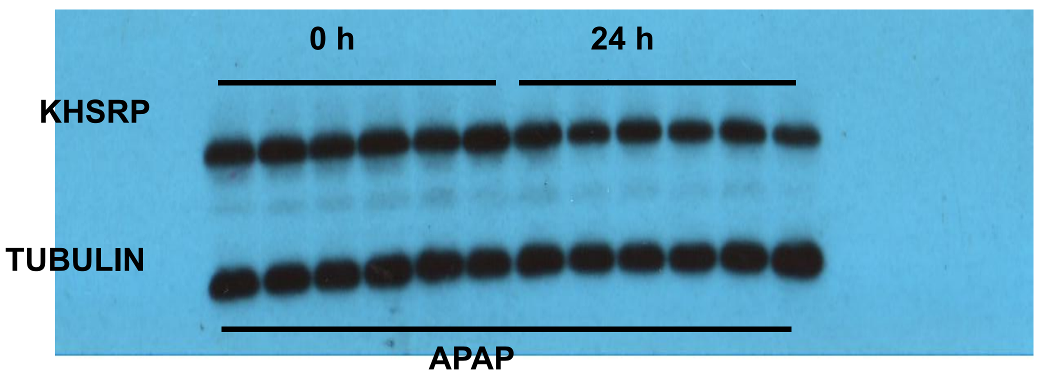

Antibody messages

Khsrp (A302-021A, BETHYL)

αTubulin(66031-1-Ig, Proteintech)

J

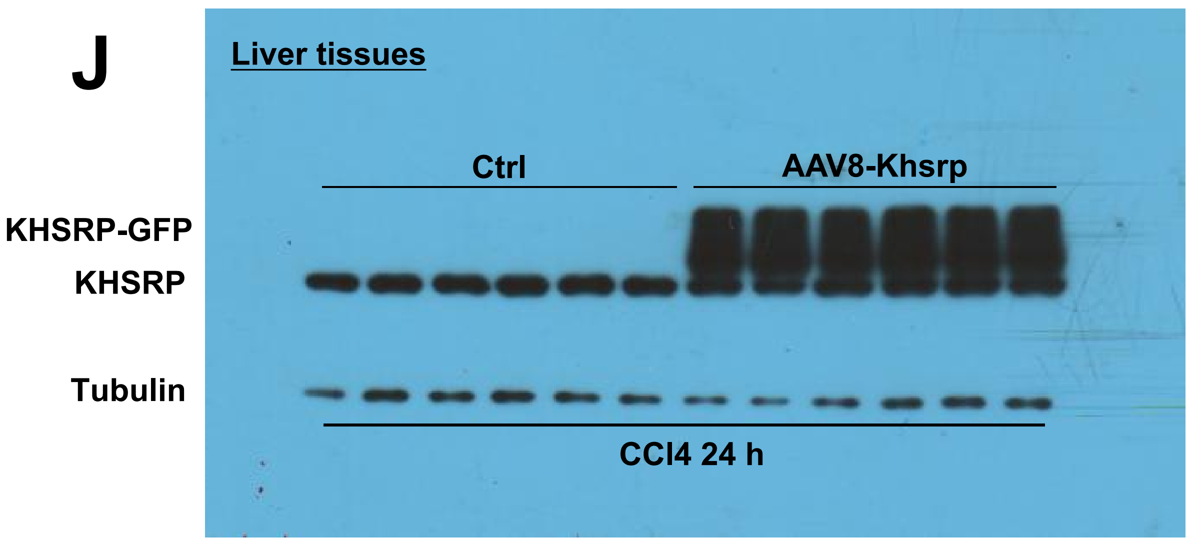

Antibody messages

KhsrpA302-021A, BETHYL)

αTubulin(66031-1-Ig, Proteintech)

Liver tissues

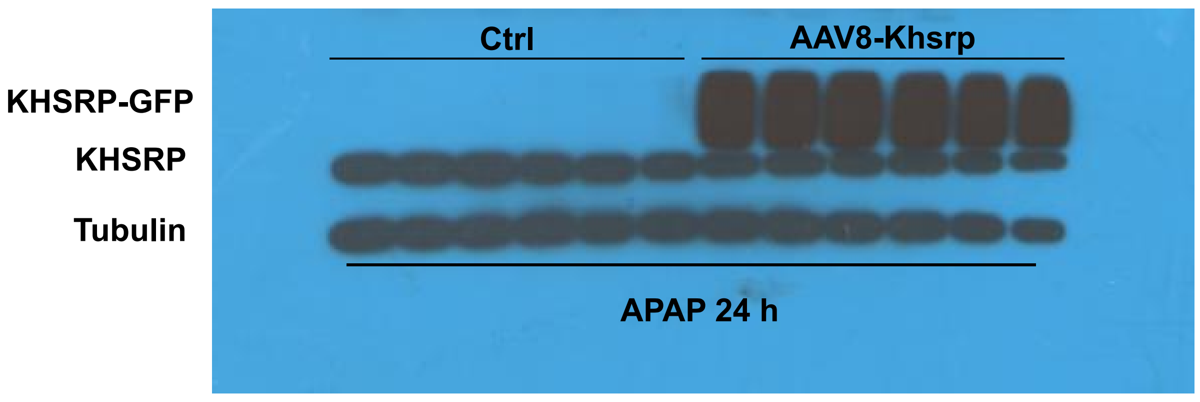

Antibody messages

Khsrp (A302-021A, BETHYL)

αTubulin(66031-1-Ig, Proteintech)

F

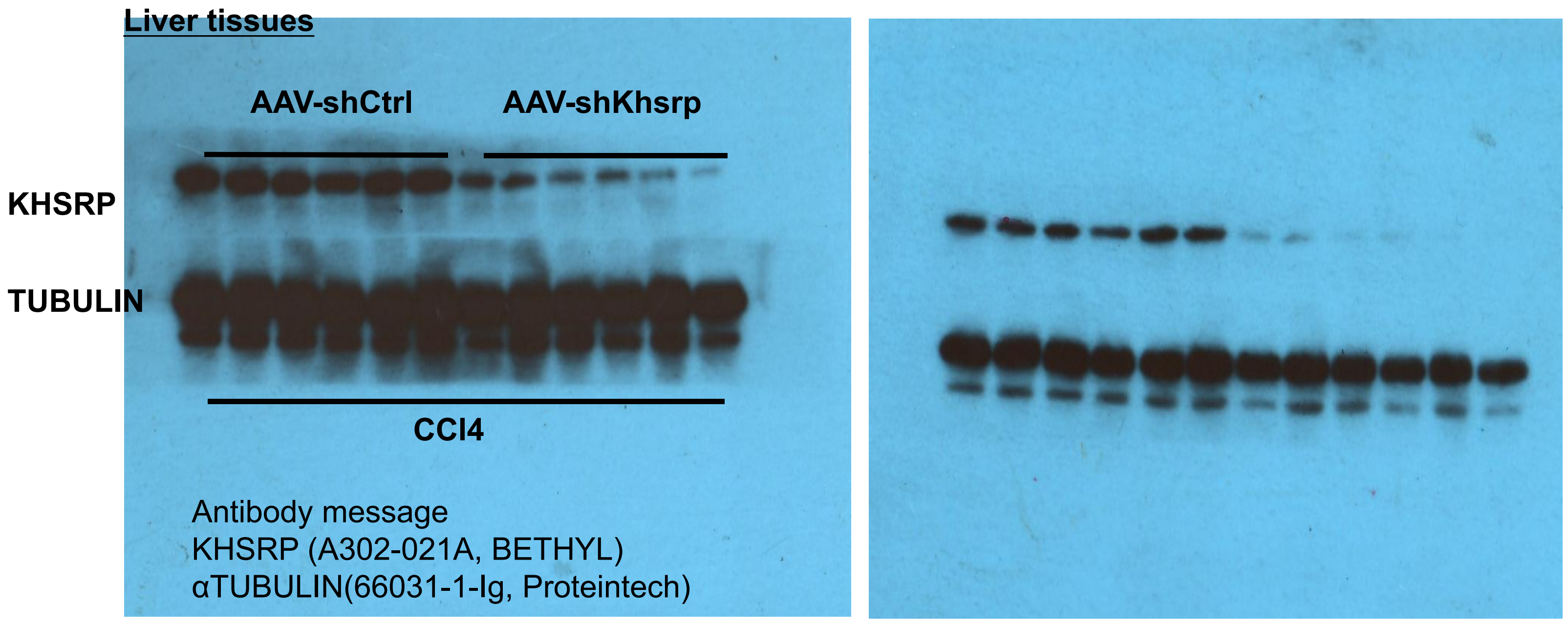

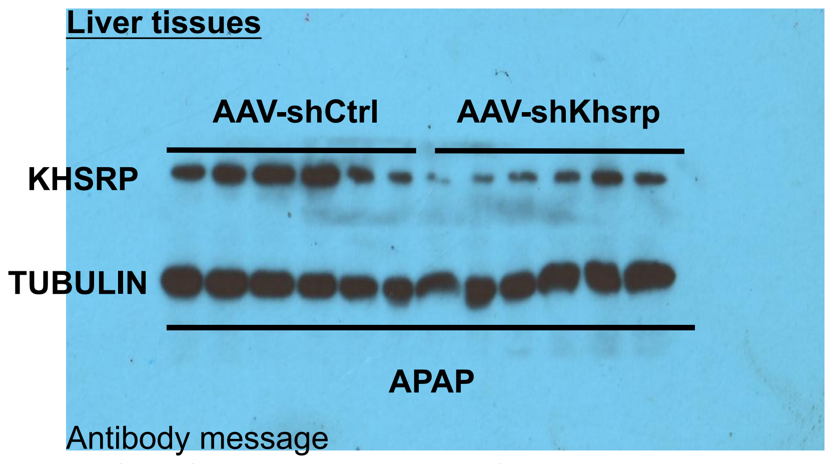

Antibody message
KHSRP (A302-021A, BETHYL)
αTUBULIN(66031-1-Ig, Proteintech)

Figure 4

A

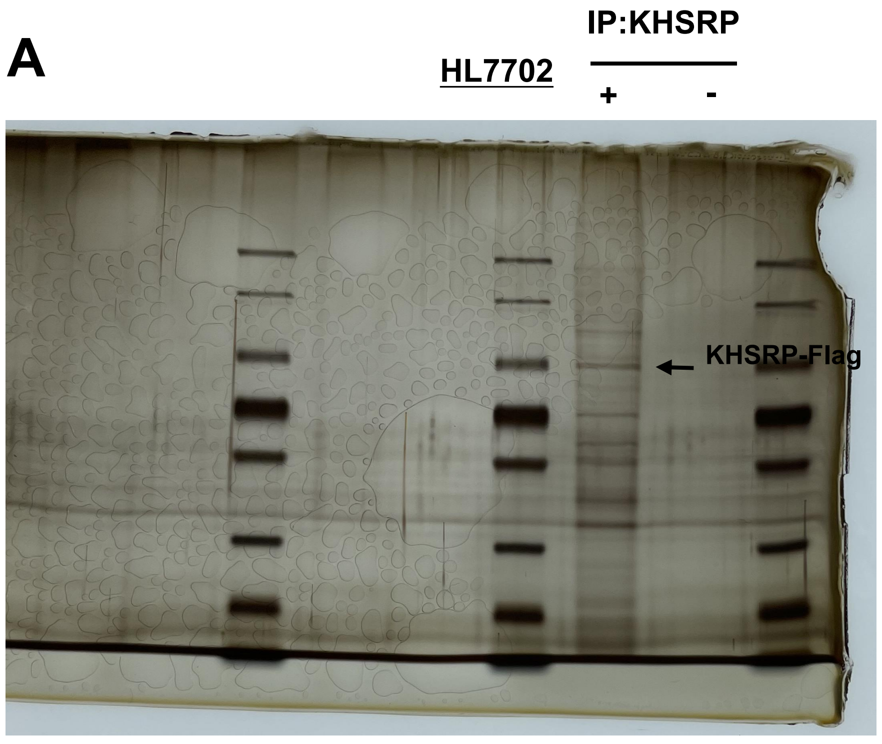

KHSRP (A302-021A, BETHYL)

IgG (B900610, Proteintech)

D

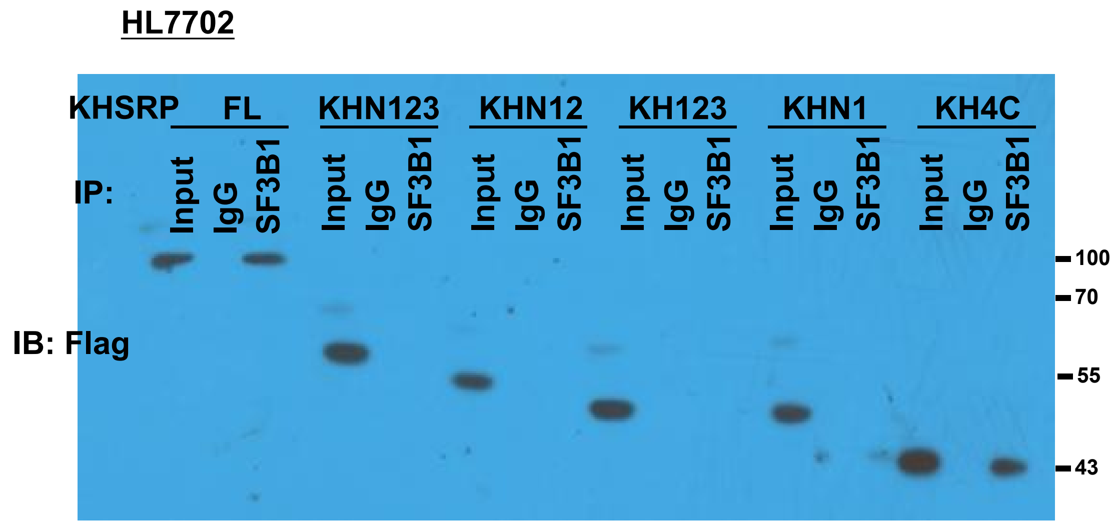

FLAG (20543-1-AP, Proteintech)

SF3B1 (sc-5a4655, Santa Cruz)

C

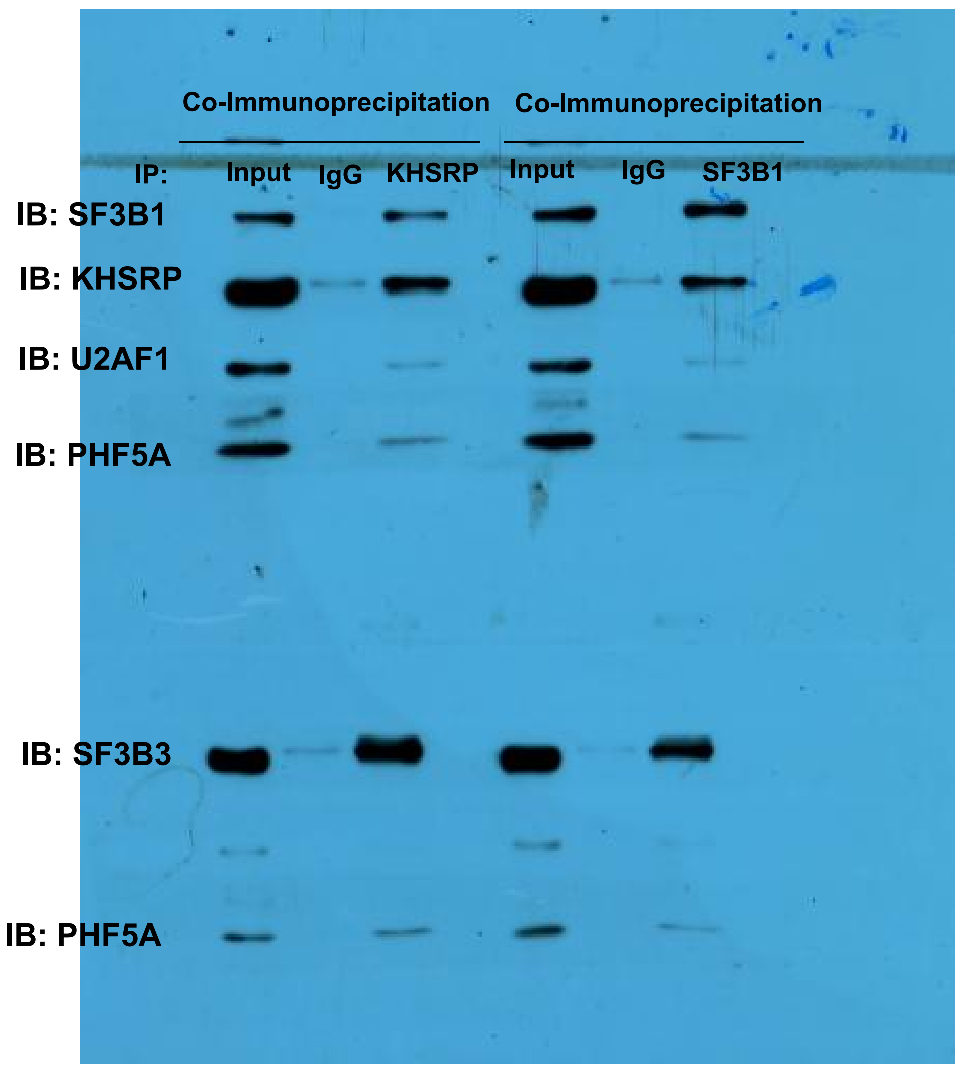

KHSRP (A302-021A, BETHYL)

SF3B1 (sc-5a4655, Santa Cruz)

U2AF1 (10334-1-AP, Proteintech)

SF3B3 (14577-1-AP, Proteintech)

PHF5A (15554-1-AP, Proteintech)

E

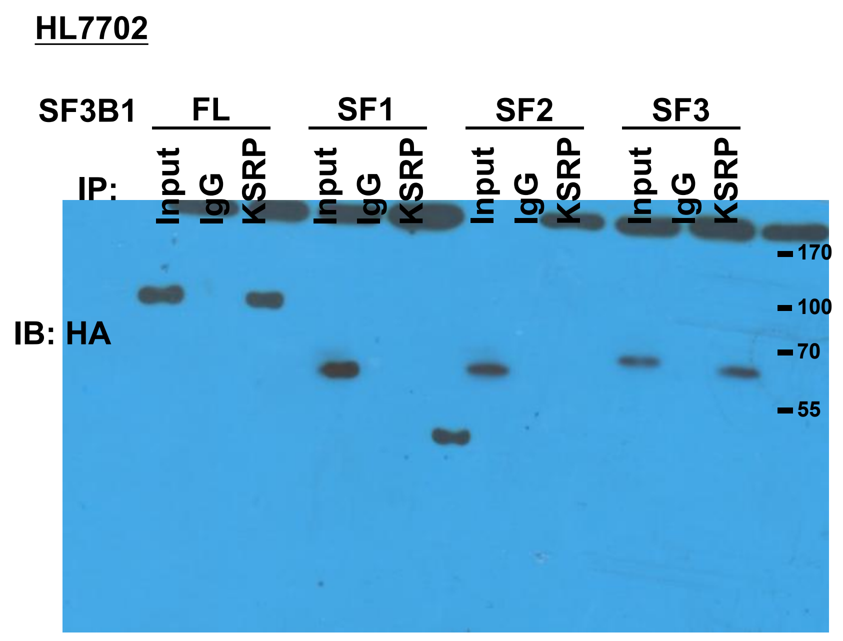

KHSRP (A302-021A, BETHYL)

HA (51064-2-AP, Proteintech)

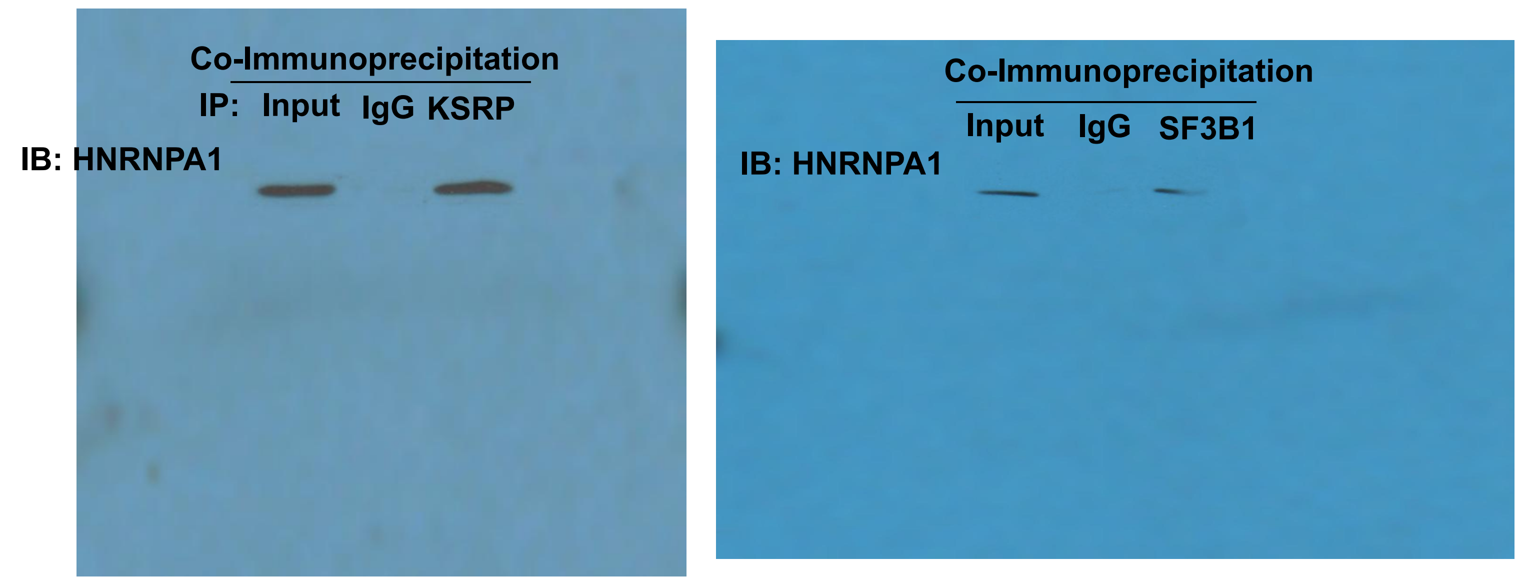

F

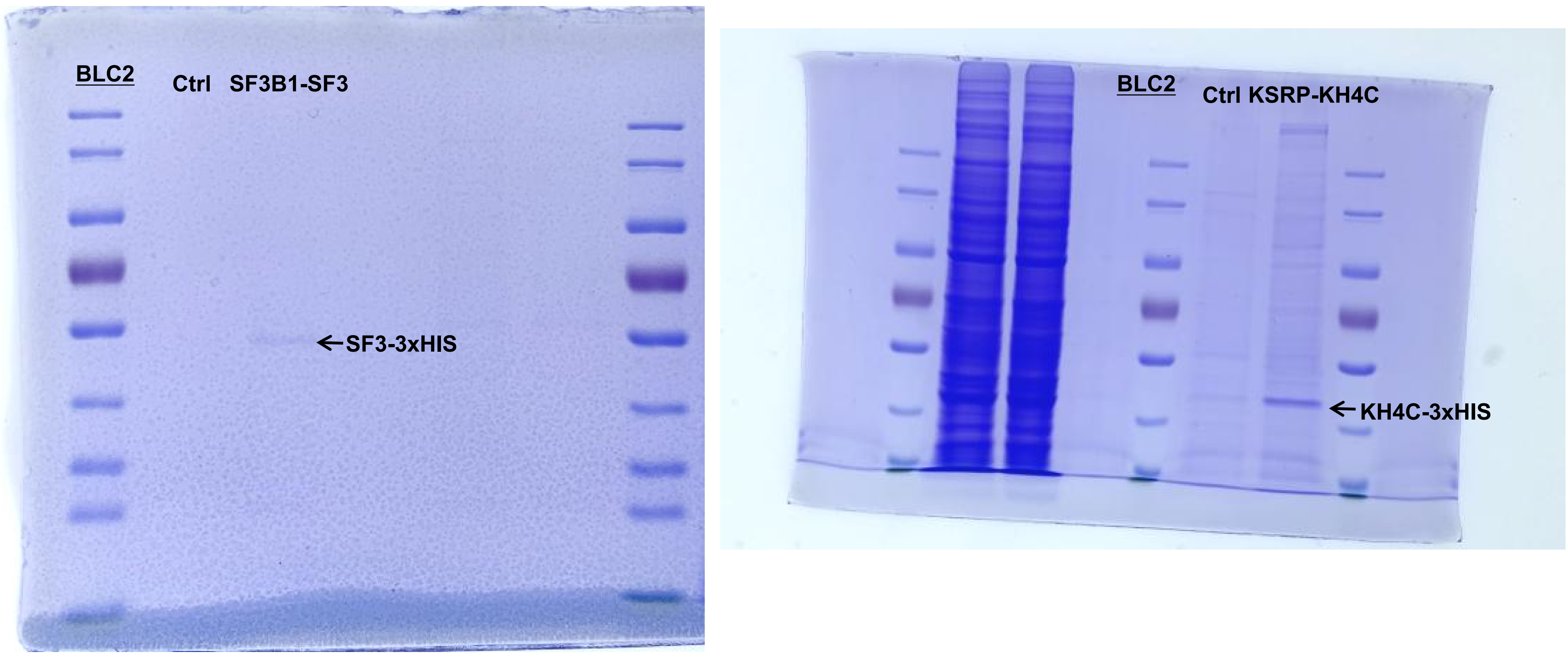

Figure 5

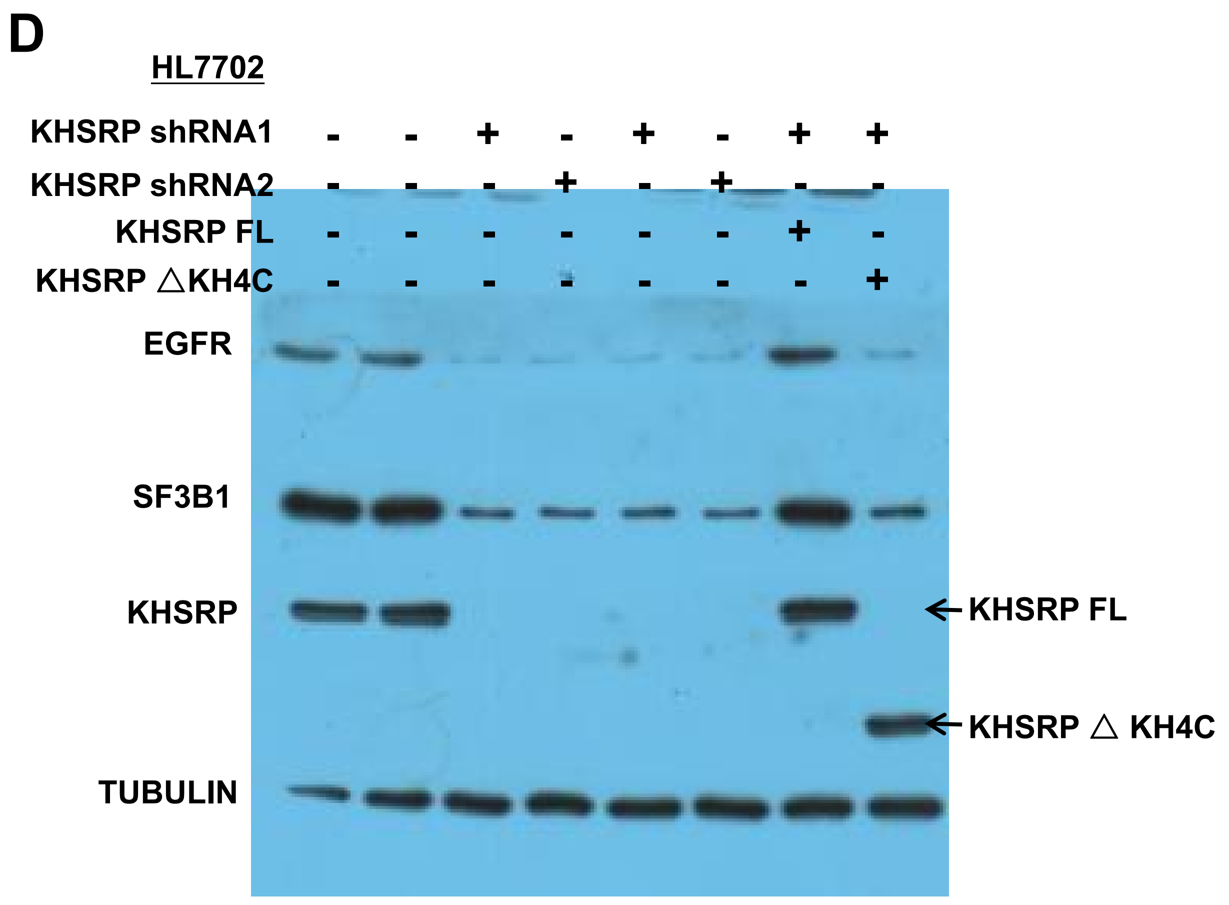

Antibody messages

KHSRP (A302-021A, BETHYL)

SF3B1 (sc-5a4655, Santa Cruz)

EGFR (510673, ZENBIO)

αTUBULIN (66031-1-Ig, Proteintech)

Figure 6

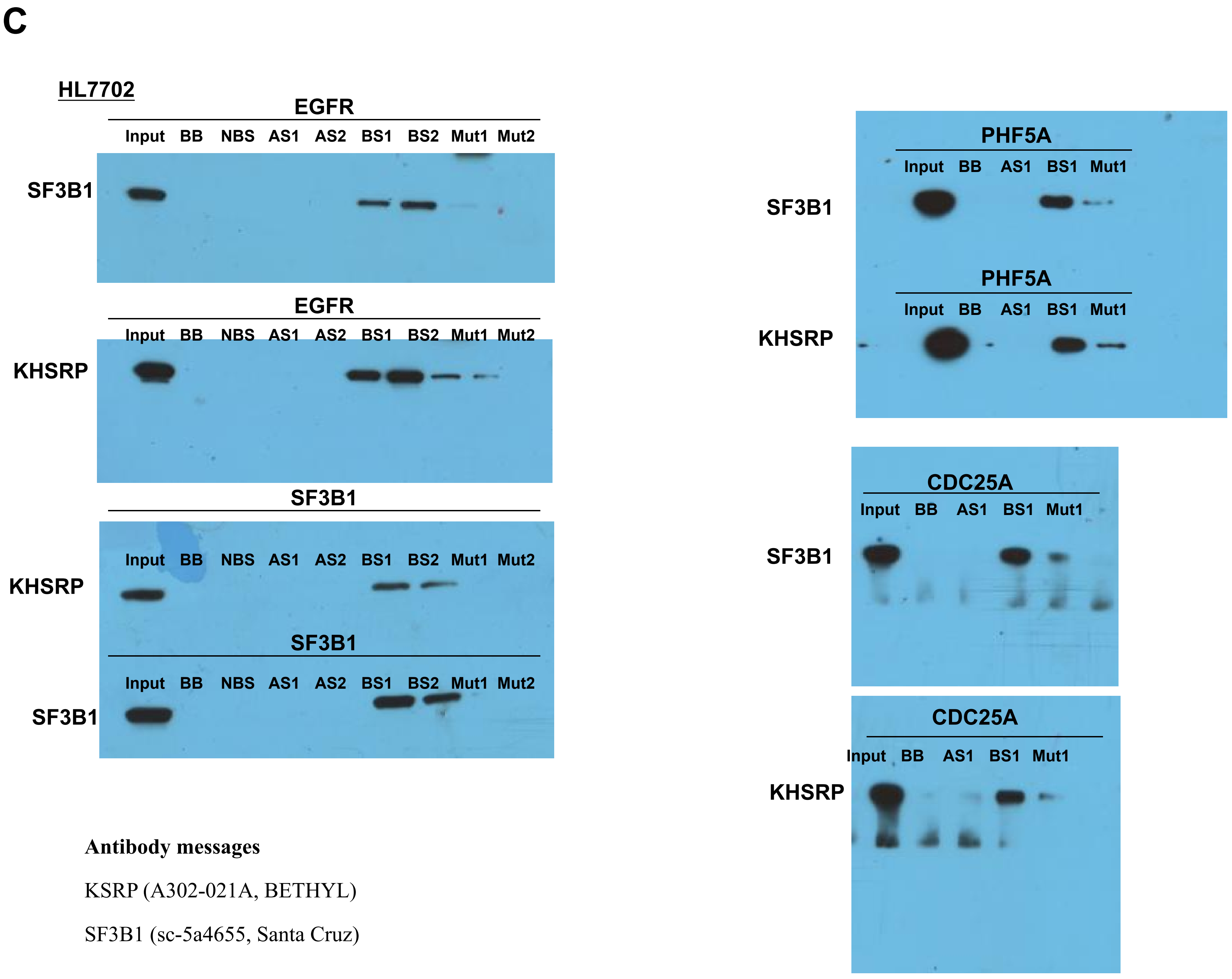

Antibody messages

KSRP (A302-021A, BETHYL)

SF3B1 (sc-5a4655, Santa Cruz)

Figure 7

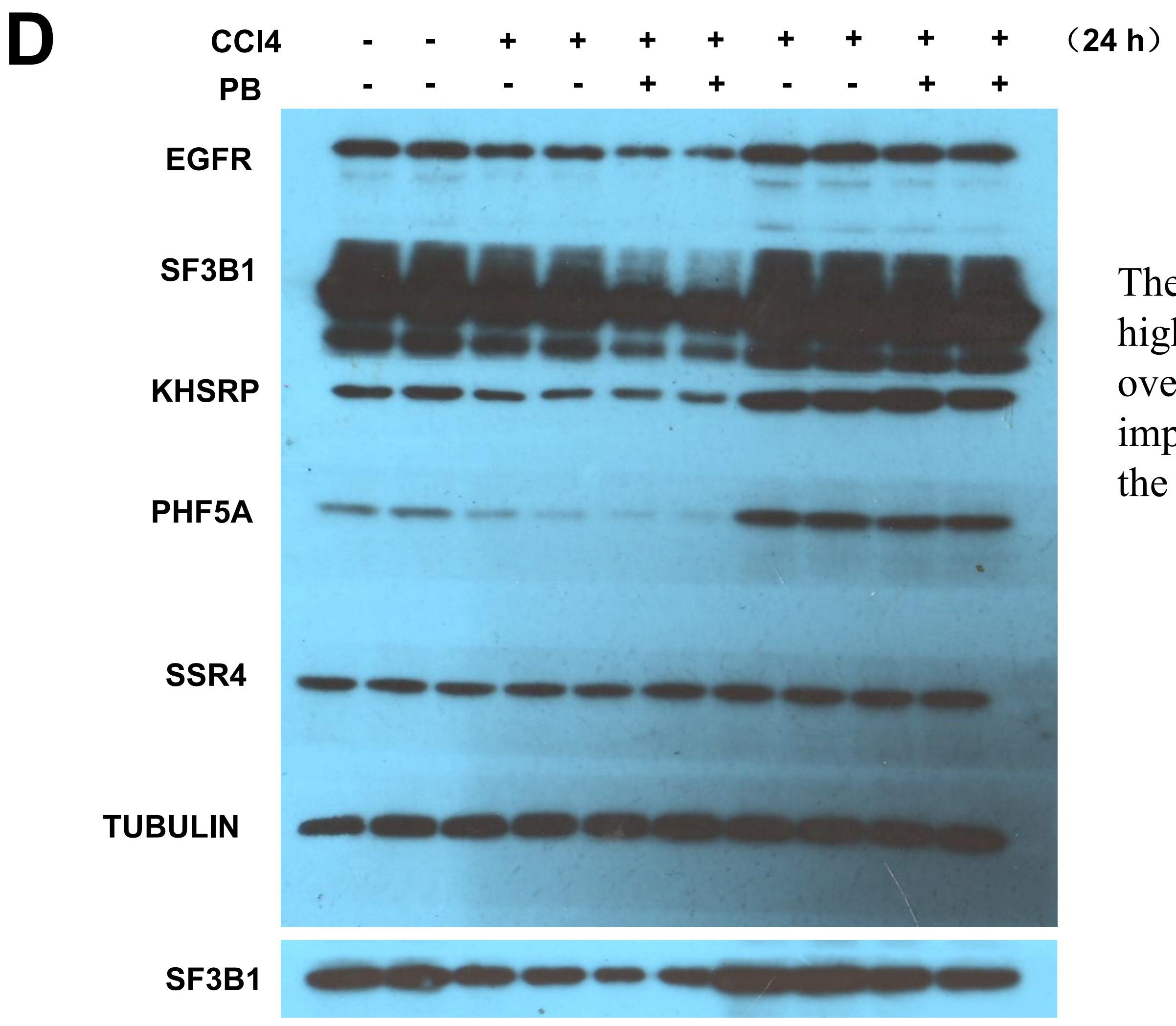

The expression level of SF3B1 is significantly higher than that of other proteins, resulting in overbright bands for the same exposure time. To improve the clarity of the banding, we shortened the exposure time on the other film.

Antibody messages

KSRP (A302-021A, BETHYL)

SF3B1 (sc-5a4655, Santa Cruz)

EGFR (510673, ZENBIO)

αTUBULIN (66031-1-Ig, Proteintech)

PHF5A (15554-1-AP, Proteintech)
