# Supplementary material for: KHSRP ameliorates acute liver failure by regulating pre-mRNA splicing through its interaction with SF3B1
Source: Cell Death Dis. 2024 Aug 26;15(8):618. doi: 10.1038/s41419-024-06886-1 (PMC11347664; doi:10.1038/s41419-024-06886-1)
Supplement: Supplementary file 4 — Supplementary tables [file 41419_2024_6886_MOESM4_ESM.pdf]

Supplementary Table 3: Khsrp-regulated genes with RIs in primary hepatocytes isolated from mice injected with AAV-shKhsrp as determined by RNA-Seq analysis

SYMBOL	Ctrl1-1	Ctrl1-2	Ctrl2-1	Ctrl2-2	Ctrl3-1	Ctrl3-2	shKhsrp-1	shKhsrp-2	baseMean	log2Fold-Change	lfcSE	stat	pvalue	padj	type
Klf6	500.4091715	504.2589839	578.1267519	485.7197818	547.5710022	474.651122	146249.2537	140882.38	36277.79632	8.136004075	0.06666917	122.0354794	0	0	Up
Tbrg4	3462.054132	3456.414132	3509.983462	3582.847668	3479.725401	3417.877137	18488.84374	17143.49485	7067.655065	2.369043691	0.05972661	39.66479376	0	0	Up
Zfp385a	488.749152	493.7734474	442.3926449	566.495938	563.1565146	486.3228709	24294.68275	22205.51173	6192.63563	5.470802698	0.09217546	59.35205337	0	0	Up
Dnajc5	4444.410778	4547.863161	4589.823691	4610.617972	4584.218713	4589.915256	45479.69873	48786.01404	15204.07029	3.361106628	0.05027398	66.85578922	0	0	Up
Chtop	6538.355952	6229.361928	6458.932615	6470.595255	6543.837139	6673.322435	39125.03972	37148.55696	14398.50025	2.528693337	0.0492979	51.2941428	0	0	Up
Efnb2	5069.193491	5436.274074	5445.451284	5218.564833	5388.431153	5446.816154	109474.5545	107648.7115	31140.99962	4.324663666	0.04558197	94.87663515	0	0	Up
Kpnb1	2892.656512	3022.694212	3009.275423	3101.379263	2863.578144	2945.171306	24654.61093	24736.52017	8403.235745	3.087809492	0.05484659	56.2990296	0	0	Up
Ifrd1	3236.627088	3213.34033	3220.417367	3224.669186	3015.277132	3423.713011	65914.786	67333.93715	19072.84591	4.370555839	0.05482644	79.71620153	0	0	Up
Ubl3	5354.863969	5214.171346	5390.152203	5204.747859	5144.258125	4964.383866	85547.318	83011.35899	24978.9068	4.059743171	0.04465123	90.92120385	0	0	Up
Akt1	3149.176941	3155.193264	2984.139477	3125.824679	3060.994635	2841.098212	23796.6098	21834.74981	7993.473352	2.950980194	0.0636859	46.33647332	0	0	Up
Rac1	4664.007812	4700.380056	4830.123332	5004.933156	4808.650092	4703.714808	30328.8835	29027.53094	11008.52796	2.641589705	0.05093661	51.86033792	0	0	Up
Uba1	6170.093668	6277.976688	6080.887992	6059.274564	6237.322061	6262.865932	28916.4238	28524.5455	11816.17378	2.200107844	0.04638051	47.43604736	0	0	Up
Supt6	1990.948335	2311.584189	2192.859906	2170.327778	2213.14276	2385.900005	54845.34967	57042.83719	15644.11873	4.604215943	0.06281074	73.30300393	0	0	Up
Sdf2	3390.150678	3247.656632	3319.955712	3333.07929	3259.450159	3241.828258	16784.11859	15798.92454	6546.895482	2.325308826	0.06003539	38.73230307	0	0	Up
Bbc3	559.6809375	734.9407875	679.6759726	721.6711856	704.4651604	628.3291492	32909.46523	30694.61945	8454.105984	5.577495457	0.08838663	63.10338176	0	0	Up
Snrpd1	6055.436809	5921.468446	5777.245767	5815.883251	5675.204581	5800.859204	61459.38252	60545.86722	19631.41847	3.410144316	0.04307287	79.17150827	0	0	Up
Puf60	1447.785758	1312.598527	1320.13987	1478.416229	1348.666339	1415.199554	21648.3178	19998.80829	6246.241546	3.913158572	0.07527885	51.98217702	0	0	Up
Axl	12000.10343	12233.76144	12168.81405	12369.38035	12613.8747	12288.4063	131119.1132	125273.7502	41258.40046	3.364058806	0.0407136	82.62738864	0	0	Up
Fam32a	712.2328596	651.0564953	694.7575401	644.0835618	644.2011791	553.4354271	34983.51616	31303.91975	8773.400372	5.791973588	0.08103676	71.47340859	0	0	Up
Tbc1d8	1082.43848	1324.037294	1220.601525	1236.08776	1222.943206	1317.93498	31341.00532	31569.26021	8789.288596	4.629433392	0.07427123	62.33144692	0	0	Up
Snrnp200	1179.605309	1406.015125	1365.384572	1207.390967	1250.997128	1289.728254	21360.75115	21877.63312	6367.188204	4.088839183	0.07961639	51.35674926	0	0	Up
Man1a	2297.023847	2291.566346	2223.023041	2550.825987	2285.875152	2387.845296	35387.61308	35981.77368	10675.6933	3.932449677	0.06217021	63.2529628	0	0	Up
Calr	1246.650421	1316.411449	1242.721157	1265.847396	1311.26111	1329.606729	16648.79311	15896.30538	5032.199594	3.623233442	0.06813532	53.17702188	0	0	Up
Ruvbl2	4442.467441	4232.343835	4354.551239	4510.710621	4165.487947	4389.550233	26035.11878	24272.84442	9550.384315	2.555741209	0.05833423	43.8120305	0	0	Up
Bax	4628.056085	4368.655809	4240.936764	4418.243179	4030.413506	4097.75651	56943.83438	53246.77123	16996.83343	3.760842191	0.05250244	71.63175781	0	0	Up
Map3k20	8252.378823	8745.890694	8883.043222	8825.857917	8695.676884	8955.149345	56134.70077	60515.49155	21126.02365	2.724336231	0.0501666	54.30578189	0	0	Up
Utp20	1728.597895	1974.140559	1858.049109	1816.400672	1847.402736	1848.02691	51080.66999	52532.04947	14335.66717	4.809319349	0.05899894	81.51535094	0	0	Up
Ralb	892.9631624	964.6693605	878.7526628	903.4175372	867.5935235	945.4116611	23306.99469	23503.62513	6532.928466	4.689576755	0.06873945	68.2224953	0	0	Up
Tax1bp1	1657.66611	1691.984303	1903.293811	1658.036892	1843.2466	1921.947986	23508.10339	23147.15765	7166.429593	3.631017643	0.06718302	54.04665785	0	0	Up
Cd44	11392.81075	11879.15965	11642.97006	11533.98484	11526.00593	11586.15608	77635.47621	76971.96011	28021.06545	2.741869963	0.04038806	67.8881348	0	0	Up
Slc2a9	1509.000861	1673.872922	1490.058863	1664.413957	1545.043796	1644.743949	24500.49025	22970.26401	7124.736076	3.895154873	0.07262875	53.63103073	0	0	Up
Ripk4	873.5297965	848.3752281	871.714598	951.2455245	902.9206849	909.4237686	94228.82346	87136.19696	23340.27875	6.644801326	0.06055601	109.7298346	0	0	Up
Eif4g2	14074.61524	13710.31562	14176.67339	13792.52868	13794.21751	14138.3785	102042.9302	100053.006	35722.83314	2.854960737	0.03971649	71.88351618	0	0	Up
Ampd3	4409.430719	4483.043481	4617.97595	4677.577155	4611.233601	4638.547543	19288.57974	18708.73551	8179.390462	2.038362832	0.05241205	38.89111287	0	0	Up
Crkl	2667.229468	2406.907248	2603.07854	2660.298936	2638.107732	2597.936776	38018.00211	36414.18035	11250.71764	3.829441978	0.05797445	66.05395462	0	0	Up
Clint1	4727.166251	4702.286517	5024.172833	4779.610194	4754.620315	4816.541714	39166.38917	38754.89412	13340.71014	3.02519263	0.04782459	63.25600185	0	0	Up
Mob1b	1753.861271	1763.476598	1772.586893	1643.157074	1602.190674	1706.020631	14329.46474	17270.35796	5230.139481	3.255616245	0.0860811	37.82033566	0	0	Up
Atp6v1b2	3092.82018	3175.211107	3351.124285	3058.865497	3349.846131	3405.232742	26286.03478	25796.09518	8939.403737	2.94666798	0.05747419	51.2694178	0	0	Up
Crip2	617.0093668	587.1900455	606.2790111	522.9193274	559.000378	529.1192836	27261.50595	24295.17948	6872.275355	5.566788719	0.08342037	66.7317693	0	0	Up
Epha2	2667.229468	2980.752065	2835.334679	2905.815937	2975.793834	3071.615253	80389.91359	74661.62201	21561.0096	4.680128967	0.05685923	82.31081089	0	0	Up
Ptbp1	7083.461865	6738.387065	6786.705348	7093.421933	6760.995278	6675.267726	44365.14303	39562.52971	15633.23899	2.643012345	0.05812865	45.46832243	0	0	Up
Gtf2h1	2901.401526	3018.881289	2947.943716	3116.259081	3042.29202	2903.347539	16502.19051	16402.86443	6354.397513	2.468598239	0.06074791	40.63676018	0	0	Up
Kif5b	1985.118325	2003.690707	2177.778338	2160.76218	2097.809969	1986.142605	31921.77717	31518.33628	9481.426947	3.957735397	0.05475195	72.28483086	0	0	Up
Ppp2r1a	3480.51583	3262.908321	3412.455993	3410.666914	3427.773693	3514.169065	22140.75218	20880.59626	7941.229783	2.631515713	0.05883941	44.72369524	0	0	Up

Bcl2l1	3103.508532	3017.928059	3109.819206	3009.974665	3276.074706	3259.335881	18279.2772	18134.27789	6898.774517	2.478143449	0.05601842	44.23801371	0	0	Up
Cct4	8705.176248	7966.148069	8053.557013	8002.153691	8563.719545	7887.184321	36988.02484	35387.66122	15194.20312	2.137475526	0.05657022	37.78446599	0	0	Up
Zfp655	1664.467788	1731.066758	1643.890851	1726.058918	1732.069944	1644.743949	21116.41348	21270.11962	6566.103914	3.65021955	0.06210805	58.77208314	0	0	Up
Rhoa	1902.52652	1810.184897	1931.44607	1765.384152	1781.943584	1895.686551	17820.67418	17544.63244	5806.559799	3.265148737	0.06753648	48.34644628	0	0	Up
Fbxw9	1586.734324	1300.206529	1523.238311	1476.29054	1536.731522	1348.086998	83820.97837	78481.80983	21384.25955	5.814812882	0.07259457	80.09983422	0	0	Up
Ubc	640.3294059	572.8915866	585.1648167	612.198237	537.1806606	507.7210772	81104.1314	75808.75044	20046.04595	7.231253986	0.06805022	106.2634874	0	0	Up
Herpud2	1624.629388	1641.463082	1775.603207	1723.93323	1593.878401	1752.707627	24340.731	23751.99094	7275.617109	3.844420875	0.06272001	61.29496439	0	0	Up
Rps18	1667.382793	1771.102442	1713.266061	1709.053412	1787.138755	1589.303142	41976.2724	39313.27049	11440.84869	4.590226191	0.06347365	72.31703983	0	0	Up
Tmem184b	3287.153839	3146.614189	3142.998654	3253.365978	3128.531855	3178.606284	23410.36832	22058.99377	8075.829111	2.849739397	0.05810444	49.04512366	0	0	Up
Ppp2cb	1401.14568	1263.030536	1216.579773	1382.760254	1342.432134	1309.181169	24227.02001	23508.09214	6956.280211	4.170259445	0.07146539	58.35355636	0	0	Up
Ccnt1	12931.93333	12538.79523	12371.91249	12783.88957	12665.82641	12649.25787	101197.1459	103181.7005	35040.05767	3.013184154	0.03985498	75.60370403	0	0	Up
Tjap1	2232.89374	2113.312226	2247.153549	2297.869077	2033.389851	2307.1157	29475.58117	28098.39265	8850.713495	3.728850345	0.06517703	57.21111319	0	0	Up
Rps5	847.2947525	792.1346231	832.5025227	745.0537572	747.0655609	751.8551585	36155.39723	33088.93735	9245.03012	5.52961212	0.0721082	76.68492385	0	0	Up
Pkd2l2	2564.232628	2707.174885	2721.720204	2549.763143	2766.947968	2479.273996	20220.82193	19500.28986	6938.778077	2.920966431	0.06736385	43.3610392	0	0	Up
Psmb1	4561.982641	4484.949942	4871.346283	4680.765687	4803.454921	4574.352924	24445.98415	23877.96065	9537.59965	2.365576115	0.05302608	44.61155968	0	0	Up
Ddx19a	800.6546744	785.4620089	774.1871286	730.1739389	805.2514739	769.3627818	25101.93683	24985.77938	6844.101027	4.991885543	0.06684929	74.67372436	0	0	Up
Ube2i	11413.21578	10598.97097	10699.86938	11062.08203	10841.28242	11139.71168	48610.97998	45810.0913	20022.02544	2.102788313	0.05076585	41.42131965	0	0	Up
Actn1	8072.620188	8761.142384	8380.324307	8937.456554	8579.305058	8996.000466	43482.70813	44598.63792	17476.02438	2.325203011	0.05345932	43.4948084	0	0	Up
Nolc1	3282.295498	3193.322488	3080.661509	2986.592094	3118.141514	2902.374894	66521.87114	61368.69065	18306.74372	4.40921404	0.05517774	79.90928431	0	0	Up
Golga7	2268.845467	2173.365753	2155.658706	2388.210831	2118.590652	2291.553368	16110.31047	16125.90974	5704.055624	2.869445812	0.06930581	41.4026747	0	0	Up
Atp6v0e	2566.175965	2368.778025	2428.132358	2606.093884	2321.202313	2308.088345	19452.09803	18680.14664	6591.339444	3.042155451	0.06476342	46.97336033	0	0	Up
Prpf3	1768.436295	1723.440913	1951.554827	1782.389659	1724.796705	1844.136327	14332.28402	15095.81701	5027.856969	3.043323813	0.07133875	42.66017826	0	0	Up
Tab2	2471.92414	2721.473344	2702.616885	2694.309949	2712.918191	2643.651126	19007.59141	18773.06047	6715.94319	2.818374596	0.06208793	45.39327788	0	0	Up
Ppil4	4115.015226	4077.920478	4266.07271	4315.147295	4320.304037	4408.030502	21665.23348	20466.05764	8454.222672	2.271007541	0.05453321	41.64448522	0	0	Up
Celsr1	322.5938737	324.0984017	292.5824083	396.4408722	299.241838	360.8515702	25242.90087	23898.5089	6392.152341	6.215879356	0.0972653	63.90644078	0	0	Up
Eif3d	715.1478645	828.3573856	653.5345891	840.7097317	694.0748188	757.6910329	26199.57683	23732.33609	6802.678543	5.103481671	0.09453801	53.9833857	0	0	Up
Nup50	816.2013671	774.9764724	788.2632582	783.316147	859.2812502	865.6547102	60777.11656	56581.84165	15280.83143	6.08813952	0.06160519	98.82510891	0	0	Up
Rnd3	1655.722773	1749.178139	1957.587454	1799.395165	1747.655457	1737.145295	91998.77232	88513.82315	23894.90997	5.694923857	0.05277014	107.9194453	0	0	Up
Traf4	1216.528704	1349.77452	1204.51452	1411.457046	1387.110603	1371.430496	33297.58622	29982.5779	8902.622501	4.519815795	0.07660584	59.00092648	0	0	Up
Rpl19	5307.252223	4667.970216	4802.976511	5061.263897	5059.057324	4653.137229	48398.59415	43212.0777	15145.29116	3.237801765	0.06875832	47.08960344	0	0	Up
Wsb1	10262.76052	10510.32052	10603.34735	10735.78887	10192.92511	10292.53724	65454.30347	63396.71365	23931.08709	2.652994847	0.04149017	63.94273119	0	0	Up
Med13l	1424.465719	1483.226803	1458.89029	1409.331358	1466.0772	1520.245295	20868.31677	23424.11233	6631.83322	3.890447343	0.07103885	54.76506706	0	0	Up
Ywhab	717.0912011	816.9186185	859.6493441	717.419809	720.0506728	669.1802704	21820.29393	20882.38307	5900.373364	4.94256417	0.08471336	58.34456408	0	0	Up
Il12rb2	683.0828108	760.6780135	820.4372687	792.8817444	758.4949367	703.2228714	62688.58896	62578.35723	16223.21798	6.422185439	0.06166384	104.1483238	0	0	Up
Vezf1	3285.210503	3272.440627	3207.346675	3255.491666	3301.011526	3354.655164	16381.90119	15881.11754	6492.396862	2.277134408	0.05678226	40.10292312	0	0	Up
Kansl1	7691.726217	8519.975044	7783.094237	7776.830729	7548.583171	7791.865038	80055.35893	86065.00772	26654.05514	3.43675871	0.05360022	64.11837042	0	0	Up
Kdm6b	1998.721681	2090.434691	2037.017042	1965.198855	2112.356447	2111.613906	22520.41534	20318.64628	6894.30053	3.342215274	0.06677384	50.05276526	0	0	Up
G3bp1	3442.620766	3363.950764	3345.091658	3321.388005	3201.264246	3272.952921	25454.34693	25625.45536	8878.383831	2.979865223	0.05204988	57.25018557	0	0	Up
Rars	1509.000861	1444.144349	1386.498767	1368.94328	1503.482429	1344.196415	41261.11483	39979.74853	11224.64118	4.835232816	0.06056394	79.83682922	0	0	Up
Rab5c	1374.910636	1445.09758	1389.51508	1275.412994	1329.963725	1397.691931	16685.44376	16453.78835	5168.978007	3.602472412	0.06952858	51.8128306	0	0	Up
H13	6061.266819	6184.56009	6281.975558	6458.903969	6153.160294	6308.580282	26039.81758	24919.66762	11050.99153	2.031745138	0.05030749	40.38653148	0	0	Up
Atp6v1e1	5273.243833	5149.351665	5169.961318	5340.791911	5451.812237	5182.256513	37972.89361	36484.75912	13253.13378	2.807885824	0.05042051	55.68935924	0	0	Up
Tnfaip3	1684.872822	1897.882111	2208.946911	1936.502062	1564.785445	1556.233187	1702056.228	1625086.098	417248.9435	10.05809507	0.05707639	176.2216355	0	0	Up
Perp	306.0755127	328.8645547	262.4192734	285.9050794	328.3347945	287.9031396	68223.77701	65949.16374	16996.55539	7.768698664	0.07614104	102.0303664	0	0	Up
Serinc1	5921.346585	5737.494942	5891.86568	5957.241525	6022.24199	5976.908084	30811.92028	30215.75587	12066.84687	2.346547169	0.04728508	49.62553118	0	0	Up
Zwint	9228.905458	9011.84203	9027.826269	9135.145568	9535.216485	9322.809436	94045.57021	94573.77029	30485.13572	3.322300212	0.03948016	84.15114448	0	0	Up
Arid5b	6035.031775	6515.331106	6438.823859	6261.214955	6536.563899	6341.650237	25890.3957	26872.64483	11361.45705	2.034689984	0.05393914	37.72195941	0	0	Up
Sgk1	859.9264404	822.6380021	913.9429868	918.2973555	949.6772221	915.2596431	27518.0605	25791.62817	7336.17879	4.837528273	0.06697475	72.22913152	0	0	Up
Apaf1	3911.936552	3989.270033	4068.001457	4051.561943	3952.485944	4024.80808	59542.27155	62435.41288	18246.96855	3.934495181	0.0468832	83.92121108	0	0	Up
Hsp90b1	8714.892931	8848.839598	9072.065534	8748.270293	8288.375493	8951.258762	37828.17053	35708.39261	15770.03322	2.092555806	0.05244244	39.90195273	0	0	Up

Ddx21	556.7659326	565.2657419	593.2083193	696.1629257	601.6007785	621.520629	21014.91937	20326.6869	5622.016324	5.078600769	0.07812572	65.00548753	0	0	Up
Ddx50	5060.448476	5487.748526	5445.451284	5193.056573	5486.100364	5504.202253	28972.80942	29174.9423	11290.5949	2.403485559	0.05384593	44.63634989	0	0	Up
Eif4ebp2	685.0261474	737.8004793	732.9641776	861.9666149	696.1528871	813.1318402	27513.3617	26435.77115	7309.521875	5.158348224	0.08053156	64.0537452	0	0	Up
Cand1	1777.18131	1905.507956	1879.163303	1930.124997	1886.886034	2007.540811	26155.4081	25872.92777	7926.842535	3.739427285	0.06034183	61.97072823	0	0	Up
Tbk1	5178.992008	5164.603355	5270.505101	5286.586859	5433.109622	5408.88297	22873.7652	22704.92356	9665.171085	2.071740742	0.04855244	42.67016358	0	0	Up
Egfr	7428.404109	8096.74066	7902.741338	7715.185768	7763.663242	7892.047549	68013.27071	70019.50417	23103.94469	3.140214246	0.04847839	64.77555357	0	0	Up
Tbc1d15	1442.927417	1586.175707	1585.575457	1545.375411	1618.815221	1502.737671	28951.19493	30354.23321	8573.379378	4.248340255	0.06442322	65.94423518	0	0	Up
Rab21	1678.071144	1807.325205	1692.151867	1698.42497	1787.138755	1937.510318	34100.1415	33499.00896	9774.97159	4.181291343	0.05942475	70.36279179	0	0	Up
Rab1a	9167.690356	9168.171847	9198.7507	9005.47858	9234.935613	9202.201364	80068.51557	84717.7572	27470.43765	3.159932719	0.04325343	73.05622726	0	0	Up
Mdm2	10712.64294	10716.21833	10985.41372	11014.25405	10627.24139	10549.31572	754484.6609	733522.5174	194076.5331	6.134794638	0.03500236	175.2680539	0	0	Up
Llph	141.863571	198.2719634	175.9516201	190.2491049	131.9573383	140.0609868	49506.57152	44335.08427	11852.5013	8.42925819	0.10988684	76.70852898	0	0	Up
Tmbim4	4794.211364	4722.30436	4374.659995	4653.131739	4593.570021	4182.37669	36393.15658	34163.7002	12234.63887	3.007429616	0.05891243	51.04915382	0	0	Up
Hmg20b	1445.842422	1354.540673	1389.51508	1411.457046	1492.053054	1279.029151	28480.37503	26803.85286	7957.083165	4.319276155	0.07158209	60.34017752	0	0	Up
Nfyb	1641.147749	1653.85508	1682.097488	1705.864879	1602.190674	1679.759196	45743.77136	43539.95631	12406.08034	4.76536559	0.05527754	86.20798812	0	0	Up
Txnrd1	6807.508069	6811.78582	6991.814665	6910.612738	6702.809365	6719.036785	40901.18664	40019.95163	15233.08821	2.591914433	0.04401724	58.8840703	0	0	Up
Fbxw11	2629.334404	2604.225981	2597.045913	2642.230585	2640.1858	2672.830499	18177.78309	18601.52725	6570.645439	2.79124236	0.05711817	48.86785279	0	0	Up
Cpeb4	2728.44457	2917.838846	2712.671263	2747.452157	2814.743539	2919.882517	23887.76654	23440.19357	8021.124126	3.044735677	0.05734519	53.09487438	0	0	Up
Cdc34	1816.048042	1880.723961	1986.745151	1897.176828	1814.153643	2063.954264	96768.05574	85795.20025	24252.75723	5.556192948	0.0650838	85.36982832	0	0	Up
Sptbn1	16643.70621	17126.69407	16801.87157	17331.79974	17218.8741	17318.93008	127847.8077	122122.7206	44051.55051	2.855492005	0.04070112	70.15757222	0	0	Up
Nudcd2	1824.793056	1794.933207	1866.092611	1901.428205	1986.633314	1931.674443	70688.76822	65960.77797	18494.38763	5.124304958	0.05302412	96.64102781	0	0	Up
Canx	6720.057923	7246.458971	6805.808667	6888.29301	6834.766703	6927.182973	40404.99322	42607.24441	15554.35073	2.592621697	0.05129446	50.54389194	0	0	Up
Rack1	2691.521175	2591.833983	2601.067664	2726.195274	2495.760052	2419.942606	15919.53913	15342.39601	5848.531988	2.669061147	0.06239236	42.7786523	0	0	Up
Clk4	9503.887586	8942.256196	9116.304798	9464.627258	9045.831395	9499.830961	49991.48783	47599.57591	19145.47524	2.395552702	0.05032054	47.60586551	0	0	Up
Myo1g	22.34837077	20.97107305	33.17944837	25.50825987	24.93681984	11.6717489	69190.79034	64756.4718	16760.73473	11.85413407	0.2461027	48.16742759	0	0	Up
Trim11	4572.670992	4515.453321	4272.105337	4392.734919	4600.84326	4247.543955	32589.00697	30222.00969	11176.54606	2.82775797	0.05783362	48.89471102	0	0	Up
Laptm4a	2544.799262	2781.526871	2968.052472	2742.137937	2662.005517	2698.119288	34633.92534	34748.87864	10722.43067	3.694174771	0.05861655	63.02272813	0	0	Up
Lpin1	964.8666161	896.0367578	911.9321112	974.628096	882.1400017	840.365921	52963.00983	53653.26923	14010.78107	5.952360651	0.06020987	98.86021087	0	0	Up
Prkar1a	4157.768631	3917.777739	3939.305415	4019.676619	3877.675484	3932.406734	23129.38	21877.63312	8606.452968	2.526659932	0.05688494	44.4170301	0	0	Up
Sh3yl1	1352.562266	1362.166518	1379.460702	1379.571722	1323.72952	1440.488344	21091.97971	22327.01443	6457.121651	3.97281107	0.06623917	59.97676666	0	0	Up
Pafah1b1	5014.780066	5165.556585	5210.178831	5097.400598	5142.180057	5257.150235	30031.91925	30831.30999	11468.80945	2.549011414	0.04904618	51.97165668	0	0	Up
Sap30bp	2585.609331	2916.885616	2866.503251	2814.411339	2811.626436	2843.043503	50008.40351	49417.6494	14533.01655	4.136050223	0.05608814	73.74197903	0	0	Up
Itgb4	1647.949427	1904.554726	1654.950667	1551.752476	1590.761299	1603.892828	17329.17955	16666.41808	5493.682382	3.41155878	0.07472651	45.65392725	0	0	Up
Fbf1	3430.960747	3505.982122	3479.820327	3650.869695	3599.21433	3444.138572	20164.43632	18966.92875	7530.293857	2.474121951	0.05966146	41.46935037	0	0	Up
Rhbdf2	1861.716452	1961.748561	1903.293811	2070.420426	1958.579391	2067.844847	21290.26913	19887.13301	6625.125704	3.353987762	0.0676617	49.56995937	0	0	Up
Ywhae	1891.838169	1990.345479	2146.609766	1965.198855	1796.490062	1966.68969	57951.25739	59891.89681	16200.04078	4.968171332	0.05832624	85.17901347	0	0	Up
Prpf8	5956.326643	5901.450604	6161.323018	6143.239253	6161.472568	6013.868622	52743.10593	51537.69282	17577.30993	3.098526581	0.04377446	70.78389058	0	0	Up
Lrrc59	633.5277278	615.7869633	594.2137571	637.7064969	601.6007785	743.1013468	36128.14418	34225.34495	9272.428276	5.707154062	0.07677379	74.33726879	0	0	Up
Nr1d1	727.7795524	762.5844747	699.7847292	739.7395364	728.362946	744.0739925	43090.8281	39067.58489	10820.09228	5.801874718	0.06953023	83.4439191	0	0	Up
Ctc1	3280.352161	3023.647442	3100.770266	3154.521471	3275.035672	3123.165477	36457.06028	35373.36678	11348.48994	3.489064748	0.05555264	62.8064647	0	0	Up
Ppm1a	7053.340148	6310.386528	6553.443771	6620.456282	6816.064088	6730.708534	29916.32874	29624.32361	12453.13146	2.135950861	0.0555618	38.44279237	0	0	Up
Glrx5	108.8268489	116.2941324	99.5383451	106.2844161	78.96659615	77.81165935	30473.60658	28215.42834	7409.594615	8.548504822	0.13136406	65.07491349	0	0	Up
Mpp5	3390.150678	3520.280581	3511.994338	3519.077019	3640.775696	3348.819289	17046.31171	17591.98276	6946.174009	2.309407306	0.06085304	37.95056726	0	0	Up
Atp6v1d	998.8750064	897.943219	903.8886085	912.9831347	869.6715918	889.9708538	38907.01533	37407.6436	10223.49892	5.438346726	0.06420013	84.70928465	0	0	Up
Eif2s1	4130.561919	4047.417099	4169.550678	4053.687632	4135.355956	4111.373551	31610.71652	30931.37103	10898.7543	2.922948826	0.0494794	59.07405942	0	0	Up
Gtpbp4	1369.080627	1364.072979	1216.579773	1311.549695	1302.948836	1422.98072	16564.21469	16336.75266	5111.022498	3.592731413	0.07015576	51.21078589	0	0	Up
Psmc1	2448.604101	2497.464155	2331.610326	2506.186533	2401.207943	2391.735879	20232.09906	19823.70146	6829.076182	3.063036834	0.05985858	51.17122079	0	0	Up
Nrde2	578.142635	543.3414382	543.9418657	533.547769	534.0635581	619.5753376	20009.37587	18993.73081	5294.464911	5.077753101	0.0804478	63.11860685	0	0	Up
Dglucy	7545.975973	7502.878	7572.95773	7507.931156	7465.460438	7768.52154	39465.23294	43187.06244	16002.00253	2.439699479	0.05234611	46.60707953	0	0	Up
Ccnk	2169.735301	2073.276541	1979.707086	2111.871349	2128.980993	2173.863233	17577.27627	17344.51035	5944.90264	3.020646636	0.06273583	48.14866723	0	0	Up
Yy1	2608.92937	2853.019166	2681.502691	2798.468677	2852.148769	2912.101351	34888.60038	33808.12612	10675.36207	3.574925206	0.05699346	62.7251869	0	0	Up

Hsp90aa1	3318.247225	3231.451712	3305.879583	3342.644888	3064.111737	3079.396419	80001.79259	76408.22332	21968.96843	4.670083199	0.04672292	99.95272812	0	0	Up
Gcnt2	1410.862363	1427.939429	1576.526516	1610.208905	1631.283631	1474.530945	18118.57819	18824.8778	5759.350972	3.572918226	0.06757121	52.87634189	0	0	Up
Edn1	106.8835124	113.4344406	135.734107	142.4211176	129.87927	129.3618837	74607.56859	68004.88221	17921.27064	9.103654647	0.10517172	86.55991311	0	0	Up
Nup153	3589.342679	3849.145136	3842.783384	3678.503643	3705.195814	3922.680277	47826.28014	47428.93609	14730.3584	3.642196179	0.05264597	69.18281156	0	0	Up
Nol8	959.0366064	831.2170774	937.0680569	854.5267058	928.8965389	960.0013472	21127.6906	20989.59133	5948.503533	4.478532077	0.07475022	59.91329393	0	0	Up
Cast	4831.134759	5056.888298	5184.037448	4991.116182	5056.979256	5212.408531	25263.57559	26056.96862	10206.63859	2.321102313	0.05261472	44.11507643	0	0	Up
Smn1	2223.177057	2140.002682	2329.599451	2220.281453	2245.352819	2252.647538	17722.93911	16662.84447	5974.605572	2.934406575	0.06390788	45.91619232	0	0	Up
Btf3	1105.758519	1283.048379	1203.509082	1318.989604	1264.504573	1246.931841	18434.33764	18261.14101	5514.777581	3.869099231	0.07575136	51.07630218	0	0	Up
Polk	5149.841959	5401.004542	5645.533412	5120.78317	5147.375228	5255.204943	42642.56244	44297.56138	14832.48338	3.06302929	0.05490016	55.79272418	0	0	Up
Plk2	651.0177571	669.1678765	702.8010427	714.2312765	622.3814617	700.3049341	198687.8773	182689.1361	48179.61472	8.169996046	0.06293852	129.809147	0	0	Up
Ppwd1	984.299982	1007.564737	1013.481332	1018.204707	1065.010014	1025.168612	17691.92702	17742.07433	5193.466342	4.083770934	0.067161	60.80569239	0	0	Up
Dnajc9	199.1920003	196.3655022	179.9733714	177.494975	178.7138755	213.9820632	61637.93697	61343.67539	15515.91677	8.287298594	0.08728003	94.95068685	0	0	Up
Zswim8	3667.076142	3748.102693	3562.266229	3697.634838	3654.28314	3657.147989	24727.91223	23656.3969	8796.352521	2.726286529	0.05423241	50.27042632	0	0	Up
Mapk1ip1l	2712.897877	2572.769372	2547.779459	2537.009013	2633.951595	2695.201351	25524.82895	24844.62183	8258.632431	3.240434053	0.05594644	57.92029074	0	0	Up
Mettl6	2867.393136	2779.62041	2598.051351	2747.452157	2714.99626	2714.654266	39723.66701	38577.10708	11840.36771	3.850085148	0.05240696	73.46515095	0	0	Up
Ppp2r2a	6112.765239	6074.938572	6021.56716	5973.184187	5873.660105	5901.041716	49382.52317	51006.11851	17043.22483	3.091827472	0.04420792	69.93832448	0	0	Up
Tnfrsf10b	711.2611914	693.951872	726.9315506	764.1849521	702.387092	742.128701	36391.27706	35638.70724	9546.353707	5.639249466	0.06429475	87.70933043	0	0	Up
Ajuba	2773.141312	2858.73855	2991.177542	2919.632911	2805.392232	2845.961441	15926.11746	15061.86772	6022.753646	2.454941649	0.06119752	40.11505182	0	0	Up
Golph3	3414.442386	3304.850467	3354.140599	3380.907278	3237.630442	3316.72198	40518.70421	40953.55693	12685.11929	3.635650849	0.04874584	74.58382031	0	0	Up
Brix1	1471.105797	1451.770194	1442.803285	1423.148332	1454.647824	1474.530945	103420.6188	101096.4998	26654.39061	6.125426194	0.04857735	126.096338	0	0	Up
Retreg1	2694.43618	3019.83452	3020.335239	3000.409068	2911.373716	2824.563234	21742.29383	21772.21166	7623.18218	2.923520865	0.06236838	46.87504807	0	0	Up
Rnf19a	5602.639384	6055.87396	5852.653604	5624.571302	5845.606183	5715.266379	31056.25796	31153.82818	12113.33712	2.427966145	0.05262168	46.14003805	0	0	Up
Eif3h	8210.597086	7909.907464	8485.895279	8478.307876	7922.635469	8157.579837	42365.33316	42683.1836	16776.67997	2.402915745	0.04446322	54.04278012	0	0	Up
Rad21	2237.752082	2348.760182	2587.996973	2057.666297	2268.211571	2310.033637	48039.60573	47554.9058	13675.61653	4.383991878	0.07369158	59.49108252	0	0	Up
Matn2	2961.644961	2940.716381	2864.492376	2971.712275	3195.030041	2980.186553	18317.80737	17707.23164	6742.3527	2.544704927	0.06033702	42.17485297	0	0	Up
Washc5	2929.579907	3058.916974	3084.68326	3192.783861	3162.819983	3067.72467	18221.01206	18112.83624	6853.794619	2.544015587	0.05766097	44.12023905	0	0	Up
Gtse1	1369.080627	1383.137591	1374.433513	1517.741463	1333.080827	1479.394173	207061.1414	195559.4882	51384.68722	7.160477029	0.05580839	128.3046665	0	0	Up
Tef	2999.540024	2779.62041	2926.829521	3035.482925	2995.535483	2776.903593	36763.42213	34812.3102	11136.20554	3.632537307	0.05963019	60.91775251	0	0	Up
St13	6838.601455	7061.532236	6995.836416	6912.738426	6503.314806	6859.097772	27779.31386	30242.55794	12399.12411	2.118326433	0.05537247	38.25594998	0	0	Up
Csnk1e	4284.085509	4131.301392	4161.507176	4273.696373	4204.971245	4104.565031	29297.02671	26862.81741	10164.99636	2.756746489	0.05752566	47.92203364	0	0	Up
Slc38a2	5861.103151	5926.234599	5906.947247	6058.21172	6008.734546	6073.200012	53279.70905	50059.11218	17396.65656	3.096403174	0.04780941	64.76556058	0	0	Up
Glyr1	3461.082464	3406.846141	3518.026965	3702.949058	3757.147522	3584.199559	40696.3189	40339.78962	12808.29503	3.464671536	0.05061038	68.45772463	0	0	Up
Zbtb11	1086.325153	1119.092717	1111.008801	1087.289577	1124.234961	1153.55785	74284.29105	74886.75937	19481.56993	6.032881385	0.05245425	115.012246	0	0	Up
Usp7	4341.413938	4511.640398	4306.290223	4609.555128	4488.62757	4417.756959	21176.55814	20809.12409	8582.620805	2.237029813	0.05566582	40.18677374	0	0	Up
Klhl22	2943.183263	3240.030787	3029.38418	3188.532484	3346.729029	3141.645746	24026.85107	22855.90853	8221.533136	2.853330816	0.06400596	44.57914363	0	0	Up
Senp2	2750.792941	2548.938607	2746.85615	2939.826951	2709.801089	2516.234534	15792.6715	16655.69725	6082.602377	2.634661973	0.06874593	38.3246269	0	0	Up
Tra2b	2221.233721	2343.994029	2391.936596	2174.579154	2264.055434	2131.06682	15282.38166	16618.17436	5678.427722	2.85988561	0.07256715	39.41019603	0	0	Up
Cxadr	675.3094645	759.7247829	726.9315506	812.0129393	781.3536882	812.1591944	28389.21828	28522.7587	7684.933575	5.158066163	0.0706917	72.96565156	0	0	Up
Ets2	2945.1266	2999.816677	2929.845835	3037.608613	2918.646955	2848.879378	28359.14595	27045.96486	9135.629359	3.264096705	0.05511534	59.22301467	0	0	Up
Dyrk1a	2795.489682	2998.863447	3239.520686	2988.717782	2978.910936	3050.217046	18224.7711	19834.42228	7013.864121	2.658179254	0.06729994	39.49749695	0	0	Up
Arl13b	1812.161369	1876.911038	1855.032795	1773.886905	1881.690863	1773.133187	21623.88403	21184.35301	6722.63165	3.550377215	0.06179691	57.45234558	0	0	Up
Kansl2	13204.00045	12861.9404	12827.37583	13316.3745	13008.70768	13038.31617	78875.02002	76769.15781	29237.61161	2.579048783	0.04115463	62.66727616	0	0	Up
Cdkn1a	3158.893624	3033.179748	3043.460309	3114.133393	2948.778946	3152.344849	335826.1552	301157.8416	81929.34846	6.705696161	0.05375056	124.7558291	0	0	Up
5-Mar	8570.114355	8642.94179	9007.717513	8600.534954	8584.500228	8527.185219	43615.21433	43822.2714	17421.30997	2.35329122	0.04404525	53.42894225	0	0	Up
Rab12	2412.652374	2657.606894	2576.937157	2425.410376	2468.745164	2379.091485	22453.69236	21727.54155	7387.70967	3.188175743	0.06413803	49.70803785	0	0	Up
Chd1	3166.666971	3394.454143	3228.46087	3266.120108	3393.485566	3400.369514	42683.91189	43137.03192	13208.81262	3.659020717	0.05052085	72.42595209	0	0	Up
Tnfrsf12a	1984.146657	2029.427933	2135.549949	2024.718128	2012.609168	1872.343053	15982.50307	14624.10064	5333.174825	2.978163522	0.07132209	41.7565368	0	0	Up
Lrpprc	3741.894601	3816.735296	3886.01721	3671.063734	3899.495202	3868.212115	19062.09751	19777.24454	7715.345026	2.3220107	0.05639373	41.17498292	0	0	Up
Atp6v0c	1390.457329	1240.153002	1293.998486	1284.978591	1207.357694	1233.314801	18524.55463	16641.40281	5352.027168	3.848660318	0.07743641	49.7009129	0	0	Up
Epc1	4012.018387	4218.045376	4164.523489	4163.16058	4214.322552	4339.9453	52950.79295	51768.19059	16228.8749	3.61358958	0.04749893	76.0772846	0	0	Up

Zfp871	2930.551575	2980.752065	2787.073663	2724.069586	2818.899676	2846.934086	20946.31687	20759.98697	7349.323061	2.879847748	0.05498586	52.37433228	0	0	Up
Brd2	3090.876844	3109.438196	3059.547315	3042.922834	2975.793840	2815.809423	27519.00026	26406.28888	9002.459699	3.219189079	0.05527284	58.24179276	0	0	Up
Hspa9	3619.464396	3811.015912	3888.028086	3819.861916	4036.647711	3827.360994	138213.3636	132941.8212	36769.69548	5.10798815	0.04605632	110.9074222	0	0	Up
Etf1	3269.66381	3577.474417	3435.581063	3300.131121	3506.740289	3626.023326	175197.6294	168258.9037	45521.51839	5.589342892	0.04869574	114.7809479	0	0	Up
Bin1	4696.072866	4802.375729	4719.525171	4573.418427	4660.068207	4400.249336	19962.38786	20457.12362	8533.902651	2.157630941	0.0548534	39.33449465	0	0	Up
Iws1	4435.665763	4372.468732	4461.127649	4386.357854	4443.949102	4621.03992	18329.08449	18461.2631	7938.869576	2.02081248	0.05319272	37.99039846	0	0	Up
Riok3	10493.04591	10753.39432	11181.4741	10660.32694	10984.66914	10827.4924	50040.35536	49005.79098	20493.31864	2.182992958	0.04396685	49.65088978	0	0	Up
Smad4	6064.181824	6276.070227	6169.366521	6006.132356	6273.688257	6290.100013	30090.18439	30616.89346	12223.32713	2.272593896	0.04762401	47.71949826	0	0	Up
Pmaip1	1948.19493	1841.641506	1963.620081	1986.455738	2133.13713	1795.504039	31308.11371	29340.22171	9039.611106	3.949120883	0.07181774	54.98809878	0	0	Up
Gna1	8003.631739	7468.561699	7688.583081	7351.693065	7793.795233	7519.52423	52837.08195	50070.7264	18591.69968	2.74856339	0.05147006	53.40120767	0	0	Up
Csnk1a1	9096.75857	9196.768765	9185.680009	9319.017608	9055.182703	9474.542171	62139.76896	63250.19569	22589.73931	2.758387429	0.0426882	64.61709773	0	0	Up
Txnl1	3875.013157	4114.143241	4075.039522	4134.463788	3965.993388	3810.826017	37643.03776	39460.68186	12634.89984	3.309732879	0.05293193	62.52809788	0	0	Up
Tle4	2400.020687	2483.165696	2367.806088	2260.669531	2458.354822	2588.210319	23341.76582	25540.58215	7930.071889	3.275746449	0.06388143	51.27854328	0	0	Up
Cndp2	1708.192861	1568.064326	1477.993609	1502.861644	1467.116234	1752.707627	27891.14533	25970.30861	7917.29878	4.063328498	0.07626004	53.28253667	0	0	Up
Ccdc86	229.3137174	206.8510388	139.7558583	206.1917673	226.5094468	223.7085206	38478.48464	36776.00824	9560.852904	7.385086027	0.17592111	41.9795337	0	0	Up
Ehd1	963.8949478	1042.834269	1088.889169	1091.540954	1065.010014	1191.491034	21856.00482	22251.96864	6318.954231	4.288025347	0.06968392	61.53536072	0	0	Up
Fas	2348.522267	2229.606358	2280.332997	2287.240635	2239.118614	2300.307179	150389.8375	145706.7519	38722.71469	6.027178676	0.04589543	131.3241575	0	0	Up
Rcl1	3753.55462	3631.808561	3913.164032	3951.654592	3887.026792	3864.321532	20713.25632	21890.14075	8200.6159	2.458503697	0.0554922	44.30359006	0	0	Up
Ankrd1	22472.74431	22067.28824	22554.98682	23006.32472	22503.40183	22365.01619	162549.3958	155616.3691	56641.94088	2.826008784	0.03833866	73.7117236	0	0	Up
Tnks2	15165.79874	15668.25126	15744.15097	15798.11562	15914.88623	16036.98299	88773.51504	92556.46812	34457.27112	2.504641292	0.04047181	61.88606586	0	0	Up
Frmd8	2360.182287	2608.038903	2686.52988	2690.058573	2533.165282	2704.927808	16859.29941	16190.2347	6079.054606	2.657237921	0.06705259	39.6291642	0	0	Up
Ppp6r3	15885.80494	16301.19638	16246.86988	16315.72072	16325.30472	16198.44218	68519.8015	71054.06392	29605.90053	2.101489459	0.04012445	52.37428578	0	0	Up
Fosl1	1201.95368	1278.282226	1180.384012	1290.292812	1239.567753	1143.831392	123474.1634	116346.8753	30894.41882	6.653586406	0.05774091	115.2317548	0	0	Up
Stip1	2002.608354	2102.826689	2115.441193	2128.876855	2273.406742	2118.422426	44948.73417	44539.67337	12778.74872	4.349285266	0.05361394	81.12228581	0	0	Up
Smc3	3822.543069	4398.205958	4062.974268	4111.081216	4314.069832	4338.972654	25143.28628	25652.25742	9480.423837	2.553413925	0.06290903	40.58898651	0	0	Up
Noc3l	1284.545485	1356.447134	1312.096367	1171.254266	1295.675597	1320.852917	40499.909	41938.08616	11272.35837	4.977432153	0.06139626	81.07061027	0	0	Up
Smndc1	1997.750013	2202.915901	2220.006727	2134.191076	2242.235717	2170.945296	34770.19058	33055.88147	10099.2646	3.942110934	0.06044738	65.2155799	0	0	Up
Csnk1d	4637.772768	5074.046449	5038.248963	4947.539572	5097.501588	4929.36862	119732.0379	114194.6694	32956.39817	4.54423386	0.04926943	92.23231539	0	0	Up
Nfkb2	2431.114072	2272.501735	2264.245992	2567.831494	2394.973738	2307.1157	25072.80426	22761.2079	7758.974361	3.346775229	0.071999	46.48363795	0	0	Up
Flnb	6772.528011	7127.305147	7156.706469	7079.604959	7043.612569	7314.295979	62368.13071	66303.84441	21395.75353	3.163690067	0.0485641	65.14463056	0	0	Up
Kdm5c	4427.892417	4260.940752	4198.708375	4306.644542	4444.988136	4422.620188	23949.79072	25431.58708	9430.396526	2.477398335	0.0546131	45.3627141	0	0	Up
Pa2g4	1117.418538	1238.246541	1079.840229	1237.150604	1169.952464	1157.448433	63747.6988	61018.47699	16470.77907	5.744449155	0.06350355	90.45870916	0	0	Up
Pidd1	668.5077864	589.0965067	711.8499832	671.71751	578.742027	667.2349789	51722.52626	44565.58204	12521.90714	6.270646319	0.08247399	76.03180967	0	0	Up
Eif4a3	2078.398481	1813.044589	1803.755466	1841.908932	1842.207565	1901.522425	21389.88372	20119.41759	6598.767346	3.470710609	0.07073989	49.06299412	0	0	Up
Pde8a	277.8971321	311.706404	309.6748514	292.2821444	365.7400243	277.2040364	19770.67676	19291.23375	5112.051887	5.928402279	0.09529586	62.21049406	0	0	Up
Faf2	3160.836961	3222.872636	3180.199854	3100.316419	3046.448157	3140.6731	14928.09204	14851.0248	6078.807996	2.266822713	0.05816348	38.9733016	0	0	Up
Smad7	6759.896323	6744.106448	6853.064245	6894.670075	6652.935725	6690.830058	29614.66569	27687.42764	12237.19953	2.102372122	0.05034527	41.75907665	0	0	Up
Mybl1	1357.420607	1270.656381	1327.177935	1322.178137	1234.372582	1424.926012	24329.45388	25467.32317	7216.688587	4.226017266	0.07075747	59.72538374	0	0	Up
Rdh10	5742.559619	6072.07888	5926.050566	5808.443342	6057.569152	5971.072209	25137.64772	24555.15952	10658.82263	2.046593627	0.05094945	40.16910193	0	0	Up
Sf3b1	6144.830292	6469.576037	6616.786355	6218.701189	6440.972757	6453.504497	37619.54375	40595.30265	14569.90219	2.600708172	0.05461926	47.61522417	0	0	Up
Nop58	6197.30038	6088.2838	6356.377957	5815.883251	6106.403757	6106.269967	37867.64046	40479.16037	14377.16499	2.68151607	0.05479996	48.93281042	0	0	Up
Ercc5	2011.353369	1983.672865	2013.891972	1883.359854	1850.519839	2036.720183	18809.30199	18740.89799	6166.214758	3.271493905	0.06600321	49.56568108	0	0	Up
Map4k4	9917.818279	10775.31863	10204.18853	10551.91683	10509.83053	10626.15473	69882.4539	78988.36888	26432.00629	2.816290177	0.05782159	48.70654878	0	0	Up
Rev1	2634.192746	2782.480102	2740.823523	2520.003507	2695.254611	2713.68162	55911.97759	56873.09077	16108.93806	4.382051808	0.0526573	83.21831576	0	0	Up
Eif5b	1599.366012	1836.875353	1816.826158	1889.736919	1778.826482	1792.586102	19008.53117	19385.93438	6138.585322	3.426300067	0.06975043	49.12228103	0	0	Up
Cln8	135.0618929	114.3876712	194.0495011	147.7353384	127.8012017	133.2524666	27890.20557	26232.07545	6871.821137	7.694932399	0.11128686	69.14502552	0	0	Up
Tnni1	107.8551807	90.55690637	127.6906043	104.1587278	98.70824518	99.20986567	31162.45087	28943.55113	7591.772692	8.246338826	0.12071197	68.31417913	0	0	Up
Nucks1	1421.550714	1470.834806	1593.618959	1517.741463	1383.993501	1406.445743	16739.94986	16742.35726	5284.561538	3.584718928	0.06529454	54.90074844	0	0	Up
Tfb2m	1438.069075	1478.46065	1452.857663	1641.031385	1510.755668	1432.707178	20209.54481	19703.98556	6108.426499	3.761608268	0.06945818	54.15644554	0	0	Up
Tagln2	3142.375263	2902.587157	2910.742516	3290.565524	3260.489194	2839.15292	25491.93734	23491.1175	8416.120926	3.005828765	0.07689773	39.08865579	0	0	Up

Ccdc181	594.660996	614.8337327	597.2300706	673.8431984	522.6341824	666.2623332	19648.50792	18732.85737	5256.353726	5.010669621	0.09086866	55.14188913	0	0	Up
Abl2	2368.927301	2543.219223	2490.469503	2566.76865	2552.906931	2737.025118	68565.84975	72791.7312	19577.11221	4.739579042	0.0547052	86.63855056	0	0	Up
Ptpn14	5967.014995	6484.827727	6248.796109	6534.365905	6276.80536	6293.990596	25987.19101	27147.81271	11367.60055	2.079599287	0.05536928	37.55872292	0	0	Up
Atf3	428.5057177	400.3568492	410.2186344	446.3945478	400.0281515	428.9367722	144934.5291	135392.4235	35355.17416	8.400426431	0.06904206	121.6711415	0	0	Up
Fam107b	2506.904199	2834.907785	2779.03016	2886.684742	2815.782573	2925.718391	24707.23751	24227.28091	8210.443284	3.091193753	0.06374811	48.4907487	0	0	Up
Stam	3715.659557	3579.380878	3838.761632	3875.129813	3864.16804	3500.552025	34038.11732	33713.42549	11265.64934	3.201943179	0.05590174	57.27805773	0	0	Up
Zbtb43	1232.075397	1190.585011	1138.155623	1205.265279	1295.675597	1146.74933	18331.90377	17264.10415	5350.56427	3.866088241	0.0745103	51.88663059	0	0	Up
Fam129b	3683.594503	4020.726643	3739.223287	3550.962343	3849.621562	3618.24216	19535.73669	19061.62938	7632.467071	2.369921904	0.06265763	37.8233582	0	0	Up
Eng	23.32003906	20.01784246	24.1305079	26.57110404	31.17102479	16.53497761	38963.40095	37991.92864	9637.134385	10.66982623	0.21746688	49.06414337	0	0	Up
Rab14	4825.304749	4986.349234	5004.064076	5029.378572	4961.388113	5350.524226	51052.47718	51247.3371	16557.10291	3.310139273	0.04904542	67.49130517	0	0	Up
Sec16a	3870.154816	4007.381415	3992.59362	4101.515619	3993.008276	4153.197318	21839.08913	21243.31756	8400.032219	2.402731377	0.05450509	44.0826963	0	0	Up
Prrg4	590.7743229	600.5352738	576.1158762	618.575302	562.1174805	551.4901356	25449.64813	24730.26635	6709.940359	5.494028807	0.0719044	76.40741299	0	0	Up
Api5	3249.258776	3228.59202	3246.558751	3296.942589	3306.206697	3101.767271	28928.64068	28216.32174	9571.786066	3.156983779	0.05401198	58.4496989	0	0	Up
Cops2	3468.85581	3448.788287	3479.820327	3357.524706	3330.104482	3372.162787	21181.25694	20965.46947	7825.497851	2.652634159	0.05387738	49.23465125	0	0	Up
Gabpb1	3959.548299	3968.29896	3811.614811	3739.08576	3876.63645	3763.166375	87116.71767	85977.4543	24526.56533	4.50205685	0.04317519	104.2741761	0	0	Up
Mapre1	1156.28527	1147.689634	1096.932672	1047.964343	1146.054678	1186.627805	21065.66642	21608.71906	6181.992486	4.19302449	0.06447664	65.03168764	0	0	Up
Rab22a	3492.175849	3546.971038	3425.526684	3402.164161	3668.829618	3503.469962	23477.0913	23193.61456	8463.730397	2.702209303	0.05339099	50.61171519	0	0	Up
Zfp64	1802.444686	1782.54121	1757.505326	1887.611231	1719.601535	1736.172649	24133.98374	23635.84865	7306.963628	3.788942999	0.06019633	62.94308918	0	0	Up
Skil	6013.655073	6180.747167	5805.398026	5809.506186	5819.630329	6107.242613	27203.24081	26111.46615	11131.3608	2.160149595	0.05110094	42.2722087	0	0	Up
Zmat3	1299.120509	1534.701255	1453.863101	1421.022644	1530.497317	1527.053815	43696.97348	41978.28926	11805.19017	4.808436018	0.06544033	73.47817835	0	0	Up
Pik3ca	2635.164414	2920.698538	2865.497814	3013.163198	2748.245353	3051.189692	22654.80106	23745.73712	7954.312149	2.999813836	0.06834555	43.89186549	0	0	Up
Sec62	7332.208948	7242.646048	7088.336697	7030.714128	7170.374737	7380.435889	34930.88959	34305.75115	14060.16965	2.250335453	0.04526082	49.71927951	0	0	Up
Kpna4	1792.728003	2119.98484	2035.006167	1892.925452	1923.25223	1920.97534	34139.61143	38002.64946	10478.39162	4.230104657	0.07438399	56.86848362	0	0	Up
Wwtr1	3125.856902	3548.877499	3341.069907	3316.073784	3228.279135	3319.639917	18232.28918	18859.72048	7121.475851	2.50192303	0.06567751	38.09406112	0	0	Up
Tsc22d2	7123.300265	7590.575215	7537.767406	7332.56187	7323.112758	7372.654723	30823.19741	29752.08013	13106.90622	2.043294243	0.05026532	40.65017585	0	0	Up
Ccnl1	6197.30038	6075.891802	6327.22026	6485.475073	6109.52086	6149.06638	260568.2725	253602.9358	68939.46038	5.390349799	0.03862066	139.5716812	0	0	Up
Prpf38b	7543.060968	7274.102658	7146.652091	7388.89261	7234.794855	7176.180283	36434.50604	35027.62013	14403.2262	2.310016584	0.04778752	48.33932473	0	0	Up
S100a11	1525.519222	1486.086495	1551.390571	1526.244216	1436.984243	1455.07803	24623.59884	23606.36638	7151.4085	4.059640889	0.06087839	66.68443761	0	0	Up
Nup210l	1958.883281	2158.114063	2050.087734	2186.27044	2126.902925	2128.148883	35670.48093	37099.41984	10672.28851	4.096101911	0.05981392	68.48074441	0	0	Up
Zbtb7b	481.9474739	454.690993	459.485088	451.7087686	472.7605427	469.7878933	29804.49726	27220.17829	7476.882039	5.918928066	0.077834	76.04553037	0	0	Up
Khdc4	9013.195097	8840.260523	9266.115035	8942.770774	9100.900206	9011.562798	50646.50074	50645.18402	19433.31115	2.483489466	0.04261506	58.27727189	0	0	Up
Pip5k1a	4385.139012	4619.355455	4219.82257	4714.7767	4670.458548	4704.687453	32875.63386	35456.45319	11955.79085	2.865652072	0.06138091	46.68637056	0	0	Up
Manba	1960.826618	2089.481461	2093.321561	2015.15253	2077.029285	2083.407179	26033.23926	28483.449	8354.488362	3.711906811	0.06378131	58.19740691	0	0	Up
Trp53inp1	3719.54623	3971.158652	3877.973708	3895.323852	4021.062199	3921.707631	110091.0372	108823.5354	30290.16811	4.784719245	0.04469846	107.0443978	0	0	Up
Tgs1	1954.996608	2094.247614	2190.84903	1988.581426	2000.140758	2001.704937	14072.91019	14154.17108	5057.200205	2.81834207	0.06948067	40.56296571	0	0	Up
Astn2	2219.290384	1839.735045	2176.7729	2086.363089	2055.209568	2144.683861	20687.88279	18645.30395	6481.905199	3.227150386	0.08154477	39.57519736	0	0	Up
Rad23b	3111.281878	3094.186506	3370.227604	3258.680199	3136.844129	3212.648885	16014.45492	16603.87992	6475.275506	2.360891958	0.05812732	40.61587828	0	0	Up
Ubap2	3569.909313	3831.986985	3778.435363	3879.381189	3797.669854	3852.649783	26567.96286	26159.70987	9429.713153	2.784902421	0.05445195	51.14421646	0	0	Up
Clta	2955.814951	2739.584725	2670.442875	2741.075092	2665.12262	2729.243952	24443.16487	22880.92379	7978.17161	3.132914663	0.06150805	50.93503144	0	0	Up
Pum1	5145.955286	5232.282727	5419.3099	5669.210757	5554.676618	5057.757858	70285.61106	74298.00732	22082.85144	3.76838274	0.05281023	71.35705627	0	0	Up
Rragc	1738.314578	1727.253835	1787.668461	1794.080945	1924.291264	1743.953815	22012.00503	21472.02852	6774.949556	3.567901722	0.0633177	56.3492018	0	0	Up
Cap1	1928.761564	1818.763972	1937.478697	1819.589204	1754.928696	1880.124219	14913.99564	14670.55756	5090.524943	3.024426735	0.06880627	43.95568553	0	0	Up
Capzb	4443.439109	4590.758538	4696.400101	4851.883597	4979.051694	4566.571758	28247.31448	28392.32198	10595.96766	2.56918254	0.05515428	46.58174266	0	0	Up
Ptpn12	6693.822879	6829.897202	6670.07456	6878.727413	6750.604936	6787.121987	40361.76424	42728.74711	15462.59504	2.617703821	0.04815836	54.35617113	0	0	Up
Gnl2	992.0733284	1014.237351	871.714598	1017.141863	887.3351725	1075.74619	38154.26735	39410.65133	10427.8959	5.302816075	0.07680401	69.04347928	0	0	Up
Sesn2	512.069191	552.8737442	528.8602982	525.0450158	506.0096358	451.3076242	51011.12773	48367.90181	12806.89938	6.699443789	0.07088507	94.51135161	0	0	Up
Srsf4	4794.211364	4584.085924	4864.308218	4574.481271	4807.611058	4727.058305	25879.11858	24815.13956	9880.751784	2.410602352	0.05499109	43.83623284	0	0	Up
Psmc2	742.3545768	774.9764724	821.4427065	742.9280688	717.9726044	798.5421541	32423.60916	30335.47177	8419.662189	5.369909821	0.07216537	74.41117267	0	0	Up
Rheb	1832.566403	1980.813173	1889.217681	1988.581426	1804.802336	2021.157852	18422.12076	18210.21708	6018.684589	3.258674976	0.07012605	46.46882493	0	0	Up
Dffb	309.9621858	289.7821004	283.5334679	324.1674692	290.9295648	278.1766822	39308.29297	35370.68657	9556.941377	7.036527086	0.08781494	80.12904665	0	0	Up

Ccnl2	6953.258313	6946.191334	7240.157809	7031.776972	7055.041945	7058.490149	33917.82801	32954.92702	13644.70894	2.244322905	0.04599614	48.79372005	0	0	Up
Fosl2	1395.31567	1437.471735	1418.672777	1573.009359	1407.891287	1489.120631	87505.77842	82865.73443	22386.62429	5.877476972	0.05685939	103.3686327	0	0	Up
Klf3	3614.606054	3801.483606	3695.989461	3752.902734	3737.405873	3814.7166	42147.30877	39730.48932	13036.8628	3.438408331	0.0524774	65.52169398	0	0	Up
Rest	556.7659326	590.0497373	649.5128377	548.4275873	564.1955488	563.1618845	19424.84498	19221.54837	5264.81336	5.099332418	0.08030425	63.50015961	0	0	Up
Aff1	2512.734209	3038.899132	2711.665826	2754.892066	2871.890418	2856.660544	22339.0416	25428.01347	8064.224658	3.059818071	0.08041683	38.04947563	0	0	Up
Tfip11	964.8666161	906.5222943	1021.524835	1102.169395	1101.376209	1055.32063	22895.37969	20972.61669	6252.472045	4.346559922	0.0726172	59.8557928	0	0	Up
Ereg	143.8069075	121.0602854	179.9733714	162.6151567	139.2305774	134.2251124	32776.01927	31811.3722	8183.53786	7.884511356	0.10451914	75.43605039	0	0	Up
Cxcl3	798.7113378	769.2570889	719.8934858	762.0592638	840.5786353	781.0345307	54245.78261	48389.34346	13413.33255	5.984664862	0.06978301	85.76106584	0	0	Up
Ccng2	2592.411009	2315.397111	2338.648391	2317.000272	2159.112984	2354.775341	17276.55298	16730.74303	6010.58014	2.91302871	0.07022321	41.48242089	0	0	Up
Sfswap	9016.110102	8347.440306	9003.695761	8952.336372	8811.009675	8699.343515	51464.09219	49705.3249	19249.9191	2.530512373	0.04814975	52.5550474	0	0	Up
Gpn3	698.6295035	674.8872601	695.7629779	692.9743933	682.645443	582.6147994	37646.7968	34965.97538	9580.035819	5.844478101	0.07335394	79.67504185	0	0	Up
Cyp3a13	1721.796217	1628.117853	1548.374257	1667.602489	1641.673973	1603.892828	33683.8277	33133.60746	9578.611597	4.363851861	0.05877307	74.24917683	0	0	Up
Cald1	11370.46238	11778.11721	11499.19245	11172.61783	11512.49849	11393.57222	67097.94419	66047.43798	25233.98034	2.539220567	0.04151804	61.15945047	0	0	Up
Gars	4497.852534	4684.175136	4725.557798	4924.157	4688.122129	4788.334987	48154.25648	47799.69801	15532.76926	3.339829021	0.04738567	70.48183505	0	0	Up
Smarcad1	3435.819088	3434.489828	3454.684382	3389.410031	3442.320171	3459.700904	16654.43167	17129.20042	6800.007062	2.291232216	0.05570002	41.13521106	0	0	Up
Plxna1	5805.718058	5864.27461	5893.876555	5926.419044	6056.530118	5968.154272	40821.30702	39315.9507	14456.5288	2.736511136	0.04609992	59.36042811	0	0	Up
Tmem43	1615.884373	1595.708013	1735.385693	1665.476801	1715.445398	1600.002245	44450.66122	41785.31438	12020.48477	4.701517008	0.05843429	80.45819345	0	0	Up
Edem1	960.0082747	987.5468947	1139.161061	1117.049214	1000.589896	1039.758298	20701.03944	20677.79396	5952.86838	4.341648057	0.06535246	66.43434911	0	0	Up
Dusp16	2718.727887	2868.270855	2884.601132	2764.457664	2863.578144	2886.812562	31323.14988	32361.70796	10083.91326	3.469189597	0.05446126	63.70013351	0	0	Up
Emp1	1063.005114	1234.433618	1132.122996	1093.666642	1168.91343	1052.402693	22754.41565	21900.86158	6424.977715	4.330037366	0.07600063	56.97370174	0	0	Up
Strap	800.6546744	808.3395432	730.9533019	842.83542	812.524713	858.8461901	19313.0135	18097.6484	5283.101969	4.483865645	0.07783381	57.60820187	0	0	Up
Far2	1929.733232	2011.316552	1985.739713	1966.261699	2047.936329	1991.978479	18560.26552	17443.67799	5992.113689	3.155875611	0.0637972	49.46730685	0	0	Up
Vgll4	4052.828455	4360.076734	4408.844882	4335.341335	4216.400621	4390.522879	23902.80271	24496.19497	9270.376573	2.49127808	0.05569945	44.7271566	0	0	Up
U2af2	8370.922354	7839.3684	7842.415069	7700.30595	7891.464444	8248.035891	34153.70784	33227.41469	14409.20433	2.061633177	0.050303	40.98430005	0	0	Up
Mtmr10	1800.501349	1967.467945	1810.793531	1681.419463	1802.724267	1930.701798	23217.71746	23563.48307	7221.851112	3.646986477	0.06591652	55.32735467	0	0	Up
Aen	2856.704785	2502.230308	2803.160668	2834.605379	2791.884787	2598.909422	106935.3222	99444.59909	27845.92708	5.258989722	0.05963598	88.18484177	0	0	Up
Ddias	466.4007812	433.71992	487.6373472	411.3206905	507.04867	517.4475347	24912.10525	24484.58074	6527.532617	5.591174494	0.07736854	72.26677336	0	0	Up
Msn	1556.612607	1834.015662	1704.217121	1680.356619	1729.991876	1778.969062	24790.87617	26329.45629	7675.561926	3.864659972	0.07161726	53.96269166	0	0	Up
Lamp1	1493.454168	1607.14678	1420.683653	1500.735956	1524.263112	1522.190586	20256.53282	19531.55894	6107.070752	3.707125593	0.06560222	56.50915204	0	0	Up
Plpbp	4240.360436	4227.577682	4441.018892	4029.242216	4374.333813	3982.984313	98074.32253	93653.56602	27127.92574	4.520210414	0.05518413	81.91142218	0	0	Up
Tnks	5380.127345	6117.833948	5802.381713	5787.186459	5868.464935	5985.661895	46132.83212	50009.97506	16385.55793	3.019762518	0.06035632	50.03225114	0	0	Up
Mak16	672.3944596	675.8404907	606.2790111	788.6303678	666.0208964	669.1802704	20391.8583	19426.13748	5487.04266	4.898240869	0.08896899	55.05559949	0	0	Up
Msmo1	6771.556342	6772.703366	6886.243693	7201.832038	6935.553017	6807.547547	33831.37006	31733.64621	13367.55653	2.254240976	0.05088137	44.30385549	0	0	Up
Irf2	5452.030799	5586.884508	5564.092947	5541.669458	5798.849646	5873.807635	35238.1912	36076.47431	13141.50006	2.611029653	0.04639213	56.28173627	0	0	Up
Eif2ak3	2396.134013	2361.15218	2546.774022	2407.342026	2397.051807	2422.860543	48072.49734	47494.15445	13762.2458	4.309349553	0.05047067	85.38325038	0	0	Up
Smad1	2393.219009	2494.604463	2318.539634	2499.809468	2524.853008	2422.860543	44065.35951	43321.07277	12755.0398	4.142818247	0.0542792	76.32423869	0	0	Up
Vps35	2602.127692	2790.105947	2583.975221	2823.976937	2601.741536	2932.526912	21504.53448	22100.98367	7492.496549	2.9776404	0.06960952	42.77634085	0	0	Up
Dnaja2	1684.872822	1509.91726	1698.184494	1655.911204	1698.820851	1547.479375	38672.07526	37657.79621	10765.63218	4.555994081	0.06313686	72.16060527	0	0	Up
Gab1	2316.457213	2529.873995	2416.067104	2351.011285	2594.468297	2404.380274	17548.1437	18166.44037	6290.85528	2.837180487	0.06634736	42.762521	0	0	Up
Ist1	2412.652374	2437.410627	2341.664704	2575.271403	2456.276754	2396.599108	34128.33431	34816.77721	10445.62331	3.828682783	0.05678569	67.42337882	0	0	Up
Ap1g1	3225.938737	3403.986449	3396.368987	3449.992148	3222.04493	3342.010769	22737.49996	23762.71177	8317.569218	2.824442494	0.05693692	49.60651707	0	0	Up
St3gal2	407.1290153	393.6842351	426.3056396	487.8454701	438.4724154	426.0188349	20630.55742	21592.63782	5600.331355	5.610428355	0.08125778	69.04481616	0	0	Up
Bcar1	2348.522267	2553.70476	2521.638076	2463.672766	2607.975741	2560.003593	58548.94493	55430.24621	16129.33854	4.463121105	0.05413054	82.45108084	0	0	Up
Ets1	4916.641569	5317.12025	5131.754681	5336.540535	5189.975628	4966.329158	125163.8523	122605.1578	34828.42149	4.608722127	0.04741184	97.20613662	0	0	Up
Ddx6	3351.283947	3592.726106	3420.499495	3540.333902	3589.863022	3401.342159	18092.2649	19223.33518	7276.456089	2.416381266	0.06258329	38.61064461	0	0	Up
Vps11	2097.831847	2139.049452	2185.821841	2291.492012	2191.323043	2195.261439	20510.2681	19050.01515	6582.632861	3.172837265	0.06328329	50.13704659	0	0	Up
Polr2m	2875.166483	2608.038903	2752.888777	2906.878782	2644.341937	2666.021978	23195.16322	22687.94892	7792.056124	3.111034264	0.0618454	50.30340421	0	0	Up
Tcf12	7921.039934	8040.500055	8114.88872	8041.478925	8044.202465	7964.023334	32734.66982	34171.74082	14379.06801	2.063381206	0.04605909	44.79857132	0	0	Up
1700017B0	5577.376009	4992.068618	5165.939567	5562.926341	5096.462554	5066.511669	39546.99208	35594.93053	13325.40092	2.886284441	0.06515453	44.2990607	0	0	Up
Neo1	4539.63427	4752.807739	4704.443603	4619.120726	4713.058949	4785.41705	40323.23407	41886.26883	13790.49816	3.113490329	0.0490276	63.50484377	0	0	Up

Ctsh	2845.044765	2570.86291	2504.545633	2566.76865	2550.828862	2616.417046	19160.77234	18674.78623	6686.253304	2.87215135	0.06364195	45.1298431	0	0	Up
Rpl4	3634.03942	3590.819645	3613.543559	3407.478382	3702.078711	3380.916599	142093.6338	132014.4698	36929.62248	5.274644775	0.05311136	99.3129384	0	0	Up
Smad3	3716.631225	4535.471163	4203.735564	4085.572957	4353.55313	4082.194179	37727.61618	38428.80231	12641.69709	3.174523452	0.07158377	44.3469753	0	0	Up
Ptgs2	26.23504394	15.25168949	40.21751317	30.82248068	37.40522975	23.3434978	21325.98002	20183.74255	5210.374753	9.426717449	0.19857761	47.47120076	0	0	Up
Trib1	69.96011718	100.0892123	153.8319879	114.7871694	76.88852783	88.51076251	104932.693	97132.47419	25333.65438	10.25308648	0.20349499	50.384959	0	0	Up
Pdcd6ip	5318.912242	5455.338686	5583.196266	5360.98595	5631.565146	5355.387455	48393.89535	52100.53621	16649.97716	3.193427927	0.0525009	60.82615087	0	0	Up
Amotl2	7576.09769	7365.612795	7212.00555	7326.184805	7326.229861	7023.474902	204039.8121	185339.8604	54151.15976	4.762192979	0.04884202	97.5019586	0	0	Up
Cish	848.2664208	914.148139	852.6112793	949.1198362	856.1641477	907.4784771	27990.75992	25810.38961	7391.11723	4.930481475	0.07357023	67.0173498	0	0	Up
Ip6k2	9541.782649	9469.392715	9632.094405	9815.365831	9690.032575	9762.445311	51644.52616	49800.02554	19919.45815	2.382631223	0.04271353	55.78165522	0	0	Up
Flcn	2821.724726	2766.275182	2997.210169	2916.444379	2911.373716	2937.39014	34794.62435	32855.75938	10625.10025	3.531824574	0.05384814	65.58860436	0	0	Up
Pygm	535.3892301	504.2589839	516.7950443	565.4330939	483.1508843	497.9946198	25479.72046	23746.63053	6541.171605	5.648419954	0.07808377	72.33795442	0	0	Up
Resf1	10734.99131	10168.11074	10496.77094	10480.70628	11035.58181	11153.32872	93645.23233	93521.34249	31404.50808	3.076384872	0.04109097	74.86765552	0	0	Up
Gtf3c1	6825.969767	6975.741482	7114.47808	7151.878362	7048.80774	6824.082525	79879.62376	77629.50413	24931.26073	3.505207498	0.04245018	82.5722605	0	0	Up
Senp1	4747.571285	4766.152967	4774.824251	4606.366596	4692.278266	4903.107185	21599.45026	21273.69323	8920.430506	2.159497214	0.05266958	41.0008469	0	0	Up
Larp4b	6054.465141	6049.201346	6016.539971	5994.441071	6291.351838	6435.024228	38124.19502	39390.99648	14294.52689	2.606593439	0.04582282	56.88418169	0	0	Up
Igf2bp2	2861.563126	3251.469554	3393.352674	3354.336174	3285.426013	3204.867719	21411.49821	22997.95948	7970.059119	2.774618803	0.06668587	41.60729457	0	0	Up
Pcgf3	1994.835008	1982.719634	1989.761464	2109.74566	1816.231711	2238.057852	56549.13506	55428.45941	15513.61822	4.786658592	0.06963165	68.74257201	0	0	Up
Sf3b3	3576.710991	3997.849109	3923.21841	3855.998618	4013.788959	3902.254716	50092.98194	48598.39958	15245.15029	3.64017281	0.05519195	65.95477391	0	0	Up
Atp6v1h	2038.560081	1903.601495	1835.929476	1963.073166	1977.282006	1968.634982	13877.44005	15589.86842	5144.29871	2.900753254	0.07545688	38.44252774	0	0	Up
Klf9	864.7847818	938.9321345	823.4535822	925.7372646	874.8667626	874.4085219	30615.51039	30317.60372	8279.412144	5.122399139	0.06754345	75.8385821	0	0	Up
Usp36	1840.339749	1869.285194	1913.348189	1876.982789	2024.038543	1962.799107	15870.6716	15285.21827	5330.33543	2.966336498	0.0632447	46.90252941	0	0	Up
Ube2f	1352.562266	1376.464977	1381.471577	1416.771267	1431.789072	1290.700899	18285.85552	18673.89282	5651.18855	3.763677016	0.06801444	55.33644149	0	0	Up
Lif	690.8561572	611.0208104	625.3823298	624.9523669	624.4595301	700.3049341	31670.86118	29309.84604	8107.210418	5.523345014	0.07582588	72.8424791	0	0	Up
Eda2r	1783.01132	1790.167054	1710.249748	1703.739191	1619.854255	1753.680273	133148.0557	135930.2516	34929.87614	6.316831953	0.04839542	130.525408	0	0	Up
Morc2a	3413.470718	3868.209748	3704.032963	3449.992148	3627.268252	3752.467272	20946.31687	21105.73362	7983.436448	2.510438022	0.06585194	38.12246172	0	0	Up
Rnps1	2698.322853	2698.59581	2665.415685	2729.383807	2772.143138	2714.654266	20063.88197	19313.5688	6956.995791	2.843415921	0.05730865	49.61582915	0	0	Up
Ttyh2	2528.280901	2718.613652	2618.160108	2649.670494	2594.468297	2574.593279	23229.93435	23077.47228	7748.89917	3.163305686	0.05751785	54.99693987	0	0	Up
Eef2	1407.947358	1470.834806	1358.346507	1387.011631	1504.521463	1424.926012	51182.1641	45265.11596	13125.10848	5.041384757	0.0656908	76.74415539	0	0	Up
Dcbld2	2304.797194	2298.238961	2249.164424	2348.885597	2561.219204	2279.881619	20583.5694	21690.91206	7039.583557	3.126900843	0.06638544	47.10220808	0	0	Up
Eif2s3x	1901.554852	1766.33629	2011.881096	1983.267205	1841.168531	1872.343053	15552.09287	15630.07152	5319.839427	3.06977199	0.06469306	47.45133575	0	0	Up
Arl6ip5	3073.386815	3105.625273	3029.38418	3125.824679	2933.193433	2924.745746	68022.66831	66517.36754	19091.5245	4.521511914	0.04607585	98.13193717	0	0	Up
Tut7	3120.998561	3371.576609	3209.357551	3220.417809	3357.11937	3400.369514	36918.48258	36541.93686	11642.53236	3.442341956	0.05249832	65.57051939	0	0	Up
Oser1	943.4899137	947.5112098	986.3345106	985.2565377	943.4430171	999.8798226	79516.87629	74941.2569	20033.00603	6.311853386	0.05749485	109.7811916	0	0	Up
Prdm4	1028.025055	1048.553653	1088.889169	1154.248759	1072.283253	985.2901365	19621.25488	19347.51808	5668.257873	4.244010415	0.0690609	61.45316116	0	0	Up
Midn	1541.065915	1445.09758	1412.64015	1585.763489	1660.376587	1594.166371	20026.29156	16763.79892	5753.65007	3.498896701	0.08617549	40.6019938	0	0	Up
Gtf3c4	539.2759033	524.2768264	603.2626976	522.9193274	605.7569152	545.6542612	22151.08955	21182.56621	5834.350211	5.235115244	0.07947019	65.87520916	0	0	Up
Ggta1	1989.004998	2085.668538	2066.174739	2137.379609	2191.323043	2061.036327	63800.32537	64511.6796	17605.32403	4.91571785	0.05103537	96.31981815	0	0	Up
Ythdc1	4462.872475	4436.335182	4653.166274	4729.656518	4519.798595	4783.471758	46836.71257	47251.14906	15209.1453	3.337954371	0.04740988	70.40630056	0	0	Up
Fnip1	7481.845865	7482.860158	7561.897914	7616.341261	7228.56065	7872.594635	49884.35516	53538.02035	18583.3095	2.775640936	0.05266748	52.70122582	0	0	Up
Tmem63b	3459.139127	3381.108915	3528.081343	3577.533447	3376.861019	3371.190141	34302.18996	32213.40319	10901.18839	3.30113205	0.05331223	61.92072788	0	0	Up
Slf2	2519.535887	2692.876426	2620.170983	2405.216337	2486.408744	2657.268167	45547.36147	44935.45055	13233.03607	4.136420761	0.05759544	71.81855121	0	0	Up
BC031181	1217.500373	1141.01702	1244.732033	1244.590513	1195.928318	1118.542603	26539.77005	23994.99634	7212.134656	4.448975855	0.07104621	62.62087598	0	0	Up
Srp72	3724.404572	3809.109451	3719.114531	3830.490358	3663.634448	3824.443057	36281.32511	36321.26652	11896.72351	3.277145489	0.04984777	65.74307423	0	0	Up
Sec24a	3474.68582	3480.244896	3787.484303	3442.552239	3590.902056	3827.360994	30957.58313	32638.66264	10649.93451	3.099581303	0.05969496	51.92366791	0	0	Up
Fam13b	8508.899252	8519.975044	8617.607635	8460.239525	8459.816129	8569.008986	64462.85637	68474.81176	23009.15184	2.964680334	0.04483457	66.12486844	0	0	Up
Tmtc3	612.1510253	709.2035615	680.6814104	620.7009903	626.5375984	701.2775799	74680.86989	73797.70208	19053.64052	6.803634736	0.06618036	102.8044396	0	0	Up
Cab39	2327.145565	2756.742876	2694.573383	2537.009013	2700.449781	2686.447539	25491.93734	25680.84629	8359.393974	3.247870702	0.07010379	46.32946018	0	0	Up
Cyld	4466.759148	4405.831803	4465.1494	4383.169322	4362.904437	4591.860547	28376.06164	27850.02684	10362.72039	2.650300421	0.05113332	51.83117761	0	0	Up
Tubb4b	382.8373079	333.6307077	344.8651755	364.5555474	310.6712138	370.5780276	42937.64717	37599.72507	10330.56378	6.882991445	0.08961406	76.80705244	0	0	Up
Siah1a	2714.841214	2728.145958	2668.431999	2617.78517	2796.040924	2666.994624	40619.25856	41021.4555	12229.11924	3.901815032	0.05041948	77.38705303	0	0	Up

Otud4	4883.604847	4793.796654	4830.123332	4753.03909	4673.575651	4975.08297	29093.09873	29510.86153	10939.14785	2.602375498	0.05129035	50.73811606	0	0	Up
Rbmxl1	678.2244693	625.3192693	664.5944052	765.2477962	620.3033934	655.56323	69224.62171	64743.96417	17247.2298	6.713578263	0.06843745	98.098022	0	0	Up
Fam91a1	2428.199067	2620.430901	2611.122043	2516.814974	2621.483185	2503.59014	22761.93373	22066.14098	7516.214378	3.128963205	0.06089806	51.38034418	0	0	Up
Spred3	803.5696793	871.2527623	879.7581006	793.9445886	813.5637471	779.0892392	20641.83454	19691.47793	5659.311323	4.662843726	0.07485525	62.29147233	0	0	Up
Mex3c	2248.440433	2242.951586	2248.158986	2289.366324	2224.572136	2274.045744	94179.95593	93796.51037	25188.00019	5.384730373	0.04519062	119.1559401	0	0	Up
Prkci	3436.790757	3454.50767	3391.341798	3487.191694	3496.349948	3711.616151	22562.70455	22440.47651	8247.622384	2.642107218	0.05496331	48.07038463	0	0	Up
Cd81	3408.612376	3499.309508	3433.570187	3644.49263	3344.65096	3340.065478	59933.21182	56875.77098	17184.96049	4.127134445	0.05065639	81.47313163	0	0	Up
Ckap2	2191.112003	2111.405764	2263.240554	2035.346569	2189.244975	2070.762784	17324.48075	17454.39882	5954.999028	3.029549272	0.06730269	45.01379112	0	0	Up
Fam222b	2158.075281	2383.076483	2237.09917	2321.251649	2458.354822	2531.796866	15798.31006	16138.41737	5753.297713	2.677947722	0.06595447	40.60297616	0	0	Up
Dusp8	61.21510253	65.77291094	93.50571813	70.14771466	48.83460551	58.35874451	61771.38293	56131.56694	14787.59808	10.09926386	0.15114301	66.81926006	0	0	Up
Arap2	2241.638755	2197.196518	2076.229118	2175.641998	2296.265493	2301.279825	50471.70533	53500.49746	14657.55681	4.499192146	0.05295611	84.96077584	0	0	Up
Dennd4c	3613.634386	3876.788823	3537.130284	3520.139863	3645.970867	3892.528259	21217.90759	21517.59203	8102.711513	2.502856689	0.05854398	42.75173761	0	0	Up
Smim3	2419.454053	2533.686917	2554.817524	2551.888832	2481.213574	2351.857404	47227.65285	45927.12699	13505.96227	4.268961342	0.05213829	81.87766119	0	0	Up
F11r	2130.868569	2425.97186	2292.398251	2273.423661	2329.514586	2342.130946	48645.75111	51897.73391	14292.22411	4.427730682	0.06059834	73.06686468	0	0	Up
Usp38	1528.434227	1497.525262	1491.064301	1355.126306	1507.638566	1551.369958	27331.98797	27841.98622	8013.141601	4.172649597	0.06158005	67.75976473	0	0	Up
Atg9b	608.2643522	540.4817464	631.4149568	596.2555746	603.6788469	503.8304943	35878.16795	32938.84578	9037.617463	5.959296336	0.08070893	73.83688902	0	0	Up
Tsr1	3939.143265	3648.013481	3673.869828	3876.192657	3817.411503	3618.24216	23010.03044	22358.2835	8492.648355	2.609327799	0.05994689	43.52732382	0	0	Up
Sgsm2	2487.470833	2359.245719	2371.827839	2413.719091	2392.89567	2347.966821	13893.41597	13388.5254	5206.883418	2.524788934	0.06386096	39.53571749	0	0	Up
Phrf1	2943.183263	3176.164337	3221.422805	3200.22377	3201.264246	3099.821979	33404.71889	33976.97914	10777.9723	3.418848712	0.05409529	63.20048803	0	0	Up
Golga4	8123.146939	8550.478423	8582.417311	8793.972592	8028.616953	8569.008986	66075.48501	66672.81952	22924.49322	2.999478922	0.04707693	63.71440928	0	0	Up
Sde2	476.1174642	532.8559017	497.6917255	565.4330939	419.7698006	544.6816154	46064.22962	44297.56138	11674.79257	6.548721412	0.14614625	44.80937106	0	0	Up
Ythdf1	1606.16769	1752.991061	1722.315002	1635.717164	1658.298519	1606.810766	23150.99449	21792.75991	6865.756825	3.783084143	0.06664405	56.76552279	0	0	Up
Irs2	438.2224007	454.690993	562.0397466	486.7826259	484.1899185	477.5690592	29955.79867	28393.21538	7656.563599	5.923050147	0.07655126	77.37364781	0	0	Up
Pogz	4490.079187	4826.206494	4628.030328	4623.372102	4841.899185	4827.240817	21977.2339	21715.03392	8991.136991	2.175921651	0.05402178	40.27860129	0	0	Up
Dido1	4524.087578	4546.90993	4784.87863	4788.112947	4739.034803	4801.952028	33970.45458	33641.05991	11974.5613	2.82499562	0.04677105	60.40051508	0	0	Up
Lmtk2	1560.49928	1797.792899	1624.787532	1693.110749	1900.393478	1903.467717	16348.06982	18498.78599	5665.863434	3.19552802	0.07956413	40.16292414	0	0	Up
Ppp1r10	1474.992471	1266.843459	1238.699406	1253.093266	1240.606787	1224.560989	136073.5294	127328.5752	33887.61263	6.739549031	0.06011067	112.1190214	0	0	Up
Rpl22l1	3392.094015	3403.986449	3458.706133	3295.879745	3259.450159	3282.679379	18103.54202	17523.19079	6964.941086	2.445076094	0.05714348	42.78836709	0	0	Up
Isg20	2160.990286	2165.739908	1982.723399	1976.89014	2021.960475	1970.580273	28286.78441	25687.99351	8281.707801	3.757015439	0.06278063	59.8435486	0	0	Up
Rab3gap2	2721.642892	2838.720707	3002.237358	3023.791639	2897.866272	2994.776239	14906.47755	15552.34553	5992.232274	2.369762449	0.06076367	38.99966199	0	0	Up
Dusp10	1514.830871	1676.732614	1742.423758	1765.384152	1511.794703	1714.774443	30059.1723	28819.36823	8600.560134	4.18892428	0.06845081	61.19612062	0	0	Up
Cep104	2054.106774	1844.501198	2018.919161	2067.231894	2077.029285	1916.112111	31282.74018	29311.63284	9071.534181	3.923983238	0.06602256	59.43397599	0	0	Up
Cpeb2	1064.94845	990.4065865	1188.427514	1095.79233	964.2237003	1035.867715	50576.95848	47651.39324	13071.00225	5.617288402	0.0638267	88.00844197	0	0	Up
Wdr70	3124.885234	3093.233276	3058.541877	3109.882016	3076.580147	3144.563683	27208.87937	29768.16137	9448.090872	3.195070289	0.05860782	54.51610531	0	0	Up
Ankrd13c	2572.005975	2606.132442	2699.600572	2483.866805	2727.46467	2661.15875	28381.7002	28817.58142	9118.688855	3.408142397	0.05669033	60.11858326	0	0	Up
Edrf1	2837.271419	2917.838846	2788.079101	2998.283379	2929.037297	3038.545298	15828.38239	15915.06682	6156.563069	2.411093456	0.0615333	39.1835568	0	0	Up
Lats1	2434.029077	2444.083241	2362.778899	2509.375065	2403.286012	2453.985207	17290.64938	17203.3528	6137.69246	2.828017319	0.06019367	46.98197379	0	0	Up
Ythdf2	4373.478992	4104.610935	4017.729566	4280.073438	4394.075462	4245.598663	23010.03044	23597.43236	9002.878732	2.431638069	0.05732917	42.41536938	0	0	Up
Elavl1	3906.106543	4326.713663	4066.99602	4282.199126	4144.707264	4451.79956	36675.08467	37062.79035	12364.54965	3.100363037	0.05907519	52.48164797	0	0	Up
Prrc2c	10193.77207	10526.52544	10861.74487	10036.43742	9954.986285	10778.86011	43590.78056	45223.12606	18895.7791	2.098679704	0.05382528	38.99059586	0	0	Up
Suco	1686.816159	1854.986735	1866.092611	1802.583698	1733.108979	1885.960093	15370.71913	17087.21051	5410.93474	3.164560646	0.07685542	41.17550329	0	0	Up
Slc25a24	3010.228375	2959.780992	2926.829521	3029.10586	3022.550371	3158.180724	20547.85851	20942.24101	7449.596921	2.746725105	0.05640991	48.69224628	0	0	Up
Trim41	2010.381701	2117.125148	1965.630956	1947.130504	2148.722643	1991.005834	20668.14783	19348.41149	6524.569512	3.273343335	0.06574075	49.79169701	0	0	Up
Aqr	1161.143612	1148.642865	1161.280693	1135.117564	1283.207187	1157.448433	24280.58634	25702.28795	7128.71433	4.356905159	0.06714317	64.88977326	0	0	Up
Rc3h1	1827.708061	2239.138664	2048.076858	1993.895647	2104.044174	2065.899556	18559.32576	19378.78716	6277.109485	3.185627048	0.07903929	40.30434785	0	0	Up
Pitpnc1	2178.480316	2201.962671	2269.273181	2329.754402	2335.748791	2165.109421	15589.68328	17180.12434	5781.26705	2.864460852	0.06966664	41.11668072	0	0	Up
Mybbp1a	3235.65542	3252.422785	3314.928523	3403.227005	3396.602668	3199.031845	23237.45243	21407.70356	8055.87803	2.759109543	0.06105266	45.19229261	0	0	Up
Ppp1r13l	850.2097574	878.8786071	799.3230743	886.4120306	847.8518744	861.7641273	38975.61783	36359.68281	10057.46751	5.461394685	0.06682022	81.73266325	0	0	Up
Bsdc1	1032.883397	1006.611507	979.2964457	929.9886413	992.2776226	898.7246655	25925.16683	24853.55585	7077.31312	4.747896468	0.06823807	69.57841435	0	0	Up
Slc19a2	1450.700763	1537.560947	1660.983294	1524.118528	1387.110603	1620.427806	153506.0826	144883.0351	38446.25245	6.631315285	0.06150829	107.8117254	0	0	Up

Arhgef1	7103.866899	6764.124291	6888.254569	7027.525595	7002.051203	6612.045753	29367.50873	29972.75047	12592.26594	2.124074319	0.05089673	41.73301986	0	0	Up
Wdr43	1602.281017	1694.843995	1708.238872	1655.911204	1646.869143	1671.97803	20266.87019	22406.52722	6581.689959	3.684536719	0.06862131	53.69376498	0	0	Up
Uspl1	1931.676569	1760.616906	1953.565702	1862.102971	1947.150016	1919.030049	41169.01832	39418.69195	11495.23156	4.381661424	0.05847718	74.9294267	0	0	Up
Pcf11	2257.185447	2303.958344	2281.338435	2036.409413	1932.603537	2231.249332	46846.11017	44577.19627	13058.25637	4.455877843	0.0673508	66.15924014	0	0	Up
Wrap53	1474.992471	1670.06	1676.064861	1631.465788	1692.586646	1517.327357	32873.75434	31763.12848	9287.422493	4.332390819	0.0678581	63.84485469	0	0	Up
Ddx24	2669.172804	2634.72936	2746.85615	2668.801689	2721.230465	2518.179826	37416.55553	36884.1099	11282.45447	3.826340684	0.05408248	70.7500923	0	0	Up
Rnf10	3160.836961	2933.090536	3105.797455	2953.643925	3011.120995	2909.183414	18832.796	18163.76017	6883.778682	2.643767957	0.06211516	42.56236272	0	0	Up
Enc1	127.2885465	197.3187328	221.1963224	188.1234166	197.4164904	180.912108	148331.7625	138016.3458	35932.54549	9.564350889	0.21701801	44.07169283	0	0	Up
Phlda3	231.257054	321.23871	281.5225922	262.5225079	231.7046176	247.0520184	101317.4353	97089.59089	24997.79045	8.69446366	0.15114117	57.52544937	0	0	Up
Ppp4r3a	3675.821157	3803.390068	3795.527806	3668.938045	3971.188559	3797.208976	50528.09095	52245.26736	15685.67912	3.725930033	0.04932439	75.53930821	0	0	Up
Ammecr1l	2545.770931	2668.092431	2682.508129	2614.596637	2538.360452	2493.863682	34779.58818	34634.52316	10619.66295	3.786070756	0.05243392	72.20652015	0	0	Up
S100a10	1010.535026	982.7807418	964.2148783	1103.23224	1125.273995	904.5605399	64833.12192	61951.18889	16609.36353	5.966491208	0.074559	80.02375631	0	0	Up
Kdm5b	2189.168667	2412.626632	2297.42544	2378.645233	2237.040546	2260.428704	21715.98054	22495.86744	7248.3979	3.297210706	0.06281486	52.49093453	0	0	Up
Atf4	1272.885465	1111.466872	1137.150185	1236.08776	1137.742405	1123.405832	42747.81559	37714.97395	10935.19101	5.153257248	0.07479577	68.89770873	0	0	Up
Usp22	1159.200275	1202.023778	1373.428075	1323.240981	1240.606787	1320.852917	18635.44634	17649.1605	5487.994957	3.823849024	0.06871797	55.64554512	0	0	Up
Sem1	2088.115164	2089.481461	2061.14755	2065.106206	2087.419627	2013.376686	14144.33197	13679.77452	5028.594147	2.762531537	0.06366217	43.39361125	0	0	Up
Maff	989.1583235	909.3819861	988.3453862	937.4285504	875.9057967	810.213903	62021.35917	58069.35632	15700.14368	6.155127532	0.06395722	96.23820409	0	0	Up
Dhx9	5042.958447	5355.249474	4932.677991	5032.567104	5114.126135	5295.083419	40816.60822	43587.30663	14397.07218	3.019358575	0.05300924	56.95910019	0	0	Up
Bmt2	2951.928278	3312.476312	3115.851833	3163.024224	3116.063445	3222.375343	55974.94153	54652.9863	16188.70591	4.125282622	0.0553497	74.53125333	0	0	Up
Tmem11	510.1258545	489.960525	520.8167956	579.250068	499.7754309	522.3107634	19488.74868	18614.92828	5153.239549	5.219805515	0.07951101	65.64884019	0	0	Up
Ptpn11	1581.875983	1699.610148	1572.504765	1665.476801	1757.006764	1695.321528	16387.53975	17836.77496	5524.513838	3.309601489	0.07125354	46.44824134	0	0	Up
Thrap3	9592.3094	9601.891767	9618.018275	9412.547894	9388.712668	9225.544861	45800.15698	48239.25189	18859.80422	2.336921447	0.04505512	51.86805011	0	0	Up
Cep68	1320.497212	1225.854543	1246.742908	1335.995111	1315.417246	1380.184308	24527.74329	26289.25319	7330.210977	4.236323569	0.06778798	62.49373061	0	0	Up
Eml6	1704.306188	1787.307363	1691.146429	1840.846088	1823.50495	1741.035878	34066.31013	34872.16815	9940.828147	4.27384692	0.05928653	72.08799676	0	0	Up
Tnip3	1444.870753	1456.536347	1495.086052	1375.320345	1336.19793	1424.926012	30734.85994	33474.8871	9092.83556	4.539037317	0.06629071	68.47169924	0	0	Up
Ankrd50	10523.16763	9850.684952	9957.856261	9988.609429	10262.5404	9801.351141	56769.03896	53326.28403	21309.9416	2.456151402	0.05011767	49.00769036	0	0	Up
Med26	1746.087925	1880.723961	1880.168741	2015.15253	1875.456658	1933.619735	39017.90704	38386.81241	11091.99113	4.344694801	0.05753257	75.51713871	0	0	Up
Lhfpl2	2060.908452	2198.149748	2169.734836	2072.546115	2068.717012	2254.59283	63898.06044	64970.88833	17711.69972	4.897049509	0.05435077	90.10083625	0	0	Up
Basp1	7739.337963	7673.506277	7535.756531	7612.089884	7563.129649	7703.354275	140472.5473	136644.9734	40368.08691	4.181980812	0.03906444	107.0533815	0	0	Up
Rpl37a	394.4973274	473.7556049	386.0881265	417.6977554	406.2623565	339.4533639	29694.54531	27001.29475	7389.199324	6.250623347	0.09097165	68.70957649	0	0	Up
Cd24a	365.3472786	380.3390068	451.4415854	516.5422625	445.7456546	428.9367722	26810.42101	24366.65165	6720.678153	5.871038262	0.08319383	70.5705937	0	0	Up
Rab2a	1770.379632	1879.77073	1906.310124	1609.14606	1778.826482	1839.273098	18743.51877	20472.31146	6249.942045	3.438022555	0.07857661	43.75376307	0	0	Up
Tgif1	3661.246133	3619.416563	3711.071028	3705.074747	3600.253364	3390.643056	114867.8387	109762.5011	30789.75559	5.006275153	0.046304	108.1175548	0	0	Up
Sned1	86.47847818	125.8264383	120.6525395	119.0385461	108.0595526	87.53811677	36025.71031	33456.12566	8766.178705	8.476795205	0.1216172	69.70062577	0	0	Up
Lonp2	10657.25785	10217.67873	10448.50992	10166.1044	10735.30094	10425.78971	63240.22825	61581.32037	23434.02377	2.560441868	0.0438015	58.4555716	0	0	Up
Arf1	1279.687143	1179.146244	1336.226875	1177.631331	1261.38747	1388.938119	119448.2303	114769.1271	30230.04682	6.464702683	0.05990242	107.9205561	0	0	Up
Inka2	133.1185563	166.8153538	152.8265501	156.2380917	230.6655835	160.4865474	19835.52022	19387.72118	5027.924011	6.653252696	0.10782443	61.70449978	0	0	Up
Foxo3	1757.747944	2046.586084	2247.153549	1929.062153	2113.395481	2125.230946	46965.45973	48637.70927	13477.79314	4.495371423	0.07089099	63.41245296	0	0	Up
Bdp1	1722.767886	1899.788573	2120.468382	2042.786478	2024.038543	2249.729601	28234.15784	33933.20243	9278.367466	3.862262851	0.08144875	47.41954528	0	0	Up
Rlf	2496.215848	2536.546609	2659.383059	2642.230585	2630.834493	2680.611665	51589.0803	53815.86844	15131.34637	4.310582776	0.04941672	87.22924155	0	0	Up
Lgals3	7907.436578	7605.826905	8144.046417	7364.447195	7724.179944	7710.162796	106461.683	103850.8588	32096.0802	3.768319955	0.04909638	76.75352736	0	0	Up
Tor1aip2	20137.8254	19738.5459	20652.69845	20444.87029	20254.93191	19585.19466	98475.60017	96465.10275	39469.34619	2.290791023	0.03859391	59.35629038	0	0	Up
Micos10	2111.435203	2274.408196	2314.517883	2177.767687	2268.211571	2227.358749	18711.56692	18870.44131	6369.46344	3.063562358	0.06302534	48.608424	0	0	Up
Gja1	4408.459051	4181.822613	4205.74644	4267.319308	4178.995391	4519.884762	29344.01473	27298.79768	10300.63	2.702724293	0.05940804	45.49425289	0	0	Up
Ppp1r37	1216.528704	1447.004041	1483.020798	1339.183643	1266.582641	1488.147985	29422.01483	28325.31681	8248.474932	4.388992524	0.08255756	53.16281794	0	0	Up
Crebzf	6208.9604	6167.401939	6417.709664	6384.504878	6536.563899	6347.486111	26744.63779	25499.48565	11288.34379	2.019743066	0.04966164	40.67008413	0	0	Up
Rap2a	366.3189469	305.0337899	385.0826886	366.6812357	363.6619559	339.4533639	21966.89653	20818.95151	5614.010003	5.92813661	0.08721743	67.96962949	0	0	Up
Erich1	1375.882305	1406.015125	1472.96642	1477.353384	1319.573383	1376.293725	16589.58821	16736.99685	5219.333676	3.627543548	0.06507873	55.74085078	0	0	Up
Ppp4r2	8415.619096	8503.770123	8221.46513	8617.540461	8426.567036	8415.330959	104576.5239	106154.0496	32666.35829	3.645280641	0.0407687	89.41371967	0	0	Up
Zfp622	1814.104705	1704.376301	1794.706525	1740.938736	1666.610792	1698.239465	20621.15981	19981.83365	6377.746248	3.59283237	0.06273215	57.27258832	0	0	Up

Ezr	4552.265958	4907.231095	4745.666554	4622.309258	4770.205828	5005.234988	36592.38576	35297.42759	12561.59088	2.878382477	0.05392082	53.3816536	0	0	Up
Atp6v1a	1994.835008	2011.316552	2110.414004	2075.734647	2015.72627	1971.552919	19760.3394	19703.98556	6455.488045	3.307213002	0.0584757	56.55704691	0	0	Up
Magi3	7784.034705	8181.578183	7911.790279	7805.527522	7500.7876	8250.953828	33759.00852	34804.26958	14499.74378	2.121737713	0.05421215	39.1376777	0	0	Up
Prkg1	6799.734723	6800.347053	6805.808667	6672.535646	6828.532498	6765.72378	41002.68075	38705.75699	15047.64001	2.551739933	0.0476929	53.50356199	0	0	Up
Pom121	676.2811328	801.666929	723.9152371	729.1110947	737.7142535	806.32332	39153.23252	35744.1287	9921.546648	5.599306978	0.07586214	73.808977	0	0	Up
Zfand2a	438.2224007	533.8091323	496.6862877	502.7252884	495.6192942	591.368611	19441.76066	18547.92311	5131.014349	5.125571907	0.09009314	56.89192388	0	0	Up
Sh3pxd2a	2827.554736	2797.731792	2708.649512	3083.310912	2900.983374	2876.113459	24896.12932	24153.12853	8280.450205	3.085837222	0.06351875	48.5815171	0	0	Up
Adnp2	1018.308372	1058.085959	1003.426954	955.4969011	964.2237003	1042.676235	24258.03209	24680.23583	6872.560755	4.607207325	0.06608293	69.71857391	0	0	Up
Pla2r1	149.6369173	152.5168949	147.7993609	110.5357928	128.8402358	147.8421528	31046.86035	30302.41588	7773.305949	7.790012266	0.10526658	74.00271017	0	0	Up
Srsf11	20298.15067	20024.51508	20405.36075	20504.38956	20535.47113	20777.65834	109838.2417	107095.6955	42434.93535	2.392557267	0.03694113	64.76676689	0	0	Up
Foxk1	804.5413476	835.0299998	917.9647382	859.8409266	826.0321571	875.3811677	20786.55762	20265.93555	5771.410438	4.592094215	0.0699823	65.61793869	0	0	Up
Rlim	769.561289	754.95863	645.4910864	792.8817444	705.5041945	717.8125575	21208.50999	21351.41922	5868.267339	4.902012851	0.07945486	61.6955694	0	0	Up
Retsat	1467.219124	1344.055137	1273.88973	1314.738228	1401.657082	1241.095967	23994.89922	25579.89184	7202.180791	4.230292006	0.07168162	59.01501568	0	0	Up
Elmod3	909.4815234	1026.629349	1120.057742	1047.964343	875.9057967	987.235428	115931.6474	112439.1341	29292.25696	6.936175581	0.06106618	113.5845711	0	0	Up
Txnl4a	6628.721103	6286.555763	6665.04737	6647.027386	6785.932098	6586.756964	43509.02142	40977.67879	15510.84261	2.659497372	0.05031005	52.86215345	0	0	Up
Mpp7	4078.091831	4303.836129	4338.464233	4542.595946	4138.473059	4601.587005	28876.95387	33826.88757	11088.36121	2.842654938	0.07303517	38.9217282	0	0	Up
Bloc1s2	1435.154071	1477.50742	1380.46614	1378.508877	1190.733147	1311.12646	49020.71546	47387.83959	13072.7564	5.26716569	0.05812082	90.6244151	0	0	Up
Rpl36	266.2371126	332.6774771	277.5008409	329.48169	287.8124623	310.2739917	20771.52146	18137.8515	5089.169567	6.02271087	0.10403417	57.89165859	0	0	Up
Arhgap35	3155.006951	3507.888584	3147.020406	3215.103588	3424.656591	3693.135882	77266.15042	84494.40665	22737.92113	4.506023733	0.0597501	75.41449139	0	0	Up
Upf1	3101.565195	3070.355741	3126.911649	3123.69899	3158.663846	3188.332742	18760.43446	17539.27203	6883.654331	2.515732837	0.05909722	42.56939232	0	0	Up
Abce1	1692.646168	1805.418744	1700.195369	1653.785515	1715.445398	1889.850676	17139.34798	17407.9419	5625.578969	3.259873088	0.06812817	47.84911847	0	0	Up
Txndc9	3947.888279	3774.79315	3793.51693	3831.553202	3911.963612	3792.345747	17721.05959	17084.53031	7232.206352	2.175673561	0.05690455	38.23373992	0	0	Up
Cxcl2	146.7219124	137.2652054	139.7558583	163.6780009	129.87927	142.9789241	36923.18138	33842.96881	8953.30367	8.016911757	0.10716773	74.80714227	0	0	Up
Fam133b	3816.71306	3761.447921	3683.924207	3793.290812	3837.153152	3849.731846	20120.26758	21262.079	8015.575948	2.428563982	0.05569864	43.60185379	0	0	Up
Rpl30	2448.604101	2710.034577	2508.567384	2581.648468	2757.59666	2704.927808	15810.52694	15707.79751	5903.712932	2.528625194	0.06480425	39.01943684	0	0	Up
Eif4b	5904.828224	6403.803126	6253.823298	6011.446577	6478.377987	6385.419295	44443.14314	43385.39773	15658.27992	2.77140641	0.05094064	54.40462846	0	0	Up
Zfp958	475.1457959	489.960525	426.3056396	457.0229894	486.2679868	471.7331848	23868.97134	24288.03226	6370.429965	5.651991645	0.07455248	75.81225425	0	0	Up
Rnf169	5596.809375	5891.918298	5907.952685	5598.000198	5784.303168	5861.16324	42254.44145	48901.26292	15724.48142	2.968566279	0.06369736	46.60422671	0	0	Up
Abi1	8252.378823	8085.301893	8688.993721	8212.596835	8398.513114	8432.838582	39950.14924	41360.94834	16422.71507	2.272304833	0.04712638	48.21725459	0	0	Up
Usp47	4539.63427	4980.62985	4936.699742	4739.222116	4698.512471	5078.183418	23698.87473	24504.23559	9646.999024	2.301519008	0.06121751	37.59576181	0	0	Up
Zc3h4	2127.953564	2296.332499	2267.262305	2219.218609	2199.635316	2111.613906	29600.56928	29290.19119	9014.097084	3.772108867	0.05768214	65.39474689	0	0	Up
Max	1741.229583	1694.843995	1588.59177	1648.471294	1573.097718	1588.330496	24824.70754	24348.78361	7376.007001	3.95914896	0.0590594	67.03672972	0	0	Up
Ptk2b	6294.46721	6697.398149	6625.835295	6720.363633	6552.149412	6513.808533	35371.63716	36186.36279	13870.25277	2.453328115	0.0485343	50.5483412	0	0	Up
Eif4a1	8483.635877	8744.937464	8559.292241	8782.281306	8710.223362	8518.431407	41725.35641	40084.27659	16701.05433	2.247501864	0.0460396	48.81670948	0	0	Up
H2afj	1023.166714	967.5290523	897.8559816	984.1936935	889.4132408	908.4511229	23295.71757	20724.25088	6211.322281	4.613600365	0.07768227	59.39064761	0	0	Up
Hnrnpc	5935.921609	5992.00751	6077.871678	5824.386005	5494.412637	5806.695079	59155.09031	62299.61574	19573.25007	3.425706099	0.0490146	69.89155011	0	0	Up
Ube2s	1261.225446	1182.005936	1278.916919	1356.18915	1326.846622	1200.244845	19473.71251	19114.34011	5774.185192	3.933287223	0.07032031	55.93387179	0	0	Up
Rrp1	3287.153839	3023.647442	3199.303173	3292.691212	3020.472303	3037.572652	18451.25333	16908.53007	6777.578003	2.545123075	0.06603986	38.53919542	0	0	Up
Blnk	4355.017295	4395.346266	4287.186904	4330.027114	4326.538242	4620.067274	44471.33595	45084.64872	14483.77097	3.323100517	0.04878126	68.12248694	0	0	Up
Naca	1795.643008	1773.962134	1840.956665	1848.285997	1777.787447	1842.191035	32772.26023	31926.62109	9447.21345	4.159403769	0.05516269	75.40248183	0	0	Up
Wdr61	2162.933623	2230.559588	2245.142673	2108.682816	2285.875152	2079.516596	18632.62706	18204.85667	6243.774272	3.077432397	0.06594653	46.66556711	0	0	Up
Ext1	8017.235095	9326.408126	8997.663134	8850.303332	8724.76984	8790.772215	60250.8508	71688.37948	23080.79775	2.913175852	0.07232029	40.2815844	0	0	Up
Rapgef2	1981.231652	2269.642043	2105.386815	2297.869077	2162.230087	2185.534982	18985.97693	20005.95551	6499.228386	3.164805946	0.07255726	43.61804777	0	0	Up
Morf4l1	5923.289921	5929.094291	6324.203946	5962.555746	5807.161919	5814.476245	37340.43495	38684.31534	13973.19154	2.709665965	0.048892	55.42145946	0	0	Up
Morf4l1b	5923.289921	5929.094291	6324.203946	5962.555746	5807.161919	5814.476245	37340.43495	38684.31534	13973.19154	2.709665965	0.048892	55.42145946	0	0	Up
Rpl17	1260.253778	1262.077306	1421.689091	1249.904734	1497.248224	1268.330047	33827.61102	31551.39216	9167.313295	4.564082512	0.07577584	60.23137052	0	0	Up
Krr1	910.4531917	928.4465979	865.681971	889.6005631	818.7589179	849.1197326	32486.5731	30570.43654	8539.883828	5.240155626	0.06562669	79.84793081	0	0	Up
Rps15	429.477386	387.9648515	488.642785	447.457392	434.3162788	399.7573999	25711.84125	22262.68947	6320.268351	5.846839538	0.09074756	64.4297142	0	0	Up
Ptcd3	5561.829316	5271.365181	5739.03913	5567.177718	5423.758314	5472.104944	29254.7375	32275.04795	11820.63251	2.497516169	0.05785655	43.16739218	0	0	Up
Siva1	912.3965282	922.7272144	921.9864895	905.5432255	920.5842656	926.931392	42146.36901	38604.80255	10782.66758	5.449730512	0.06484092	84.04770417	0	0	Up

Fbxl12	2531.195906	2437.410627	2450.25199	2696.435638	2479.135505	2379.091485	24695.02062	23824.35652	7936.612287	3.320242893	0.06217894	53.3981921	0	0	Up
Prkag1	2908.203205	2928.324383	3081.666947	3108.819172	3078.658216	3170.825118	15459.99636	14624.99404	6045.18593	2.267076734	0.06034152	37.57075931	0	0	Up
Blcap	1515.802539	1517.543105	1285.954984	1493.296047	1406.852252	1371.430496	60342.00755	57367.14219	15787.50364	5.405080737	0.06171655	87.57910785	0	0	Up
Shroom4	4222.870407	4707.05267	4637.079269	4489.453738	4252.766816	4600.614359	146222.9404	155853.1207	41123.2373	5.092276612	0.05540002	91.91831377	0	0	Up
Psrc1	952.2349283	866.4866094	893.8342303	954.434057	932.0136414	944.4390153	27207.93961	25166.24662	7239.70359	4.802635668	0.07117351	67.47785617	0	0	Up
Scyl2	1525.519222	1643.369543	1636.852786	1531.558437	1709.211193	1640.853367	16412.91328	16839.7381	5367.501991	3.311454857	0.06813517	48.60125494	0	0	Up
Marcks	108.8268489	89.60367578	103.5600964	95.65597453	116.3718259	75.86636786	39629.69099	36809.95752	9628.691662	8.639752339	0.19538761	44.21852821	0	0	Up
Fat1	5957.298312	6281.78961	6272.926617	6136.862188	6116.794099	6100.434093	132392.4884	139990.7646	38656.16974	4.478666782	0.04493308	99.6741542	0	0	Up
Lsmem1	148.665249	146.7975114	165.8972418	166.8665333	172.4796705	149.7874442	40967.90962	40243.30218	10270.21318	7.980044536	0.09627105	82.89142201	0	0	Up
Hpcal1	4376.393997	4828.112955	4365.611055	4547.910167	4829.430775	4416.784314	34871.68469	35347.45812	12197.92326	2.925187499	0.05991729	48.82042717	0	0	Up
Cep170b	11695.97126	11023.15858	11544.43716	11717.85688	11859.5359	11096.91527	59199.25905	54817.3723	22869.3133	2.31235731	0.05245309	44.08428894	0	0	Up
Zfp568	1285.517153	1247.778847	1328.183373	1233.962071	1246.840992	1444.378927	17639.30045	18599.74044	5503.212781	3.750403772	0.07635447	49.11832596	0	0	Up
Chd2	6355.682312	7058.672544	6952.60259	6901.04714	6529.29066	7118.794185	47854.47295	53153.8574	17740.55247	2.887569812	0.0632033	45.687011	0	0	Up
Rab7	4777.693003	5000.647693	4578.763875	4781.735882	4862.679868	4810.705839	82404.75963	85314.54987	24566.44196	4.115939626	0.04585487	89.76014343	0	0	Up
Maea	6269.203834	6097.816106	6517.24801	6399.384696	6380.708775	6537.152031	83072.92919	81541.71237	25352.01938	3.671551308	0.04204855	87.31695337	0	0	Up
Ulbp1	2339.777252	2364.965102	2320.55051	2243.664025	2378.349192	2504.562785	27592.30157	26674.30954	8552.309997	3.473941907	0.05654773	61.43379823	0	0	Up
Snhg15	1001.790011	960.8564381	1090.900045	1004.387733	1014.09734	1024.195966	18453.13285	17982.39952	5316.469988	4.159816885	0.06976842	59.62320274	0	0	Up
Gm15638	2259.128784	2250.577431	2168.729398	2225.595674	2248.469922	2177.753816	35797.34857	35235.78284	10545.4233	4.004561834	0.05249865	76.27933219	0	0	Up
Trp53cor1	1378.797309	1423.173276	1365.384572	1239.276292	1463.999131	1387.965474	22281.71623	20821.63172	6420.243	3.918084688	0.06930592	56.53319146	0	0	Up
Plcxd2	411.9873567	465.1765296	447.419834	361.3670149	503.9315675	440.6085211	98968.03455	105727.0034	25915.69109	7.760407363	0.12839724	60.44061143	0	0	Up
Rpl41	11661.96287	11527.41757	12189.92824	11773.12478	10610.61684	10721.47401	53105.85339	47094.80367	21085.64767	2.231750283	0.05471389	40.78946186	0	0	Up
9230114K1	1609.082695	1493.71234	1561.444949	1669.728178	1557.512206	1755.625564	16159.17801	15768.54886	5196.8541	3.267938101	0.07425451	44.0099599	0	0	Up
Vamp9	160.3252685	170.6282762	180.9788093	144.546806	147.5428507	161.4591931	27100.80694	27939.36706	7000.7069	7.475049846	0.10233256	73.04664507	0	0	Up
Gm4881	37.89506347	54.33414382	38.20663751	28.69679236	42.60040055	51.55022432	32873.75434	32795.00803	8240.255703	9.441806001	0.1593375	59.25664604	0	0	Up
Hspa14	2543.827594	2458.3817	2438.186736	2393.525052	2379.388226	2228.331395	26094.32368	24744.56079	8160.065646	3.464139898	0.05979547	57.93315129	0	0	Up
Dicer1	2630.306072	2901.633926	2865.497814	2776.14895	3237.630442	2986.995073	17963.51774	17298.05343	6582.472932	2.502290935	0.06692912	37.38717985	6.31E-306	9.56E-305	Up
Ap3d1	3773.959655	3980.690958	4182.62137	3830.490358	4031.45254	4047.178932	19720.86946	19018.74607	7823.251169	2.261595589	0.0605649	37.34168778	3.46E-305	5.22E-304	Up
Arhgap11a	2557.43095	2834.907785	2616.149232	2496.620935	2664.083586	2500.672202	15091.61033	15080.62917	5730.263023	2.546693225	0.06831268	37.27995196	3.47E-304	5.21E-303	Up
Cltc	3064.6418	3527.906426	3353.135161	3239.549004	3273.996638	3327.421083	18975.63956	20140.85924	7362.893614	2.566904383	0.0688925	37.25956109	7.42E-304	1.11E-302	Up
Cdc27	5006.035052	5224.656882	5115.667676	5069.76665	5636.760317	5195.873553	22575.86119	22500.33445	9540.619472	2.057211924	0.05548124	37.07941648	6.03E-301	8.92E-300	Up
Wdr35	3182.213663	3336.307077	3250.580502	3132.201744	2995.535483	3381.889244	18397.68699	20042.585	7214.874963	2.591240189	0.06994701	37.04576232	2.10E-300	3.11E-299	Up
Ell2	4105.298543	3957.813424	4065.990582	3925.083488	4105.223965	4265.051578	17457.92671	17588.40915	7433.84968	2.065784324	0.05581416	37.01183099	7.39E-300	1.09E-298	Up
Kat6a	2753.707946	3004.58283	2903.704451	3166.212757	3052.682362	3180.551576	16698.60041	17172.97712	6491.627432	2.4418968	0.06650369	36.71821375	3.74E-295	5.38E-294	Up
Elf1	3370.717313	3546.971038	3476.804014	3520.139863	3627.268252	3581.281622	15438.38187	15501.4216	6507.873197	2.101736279	0.05730932	36.67355246	1.93E-294	2.76E-293	Up
Ercc3	2815.894717	2767.228413	2669.437437	2592.27691	2486.408744	2631.006732	13893.41597	13002.57565	5357.280571	2.393625821	0.0658771	36.33471764	4.58E-289	6.43E-288	Up
Rprd2	2286.335496	2395.468481	2528.676141	2335.068623	2421.988627	2589.182965	13854.8858	13637.78461	5256.173843	2.455549689	0.06782918	36.20196421	5.67E-287	7.89E-286	Up
St7	2929.579907	3069.402511	3200.308611	2901.564561	3072.424011	3026.873549	17198.55287	19343.05107	6842.719637	2.58291652	0.07148947	36.13003014	7.66E-286	1.06E-284	Up
Rad50	3080.188493	2924.51146	2898.677262	2995.094847	2995.535483	3111.493728	14005.24745	14115.75479	5765.812938	2.202957097	0.0612609	35.96024765	3.50E-283	4.79E-282	Up
Fbrsl1	5041.01511	5265.645798	4976.917255	5181.365287	5189.975628	5464.323778	25459.04573	23042.62959	9952.614772	2.18643472	0.06104061	35.81934784	5.52E-281	7.49E-280	Up
Ppm1d	739.4395719	965.6225911	743.0185559	927.8629529	818.7589179	758.6636786	58647.61976	58128.32086	15216.16336	6.210182971	0.17355133	35.78297612	2.03E-280	2.75E-279	Up
Tm9sf4	2319.372218	2313.49065	2621.176421	2466.861299	2550.828862	2328.513906	13026.01723	13602.04852	5153.538639	2.448579262	0.06891721	35.52928576	1.74E-276	2.31E-275	Up
Atxn7l3b	3504.807537	3251.469554	3350.118847	3320.32516	3236.591408	3160.126015	15383.87578	14591.04476	6224.794882	2.228407353	0.06285613	35.45250698	2.65E-275	3.50E-274	Up
Hsf2	2866.421468	2710.987808	2605.089416	2833.542534	2734.737909	2743.833638	13564.49987	13561.84542	5452.619759	2.307809688	0.0653517	35.31369245	3.62E-273	4.75E-272	Up
Pmepa1	5925.233258	6545.834485	6083.904305	6247.397981	6101.208586	6062.500909	26323.62519	24682.02263	10996.46592	2.06806172	0.05858972	35.2973491	6.45E-273	8.45E-272	Up
Pvt1	10817.58312	11814.33997	11545.44259	11121.60131	11769.13993	11554.05877	53826.64953	63953.30322	23300.2648	2.336297249	0.06656271	35.09919117	6.94E-270	8.99E-269	Up
Tnpo2	4850.568125	4949.173241	4881.400661	5199.433638	5385.31405	5005.234988	21313.76314	20733.1849	9039.759093	2.016916996	0.057568	35.03538146	6.51E-269	8.42E-268	Up
Ppp2r2b	2404.879028	2587.06783	2577.942594	2802.720054	2636.029663	2604.745297	13509.99378	14201.5214	5415.612456	2.402707296	0.06858216	35.0340006	6.83E-269	8.83E-268	Up
Nsmce2	7845.249807	8402.727681	7925.866408	7707.745859	7804.185574	8049.61616	39480.2691	47863.12956	16884.84877	2.461863006	0.07065626	34.84281498	5.47E-266	6.98E-265	Up
Spin1	2903.344863	3231.451712	3039.438558	3182.155419	3335.299653	2947.116598	17599.83051	17697.40422	6742.005192	2.490529098	0.07156843	34.79926921	2.49E-265	3.18E-264	Up
Arhgap23	3803.109703	4422.989953	4260.040083	4020.739463	4341.08472	4231.981623	21752.63119	21824.02899	8582.075715	2.345735942	0.06743732	34.78394348	4.25E-265	5.41E-264	Up

Ppl	3485.374171	3811.969143	3676.886142	3904.889449	3782.084342	3827.360994	17236.14329	16825.44367	7068.768899	2.162230827	0.06220672	34.75879992	1.02E-264	1.29E-263	Up
Pigf	3085.046834	2918.792077	2979.112288	3389.410031	3086.970489	3051.189692	16771.90171	15933.82827	6402.031424	2.413678234	0.06957174	34.69336968	9.92E-264	1.25E-262	Up
Dlgap4	3377.518991	3485.011049	3261.640318	3386.221498	3496.349948	3394.533639	14790.88704	14190.80057	6172.870382	2.072462552	0.05998454	34.54994357	1.43E-261	1.78E-260	Up
Nsun2	3777.846328	3809.109451	3938.299977	3930.397709	3862.089972	3839.032743	16012.5754	15020.77122	6773.76535	2.010655022	0.05821548	34.53815095	2.15E-261	2.68E-260	Up
Btg1	163.2402734	124.8732077	97.52746944	119.0385461	135.0744408	88.51076251	44010.85341	38135.76639	10359.36056	8.523824273	0.24982667	34.11895184	3.86E-255	4.68E-254	Up
Rbpj	2144.471925	2509.856152	2206.936035	2129.9397	2164.308155	2445.231395	15204.38156	16062.47819	5608.450389	2.761580361	0.08095639	34.11195077	4.91E-255	5.94E-254	Up
Kctd10	3031.605078	3087.513892	3024.356991	3182.155419	3028.784576	3132.891935	15188.40564	13908.48548	5948.024876	2.239300099	0.06590839	33.97594763	5.05E-253	6.05E-252	Up
Afg3l2	2334.918911	2640.448744	2516.610887	2396.713584	2470.823232	2403.407628	13041.99316	12816.74799	5077.708017	2.407499425	0.07138684	33.72469627	2.51E-249	2.96E-248	Up
Dennd5a	3076.301819	3240.984017	2933.867586	3047.174211	3187.756802	3234.047092	14335.1033	15743.5336	6099.846054	2.227693061	0.06630957	33.59534667	1.96E-247	2.29E-246	Up
Copa	3215.250385	3243.843709	3088.705012	3188.532484	3429.851762	3237.937675	14691.27245	13864.70877	5995.012781	2.098680752	0.06258353	33.53407503	1.54E-246	1.79E-245	Up
Odc1	3673.87782	3505.028892	3624.603375	3564.779317	3596.097227	3763.166375	14986.35718	14465.96845	6397.48483	2.000561017	0.05974084	33.48732426	7.37E-246	8.56E-245	Up
Psmd1	2814.923048	2953.108378	2948.949153	3049.299899	2760.713763	2821.645297	11891.72658	12281.60007	5190.245773	2.114403703	0.06333123	33.38643216	2.16E-244	2.50E-243	Up
Ppp2r2d	3833.231421	3850.098367	3608.516369	3988.854138	3819.489572	3993.683416	16462.72058	16348.36689	6988.120094	2.070058717	0.06203237	33.37062344	3.66E-244	4.23E-243	Up
Fmnl2	3625.294406	4157.991848	3925.229286	3622.172902	3790.396615	3841.95068	19333.68823	21114.66764	7926.423951	2.405880294	0.07215483	33.34330261	9.11E-244	1.05E-242	Up
Kdm5a	5964.09999	6051.107807	5813.441529	5938.11033	6055.491083	6098.488801	23306.99469	26460.78642	10711.06508	2.033817528	0.06106909	33.30354944	3.43E-243	3.94E-242	Up
Trim8	3107.395205	2956.921301	3075.63432	2996.157691	2900.983374	3033.682069	14088.88611	13053.49957	5651.644955	2.193099411	0.06606769	33.19473217	1.28E-241	1.47E-240	Up
Rapgef6	4725.222915	5203.685809	5030.20546	4885.89461	4843.977253	5040.250234	23419.76593	27371.16326	10065.02068	2.361338305	0.07167351	32.94576024	4.87E-238	5.48E-237	Up
Brd4	4835.9931	5350.483321	5157.896064	5036.818481	5240.888302	5548.943957	23318.27181	26096.27832	10073.19667	2.195185701	0.06665504	32.93352785	7.28E-238	8.19E-237	Up
Vapa	3178.32699	3374.436301	3099.764828	3201.286614	3482.842504	3346.873998	13773.12666	13754.8203	5901.434774	2.011144314	0.06121514	32.85370714	1.01E-236	1.13E-235	Up
Ymel11	2754.679614	2756.742876	2808.187857	2616.722325	2881.241725	2783.712113	12463.10083	13399.24622	5307.954195	2.190888674	0.0673035	32.55237184	1.94E-232	2.14E-231	Up
Rela	2920.834892	2757.696107	2721.720204	2829.291158	2700.449781	2832.3444	11890.78682	11925.13259	5072.281994	2.105657645	0.06486505	32.46213025	3.65E-231	4.01E-230	Up
Cstf3	3253.145449	3673.750707	3432.564749	3328.827914	3302.05056	3158.180724	14970.38125	14624.99404	6217.986925	2.195843062	0.06775267	32.40969274	2.00E-230	2.20E-229	Up
Notch1	2844.073097	2678.577967	2792.100852	2909.00447	2839.680359	2904.320185	13083.34261	12295.0011	5293.26258	2.143344775	0.06616617	32.39336258	3.40E-230	3.73E-229	Up
Sp1	2601.156024	2592.787214	2783.051912	2687.932884	2547.71176	2873.195521	13186.71624	14306.04946	5447.325127	2.342092618	0.07262335	32.24985558	3.53E-228	3.84E-227	Up
Gnl3	3031.605078	2894.961312	2928.840397	3095.002198	3094.243728	3155.262787	13287.27059	12665.76302	5519.118638	2.053983797	0.06392658	32.13035572	1.66E-226	1.79E-225	Up
Ubb	178.7869661	204.9445776	164.891804	258.2711312	194.2993879	196.4744399	23048.56061	22239.46101	5810.711242	6.856594552	0.21344713	32.12315174	2.09E-226	2.26E-225	Up
Cxxc4	3188.043673	3018.881289	3106.802893	3245.926069	3277.11374	3025.900903	13382.18638	13345.64209	5698.81213	2.084507774	0.06502737	32.05585483	1.82E-225	1.95E-224	Up
Sdc1	2809.093039	2699.54904	2626.20361	2720.881053	2631.873527	2773.985656	13051.39076	11976.94992	5161.240826	2.210722642	0.0690858	31.99966833	1.10E-224	1.18E-223	Up
9530053A0	157.4102637	156.3298173	211.1419442	177.494975	179.7529096	109.9089688	29460.545	27873.2553	7290.729897	7.631455029	0.23911578	31.91531297	1.64E-223	1.74E-222	Up
Ubp1	2732.331243	2957.874531	2898.677262	2809.097119	2746.167284	2920.855163	13065.48717	14454.35422	5573.105499	2.279664553	0.07217721	31.58427001	6.07E-219	6.34E-218	Up
Phb2	2748.849604	2755.789645	2775.008409	2850.548041	2806.431266	2615.4444	12426.45018	11511.48737	5061.251114	2.142643891	0.06841077	31.32027018	2.47E-215	2.54E-214	Up
Atg2b	2878.081487	3127.549577	3060.552752	2929.198509	3069.306908	3001.584759	12285.48613	12638.06755	5373.728459	2.037629216	0.06558084	31.07049664	6.03E-212	6.10E-211	Up
Tubb6	212.7953564	322.1919406	316.7129162	263.585352	252.4853008	248.9973099	27090.46958	24921.45442	6703.586522	6.696550604	0.21596559	31.00748818	4.27E-211	4.31E-210	Up
Cmbl	2741.076258	2582.301677	2495.496692	2670.927378	2747.206319	2403.407628	12813.63141	12561.23496	5126.91029	2.301009534	0.07481087	30.75768807	9.65E-208	9.62E-207	Up
Tet3	3070.47181	3728.084851	3383.298296	3402.164161	3519.208699	3515.141711	17399.66157	18304.02431	7040.256927	2.343604364	0.0762041	30.7543076	1.07E-207	1.07E-206	Up
Slc1a4	567.4542838	690.1389496	830.491647	623.8895228	615.1082226	577.7515707	22867.18688	22097.41006	6108.678892	5.23646075	0.1707356	30.66999946	1.43E-206	1.41E-205	Up
Trp53	3597.116025	3347.745844	3127.917087	3431.923797	3514.013529	3260.308527	15176.18875	14331.95812	6223.396461	2.123149779	0.07006874	30.30095532	1.11E-201	1.08E-200	Up
Spty2d1	144.7785758	190.6461187	165.8972418	114.7871694	112.2156893	148.8147985	24563.45419	23198.97498	6079.946094	7.513977605	0.24836635	30.25360625	4.68E-201	4.50E-200	Up
Cemip2	2861.563126	3226.685559	2960.008969	2852.673729	3029.82361	3025.900903	13045.7522	14080.0187	5635.30335	2.163336957	0.07158085	30.22228484	1.21E-200	1.16E-199	Up
Epc2	2716.784551	2977.892374	3019.329801	2839.919599	2870.851384	2932.526912	12144.52209	13093.70267	5324.441173	2.120620557	0.07086298	29.92564713	9.13E-197	8.58E-196	Up
Cacul1	6598.599386	6726.948297	6930.482957	6703.358126	6973.997281	6577.030506	29634.40065	37340.63843	13435.68195	2.305330046	0.07826066	29.45707298	1.02E-190	9.34E-190	Up
Pdcd6	3048.123439	2918.792077	2868.514127	3067.36825	2835.524222	2977.268616	12540.16117	11552.58387	5226.041972	2.051081947	0.06987071	29.35539236	2.04E-189	1.85E-188	Up
Scaf1	3227.882073	3203.808024	3274.71101	3359.650394	3215.810725	2960.733638	14211.99471	12571.06238	5753.206619	2.116625971	0.07258169	29.16198206	5.89E-187	5.30E-186	Up
Camsap1	2876.138151	3165.678801	3244.547875	3143.89303	3273.996638	3366.326913	13739.29529	15712.26452	6065.267652	2.149006422	0.07475531	28.74720912	9.82E-182	8.56E-181	Up
Tm9sf3	2803.263029	3077.981586	3045.471185	2931.324197	2611.092844	3038.545298	13085.22213	12936.46388	5441.170519	2.203048728	0.0768781	28.65638864	1.33E-180	1.16E-179	Up
Mon2	3124.885234	3621.323024	3443.624565	3385.158654	3584.667851	3449.001801	14133.05484	14593.72497	6166.930118	2.030166898	0.07092292	28.62497657	3.29E-180	2.84E-179	Up
Ireb2	3334.765586	3646.10702	3870.935643	3445.740771	3789.357581	3819.579828	14684.69413	15798.03114	6548.651461	2.00223951	0.07144164	28.02622385	7.79E-173	6.47E-172	Up
Nufip1	832.7197281	583.3771231	738.9968045	732.2996272	682.645443	745.0466383	22722.4638	22120.63852	6144.77346	4.972942997	0.177712	27.98315892	2.60E-172	2.16E-171	Up
Rnf111	2628.362736	2849.206244	2861.476062	2944.078327	2888.514964	3179.57893	11688.73836	12593.39744	5204.169132	2.000340562	0.07298581	27.40725289	2.25E-165	1.81E-164	Up
Scaf8	2885.854834	3453.55444	3250.580502	3334.142134	3192.951973	3077.451127	12604.06487	13044.56555	5605.395679	2.032361317	0.07752248	26.21641325	1.73E-151	1.29E-150	Up

Akr1d1	12.63168782	9.532305934	19.10331876	10.62844161	3.117102479	13.61704039	36272.86727	36552.65769	9111.769357	12.07191066	0.59164383	20.40401694	1.54E-92	8.07E-92	Up
P2rx7	35.95172689	14.2984589	36.19576186	24.44541571	20.7806832	42.79641264	34339.78037	33798.2987	8539.068441	10.06229546	0.53059175	18.9642894	3.37E-80	1.61E-79	Up
Gpr132	20.40503418	30.50337899	21.11419442	31.88532484	7.273239119	27.23408077	22472.48756	21800.80053	5551.462918	10.31728451	0.59348795	17.38415168	1.09E-67	4.72E-67	Up
Cpm	69.96011718	27.64368721	41.222951	41.4509223	20.7806832	57.38609877	51074.09167	51337.57073	12833.76336	10.35258902	0.61437075	16.85071932	1.04E-63	4.35E-63	Up

Supplementary Table 4: KHSRP-regulated genes with intron retention in HL7702 cells transfected with shKHSRP as determined by RNA-Seq analysis

SYMBOL	shNC 1	shNC 2	shKHSRP 1	shKHSRP 2	baseMean	log2Fold-Change	lfcSE	stat	pvalue	padj	type
NFYA	595.0663865	494.3370937	23386.40233	18448.93005	10731.18396	5.263493356	0.185419734	28.38691032	2.93E-177	3.99E-174	Up
AK2	12154.98874	10625.59793	25404.99015	22705.69891	17722.81893	1.078613553	0.163068508	6.614481031	3.73E-11	2.50E-10	Up
KDM1A	3093.387999	2981.163055	14646.93521	11014.24284	7933.932276	2.078929471	0.181983957	11.42369641	3.18E-30	6.08E-29	Up
CREBBP	2887.587559	3227.95309	13932.01869	10860.33074	7726.97252	2.019476364	0.179860587	11.22800943	2.97E-29	5.40E-28	Up
KMT2E	3659.738045	3739.70175	29856.06483	22789.25119	15011.18895	2.830899804	0.177096193	15.9850969	1.62E-57	8.57E-56	Up
ZNF263	1750.10141	1773.708745	9054.871467	8079.652188	5164.583453	2.281824265	0.163970018	13.91610669	5.06E-44	1.68E-42	Up
KIAA0100	3391.718868	3650.372842	7857.44163	7314.48919	5553.505633	1.107402114	0.161662334	6.850093573	7.38E-12	5.21E-11	Up
SEC62	3800.129042	3848.713299	9109.098881	7755.703869	6128.411273	1.140884267	0.166395851	6.856446594	7.06E-12	4.99E-11	Up
CSDE1	20101.43826	19465.37472	46219.46369	39106.86515	31223.28546	1.108742662	0.161340653	6.872060071	6.33E-12	4.49E-11	Up
UBE3C	7226.94566	5000.90481	15738.12358	10938.01971	9725.998439	1.125568628	0.219305886	5.132414136	2.86E-07	1.33E-06	Up
REV3L	4246.029994	3308.197702	9597.145608	7433.22138	6146.148671	1.1730204	0.19414932	6.041846547	1.52E-09	8.91E-09	Up
VTA1	1873.741209	1653.341827	12125.91379	9340.265555	6248.315596	2.605775693	0.184031442	14.15940487	1.63E-45	5.72E-44	Up
BAZ1B	2739.219801	2724.5317	13530.29315	9952.982285	7236.756734	2.103875924	0.185569676	11.33739075	8.57E-30	1.59E-28	Up
ZNF207	10325.91739	9504.444429	21808.27391	18684.9286	15080.89108	1.030055262	0.163547734	6.298193439	3.01E-10	1.88E-09	Up
AKAP8L	3165.17885	2589.781313	11947.73801	9438.476132	6785.293575	1.894035908	0.186112436	10.17683688	2.52E-24	3.68E-23	Up
MBTD1	2594.840423	2236.250804	9892.62968	7494.786219	5554.626781	1.847917528	0.188355696	9.810786568	1.01E-22	1.36E-21	Up
ELOVL5	3936.531659	3983.463686	20105.09044	16890.75329	11228.95977	2.223882158	0.164810572	13.49356498	1.71E-41	5.09E-40	Up
CLK1	6334.34608	5129.599	16759.59099	14187.7637	10602.82494	1.432840135	0.177522766	8.071303578	6.96E-16	6.45E-15	Up
GPRC5A	5379.527763	7688.342303	16901.24628	17471.22178	11860.08453	1.395170264	0.191181005	7.297640619	2.93E-13	2.27E-12	Up
TACC3	5251.101907	4723.83379	13917.63182	12946.20612	9209.693409	1.429367649	0.160496135	8.905931878	5.29E-19	5.92E-18	Up
MDH1	6960.521835	7083.479611	15879.77887	14609.9226	11133.42573	1.118425528	0.156067121	7.166311033	7.70E-13	5.82E-12	Up
STRAP	7124.04544	7400.672938	30481.34011	24765.18935	17442.81196	1.92743997	0.167822098	11.48501895	1.57E-30	3.07E-29	Up
HSF2	2223.12335	1352.803042	20230.14549	16581.46327	10096.88379	3.363999147	0.221754752	15.16990783	5.60E-52	2.50E-50	Up
IFNGR1	1616.889498	1041.665913	10961.68441	9555.742491	5793.995579	2.948436806	0.211443487	13.94432547	3.41E-44	1.15E-42	Up
BRD9	8370.015542	10488.57647	20826.64705	19451.55743	14784.19912	1.094783953	0.171583449	6.380475261	1.77E-10	1.12E-09	Up
BCLAF1	19763.22358	18080.77664	44394.54521	37984.03976	30055.6463	1.122248911	0.162535038	6.904658352	5.03E-12	3.59E-11	Up
SLC39A9	7147.975724	6828.362306	16833.73868	14982.24329	11448.08	1.186851189	0.159178002	7.456125687	8.91E-14	7.20E-13	Up
NUP160	7918.530857	8415.842988	24590.47226	18810.98994	14933.95901	1.409930808	0.177289811	7.952689434	1.83E-15	1.64E-14	Up
GRN	3469.891128	4059.166151	7508.836825	8103.10546	5785.249891	1.051971201	0.168226209	6.253313378	4.02E-10	2.48E-09	Up
ZCCHC8	3548.063388	3283.215889	13210.46208	9996.95717	7509.674631	1.764573288	0.182436208	9.672275605	3.95E-22	5.17E-21	Up
PIAS1	3549.65874	2861.553161	13183.90171	10606.74224	7550.463963	1.89192911	0.18493572	10.23019841	1.45E-24	2.15E-23	Up
RFC1	6221.873747	5424.081588	13063.27338	10515.86081	8806.272382	1.017863607	0.176528342	5.766006734	8.12E-09	4.44E-08	Up
CUL3	3454.735282	3137.867157	12164.64766	9555.742491	7078.248148	1.720346192	0.17835422	9.645671372	5.13E-22	6.67E-21	Up

NSUN2	10353.83606	12847.46526	46585.77541	36119.50464	26476.64534	1.833805504	0.185397343	9.891217811	4.54E-23	6.24E-22	Up
LSG1	1198.109534	1437.589802	13627.68116	10350.22208	6653.400644	3.185505461	0.190181702	16.74979992	5.68E-63	3.59E-61	Up
HSPA5	535.2406774	527.6461782	22986.89015	24835.54917	12221.33154	5.491622034	0.160592486	34.1960087	2.77E-256	2.83E-252	Up
POLR2B	4340.953453	3143.923354	18041.12865	13910.72193	9859.181847	2.094021229	0.201367842	10.39898532	2.51E-25	3.84E-24	Up
SCML1	4268.364925	3323.338195	9903.696499	8806.703618	6575.52581	1.301509398	0.181006797	7.190389645	6.46E-13	4.90E-12	Up
NOP16	2044.443899	2379.328462	12254.2889	10363.41454	6760.368951	2.35416774	0.174067863	13.52442493	1.12E-41	3.37E-40	Up
RSF1	3596.721631	3705.635641	10049.77851	8945.957421	6574.523302	1.379358364	0.161865731	8.521620715	1.57E-17	1.61E-16	Up
CLPTM1L	9852.097775	13156.33132	26327.96287	21083.02566	17604.85441	1.043107196	0.19123959	5.454452163	4.91E-08	2.49E-07	Up
LIMA1	3189.109133	3468.686927	8911.002817	7329.147485	5724.486591	1.286639218	0.17313353	7.431484904	1.07E-13	8.63E-13	Up
MSMO1	2658.654513	2281.672283	57619.39415	53146.58008	28926.57526	4.486850206	0.162469896	27.61650203	7.05E-168	5.99E-165	Up
MRTO4	468.2358832	364.1288547	11124.36666	8733.412144	5172.535885	4.576975511	0.196610783	23.2793718	7.17E-120	2.15E-117	Up
THRAP3	6205.122548	5592.141059	16957.68706	13699.64248	10613.64829	1.377922853	0.173058929	7.962159837	1.69E-15	1.52E-14	Up
ARID4B	6024.050069	5095.532891	20980.47583	15388.27806	11872.08421	1.709748293	0.190351713	8.982048378	2.66E-19	3.02E-18	Up
NOP58	8607.723026	6510.411954	59397.83199	43705.17229	29555.28481	2.769802992	0.198932869	13.92330492	4.57E-44	1.53E-42	Up
TAB2	3002.452921	2707.877158	8301.22108	7110.73889	5280.572512	1.432628866	0.17048068	8.403467575	4.33E-17	4.31E-16	Up
USP36	3481.85627	3226.439041	19217.53154	14444.28386	10092.52768	2.327253375	0.182133819	12.77771137	2.18E-37	5.56E-36	Up
PUM2	4522.823608	3981.949637	20102.87707	15441.04792	11012.17456	2.063407681	0.181252888	11.38413683	5.02E-30	9.47E-29	Up
ZFR	3816.082565	4000.875253	11353.44981	8649.859862	6955.066874	1.355771947	0.180738029	7.501309805	6.32E-14	5.16E-13	Up
ATG5	3259.304632	3337.721664	7300.780624	6115.440662	5003.311895	1.024329307	0.169975306	6.026341891	1.68E-09	9.75E-09	Up
PPP1R12A	3717.968402	3701.850518	9970.097415	8347.898986	6434.45383	1.303992514	0.167940696	7.764601113	8.19E-15	7.07E-14	Up
YBX3	11511.26411	9723.981576	24435.53679	22644.13407	17078.72914	1.148693762	0.164368718	6.9885181	2.78E-12	2.02E-11	Up
GPBP1	5400.267342	4519.437136	20618.59085	15455.70622	11498.50039	1.862754105	0.188285304	9.893252779	4.45E-23	6.11E-22	Up
WAPL	2330.809627	2353.589624	9855.002495	7461.07214	5500.118471	1.886430981	0.182948051	10.31129314	6.27E-25	9.42E-24	Up
U2AF2	2016.525235	1882.720294	13107.54066	9759.492792	6691.569744	2.552235336	0.185656967	13.74704854	5.30E-43	1.67E-41	Up
RNF4	3844.001229	4295.357841	18635.41685	14634.8417	10352.4044	2.031344087	0.177390748	11.45124034	2.32E-30	4.49E-29	Up
HMG20B	4112.818082	4960.02548	15274.42386	14438.42055	9696.421991	1.711452053	0.167731701	10.20350977	1.91E-24	2.82E-23	Up
IPO5	3796.938338	3192.372932	8239.246892	6212.185409	5360.185893	1.048319266	0.190926873	5.49068474	4.00E-08	2.04E-07	Up
WDR3	1786.794512	1582.18151	22206.6794	16742.70452	10579.58998	3.531397931	0.184469134	19.14357084	1.10E-81	1.16E-79	Up
ZC3H15	3531.312189	2779.7945	25609.7263	22003.56658	13481.09989	2.915524829	0.179048288	16.28345554	1.29E-59	7.31E-58	Up
YBX1	24072.26999	23628.25325	66075.5507	58112.81041	42972.22109	1.380476827	0.156528159	8.819351344	1.15E-18	1.26E-17	Up
ELAVL1	8681.906905	8185.707496	19101.32993	18054.62192	13505.89156	1.139380406	0.155041302	7.348883135	2.00E-13	1.57E-12	Up
ZBTB11	1615.294146	1358.859239	21555.95043	15757.66709	10071.94273	3.649367877	0.192728265	18.93530187	5.84E-80	5.86E-78	Up
THUMPD1	4265.971897	4160.607454	11834.85645	10681.49955	7735.733836	1.418058194	0.159559004	8.887359277	6.26E-19	6.96E-18	Up
IDI1	2450.461045	2149.949994	12347.25018	12235.27881	7295.735007	2.417871475	0.162457764	14.88307743	4.25E-50	1.76E-48	Up
DNTTIP2	3504.191201	3751.814145	20014.34252	17500.53837	11192.72156	2.370267624	0.162144594	14.618234	2.15E-48	8.32E-47	Up
COASY	1702.240843	1554.928623	9822.908719	8195.452718	5318.882726	2.468015701	0.173079346	14.25944668	3.92E-46	1.41E-44	Up
MEF2A	10323.52436	10203.9352	20634.08439	20788.39393	15487.48447	1.012856205	0.151907795	6.667572309	2.60E-11	1.77E-10	Up
IP6K2	4789.247433	3832.815782	22820.88787	18583.78636	12506.68436	2.2638163	0.182301767	12.41796134	2.09E-35	4.88E-34	Up

DNAJA2	307.1053067	326.2776224	11521.66547	9224.465025	5344.878355	5.033643201	0.183207549	27.47508625	3.49E-166	2.73E-163	Up
BUD23	4492.511916	4650.402399	10759.16162	9454.600256	7339.169049	1.144734132	0.162309295	7.05279469	1.75E-12	1.29E-11	Up
AFF4	3062.27863	2713.933355	11954.3781	8852.144333	6645.683604	1.84909439	0.188534086	9.807745787	1.04E-22	1.41E-21	Up
MCM2	1917.613396	2194.614448	17457.90728	13925.38022	8873.878837	2.93206515	0.178112858	16.4618388	6.90E-61	4.10E-59	Up
KDM5A	1927.185509	1824.429396	10486.91787	8705.561383	5736.02354	2.355166973	0.171345936	13.74509966	5.45E-43	1.72E-41	Up
PICALM	10295.6057	10836.80781	26270.41541	26657.57523	18515.10104	1.324560694	0.152498699	8.685718022	3.76E-18	3.99E-17	Up
CLASP1	3189.906809	3295.328283	8133.005428	6688.579995	5326.705129	1.192699442	0.171835438	6.940939882	3.89E-12	2.81E-11	Up
ENO1	36102.02358	38933.02051	78039.88893	73328.12059	56600.7634	1.012430858	0.153407811	6.599604347	4.12E-11	2.75E-10	Up
FNDC3B	2933.852774	3496.696839	8705.15998	7767.430505	5725.785025	1.357123473	0.172622991	7.861777078	3.79E-15	3.34E-14	Up
RAB7A	6369.443829	7320.428325	22896.14224	18923.85881	13877.4683	1.611146378	0.171902424	9.372447108	7.09E-21	8.72E-20	Up
SART3	2446.472664	2231.708656	20826.64705	15641.86656	10286.67373	2.96279354	0.183166554	16.17540686	7.52E-59	4.11E-57	Up
TOP2B	4681.561156	3982.706662	9677.933388	8062.062234	6601.06586	1.034070796	0.17634632	5.86386376	4.52E-09	2.52E-08	Up
SNRPA	4923.257021	4271.133052	13779.29658	11492.10326	8616.447478	1.458840703	0.172977666	8.433693994	3.35E-17	3.35E-16	Up
SAR1A	2702.526699	2808.561436	11636.76039	9322.675601	6617.631031	1.927365515	0.174010719	11.07613097	1.64E-28	2.89E-27	Up
FDFT1	6118.175851	6986.580457	31621.22249	27742.28906	18117.06697	2.179507267	0.164511195	13.24838267	4.61E-40	1.30E-38	Up
CNOT4	4817.963773	3396.012561	11582.53297	9351.992191	7287.125374	1.349929562	0.20189777	6.686203424	2.29E-11	1.56E-10	Up
RSBN1	502.5359565	417.1205799	11696.52121	9335.868067	5488.011453	4.515877962	0.188002043	24.02036644	1.70E-127	6.43E-125	Up
BZW1	5663.500462	7046.385404	18318.90582	17112.09355	12035.22131	1.479061071	0.171233229	8.637698864	5.74E-18	6.01E-17	Up
CCNT2	3682.870652	2957.695291	13543.57333	10444.03517	7657.043611	1.853127752	0.190221043	9.741970315	2.00E-22	2.66E-21	Up
GSK3B	2233.49314	2727.559799	14017.23319	10272.53312	7312.704812	2.291746836	0.195477054	11.72386626	9.62E-32	1.97E-30	Up
DIS3	3466.700423	2771.467228	9747.654349	8064.993893	6012.703973	1.513941017	0.184224916	8.217894996	2.07E-16	1.99E-15	Up
PIBF1	6531.372082	5321.126236	14330.42418	11733.96512	9479.221905	1.137047678	0.180629474	6.294917714	3.08E-10	1.92E-09	Up
YTHDC1	5579.744469	3954.69675	22373.78837	18702.51855	12652.68704	2.107204263	0.195976391	10.75233731	5.78E-27	9.55E-26	Up
WBP11	3209.848712	2948.610996	20347.45378	17154.6026	10915.12902	2.606446635	0.16701486	15.6060762	6.62E-55	3.26E-53	Up
EIF3I	7493.369484	6843.502798	22146.91858	18896.00805	13844.94973	1.517494383	0.164783487	9.209019706	3.29E-20	3.92E-19	Up
RAB10	3185.918429	3096.230802	11237.24821	8327.377373	6461.693704	1.639165626	0.185109539	8.855111589	8.36E-19	9.19E-18	Up
ATRX	4693.526298	4920.660198	10823.34917	8482.7553	7230.072743	1.006028765	0.17637935	5.703778609	1.17E-08	6.33E-08	Up
OVGP1	587.0896253	342.9321646	13139.63443	10483.61256	6138.317196	4.667522017	0.231516731	20.16062509	2.17E-90	2.99E-88	Up
RRN3	4156.690268	4264.31983	24893.7031	18624.82959	12984.8857	2.369669152	0.180509167	13.12769423	2.29E-39	6.28E-38	Up
SRRT	2590.852042	2601.893708	9154.47284	7974.112464	5580.332763	1.721995094	0.164973774	10.43799296	1.66E-25	2.57E-24	Up
OGFOD1	2439.293579	2608.70693	12340.61009	9686.201317	6768.702978	2.125639347	0.177657925	11.96478765	5.43E-33	1.17E-31	Up
NOP14	2518.263515	2084.845875	9608.212427	7575.406841	5446.682164	1.900638823	0.186697338	10.18032094	2.43E-24	3.56E-23	Up
SF3B2	4370.467469	4113.671926	19058.16934	15222.63933	10691.23702	2.014694369	0.172477171	11.6809335	1.60E-31	3.24E-30	Up
DDX18	2110.651017	2028.826051	15002.1801	11670.93446	7703.147907	2.688045843	0.177586163	15.13657259	9.29E-52	4.12E-50	Up
TPX2	1010.655646	926.5981665	11042.47219	8136.819538	5279.136386	3.30777755	0.190579163	17.35644913	1.76E-67	1.24E-65	Up
DDX24	9059.20771	9309.889095	37222.13969	31093.17529	21671.10295	1.894978147	0.16352196	11.58852391	4.71E-31	9.38E-30	Up
CCNK	5320.49973	5902.521164	25209.10745	20674.05923	14276.54689	2.03156508	0.170146992	11.94005875	7.32E-33	1.56E-31	Up
PABPC4	11659.63187	10857.24747	24652.44644	21659.09665	17207.10561	1.040431418	0.160031603	6.501412219	7.96E-11	5.20E-10	Up

TNRC6A	5628.402712	5302.957644	13291.24986	10590.61812	8703.307082	1.127623767	0.173481628	6.499960725	8.03E-11	5.24E-10	Up
ALKBH5	2473.593652	2427.778039	27200.02822	21928.80928	13507.5523	3.325403032	0.169932621	19.56895038	2.84E-85	3.43E-83	Up
EZR	1245.172425	1253.632814	11593.59979	8846.281015	5734.671511	3.032287883	0.182474077	16.61763649	5.19E-62	3.20E-60	Up
NUP50	3952.485181	3221.896893	13810.28368	11162.29162	8036.739343	1.799607291	0.182867886	9.841024195	7.49E-23	1.02E-21	Up
CDC45	3360.609499	2852.468866	8082.09806	6226.843704	5130.505032	1.203860525	0.187651545	6.415404289	1.40E-10	8.99E-10	Up
IKZF5	1423.851877	1004.571705	11129.90007	9353.458021	5727.945417	3.076690325	0.199956127	15.38682693	2.01E-53	9.34E-52	Up
WAC	7015.561487	6697.397042	25836.5961	20248.96867	14949.63082	1.748876613	0.173390681	10.08633569	6.35E-24	9.10E-23	Up
HSP90AB1	10341.87091	11481.03578	33301.16565	27633.81768	20689.4725	1.481475825	0.167693925	8.834403654	1.01E-18	1.10E-17	Up
SCD	1455.758922	1578.396387	17907.22014	14850.31863	8947.923521	3.432512512	0.170390241	20.14500644	2.98E-90	4.05E-88	Up
HNRNPM	12931.1276	15130.6516	43049.92668	36386.28561	26874.49787	1.501218337	0.169445628	8.859587313	8.03E-19	8.85E-18	Up
POLR2E	3158.797441	3283.215889	6990.909687	6600.630225	5008.38831	1.077199994	0.160444052	6.713866799	1.90E-11	1.30E-10	Up
RANBP1	4829.131239	4866.154424	15771.32404	13517.87962	9746.122331	1.595118954	0.162949017	9.789067687	1.25E-22	1.68E-21	Up
MED15	3741.101009	4025.857067	9780.854806	8227.700967	6443.878462	1.21342187	0.169111859	7.175261867	7.22E-13	5.46E-12	Up
CRKL	9608.806558	8444.609924	29569.43421	24946.95221	18142.45073	1.594493167	0.167961221	9.493222062	2.24E-21	2.82E-20	Up
PES1	2221.527998	2229.437582	13298.99663	11002.51621	7188.119604	2.449002047	0.169201576	14.47387254	1.77E-47	6.67E-46	Up
SNU13	1068.088327	1031.824592	10561.06556	8535.525162	5299.12591	3.185143395	0.175798598	18.11813873	2.29E-73	1.90E-71	Up
POLR2F	2084.327705	2041.69547	11136.54016	8991.398135	6063.490367	2.28657696	0.173535193	13.17644521	1.20E-39	3.34E-38	Up
GTPBP1	2520.656544	1932.683921	9695.640298	8091.378824	5560.089897	1.998119314	0.189166476	10.56275594	4.43E-26	7.05E-25	Up
MCM5	3398.897953	3124.240713	12219.98176	10470.4201	7303.38513	1.798604099	0.167048822	10.76693673	4.93E-27	8.19E-26	Up
RPL3	7511.716035	6501.327659	31569.20844	27764.2765	18336.63216	2.082141964	0.165325874	12.59416879	2.27E-36	5.55E-35	Up
MIEF1	1692.66873	1564.769943	12824.23009	10792.90259	6718.642836	2.858212059	0.16994031	16.81891752	1.77E-63	1.13E-61	Up
EIF3D	1634.438373	1215.024557	30192.49613	24642.05968	14421.00468	4.266548701	0.191230065	22.31107696	2.88E-110	6.68E-108	Up
TRMU	2637.117257	2127.239255	11668.85416	10521.72413	6738.733701	2.219764545	0.175548168	12.6447605	1.20E-36	2.96E-35	Up
AHSA1	5741.672722	6215.929367	14073.67397	12384.79342	9604.017371	1.145884867	0.162488423	7.052101607	1.76E-12	1.30E-11	Up
ERH	6875.17049	7602.798518	16073.4482	13995.74004	11136.78931	1.05450319	0.164118362	6.425260259	1.32E-10	8.44E-10	Up
HIF1A	2160.904613	1947.824414	17414.74669	12975.52271	8624.749606	2.887041303	0.185738416	15.54358743	1.76E-54	8.50E-53	Up
EIF5	6976.475358	8047.171985	20559.9367	18152.83249	13434.10413	1.365616676	0.165663391	8.243322016	1.67E-16	1.61E-15	Up
PPP4R3A	5259.078668	5161.394035	21586.93753	17327.57049	12333.74518	1.900996309	0.170200865	11.16913421	5.77E-29	1.04E-27	Up
YY1	5391.492904	5884.352572	27963.63875	22427.19131	15416.66888	2.159989582	0.171891463	12.56600851	3.25E-36	7.88E-35	Up
ACIN1	7348.990106	5954.755864	21933.32897	17576.7615	13203.45911	1.570508193	0.182435285	8.60857695	7.40E-18	7.72E-17	Up
CCNB1IP1	2120.223131	1676.052566	15952.81987	12560.69296	8077.447132	2.909219552	0.189466334	15.35480998	3.29E-53	1.52E-51	Up
CHD8	7108.091918	6639.863169	16394.38596	12252.86877	10598.80245	1.059353271	0.182139252	5.816172282	6.02E-09	3.32E-08	Up
TRPC4AP	3332.690835	2973.592809	7918.309135	6906.98859	5282.895342	1.233410788	0.169445974	7.279079906	3.36E-13	2.59E-12	Up
ADNP	15221.25575	16159.44809	45590.86836	38696.43289	28917.00127	1.425473202	0.162004525	8.798971516	1.38E-18	1.50E-17	Up
CSTF1	763.3760482	757.7816705	13912.09841	11525.81734	6739.768367	4.063887709	0.17183615	23.64978327	1.19E-123	3.84E-121	Up
PRELID3B	4135.153013	4393.01402	10814.49572	10704.95282	7511.903892	1.335326475	0.156844957	8.513671739	1.69E-17	1.72E-16	Up
DIDO1	6205.122548	5274.947732	31094.4419	25709.18355	17070.92393	2.306940299	0.173368785	13.30654938	2.12E-40	6.06E-39	Up
NOP56	3891.06412	3027.341559	30080.72126	25089.13767	15522.06615	2.995487725	0.182740631	16.39201806	2.18E-60	1.27E-58	Up

MAPRE1	307.9029828	353.5305096	12670.4013	9960.311433	5823.036556	5.09637682	0.187820126	27.13434891	3.87E-162	2.82E-159	Up
RPRD1B	5661.107433	5765.499703	29749.82337	23277.37241	16113.45073	2.214415434	0.172985877	12.80113423	1.62E-37	4.15E-36	Up
CHMP4B	288.7587559	401.2230623	19205.35803	14253.72603	8537.266471	5.599184233	0.209120925	26.77486358	6.34E-158	4.31E-155	Up
FAM83D	932.4833859	1158.247708	10801.21554	9391.569588	5570.879055	3.271687345	0.180249957	18.15083561	1.26E-73	1.06E-71	Up
ADNP2	6469.153344	5303.714669	15256.71694	13122.10566	10037.92265	1.269476176	0.174874983	7.259335513	3.89E-13	2.98E-12	Up
RIOK3	6676.549136	6686.041672	14653.5753	12869.98298	10221.53727	1.042566276	0.160094003	6.512213177	7.41E-11	4.85E-10	Up
RBM3	2662.642893	2660.184606	8069.924558	7562.214375	5238.741608	1.554338998	0.159973886	9.716204559	2.57E-22	3.40E-21	Up
FNDC3A	4656.03552	4613.308192	9602.679017	9208.340901	7020.090908	1.021090711	0.156486162	6.525118267	6.79E-11	4.47E-10	Up
NUP93	4568.291147	4477.043756	12642.73425	9755.095303	7860.791114	1.308314553	0.177665447	7.363922353	1.79E-13	1.41E-12	Up
CTCF	2962.569115	2925.900256	9142.299339	6882.069489	5478.20955	1.444589267	0.183620047	7.867274266	3.62E-15	3.20E-14	Up
PSMD7	2509.489078	2917.572985	28819.10387	23783.08359	14507.31238	3.276889592	0.173345988	18.90375212	1.06E-79	1.06E-77	Up
USP10	9209.170821	9037.360222	23532.48434	18909.20051	15172.05397	1.217958792	0.169234199	7.19688335	6.16E-13	4.68E-12	Up
CRISPLD2	3533.705218	4940.342839	10426.05037	10839.80913	7434.976888	1.327322308	0.189678414	6.997751	2.60E-12	1.89E-11	Up
GSPT1	5988.952319	5852.557537	19485.34856	16078.68375	11851.38554	1.586674993	0.166709526	9.517602435	1.77E-21	2.24E-20	Up
ELOB	2198.395391	2307.411121	12592.93356	10888.1815	6996.730395	2.38174327	0.165149846	14.42171047	3.78E-47	1.41E-45	Up
RNF40	3051.90884	2735.130045	20044.22293	17040.2679	10717.88243	2.680043851	0.167757495	15.97570263	1.89E-57	9.94E-56	Up
RAB11A	3970.034056	3348.320009	9276.207851	8182.260253	6194.205542	1.254514204	0.172335282	7.279497193	3.35E-13	2.58E-12	Up
TMEM87A	1961.485582	1911.487231	11549.33251	8865.336798	6071.910531	2.398319987	0.180668102	13.27472842	3.24E-40	9.20E-39	Up
BPNT2	1731.754859	1882.720294	8889.975861	7754.23804	5064.672264	2.203271036	0.168358441	13.08678687	3.92E-39	1.07E-37	Up
RAB2A	3883.885035	3989.519884	8654.252612	7364.327393	5972.996231	1.024874426	0.166806679	6.144085077	8.04E-10	4.83E-09	Up
NDRG1	35012.398	40687.04662	142276.1343	155726.7941	93425.59325	1.976961064	0.160974529	12.28120422	1.14E-34	2.60E-33	Up
UBR5	6385.397352	5433.165883	13622.14775	10811.95837	9063.167339	1.048023954	0.180159001	5.817216715	5.98E-09	3.31E-08	Up
SQLE	5038.920059	4504.296643	28611.04767	23511.90513	15416.54238	2.449466049	0.170313786	14.38207736	6.71E-47	2.47E-45	Up
BNIP3L	18297.89255	21260.28015	51227.19938	52075.05871	35715.1077	1.384811711	0.158947834	8.71236606	2.98E-18	3.17E-17	Up
HNRNPL	3031.966937	3126.511787	17540.90843	15193.32274	9723.177472	2.410232909	0.16249177	14.83295377	8.97E-50	3.67E-48	Up
SNRNP70	4644.070379	4074.306644	26313.576	19818.0148	13712.49196	2.403748743	0.183582003	13.09359687	3.58E-39	9.78E-38	Up
RPS16	10299.59408	10398.49054	24614.81926	23530.96092	17210.9662	1.217932734	0.152403027	7.991525856	1.33E-15	1.21E-14	Up
FBL	5810.272868	5517.952644	22419.16233	18708.38187	13113.94243	1.860279645	0.165714565	11.22580656	3.05E-29	5.54E-28	Up
RPS19	2920.29228	3199.186153	17219.97067	15794.31283	9783.440484	2.431629983	0.160295276	15.16969207	5.61E-52	2.50E-50	Up
CDC37	5456.902346	5270.405584	18584.50948	15303.25995	11153.76934	1.659593931	0.16745427	9.91072922	3.74E-23	5.16E-22	Up
TNPO2	1973.450724	1492.095577	11385.54359	9050.031315	5975.280302	2.560214913	0.19529532	13.10945349	2.91E-39	7.96E-38	Up
RPL18A	18829.14484	17606.87921	54120.06592	51088.55546	35411.16136	1.529832984	0.153256503	9.982173366	1.82E-23	2.56E-22	Up
FKBP8	4389.611696	4066.736398	8701.839935	8780.318688	6484.626679	1.047787633	0.158668222	6.603638816	4.01E-11	2.68E-10	Up
CDK6	3189.109133	4083.39094	9337.075357	7616.450067	6056.506374	1.221186426	0.18830883	6.485019462	8.87E-11	5.77E-10	Up
HBP1	1887.301703	1668.482319	10273.32826	8362.557281	5547.917391	2.3901003	0.177546091	13.46185819	2.62E-41	7.74E-40	Up
MTPN	2433.709846	2495.910257	8753.853985	7912.547625	5399.005428	1.75750735	0.162314769	10.82777221	2.54E-27	4.28E-26	Up
DNAJB6	5032.53865	4468.716484	14258.48985	10911.63478	8667.844941	1.405717564	0.182094691	7.719706451	1.17E-14	9.93E-14	Up
GRB10	5569.374679	5367.304739	11280.40881	11471.58164	8422.167468	1.056810112	0.15523657	6.807739374	9.91E-12	6.92E-11	Up

BUD31	2338.786388	2689.708567	11833.74977	9891.417446	6688.415542	2.111260521	0.174230229	12.11764762	8.52E-34	1.88E-32	Up
EIF3B	4790.045109	4592.111502	51483.94959	39002.79126	24967.22436	3.269776134	0.177441252	18.427373	7.92E-76	7.09E-74	Up
RHEB	3505.786553	2761.625908	7163.552066	6621.151838	5013.029092	1.137294316	0.179298237	6.343031241	2.25E-10	1.42E-09	Up
TLE4	4168.65541	3218.11177	8742.787166	6682.716677	5703.067756	1.062597599	0.197240021	5.38733261	7.15E-08	3.56E-07	Up
FUBP3	3564.01691	3389.199339	11630.12029	9247.918297	6957.81371	1.586441751	0.174844372	9.073450469	1.15E-19	1.34E-18	Up
BAG1	1922.399453	2305.897071	10003.29787	8443.177903	5668.693075	2.125271833	0.178258306	11.92242809	9.04E-33	1.92E-31	Up
ZFAND5	2479.975061	2146.921896	13015.68606	11562.46307	7301.261522	2.409418001	0.168483152	14.30064655	2.17E-46	7.89E-45	Up
EIF3A	2462.426187	2401.282177	28364.2576	20568.5195	13449.12137	3.330784307	0.185715335	17.93489107	6.30E-72	5.04E-70	Up
TWNK	1497.23808	1576.882337	20877.55442	16956.71562	10227.09761	3.621515835	0.171256262	21.14676449	2.96E-99	5.19E-97	Up
GTPBP4	1804.343387	1607.163323	23189.41295	16684.07134	10821.24775	3.547127151	0.191005877	18.57077495	5.54E-77	5.04E-75	Up
EDRF1	3381.349078	2623.090398	12017.45897	9256.713274	6819.652929	1.825243158	0.19475595	9.371950692	7.12E-21	8.75E-20	Up
BCCIP	4770.900882	4746.544529	15710.45653	12724.86586	9488.191952	1.579177409	0.169908989	9.294254647	1.48E-20	1.79E-19	Up
TASOR2	3819.273269	3520.164603	8941.989911	7491.85456	5943.320586	1.163151402	0.17053644	6.820544657	9.07E-12	6.35E-11	Up
SMC3	1706.229224	1651.827777	15936.21964	12166.38483	7865.165368	3.065183293	0.180366386	16.99420475	9.07E-65	5.96E-63	Up
UBE2S	2175.262783	2053.807864	13182.79503	11725.17015	7284.258955	2.558322398	0.162693049	15.72484145	1.02E-55	5.11E-54	Up
RPL19	3020.799472	2978.134957	18704.03112	15939.42995	10160.59888	2.529903578	0.163813649	15.44378986	8.31E-54	3.92E-52	Up
RNF167	3053.504193	2798.720116	8118.618563	7600.325942	5392.792203	1.425567713	0.162523866	8.771436163	1.76E-18	1.91E-17	Up
C1QBP	5847.763646	5942.64347	25710.43436	21121.13722	14655.49467	1.989946642	0.166530521	11.94944102	6.54E-33	1.40E-31	Up
UTP6	1734.945564	1579.153411	14276.19676	9992.559682	6895.713855	2.872661898	0.19622703	14.63948114	1.57E-48	6.14E-47	Up
LRRC59	10238.17302	12054.10344	40596.41286	33476.61405	24091.32584	1.732436451	0.172912485	10.0191519	1.26E-23	1.78E-22	Up
SUPT6H	1536.32421	1839.569889	17775.52499	13853.55458	8751.243418	3.227941233	0.185018395	17.4465962	3.65E-68	2.68E-66	Up
PHF12	3812.094184	3869.90999	19599.3368	15718.0897	10749.85767	2.200936959	0.170943708	12.87521481	6.21E-38	1.62E-36	Up
MANBA	2550.17056	1737.371562	24973.3842	18227.5898	11872.12903	3.333042099	0.214985976	15.50353267	3.28E-54	1.56E-52	Up
RPL34	1124.723331	907.6725504	11147.60698	10271.06729	5862.767536	3.397886822	0.175925596	19.31434021	4.07E-83	4.51E-81	Up
FBXW7	1802.748034	1269.530331	9464.343777	7865.641081	5100.565806	2.496199607	0.201844621	12.36693649	3.95E-35	9.09E-34	Up
NCAPG	4913.684908	3700.336469	9832.868857	7982.907441	6607.449419	1.048619551	0.192822686	5.438258182	5.38E-08	2.72E-07	Up
KMT5B	2759.161704	2592.052387	8922.069637	6735.486539	5252.192567	1.549225265	0.184557374	8.394274527	4.69E-17	4.65E-16	Up
CCDC86	892.5995798	1119.639451	17735.68445	14658.29497	8601.554612	4.008749274	0.183612841	21.83261938	1.14E-105	2.37E-103	Up
C11orf58	6635.069978	6499.056585	17525.41488	14926.54177	11396.5208	1.305089257	0.163005851	8.006395187	1.18E-15	1.08E-14	Up
KCTD10	1376.788985	1536.760031	10946.19087	8535.525162	5598.816261	2.741411531	0.182402121	15.02949369	4.71E-51	2.02E-49	Up
RSRC2	9080.744966	7040.329206	46906.71317	36541.66353	24892.36272	2.372007829	0.189297669	12.53057076	5.08E-36	1.22E-34	Up
CDKN1B	2613.98465	2645.044113	7891.748769	7543.158592	5173.484031	1.553385771	0.159303785	9.751091436	1.82E-22	2.43E-21	Up
DDX55	4652.04714	3836.600905	15707.13649	12173.71397	9092.374626	1.715849715	0.186091716	9.220451905	2.96E-20	3.53E-19	Up
GAPDH	2168.083698	2367.216068	9692.320253	8534.059332	5690.419838	2.006852548	0.166586555	12.04690588	2.01E-33	4.40E-32	Up
SRSF9	4228.481119	3741.972824	15922.93946	14054.37322	9486.941656	1.911253685	0.165415266	11.55427628	7.02E-31	1.39E-29	Up
PAK1IP1	487.3801102	369.4280272	18232.58463	14171.63958	8315.258086	5.241658013	0.197627181	26.52296101	5.27E-155	3.36E-152	Up
SERINC1	648.5106867	425.447851	18470.52124	15427.85546	8743.083809	4.980797637	0.208917483	23.8409805	1.26E-125	4.42E-123	Up
FBXO5	1357.644759	954.6080784	23598.88526	19988.05102	11474.79728	4.236812977	0.196590268	21.55148889	5.13E-103	1.02E-100	Up

SRSF3	3179.53702	2761.625908	19265.11886	16898.08244	10526.09106	2.605826731	0.167301842	15.575601	1.07E-54	5.19E-53	Up
MCM3	5440.151148	4670.08504	27289.66946	21490.52626	14722.60798	2.270590096	0.178817162	12.69783098	6.08E-37	1.52E-35	Up
MDN1	3600.710012	3028.855608	7999.096916	5554.027964	5045.672625	1.032025904	0.203180273	5.079360743	3.79E-07	1.74E-06	Up
E2F3	5230.362328	4737.460234	37057.24408	28827.00289	18963.01738	2.724668913	0.176201587	15.46336194	6.13E-54	2.91E-52	Up
GMNN	3753.066151	2827.487052	11401.03714	9920.734036	6975.581094	1.696222365	0.186681203	9.086197934	1.03E-19	1.19E-18	Up
HBS1L	6217.885366	4249.179337	20683.88508	15895.45507	11761.60121	1.805310887	0.209051911	8.635706227	5.84E-18	6.11E-17	Up
SLC39A7	2572.505491	2902.432492	10001.08451	8368.420599	5961.110773	1.746528426	0.173293849	10.07842136	6.88E-24	9.86E-23	Up
BYSL	504.1313087	523.8610549	33297.8456	27007.90848	15333.43661	5.874387408	0.17333827	33.88973129	9.44E-252	6.41E-248	Up
VEGFA	2602.019508	2738.915169	7652.705475	7578.3385	5142.994663	1.511841543	0.159531398	9.476764815	2.62E-21	3.29E-20	Up
PRPF4B	8235.208277	6826.091232	21061.26361	16415.82454	13134.59692	1.315291376	0.183451742	7.169685949	7.52E-13	5.68E-12	Up
TTK	2907.529462	2251.391297	10316.48886	9677.40634	6288.203989	1.954565304	0.179428221	10.89329927	1.24E-27	2.11E-26	Up
CEP72	3839.215172	4605.737945	13581.20052	11360.1786	8346.58306	1.562461877	0.176610077	8.846957695	8.99E-19	9.88E-18	Up
HMGCS1	2547.777532	2733.615996	19862.72709	17726.27611	10717.59918	2.831352626	0.1608714	17.6000994	2.46E-69	1.84E-67	Up
HMGCR	2138.569681	2302.111948	31689.83677	24186.1867	15079.17628	3.653426824	0.178910811	20.42038042	1.10E-92	1.63E-90	Up
CLINT1	3124.497367	2804.019288	9230.833892	7544.624421	5675.993742	1.500856002	0.17541401	8.556078265	1.17E-17	1.20E-16	Up
TARS1	13919.44832	16307.0679	34115.68354	29810.57448	23538.19356	1.080623558	0.166607347	6.48604983	8.81E-11	5.73E-10	Up
PDE4D	8898.077134	11997.32659	20865.38091	21099.14978	15714.9836	1.00595413	0.180824656	5.563146937	2.65E-08	1.38E-07	Up
BRIX1	3508.179582	4343.807418	30713.74332	24306.38472	15718.02876	2.808849028	0.18390416	15.27343929	1.15E-52	5.20E-51	Up
NUP155	3151.618356	3297.599357	11183.0208	8268.744193	6475.245676	1.592934257	0.185734849	8.576388665	9.79E-18	1.01E-16	Up
TCERG1	9609.604234	7164.481248	31226.13705	24230.16159	18057.59603	1.725209741	0.195114256	8.842048619	9.40E-19	1.03E-17	Up
CSNK1A1	9320.845478	9774.702227	37228.77978	30457.00529	21695.33319	1.825668749	0.166632462	10.956261	6.20E-28	1.07E-26	Up
HMGXB3	2526.240276	2382.35656	8410.78259	7395.109813	5178.62231	1.687266533	0.165911448	10.16968119	2.71E-24	3.95E-23	Up
ATP6V0E1	2297.30723	2707.120134	9245.220757	7686.809883	5484.114501	1.758601086	0.178273623	9.864617405	5.93E-23	8.07E-22	Up
AMOTL2	1507.607869	1992.488868	19670.16444	14722.79147	9473.263162	3.296640715	0.19981014	16.49886591	3.74E-61	2.24E-59	Up
KPNA1	3512.167962	3037.182879	10935.12405	8912.243342	6599.179558	1.599743401	0.177375376	9.01897116	1.90E-19	2.17E-18	Up
TFDP2	3606.293745	2775.252352	7889.535406	6194.595455	5116.419239	1.142399947	0.195298836	5.849496971	4.93E-09	2.74E-08	Up
TFG	5954.652246	5383.959281	14576.10756	12856.79052	9692.877403	1.274775597	0.163601512	7.791954861	6.60E-15	5.72E-14	Up
RPL24	12178.91902	10546.11034	30771.29078	29322.45326	20704.69335	1.402964763	0.160009929	8.767985648	1.82E-18	1.96E-17	Up
FXR1	5557.409538	5350.650197	21230.58595	16566.80498	12176.36266	1.793015129	0.174496561	10.27536083	9.10E-25	1.36E-23	Up
NCBP2	6308.820444	6542.206989	17777.73836	15872.00179	11625.1919	1.38877723	0.158591742	8.756932829	2.01E-18	2.16E-17	Up
KLHL18	727.4806227	702.5188713	12652.69439	9587.99074	5917.671156	3.959357861	0.185101511	21.39019743	1.65E-101	3.11E-99	Up
EIF4G1	6608.746666	6975.982112	32690.27723	26195.83894	18117.71124	2.116003513	0.170069042	12.44202645	1.55E-35	3.65E-34	Up
EEF1B2	4632.902913	4007.688475	17350.55914	15109.77046	10275.23025	1.909593182	0.16818625	11.35403864	7.08E-30	1.32E-28	Up
NCL	5105.924853	4744.273456	66274.75344	52456.17438	32145.28153	3.591450671	0.171702374	20.91672105	3.77E-97	6.46E-95	Up
SF3B6	432.3404578	280.8561436	10206.92735	9316.812283	5059.234058	4.775645498	0.208371695	22.91887817	3.01E-116	8.30E-114	Up
PPM1G	2341.977092	2174.931808	11949.95137	9126.254449	6398.278679	2.222445431	0.182174471	12.19954375	3.13E-34	6.98E-33	Up
FN1	3568.802967	4249.179337	13065.48674	14463.33965	8836.702174	1.815957748	0.169043048	10.74257574	6.42E-27	1.06E-25	Up
SF3B1	5633.986445	4800.293279	20706.01872	16266.30993	11851.65209	1.825251603	0.180382008	10.11881187	4.56E-24	6.57E-23	Up

KDM3A	2884.396855	2548.144958	13479.38578	10603.81058	7378.934544	2.148536918	0.179755356	11.95256132	6.30E-33	1.35E-31	Up
BIRC6	4570.684175	4228.739672	10623.03975	8252.620069	6918.770916	1.101287378	0.17894619	6.154293526	7.54E-10	4.54E-09	Up
NOL10	1809.129443	1637.444309	10413.87686	7434.687209	5323.784456	2.37288732	0.194823669	12.17966651	3.99E-34	8.88E-33	Up
GORASP2	2795.854805	2691.222616	10982.71137	9480.985187	6487.693495	1.899114928	0.165258736	11.49176725	1.45E-30	2.84E-29	Up
CEBPZ	934.8764142	728.2577093	14074.78065	10578.89148	6579.201565	3.890213189	0.198601602	19.5880252	1.96E-85	2.37E-83	Up
RAB3GAP1	3564.01691	2932.713478	11554.86592	9473.65604	6881.313088	1.694772504	0.181150211	9.355619815	8.31E-21	1.02E-19	Up
SRSF7	6342.322841	5458.147697	18204.91758	15565.64343	11392.75789	1.517035351	0.169876296	8.930235643	4.25E-19	4.77E-18	Up
RND3	1010.655646	973.5336946	12847.47041	10040.93206	6218.14795	3.528213578	0.179438109	19.66256558	4.51E-86	5.54E-84	Up
DHCR24	4647.261083	6005.476516	23645.3659	19077.7709	13343.9686	2.003829183	0.187011048	10.71503103	8.65E-27	1.41E-25	Up
CACYBP	2777.508255	3162.84897	9407.902999	8072.32304	5855.145816	1.557218151	0.171481459	9.080970986	1.08E-19	1.25E-18	Up
TMEM59	3257.70928	3224.924991	9373.59586	8473.960323	6082.547613	1.461196237	0.160957161	9.078168521	1.10E-19	1.28E-18	Up
ERRFI1	12218.80283	15702.2052	44267.27679	48099.72912	30072.00348	1.725992801	0.174211847	9.907436457	3.86E-23	5.33E-22	Up
ASH1L	5270.246134	4551.989195	13487.13256	10404.45777	8428.456414	1.282580959	0.183019841	7.007879332	2.42E-12	1.76E-11	Up
MEF2D	2489.547175	2866.852334	15263.35704	12488.86732	8277.155965	2.373343156	0.175586231	13.51668149	1.25E-41	3.73E-40	Up
IVNS1ABP	4230.076472	4087.176063	18160.6503	14422.29642	10225.04981	1.970074461	0.172706923	11.40703818	3.86E-30	7.34E-29	Up
SMG7	5515.930379	5396.071676	17005.27438	13616.0902	10383.34166	1.488760389	0.171161245	8.697999301	3.38E-18	3.59E-17	Up
RLF	2464.819215	1868.336826	19058.16934	14171.63958	9390.741239	2.939203673	0.200348003	14.67049151	9.96E-49	3.92E-47	Up
SDHB	3487.440003	3463.387755	9546.238239	8298.060783	6198.781695	1.360366178	0.164184597	8.285589512	1.18E-16	1.14E-15	Up
KDM5B	6317.594881	6455.149155	26951.02479	21549.15944	15318.23207	1.925004157	0.170045132	11.32054848	1.04E-29	1.92E-28	Up
PRPF3	1555.468437	1374.756757	20650.68462	14961.72168	9635.657873	3.603500439	0.190981174	18.8683542	2.08E-79	2.04E-77	Up
SLC2A1	3654.154312	4681.44041	8188.339524	8665.983987	6297.479558	1.015647641	0.177994548	5.706060411	1.16E-08	6.25E-08	Up
DR1	492.1661669	395.1668651	13966.32583	11731.03347	6646.173081	4.85653455	0.184448391	26.33004564	8.69E-153	5.37E-150	Up
CNN3	3511.370286	3005.387844	8305.647807	7754.23804	5644.160994	1.301368761	0.167970597	7.747598613	9.36E-15	8.04E-14	Up
PRRC2C	6168.429447	5242.395673	19178.79767	14955.85836	11386.37029	1.58097916	0.18165948	8.702981856	3.23E-18	3.44E-17	Up
NEK2	2992.083131	3381.629093	7019.683417	6644.60511	5009.500188	1.100241416	0.165238423	6.658508321	2.77E-11	1.88E-10	Up
ARID1A	2209.562856	2487.582986	14418.95873	10876.45487	7498.139861	2.429150259	0.185276145	13.11097152	2.85E-39	7.81E-38	Up
CENPF	1342.488912	937.9535362	11574.7862	9264.042422	5779.817767	3.192248537	0.205407147	15.54107819	1.83E-54	8.82E-53	Up
CAMSAP2	6019.264012	6053.169068	23605.52535	18472.38332	13537.58544	1.801450121	0.173252496	10.39783068	2.54E-25	3.88E-24	Up
ANKRD13C	3733.921924	2929.685379	13567.92034	10592.08395	7705.902896	1.85845666	0.191237772	9.718041794	2.53E-22	3.34E-21	Up
TNFAIP3	654.0944195	725.2296107	13228.16899	11061.14938	6417.1606	4.13827867	0.174329987	23.73819178	1.46E-124	4.86E-122	Up
MED28	3810.498832	3222.653917	8353.23513	7075.558982	5615.486715	1.133615099	0.176308165	6.429736828	1.28E-10	8.20E-10	Up
MYL12B	1683.894292	2348.290451	12768.89599	10784.10761	6896.297086	2.546249848	0.196025273	12.98939577	1.41E-38	3.76E-37	Up
RPN2	9292.129138	10989.72678	21905.66192	20058.41084	15561.48217	1.048988216	0.16517599	6.350730593	2.14E-10	1.35E-09	Up
RAB3GAP2	4223.695063	3792.693476	8567.931422	7755.703869	6085.005957	1.026089393	0.164844141	6.224603334	4.83E-10	2.96E-09	Up
UBN1	1829.869022	1944.796315	10911.88373	8484.221129	5792.692549	2.361533	0.17998696	13.12057832	2.51E-39	6.89E-38	Up
ELL2	3490.630707	4255.235534	10644.0667	10833.94581	7305.96969	1.47129274	0.169603069	8.674918125	4.14E-18	4.37E-17	Up
HEATR1	4084.101741	3155.278724	10983.81805	7953.590851	6544.197342	1.387574518	0.203143002	6.830530724	8.46E-12	5.94E-11	Up
SET	8070.08932	8773.158621	20272.19941	17704.28867	13704.934	1.172998002	0.161983196	7.241479557	4.44E-13	3.40E-12	Up

RAB14	2009.34615	1850.925259	8842.388538	8054.733086	5189.348258	2.13016186	0.164527299	12.94716361	2.44E-38	6.43E-37	Up
AREL1	3043.932079	2933.470503	9009.497508	7676.549076	5665.862292	1.481249916	0.167448411	8.846007607	9.07E-19	9.95E-18	Up
SLIRP	2571.707815	2916.058936	8447.303093	7402.43896	5334.377201	1.530276253	0.169951889	9.004173272	2.17E-19	2.47E-18	Up
FAM98A	2076.350944	2225.652459	15584.29479	11901.06969	7946.841971	2.675715276	0.18092896	14.78876169	1.73E-49	6.99E-48	Up
MXI1	5024.561889	5215.142785	14579.42761	14416.4331	9808.891347	1.501672482	0.154431018	9.723904596	2.38E-22	3.16E-21	Up
SMNDC1	567.9453984	551.1139422	22208.89276	18378.57023	10426.63058	5.180818498	0.171461697	30.21560267	1.48E-200	3.77E-197	Up
KANSL1	4156.690268	4077.334743	19084.72971	15048.20562	10591.74008	2.051624432	0.173340127	11.83583092	2.55E-32	5.32E-31	Up
TCP1	14413.20984	13917.89811	40970.47135	36500.62031	26450.5499	1.451307127	0.156385105	9.280341152	1.69E-20	2.04E-19	Up
EPC1	1214.063057	1200.641088	10008.83128	8089.912994	5128.362105	2.906189177	0.175393338	16.56955279	1.16E-61	7.02E-60	Up
HSPH1	6990.833528	8052.471158	17945.95401	15105.37297	12023.65792	1.135663738	0.170440158	6.663123011	2.68E-11	1.82E-10	Up
ETF1	2866.050304	3196.158055	27693.60836	20539.20291	13573.75491	2.992165469	0.184775492	16.19351913	5.60E-59	3.09E-57	Up
CLU	4379.241906	5635.291464	11017.01851	11814.58575	8211.534407	1.188836345	0.177547513	6.695877213	2.14E-11	1.46E-10	Up
TENT4B	3302.379142	3105.315097	7590.731287	6099.316537	5024.435516	1.095546607	0.17605031	6.222917808	4.88E-10	2.99E-09	Up
B4GALT4	4064.159838	5156.094863	9629.239383	9758.026962	7151.880262	1.072167796	0.174956215	6.12820638	8.89E-10	5.33E-09	Up
TBC1D15	3663.726425	2812.346559	13937.5521	11278.09215	7922.929308	1.961322325	0.189782773	10.3345646	4.92E-25	7.45E-24	Up
GTF3A	4250.018375	4040.240535	10639.63998	9349.060532	7069.739854	1.269840997	0.163075816	7.786813698	6.87E-15	5.95E-14	Up
MED13L	6300.843683	5434.679933	13162.87475	10430.8427	8832.310267	1.007709251	0.179322214	5.619544999	1.91E-08	1.01E-07	Up
DDX39A	1955.90185	1626.088939	15797.8844	12823.07644	8050.737908	2.998454683	0.180819879	16.58255003	9.32E-62	5.69E-60	Up
TRIR	2650.677751	2283.943357	11706.48135	10057.05618	6674.539658	2.141090904	0.172266514	12.42894431	1.82E-35	4.27E-34	Up
STIL	3568.802967	3112.128319	7715.786344	5952.733588	5087.362804	1.03306865	0.185422105	5.571442779	2.53E-08	1.32E-07	Up
AMD1	2868.443332	2274.859061	12819.80336	9501.5068	6866.153138	2.117910464	0.197265105	10.7363665	6.87E-27	1.13E-25	Up
EXOSC9	5409.041779	4444.491696	18400.80028	14705.20151	10739.88382	1.748526383	0.182024356	9.606002291	7.54E-22	9.72E-21	Up
ACSL3	3701.217203	3813.890166	10646.28007	8746.604609	6726.998011	1.367841982	0.170236297	8.034960847	9.36E-16	8.60E-15	Up
PIGT	4090.48315	4055.381028	8149.605657	8208.645184	6126.028755	1.005871883	0.156822432	6.414081628	1.42E-10	9.06E-10	Up
CHD6	3667.714806	3580.726575	10448.184	9085.211223	6695.459152	1.430351678	0.16379773	8.73242672	2.49E-18	2.67E-17	Up
ZNFX1	2986.499398	3293.057209	11256.06181	9117.459472	6663.269471	1.698112489	0.174814248	9.713810579	2.63E-22	3.47E-21	Up
STAU1	4284.318448	4590.597452	10929.59064	8538.456821	7085.74084	1.133503773	0.177671011	6.379790195	1.77E-10	1.13E-09	Up
USP9X	7251.673619	4591.354477	14706.69603	10344.35876	9223.520722	1.081019323	0.229883804	4.702459697	2.57E-06	1.07E-05	Up
WRNIP1	4178.227524	3422.508424	11441.98437	9916.336548	7239.764216	1.490755481	0.176015395	8.469460762	2.47E-17	2.49E-16	Up
SNRPC	2663.440569	2638.230891	9801.881763	8726.082996	5957.409055	1.805318192	0.162514471	11.10866119	1.14E-28	2.03E-27	Up
XPO5	2899.552701	2707.877158	7964.789776	6574.245294	5036.616232	1.374782487	0.173057584	7.944075347	1.96E-15	1.76E-14	Up
RIOK1	765.7690765	632.1155793	12942.64505	9804.933506	6036.365803	4.024794147	0.193072318	20.84604454	1.66E-96	2.74E-94	Up
NUP153	5171.334295	4026.614091	26492.85848	18890.14473	13645.2379	2.302907118	0.201495253	11.42908868	2.99E-30	5.73E-29	Up
GOT2	4427.90015	4619.364389	10400.59668	9140.912744	7147.193491	1.111112334	0.162672807	6.830350776	8.47E-12	5.95E-11	Up
TTF1	2767.936141	2994.032474	11082.31274	9246.452468	6522.683457	1.819026293	0.170422945	10.67359969	1.35E-26	2.20E-25	Up
GTF3C4	2026.097348	2362.67392	34931.30811	26236.88217	16389.24039	3.800904766	0.185586154	20.48054061	3.21E-93	4.85E-91	Up
POLR1B	3316.737313	3548.174515	18062.15561	12543.10301	9367.542611	2.156619446	0.194483955	11.08893251	1.42E-28	2.52E-27	Up
PANK2	3138.057861	2311.196244	8492.677052	6717.896585	5164.956936	1.481236294	0.199959371	7.407686291	1.29E-13	1.03E-12	Up

PSMF1	5706.574972	4855.556078	11425.38414	9951.516456	7984.757911	1.017303013	0.170859286	5.954039936	2.62E-09	1.50E-08	Up
STK35	3369.383937	2841.113496	7400.381997	6937.77101	5137.16261	1.207220253	0.170135509	7.095639586	1.29E-12	9.57E-12	Up
SNRPB	1640.819782	1484.525331	18634.31016	16874.62917	9658.571112	3.506225436	0.162617165	21.56122598	4.15E-103	8.31E-101	Up
MGME1	3436.388731	3127.268812	13241.44917	10700.55533	7626.41551	1.867159265	0.174138349	10.72227502	8.00E-27	1.31E-25	Up
MAX	5427.38833	4711.721396	14395.71841	12396.52006	9232.837048	1.402018875	0.169292051	8.281658022	1.21E-16	1.18E-15	Up
KDM5C	3061.480954	2576.911894	18799.20577	14381.2532	9704.712954	2.557157516	0.185905464	13.75514987	4.74E-43	1.51E-41	Up
AMOT	3899.040881	1946.310364	8483.823596	5743.11997	5018.073703	1.283335358	0.555861237	2.308733318	0.020958384	0.044507646	Up
PCID2	11678.77609	14044.32123	40634.04005	34768.00984	25281.2868	1.551562679	0.171107396	9.067770955	1.21E-19	1.41E-18	Up
PRMT1	3729.933544	3598.138142	9531.851374	7947.727533	6201.912648	1.254371473	0.169093278	7.41822198	1.19E-13	9.50E-13	Up
DNAJC8	6357.478687	6055.440142	17297.4384	14243.46522	10988.45561	1.345507947	0.167817782	8.017672113	1.08E-15	9.86E-15	Up
ZNF384	1991.797275	2067.434308	15691.64294	12087.23003	7959.526139	2.774851237	0.178826474	15.51700466	2.66E-54	1.27E-52	Up
PPAT	1393.540184	1205.940261	9831.762175	7930.137579	5090.34505	2.772793385	0.18119133	15.30312396	7.29E-53	3.32E-51	Up
KRT17	3060.683278	4437.678474	15956.13992	17890.44901	10336.23767	2.174238988	0.196660517	11.05579816	2.06E-28	3.60E-27	Up
MKLN1	6418.102073	7412.028308	17094.91561	14662.69246	11396.93461	1.19935665	0.168962614	7.098355201	1.26E-12	9.39E-12	Up
HAT1	2966.557495	2874.42258	9753.187758	7931.603409	5881.442811	1.598449651	0.172598155	9.261105082	2.02E-20	2.42E-19	Up
LDLR	3220.218502	3907.761222	16125.46225	12566.55628	8954.999564	2.00917253	0.185617927	10.82423751	2.64E-27	4.44E-26	Up
PRKCSH	3814.487212	3311.225801	9904.803181	8315.650737	6336.541733	1.354663515	0.174010112	7.784970085	6.97E-15	6.04E-14	Up
TOMM40	650.903715	710.0891178	11235.03485	8582.431705	5294.614847	3.864148483	0.186336662	20.73745687	1.59E-95	2.55E-93	Up
SAFB2	3989.178283	3751.814145	15453.70633	11757.4184	8738.029288	1.813787344	0.180249697	10.06263741	8.08E-24	1.15E-22	Up
NDUFA10	7184.668825	5727.64847	14822.89763	11775.00835	9877.55582	1.04272976	0.186192863	5.600267076	2.14E-08	1.13E-07	Up
ZSWIM6	2965.759819	3231.738213	9393.516134	7493.320389	5771.083639	1.446344459	0.177056677	8.168821885	3.11E-16	2.95E-15	Up
PXDN	3694.835794	4841.17261	10152.69993	10458.69346	7286.850449	1.271725206	0.179668691	7.078168162	1.46E-12	1.08E-11	Up
H19	2716.087193	3421.751399	8820.2549	10221.22908	6294.830644	1.633146207	0.180990672	9.023372234	1.82E-19	2.09E-18	Up
COL5A1	7321.869118	10603.64421	27501.0457	29190.5286	18654.27191	1.661071282	0.192737362	8.618314928	6.79E-18	7.10E-17	Up
EXOSC2	4476.558393	4313.526432	20666.17817	16036.1747	11373.10942	2.062052592	0.17562789	11.7410315	7.85E-32	1.61E-30	Up
PRRC2B	12970.21373	13381.92466	34227.45841	28711.20236	22322.69979	1.256080463	0.162950831	7.708340344	1.27E-14	1.08E-13	Up
ATP5IF1	3069.457715	3078.06221	8134.11211	7826.063685	5526.92393	1.376451347	0.158349476	8.692490707	3.55E-18	3.77E-17	Up
ZNF317	3147.629975	2788.121771	10234.59439	9003.124771	6293.367728	1.696606796	0.168037612	10.09658952	5.72E-24	8.21E-23	Up
EIF3G	4316.225493	3550.445589	9462.130413	8586.829194	6478.907672	1.198238263	0.172859173	6.931875461	4.15E-12	2.99E-11	Up
SLC6A8	3458.723662	3721.533159	8457.26323	9322.675601	6240.048913	1.308007278	0.162367379	8.055850181	7.89E-16	7.29E-15	Up
DKC1	5935.508019	5399.856799	30717.06336	23312.55232	16341.24513	2.253020272	0.179800374	12.53067619	5.07E-36	1.22E-34	Up
RBM39	16274.9859	15061.76235	47734.51124	38574.76905	29411.50714	1.46171221	0.168824375	8.65818228	4.79E-18	5.04E-17	Up
EMC8	2272.57927	2228.680557	8100.911652	7484.525412	5021.674223	1.791913776	0.161448759	11.09896283	1.27E-28	2.25E-27	Up
CAP1	4498.893325	5059.195708	14404.57187	12109.21748	9017.969594	1.472045252	0.169928411	8.662737714	4.61E-18	4.85E-17	Up
RLIM	285.5680515	299.0247351	12813.16327	9687.667146	5771.3558	5.266476559	0.190735555	27.61140454	8.12E-168	6.62E-165	Up
SH3BP5	2724.063954	3293.057209	7484.489823	7798.212924	5324.955978	1.34463976	0.171243965	7.852187725	4.09E-15	3.60E-14	Up
SLC6A6	5102.734148	6300.716127	16140.9558	17330.50214	11218.72705	1.553383182	0.170636778	9.10344887	8.75E-20	1.02E-18	Up
PSMC3IP	1208.479324	1040.151863	13378.67773	11267.83134	6723.785065	3.454518605	0.175261443	19.71065933	1.75E-86	2.16E-84	Up

RAF1	5495.988476	5146.253542	23324.42814	18699.58689	13166.56426	1.981521271	0.1713575	11.56366819	6.30E-31	1.25E-29	Up
EIF5A	2929.864394	3415.695202	17877.33973	14765.30052	9747.049963	2.362994375	0.174664086	13.52879367	1.06E-41	3.18E-40	Up
PCNA	1760.4712	1519.348464	11331.31618	10876.45487	6371.897677	2.759511182	0.165788869	16.64473129	3.30E-62	2.05E-60	Up
KHDC4	5977.784854	5500.541077	21158.65162	17110.62772	12436.90132	1.737391682	0.171145506	10.15154718	3.26E-24	4.74E-23	Up
OSER1	1784.401484	1934.19797	13891.07146	11760.35006	7342.505241	2.786285813	0.16868361	16.5178218	2.73E-61	1.64E-59	Up
USPL1	1846.620221	1448.945172	16806.07163	13262.82529	8341.115579	3.189915	0.189982069	16.79061087	2.86E-63	1.82E-61	Up
SRRM1	7368.134333	6767.800334	28734.99605	23306.689	16544.40493	1.880388167	0.169974749	11.06275005	1.90E-28	3.35E-27	Up
ZFC3H1	6212.301633	5860.127784	22280.82709	18532.48233	13221.43471	1.757422399	0.166296118	10.56803019	4.19E-26	6.68E-25	Up
EIF2S1	682.0130838	585.1800512	23144.03899	17894.8465	10576.51966	5.017592443	0.185102606	27.10708692	8.12E-162	5.71E-159	Up
LOXL2	6018.466336	8299.261192	14738.78981	16534.55673	11397.76852	1.127037337	0.18853619	5.97783023	2.26E-09	1.30E-08	Up
CCNB1	5730.505256	5997.906269	15888.63232	13770.0023	10346.76154	1.338534631	0.162151454	8.254842011	1.52E-16	1.47E-15	Up
PRPF38B	2977.724961	2856.253989	29794.09064	24886.8532	15128.7307	3.228567218	0.165341761	19.5266289	6.52E-85	7.78E-83	Up
NOTCH2	1651.189571	1565.526968	9511.9311	7399.507301	5032.038735	2.394615168	0.1809258	13.23534378	5.48E-40	1.55E-38	Up
SLC38A2	8437.818012	10038.90383	24408.97642	20781.06478	15916.69076	1.290341942	0.171543309	7.521960189	5.40E-14	4.42E-13	Up
RBM17	2968.950524	2719.989553	12259.82231	9064.68961	6753.362998	1.906525375	0.187016705	10.1944122	2.10E-24	3.09E-23	Up
PUM1	8981.833127	8309.102513	51591.29773	38996.92794	26969.79033	2.389368343	0.178571356	13.38046814	7.87E-41	2.29E-39	Up
GNL2	1379.97969	988.6741875	21388.84146	15505.54442	9815.75994	3.961558976	0.210247358	18.8423722	3.39E-79	3.31E-77	Up
SYNCRIP	2720.87325	2588.267264	28366.47097	20760.54317	13609.03866	3.210083591	0.184609819	17.38847696	1.01E-67	7.19E-66	Up
AKIRIN2	3587.947194	2510.293726	20756.92609	17126.75184	10995.47971	2.635268957	0.19939888	13.21606698	7.09E-40	1.99E-38	Up
NAT10	7395.255321	7703.482796	20214.65195	16560.94166	12968.58293	1.28441876	0.167599437	7.663622149	1.81E-14	1.52E-13	Up
LTV1	815.224996	561.7122872	11378.9035	9133.583596	5472.356094	3.897474321	0.207624245	18.77176875	1.29E-78	1.22E-76	Up
USP15	4880.980187	4751.086677	17613.94943	14023.5908	10317.40177	1.715856963	0.172101123	9.970050959	2.06E-23	2.88E-22	Up
CPM	2739.219801	3744.243898	9030.524465	9815.194312	6332.295619	1.539251018	0.188408264	8.169763834	3.09E-16	2.93E-15	Up
DHX9	5775.175119	5212.871711	60096.14829	43277.15007	28590.3363	3.233921592	0.187190851	17.27606648	7.12E-67	4.94E-65	Up
MED4	3963.652647	3079.576259	12227.72853	9502.97263	7193.482517	1.625651271	0.193203313	8.414199774	3.96E-17	3.94E-16	Up
RAC1	2494.333232	2927.414305	11579.21293	9703.791271	6676.187933	1.972959604	0.175680427	11.23038941	2.89E-29	5.27E-28	Up
BZW2	5988.154643	7151.611829	14227.50276	12356.94266	9931.052973	1.016711891	0.171707775	5.921175626	3.20E-09	1.81E-08	Up
TBRG4	2162.499965	2124.968181	9321.58181	8582.431705	5547.870415	2.062191731	0.160879654	12.81822582	1.30E-37	3.35E-36	Up
TRA2B	7609.032522	8103.191809	34790.75951	28513.31538	19754.0748	2.010462902	0.167190517	12.02498166	2.63E-33	5.70E-32	Up
WDR33	4147.118155	3798.749673	10741.45471	8655.72318	6835.76143	1.287788675	0.174626959	7.374512404	1.65E-13	1.31E-12	Up
YME1L1	5772.78209	6582.329296	16610.18893	13597.03441	10640.58368	1.289882937	0.173279734	7.443934193	9.77E-14	7.86E-13	Up
PSMB7	3513.763315	4146.98101	25656.20694	20712.17079	13507.28052	2.597622501	0.1771992	14.65933536	1.17E-48	4.60E-47	Up
ANP32B	1730.957183	1670.753393	24168.82644	18686.39443	11564.23286	3.655262818	0.177021999	20.648636	1.00E-94	1.59E-92	Up
RPL35	898.9809888	831.2130611	55996.99845	48309.34274	26509.13381	5.913877522	0.164242857	36.00690852	6.52E-284	1.33E-279	Up
RPS6	2248.648986	1509.507144	22429.12247	19970.46107	11539.43492	3.496147552	0.200622123	17.42653052	5.19E-68	3.79E-66	Up
MTCH1	2277.365327	2358.888796	15284.38399	12368.6693	8072.326853	2.576532373	0.171727657	15.00359594	6.95E-51	2.97E-49	Up
CREBZF	12433.3777	10845.13508	25771.30186	23056.03216	18026.4617	1.068749869	0.163151507	6.550658878	5.73E-11	3.79E-10	Up
RNF121	1711.812956	1789.606263	11859.20345	8366.954769	5931.89436	2.530436557	0.194199185	13.03010904	8.25E-39	2.22E-37	Up

TGS1	1695.061758	1760.839326	9848.362403	7636.97168	5235.308792	2.339232853	0.180512583	12.95883543	2.09E-38	5.54E-37	Up
DCUN1D5	4437.472263	3822.974462	12452.38496	11473.04747	8046.46979	1.534360833	0.165621581	9.26425667	1.96E-20	2.36E-19	Up
YAP1	3087.00659	3757.113317	7674.839113	6763.3373	5320.57408	1.077047505	0.176384612	6.106244153	1.02E-09	6.07E-09	Up
FNBP1L	4388.81402	4239.338017	10762.48167	9030.975532	7105.402309	1.1980794	0.16737913	7.157878036	8.19E-13	6.18E-12	Up
FBXO11	4216.515978	4093.989285	10574.34574	8493.016106	6844.466778	1.198311165	0.172984169	6.927288026	4.29E-12	3.08E-11	Up
LRPPRC	4652.04714	3962.266996	10509.05151	8233.564285	6839.232483	1.121756773	0.1832005	6.123109775	9.18E-10	5.50E-09	Up
RPS24	12303.3565	11280.42425	27733.44891	24439.7752	18939.25121	1.145574194	0.160392138	7.142333834	9.18E-13	6.89E-12	Up
SSB	3103.757788	2713.933355	7896.175497	7103.409743	5204.319096	1.366587151	0.168780077	8.096851077	5.64E-16	5.28E-15	Up
PPIG	3552.849445	2782.065573	11497.31846	8306.85576	6534.77231	1.644680165	0.202095646	8.138127647	4.01E-16	3.79E-15	Up
ITPRID2	5424.995301	6302.230177	13781.50995	13056.14333	9641.219689	1.194408546	0.163408608	7.309336747	2.68E-13	2.09E-12	Up
FAM13A	6318.392557	6500.570634	15566.58788	14938.2684	10830.95487	1.250784434	0.154297594	8.106311967	5.22E-16	4.89E-15	Up
G3BP2	3683.668328	3751.05712	21575.87071	17024.14378	11508.68498	2.376355778	0.173008723	13.73546802	6.23E-43	1.95E-41	Up
AEBP2	7431.948423	7605.826617	18767.11199	15776.72288	12395.40248	1.199934135	0.16419705	7.307890941	2.71E-13	2.11E-12	Up
KANSL2	2498.321612	2894.105221	13588.94729	10803.16339	7446.13438	2.177513827	0.18004267	12.094432	1.13E-33	2.49E-32	Up
DIAPH3	5156.178449	4903.248631	13858.97768	10193.37832	8527.945771	1.257839013	0.185011953	6.798690519	1.06E-11	7.34E-11	Up
RBM26	8769.651278	7538.451423	18082.07589	15602.28917	12498.11694	1.046593002	0.168717708	6.203219635	5.53E-10	3.37E-09	Up
SERF2	5023.764212	5180.319652	11173.06066	10745.99604	8030.785142	1.10307115	0.155859997	7.077320467	1.47E-12	1.09E-11	Up
TLE3	3364.59788	2927.414305	10253.40799	8689.437259	6308.714358	1.590262443	0.172939911	9.195462359	3.73E-20	4.43E-19	Up
ANP32A	9008.954115	8535.452882	24312.69509	19763.77911	15405.2203	1.329093466	0.168460543	7.889642522	3.03E-15	2.69E-14	Up
HMG20A	2187.227925	1645.014555	11802.76267	8605.884977	6060.222533	2.413222232	0.205858748	11.72270919	9.75E-32	1.99E-30	Up
TICRR	2021.311292	1682.108763	14141.18157	10599.41309	7111.003679	2.740203869	0.191337153	14.32133708	1.61E-46	5.87E-45	Up
PMM2	2277.365327	2415.665645	11085.63279	8406.532166	6046.298982	2.054508297	0.182977654	11.22819239	2.96E-29	5.40E-28	Up
GNAL	3532.907542	3615.549709	9772.001351	8388.942212	6327.350203	1.345293891	0.165467839	8.130243931	4.28E-16	4.04E-15	Up
PELP1	2540.598447	2433.077212	12302.9829	8981.137329	6564.448972	2.09763366	0.187796113	11.16973949	5.73E-29	1.03E-27	Up
EIF4A3	1554.670761	1679.080664	12949.28514	10361.94871	6636.246321	2.849866149	0.176088415	16.18429103	6.51E-59	3.57E-57	Up
CSNK1D	5694.60983	5215.142785	21324.65391	17062.25535	12324.16547	1.815117319	0.172791586	10.50466264	8.22E-26	1.29E-24	Up
RPTOR	1836.250431	2426.26399	13965.21914	10666.84125	7223.643704	2.530810723	0.198453923	12.7526364	3.01E-37	7.64E-36	Up
FOXK2	3205.062656	3099.2589	8277.980759	6835.162945	5354.366315	1.261634529	0.17134934	7.362937766	1.80E-13	1.42E-12	Up
WDR45B	6918.245001	5635.291464	13915.41846	11313.27206	9445.556745	1.007135843	0.181429227	5.55112239	2.84E-08	1.48E-07	Up
BRD4	1855.394658	2154.492142	23096.45166	18251.04307	11339.34538	3.366192701	0.179494209	18.75376767	1.80E-78	1.71E-76	Up
URB1	894.9926082	878.1485892	10648.49343	8104.571289	5131.55148	3.403000257	0.184518337	18.44261288	5.98E-76	5.37E-74	Up
RPL13A	23857.69511	21881.04036	65918.40187	61424.11925	43270.31415	1.477243266	0.15483743	9.540608292	1.42E-21	1.81E-20	Up
ZNF614	1625.663935	1523.133587	12009.71219	10423.51355	6395.505817	2.832933579	0.166473778	17.01729613	6.11E-65	4.06E-63	Up
SERBP1	12166.95388	9044.930469	28889.93151	22768.72958	18217.63636	1.284226119	0.193778877	6.627276095	3.42E-11	2.30E-10	Up
MTF2	7091.340719	4713.99247	14397.93178	11001.05038	9301.078835	1.10550101	0.214416479	5.15585842	2.52E-07	1.18E-06	Up
POU2F1	2317.249133	1844.869062	9324.901855	7475.730435	5240.687621	2.013417051	0.189123409	10.64604888	1.82E-26	2.94E-25	Up
PFDN2	2145.748766	2164.333462	12468.98519	10618.46888	6849.384074	2.421454343	0.166177614	14.57148342	4.27E-48	1.64E-46	Up
PRCC	3144.43927	2773.738302	22447.93606	17148.73929	11378.71323	2.742303979	0.182006129	15.06709686	2.67E-51	1.16E-49	Up

MCL1	3394.909573	3793.4505	9234.153938	8384.544723	6201.764684	1.293447302	0.164801403	7.84852116	4.21E-15	3.70E-14	Up
PIP5K1A	5180.906408	4789.694934	17186.77021	13034.15589	10047.88186	1.599960805	0.180673961	8.855514085	8.33E-19	9.17E-18	Up
POGZ	5693.014478	5001.661835	17612.84275	13780.2631	10521.94554	1.553709362	0.178918355	8.683901433	3.82E-18	4.06E-17	Up
DTL	8237.601305	7733.763782	34571.63649	27139.83314	19420.70868	1.95012962	0.173033061	11.27027177	1.84E-29	3.39E-28	Up
TPM3	12312.92861	14699.90457	28908.74511	25594.84885	20379.10678	1.012735119	0.167839501	6.033949765	1.60E-09	9.33E-09	Up
ILF2	861.4902111	682.0792059	23470.51015	18421.07929	10858.78971	4.762656229	0.189545405	25.12673004	2.54E-139	1.18E-136	Up
SDE2	266.4238245	167.3024467	18679.68412	15426.38963	8634.950005	6.298450253	0.215994219	29.16027234	6.19E-187	1.05E-183	Up
ARF1	1932.769242	1679.080664	42318.40993	34741.62491	20167.97119	4.415298244	0.172747316	25.55928713	4.33E-144	2.26E-141	Up
LBR	10785.37884	8553.621473	22576.31116	19071.90759	15246.80476	1.106832475	0.179032085	6.18231351	6.32E-10	3.83E-09	Up
PDIA6	1159.023404	970.505596	12611.74716	10445.501	6296.694288	3.436906536	0.179852276	19.10960822	2.10E-81	2.21E-79	Up
SNRNP200	1820.296909	2064.406209	26327.96287	19684.62432	12474.32258	3.566195108	0.185207464	19.25513712	1.28E-82	1.39E-80	Up
SLC20A1	3571.993672	3319.553072	15398.37223	12006.60941	8574.132096	1.99171466	0.17732027	11.23230106	2.83E-29	5.17E-28	Up
CNOT9	2949.008621	2294.541702	8949.736685	7189.893683	5345.795173	1.622270861	0.191256234	8.48218555	2.21E-17	2.24E-16	Up
EAF1	2480.772737	2090.145047	12918.29805	10668.30708	7039.380728	2.367622038	0.17810871	13.29312886	2.54E-40	7.21E-39	Up
RPL32	1701.443167	1451.973271	44777.45715	40473.01824	22100.97296	4.756854363	0.165216398	28.79166006	2.73E-182	3.97E-179	Up
ARL6IP5	3154.80906	2896.376295	9258.50094	8026.882326	5834.142155	1.514452837	0.167428597	9.045365415	1.49E-19	1.72E-18	Up
ATG3	2644.296342	2800.234165	9759.82785	7714.660643	5729.75475	1.682581334	0.177258705	9.492235266	2.26E-21	2.84E-20	Up
MANF	299.1285455	256.6313549	12331.75663	11390.96102	6069.619388	5.416233999	0.174547241	31.03018976	2.11E-211	6.15E-208	Up
NDUFS6	3413.256123	3667.784409	10666.20034	9212.738389	6739.994816	1.489329565	0.166013678	8.971125677	2.93E-19	3.32E-18	Up
MYO10	3579.970433	4987.278367	15979.38024	13355.17255	9475.450397	1.775722548	0.195138579	9.099802605	9.05E-20	1.06E-18	Up
ATG12	2968.950524	2851.711841	10116.17943	8572.170899	6127.253173	1.683069559	0.167609865	10.04159008	1.00E-23	1.42E-22	Up
RNF145	5128.259784	4669.328016	14832.85777	12097.49084	9181.984102	1.458892292	0.171841293	8.489765563	2.07E-17	2.10E-16	Up
G3BP1	4026.669061	4678.412311	24898.12983	18796.33164	13099.88571	2.327591583	0.184706893	12.60154152	2.07E-36	5.07E-35	Up
FAM193B	4210.134569	3283.215889	10006.61792	7902.286819	6350.563799	1.257242232	0.191623123	6.561015243	5.34E-11	3.54E-10	Up
WTAP	4860.240608	4025.857067	20374.01414	16824.79097	11521.2257	2.06576372	0.177277437	11.65271651	2.22E-31	4.48E-30	Up
EGFR	4869.015045	7365.849804	13358.75745	13576.5128	9792.533776	1.13844031	0.201168081	5.659149815	1.52E-08	8.12E-08	Up
CDCA5	4509.263114	4077.334743	16266.01086	14582.07184	9858.670137	1.845122167	0.162303573	11.36833982	6.01E-30	1.13E-28	Up
CCT6A	5945.080133	5601.225355	18352.10627	15921.84	11455.06294	1.56977811	0.162167593	9.679974151	3.67E-22	4.81E-21	Up
NOM1	1757.280495	1096.928712	25818.88919	20558.2587	12307.83927	4.022510599	0.219143588	18.35559342	2.98E-75	2.61E-73	Up
KDM6A	3820.868621	3376.32992	15092.92802	11245.8439	8383.992616	1.871883367	0.186508046	10.03647515	1.05E-23	1.50E-22	Up
RPL7	17189.92042	16698.44964	36221.69923	34798.79226	26227.21539	1.067460767	0.151508759	7.045538334	1.85E-12	1.36E-11	Up
MTDH	7285.973693	7820.821616	20659.53808	15625.74244	12848.01896	1.264305777	0.179784958	7.032322357	2.03E-12	1.49E-11	Up
EIF3H	4435.079235	4358.190886	10731.49458	9325.60726	7212.592989	1.18978791	0.163192142	7.290718131	3.08E-13	2.38E-12	Up
REXO4	2129.795244	2210.511966	12539.81283	10375.14118	6813.815306	2.400557729	0.169957458	14.12446246	2.68E-45	9.37E-44	Up
ASB6	225.7423423	262.6875521	11551.54588	9750.697815	5447.668397	5.446336894	0.183236375	29.7230116	3.87E-194	7.90E-191	Up
SEC16A	2665.035922	3079.576259	10554.42547	8660.120669	6239.789579	1.742046197	0.177064348	9.838492167	7.69E-23	1.04E-21	Up
RPP30	2298.104906	2414.90862	11080.09938	9001.658942	6198.692962	2.091329589	0.173037182	12.08601278	1.25E-33	2.75E-32	Up
TCF7L2	3374.169993	3284.729938	7425.835681	6445.252299	5132.496978	1.058933969	0.166016763	6.378476173	1.79E-10	1.13E-09	Up

MKI67	5662.702786	5561.860073	13477.17242	11868.82144	9142.639179	1.175207311	0.160528183	7.32087843	2.46E-13	1.92E-12	Up
PPRC1	1775.627046	1560.227795	13078.76693	10092.23609	6626.714464	2.796440892	0.183787522	15.21561885	2.79E-52	1.25E-50	Up
PDCD11	1449.377513	1433.804679	10496.87801	7529.966127	5227.506582	2.644688559	0.192226135	13.75821537	4.55E-43	1.45E-41	Up
SSRP1	1926.387833	1750.240981	10208.03403	8956.218227	5710.220267	2.382150626	0.167608672	14.21257384	7.66E-46	2.71E-44	Up
CELF1	12881.67168	13281.24039	31791.65151	25429.21012	20845.94342	1.129088972	0.169234044	6.671760253	2.53E-11	1.72E-10	Up
INCENP	2431.316818	2265.774765	10898.60355	9117.459472	6178.28865	2.091516601	0.170281751	12.28268199	1.12E-34	2.55E-33	Up
YTHDF1	3712.384669	3754.842243	9861.642586	8135.353709	6366.055802	1.269308304	0.169827448	7.474105748	7.77E-14	6.30E-13	Up
TAOK2	1452.568217	1260.446035	10802.32222	8438.780415	5488.529221	2.826520517	0.184451603	15.3239141	5.29E-53	2.43E-51	Up
CWC15	665.2618852	504.9354388	12480.05201	10187.515	5959.441084	4.276309408	0.19325999	22.12723598	1.73E-108	3.92E-106	Up
DCUN1D2	3350.23971	3072.006013	7848.588175	6668.058382	5234.72307	1.17679564	0.17030776	6.909818079	4.85E-12	3.47E-11	Up
CRIM1	4785.259052	5443.764228	12599.57366	10826.61667	8413.8034	1.195549558	0.168865199	7.079904955	1.44E-12	1.07E-11	Up
EIF4E	4333.774367	4145.466961	17186.77021	12371.60096	9509.403124	1.801737643	0.187903924	9.588611068	8.93E-22	1.14E-20	Up
QTRT2	1902.457549	1734.343464	19932.44806	15084.85135	9663.525106	3.267501313	0.182594396	17.8948609	1.29E-71	1.03E-69	Up
TIAL1	7174.299036	4732.161061	21755.15318	16464.19691	12531.45255	1.682688407	0.215455897	7.809897183	5.72E-15	4.98E-14	Up
CDYL	2657.05916	3036.425855	8252.527075	6890.864466	5209.219139	1.411467074	0.1754284	8.045829958	8.57E-16	7.89E-15	Up
AHCTF1	2753.577971	2995.546524	13767.12308	9992.559682	7377.201814	2.047279018	0.189207495	10.82028497	2.76E-27	4.63E-26	Up
CMIP	2152.130175	2700.306912	8860.095449	7782.0888	5373.655334	1.778106357	0.180296545	9.862121065	6.08E-23	8.26E-22	Up
CHD1	3476.272537	2945.582897	12010.81887	9011.919748	6861.148514	1.711136736	0.189488409	9.030297655	1.71E-19	1.96E-18	Up
PRKCA	4183.01358	5072.822152	10365.18286	10153.80093	7443.70488	1.148498881	0.168852327	6.801794788	1.03E-11	7.19E-11	Up
TRIM11	1300.212078	1250.604715	13832.41731	11336.72533	6929.989859	3.302802151	0.17195043	19.2078737	3.18E-82	3.41E-80	Up
ENAH	3779.389463	3292.300185	10247.87458	8377.215576	6424.19495	1.397345496	0.176659082	7.909842369	2.58E-15	2.30E-14	Up
ELOC	3674.096215	4155.308281	12006.39215	9702.325441	7384.530521	1.471439943	0.176002819	8.360320304	6.25E-17	6.18E-16	Up
BTG3	2743.208181	1723.745119	10670.62707	9063.22378	6050.201038	2.143536777	0.216820536	9.886225804	4.78E-23	6.54E-22	Up
GABPA	2799.04551	1872.121949	15097.35475	11782.3375	7887.714926	2.524886709	0.212202123	11.89849881	1.20E-32	2.55E-31	Up
ADAMTS1	1231.611931	728.2577093	10526.75842	8995.795624	5370.605921	3.316704786	0.22675019	14.62713123	1.89E-48	7.32E-47	Up
AGPAT5	3402.088658	3216.59772	7735.706619	6989.075042	5335.86701	1.153788852	0.163097799	7.074214727	1.50E-12	1.11E-11	Up
GRAMD2B	4735.005456	6694.368943	11450.83782	12305.63863	8796.462713	1.055480208	0.191547039	5.510292476	3.58E-08	1.84E-07	Up
SLC16A1	2221.527998	2019.741755	15968.31342	13299.47103	8377.26355	2.786908897	0.170471695	16.34822074	4.48E-60	2.59E-58	Up
NIFK	2989.690103	3348.320009	14032.72674	11313.27206	7921.002228	1.999770025	0.175462465	11.39713858	4.32E-30	8.19E-29	Up
LARP1	6251.387763	6072.851709	20175.91808	16043.50385	12135.91535	1.55538546	0.171613638	9.06329753	1.27E-19	1.46E-18	Up
CNOT8	2865.252628	3415.695202	8640.972429	7606.18926	5632.02738	1.371217824	0.173784473	7.890335658	3.01E-15	2.68E-14	Up
BACH1	6006.501194	5994.121146	16499.52074	14850.31863	10837.61543	1.385422249	0.157729269	8.783545735	1.58E-18	1.72E-17	Up
SCAF4	3175.548639	2136.323551	15193.63608	11036.23028	7885.434637	2.304148685	0.218706758	10.53533374	5.94E-26	9.39E-25	Up
FGF18	61.42106135	28.0099119	12730.16212	9189.285117	5502.219553	7.938773947	0.562418935	14.11541016	3.05E-45	1.06E-43	Up
RPL30	21783.7372	21852.27343	52564.07114	48337.1935	36134.31882	1.209372255	0.15289238	7.909957654	2.57E-15	2.30E-14	Up
SUPV3L1	2308.474695	2213.540064	20453.69524	15259.28506	10058.74877	2.981560704	0.182598413	16.32851376	6.19E-60	3.54E-58	Up
ZFAND3	2683.382472	3079.576259	12107.1002	9330.004749	6800.015921	1.895379371	0.18386619	10.30847143	6.45E-25	9.69E-24	Up
NPTN	3519.347047	2929.685379	9786.388216	7947.727533	6045.787044	1.459616389	0.181811419	8.028188751	9.89E-16	9.07E-15	Up

UTP14A	1242.779397	1208.968359	10614.18629	7994.634077	5265.142031	2.924365444	0.185168412	15.79300387	3.48E-56	1.77E-54	Up
FBRS	1265.912005	1027.282444	11128.79338	9395.967076	5704.488727	3.162236574	0.181680938	17.40543953	7.50E-68	5.39E-66	Up
EIF4A2	14245.69785	13300.92303	68185.99312	57914.92343	38411.88436	2.194663996	0.161977451	13.54919451	8.01E-42	2.41E-40	Up
SMG1	3037.55067	2928.17133	9584.972107	7483.059583	5758.438422	1.516785511	0.178298756	8.506988769	1.79E-17	1.81E-16	Up
SSBP3	5278.222895	5692.825337	11015.91183	11087.53432	8268.623594	1.010546606	0.156984543	6.437236337	1.22E-10	7.83E-10	Up
DYRK1A	1650.391895	1451.216246	13674.1618	10587.68646	6840.8641	2.967846145	0.183199281	16.20009711	5.03E-59	2.78E-57	Up
EIF5B	2251.839691	2523.163145	14489.78637	10817.82169	7520.652725	2.406135292	0.186446553	12.90522808	4.21E-38	1.10E-36	Up
ZC3H18	3674.096215	3794.96455	11918.96428	9624.636478	7253.165379	1.528432472	0.172067181	8.8827658	6.52E-19	7.23E-18	Up
SON	22202.51716	20974.88186	46390.99939	40030.33774	32399.68404	1.001150457	0.159875	6.262082622	3.80E-10	2.35E-09	Up
SNF8	3080.625181	3550.445589	8946.416639	7458.140481	5758.906972	1.306934952	0.175472744	7.448079521	9.47E-14	7.63E-13	Up
RUNX1	3273.662802	3389.199339	9975.630824	9407.693712	6511.54667	1.540654896	0.158298088	9.732618492	2.19E-22	2.91E-21	Up
ADPGK	2978.522637	2482.283814	8331.101492	6975.882577	5191.94763	1.487261241	0.179542896	8.283598386	1.20E-16	1.16E-15	Up
GPBP1L1	3390.123516	2951.639094	12463.45178	10725.47443	7382.672205	1.870646511	0.170434358	10.97575941	5.00E-28	8.63E-27	Up
CALM3	3382.146755	2617.791225	8379.795496	8089.912994	5617.411618	1.456911822	0.179429894	8.119671643	4.67E-16	4.40E-15	Up
BSDC1	3624.640296	2479.255715	8158.459112	7104.875572	5341.807674	1.322478173	0.202702214	6.524241395	6.83E-11	4.49E-10	Up
SIK3	4390.409372	4257.506608	10235.70108	8583.897535	6866.878648	1.12199868	0.167564638	6.695915624	2.14E-11	1.46E-10	Up
SAFB	6764.293509	6549.777236	19904.78101	15356.02981	12143.72039	1.405246423	0.175962291	7.986065757	1.39E-15	1.26E-14	Up
CHTOP	6450.009117	5361.248542	40781.22874	35235.60945	21957.02396	2.686224707	0.170763076	15.73071168	9.32E-56	4.68E-54	Up
ZNF394	1227.623551	1146.135314	9815.161946	8283.402488	5118.080825	2.930863219	0.171636668	17.07597364	2.24E-65	1.51E-63	Up
SAP30BP	1987.011218	2119.669008	13711.78899	10359.01706	7044.371567	2.55139948	0.183006276	13.94159553	3.54E-44	1.19E-42	Up
SRSF2	10049.92145	10055.55837	36302.48701	30404.23543	21703.05057	1.730297391	0.162922144	10.62039416	2.40E-26	3.86E-25	Up
ITGA5	2501.512317	3293.057209	14769.7769	14202.422	8691.692106	2.321829996	0.179669877	12.92275607	3.35E-38	8.80E-37	Up
LSM12	1099.197695	1009.113853	10339.72918	7685.344053	5033.346194	3.096162969	0.189367287	16.35004134	4.35E-60	2.52E-58	Up
RPL29	525.668564	435.2891714	17416.96005	15533.39518	8477.828242	5.100113466	0.174803746	29.17622522	3.88E-187	7.20E-184	Up
PEF1	1447.78216	1024.254346	11433.13091	9813.728483	5929.723975	3.103791408	0.197782742	15.69293342	1.69E-55	8.43E-54	Up
USP1	2281.353707	1737.371562	11813.82949	9999.888829	6458.110898	2.440658857	0.18790207	12.98899399	1.41E-38	3.77E-37	Up
C1orf52	3766.626645	2505.751578	8997.324007	8240.893433	5877.648916	1.458686004	0.204140639	7.145495438	8.97E-13	6.74E-12	Up
ZNF326	3576.779728	3300.627456	9225.300483	7702.934007	5951.410418	1.299715979	0.170890806	7.605534858	2.84E-14	2.36E-13	Up
ATF3	4304.260351	2652.614359	14703.37599	14911.88347	9143.033542	2.089915287	0.213298976	9.798055885	1.15E-22	1.54E-21	Up
SNED1	2694.549938	3623.119955	7239.913119	7719.058132	5319.160286	1.243398548	0.186429286	6.669545188	2.57E-11	1.74E-10	Up
RPRD2	2117.032426	2156.006191	13953.04564	9505.904289	6932.997137	2.457020472	0.198444612	12.38139173	3.30E-35	7.61E-34	Up
IWS1	5098.745768	5106.131236	18346.57286	15185.99359	10934.36086	1.716413552	0.166511116	10.3081019	6.48E-25	9.72E-24	Up
DHX57	2424.935409	1721.474045	11340.16963	9211.27256	6174.462911	2.30956272	0.20131388	11.47244654	1.81E-30	3.53E-29	Up
YY1AP1	4252.411403	3629.176152	18560.16248	14241.99939	10170.93736	2.057397184	0.18400961	11.18092247	5.06E-29	9.11E-28	Up
ATP1A1	6177.203884	6013.803787	22944.83624	19889.84045	13756.42109	1.813036927	0.160600946	11.28908	1.49E-29	2.74E-28	Up
ARPC2	3861.550104	3828.273634	9762.041214	8532.593503	6496.114613	1.250549806	0.163274851	7.659169788	1.87E-14	1.57E-13	Up
AZI2	4698.312355	3983.463686	9764.254578	8871.200116	6829.307684	1.102125713	0.169029717	6.520307395	7.02E-11	4.61E-10	Up
SNHG16	4776.484615	3879.75131	21459.6691	17792.23844	11977.03587	2.181077196	0.17886125	12.19424103	3.34E-34	7.44E-33	Up

PPP4R2	2959.37841	2693.49369	16315.81154	12455.15324	8605.95922	2.347742123	0.1814204	12.94089373	2.65E-38	6.96E-37	Up
CCNL1	10128.89139	8814.794976	92832.90615	78212.26448	47497.21425	3.174623599	0.167529896	18.94959448	4.45E-80	4.49E-78	Up
SLMAP	3971.629408	3224.924991	9005.070781	7138.589651	5835.053708	1.165860228	0.187299037	6.22459275	4.83E-10	2.96E-09	Up
WDR43	3213.837093	2460.330099	44198.66251	33555.76885	20857.14964	3.776570088	0.19438972	19.42782824	4.49E-84	5.09E-82	Up
ZNF148	4719.84961	5184.104775	11564.82606	9303.619818	7693.100066	1.075412017	0.174416536	6.165768675	7.01E-10	4.23E-09	Up
TKT	7202.2177	8356.795066	16733.03063	14790.21963	11770.56575	1.018720984	0.166583988	6.115359576	9.63E-10	5.75E-09	Up
GNL3	6286.485513	4240.852066	34526.26253	29448.5146	18625.52868	2.603451442	0.201147616	12.9429893	2.57E-38	6.78E-37	Up
RNF168	1616.889498	1569.312091	12774.4294	9664.213874	6406.211216	2.816294595	0.182826657	15.40417927	1.53E-53	7.18E-52	Up
PGRMC2	2053.218336	1863.794678	8414.102635	7735.182256	5016.574477	2.043803249	0.165111143	12.37834837	3.42E-35	7.90E-34	Up
CDC25A	1177.369955	996.2444339	20194.73167	14351.93661	9180.070666	3.990634935	0.197278147	20.22846932	5.50E-91	7.79E-89	Up
NAA15	6752.328367	6337.05331	20553.29661	15590.56253	12308.31021	1.465490619	0.179262481	8.175110663	2.96E-16	2.81E-15	Up
ICE1	4074.529628	4849.499881	13303.42336	11165.22328	8348.169036	1.45525412	0.175488017	8.292612456	1.11E-16	1.08E-15	Up
NIPBL	3694.835794	4300.657013	19774.19254	15026.21817	10698.97588	2.12193627	0.184621442	11.49344434	1.42E-30	2.79E-29	Up
PRRC1	5228.766976	4536.091678	11097.80629	9652.487238	7628.788046	1.08761798	0.169399392	6.420436138	1.36E-10	8.71E-10	Up
RICTOR	3615.865858	3998.604179	8984.043824	7949.193363	6136.926806	1.153154656	0.166050368	6.94460765	3.80E-12	2.74E-11	Up
RPS14	1582.589425	1755.540154	18179.46389	16429.017	9486.652619	3.374003822	0.162987494	20.70099824	3.39E-95	5.40E-93	Up
RAD21	2243.065253	2085.602899	25717.07445	20505.48883	12637.80786	3.416724656	0.17321687	19.72512637	1.31E-86	1.65E-84	Up
DNAAF5	2010.941502	1607.920348	11260.48853	8975.274011	5963.656098	2.483574959	0.188528986	13.17343823	1.25E-39	3.48E-38	Up
VIRMA	3387.730487	3100.772949	12845.25704	9605.580694	7234.835293	1.791039482	0.184894156	9.686836648	3.43E-22	4.50E-21	Up
GAPVD1	3516.156343	3492.911716	11646.72052	8998.727283	6913.628966	1.558742739	0.178195856	8.747356834	2.18E-18	2.34E-17	Up
PCF11	2630.735848	2265.774765	16819.35182	15935.03246	9412.723723	2.741964152	0.163881965	16.73133557	7.75E-63	4.88E-61	Up
VDAC2	1464.533359	1633.659186	24136.73267	18806.59245	11510.37942	3.79296779	0.178920631	21.19916399	9.72E-100	1.79E-97	Up
QSOX2	2114.639398	2311.196244	9747.654349	8298.060783	5617.887693	2.027759902	0.170005997	11.92757864	8.50E-33	1.81E-31	Up
ENTR1	2583.672957	2497.424307	9430.036638	7282.240942	5448.343711	1.717965195	0.18032422	9.527090679	1.62E-21	2.05E-20	Up
DDX21	1776.424722	1542.059204	51429.72217	38765.32688	23378.38324	4.764578272	0.183840573	25.91690287	4.30E-148	2.43E-145	Up
COPS2	7379.301799	6736.005299	23572.32489	19250.73879	14234.59269	1.601229535	0.169835419	9.428124866	4.17E-21	5.18E-20	Up
CCT2	6304.034387	6647.433415	21939.96906	18334.59535	13306.50805	1.636832105	0.165490133	9.890813906	4.56E-23	6.26E-22	Up
WEE1	5316.511349	4944.127962	55441.44413	44822.13436	27631.05445	3.28866032	0.168972036	19.46274897	2.27E-84	2.60E-82	Up
ZNF3	2364.312024	2264.260716	15585.40148	12639.84775	8213.455492	2.608502964	0.171550867	15.20541992	3.26E-52	1.46E-50	Up
BLCAP	4605.781925	5445.278278	12274.20917	10908.70312	8308.493123	1.205774423	0.169650749	7.107392283	1.18E-12	8.82E-12	Up
PPIB	4222.897387	4667.813966	13023.43283	12006.60941	8480.188399	1.493331352	0.161038451	9.273135366	1.81E-20	2.17E-19	Up
TERF2IP	4204.550836	3408.124956	11137.64684	10111.29187	7215.403626	1.481051869	0.17397256	8.513134888	1.69E-17	1.73E-16	Up
PLK1	1813.117824	1642.743481	15361.85173	12466.87987	7821.148226	3.009648768	0.174384324	17.25871169	9.62E-67	6.65E-65	Up
PDIA3	7565.958011	8583.902459	19726.60522	18281.82549	13539.57279	1.23482127	0.160734419	7.682369969	1.56E-14	1.32E-13	Up
ZNF146	2188.025601	1951.609537	14423.38546	10945.34885	7377.092363	2.615698108	0.184376149	14.18674875	1.11E-45	3.90E-44	Up
CHAF1A	3611.079801	3449.761311	9943.537049	7714.660643	6179.759701	1.322680202	0.178821629	7.396645534	1.40E-13	1.11E-12	Up
TSR1	2412.970267	2632.931718	14684.56239	11860.02646	7897.62271	2.395343187	0.173882689	13.77562769	3.57E-43	1.15E-41	Up
C19orf48	2037.264814	1717.688921	13168.40816	11442.26505	7091.406738	2.712611999	0.173192185	15.6624388	2.73E-55	1.36E-53	Up

SLC3A2	6528.979053	7583.115877	16374.46568	14945.59755	11358.03954	1.150189344	0.164528637	6.990815487	2.73E-12	1.99E-11	Up
RPSA	2401.005125	2180.988005	8774.880941	8344.967327	5425.46035	1.901731116	0.162602621	11.69557477	1.34E-31	2.73E-30	Up
SETD5	20296.86891	16472.09927	57910.45149	47015.01529	35423.60874	1.512848658	0.178805411	8.460866213	2.65E-17	2.68E-16	Up
RFWD3	3018.406443	2977.377932	14249.6364	11263.43386	7877.213657	2.089384215	0.174447884	11.97712561	4.68E-33	1.01E-31	Up
MAP2K1	2033.276433	1946.310364	10165.98011	7724.92145	5467.62209	2.168802605	0.183111083	11.8441908	2.31E-32	4.83E-31	Up
XPO6	6529.77673	6155.367395	18069.90238	14132.06218	11221.77717	1.344152149	0.174974017	7.682010003	1.57E-14	1.32E-13	Up
PRELID1	3729.135867	3887.321556	12590.7202	11261.96803	7867.286413	1.647045058	0.160654139	10.25211719	1.16E-24	1.72E-23	Up
CNBP	2972.938904	3166.634094	14451.05251	12314.4336	8226.264778	2.124258489	0.166016757	12.79544622	1.74E-37	4.45E-36	Up
HNRNPF	2073.160239	2535.275539	14392.39837	11723.70432	7681.134615	2.502639358	0.182120785	13.74164597	5.72E-43	1.79E-41	Up
TOR1AIP2	6230.648184	7061.525896	16444.18664	14121.80137	10964.54053	1.201425579	0.167382474	7.177726249	7.09E-13	5.37E-12	Up
YWHAG	1090.423258	1436.075753	16889.07278	13276.01776	8172.897386	3.577611135	0.194514637	18.39250344	1.51E-75	1.33E-73	Up
HNRNPA3	9681.395085	8879.899096	22916.06251	19617.19616	15273.63821	1.196378141	0.16381931	7.303034912	2.81E-13	2.19E-12	Up
KIF5B	2387.444631	2695.764764	8795.907898	6688.579995	5141.924322	1.607244126	0.186492313	8.618286205	6.80E-18	7.10E-17	Up
RPS9	10509.3829	10100.22282	38843.4287	33917.82873	23342.71579	1.819898255	0.158702249	11.46737529	1.92E-30	3.74E-29	Up
PGAM1	819.2133766	670.7238362	10779.0819	8771.523711	5260.135705	3.714288644	0.185842864	19.98617844	7.27E-89	9.56E-87	Up
POLR1C	2542.193799	2620.819324	14300.54377	11967.03201	7857.647226	2.347116258	0.167469664	14.01517263	1.26E-44	4.31E-43	Up
RSL1D1	10321.13133	10505.23101	27412.51115	22638.27075	17719.28606	1.265055362	0.165194722	7.657964768	1.89E-14	1.59E-13	Up
EXOSC10	3510.57261	2986.462228	11880.23041	9564.537468	6985.450679	1.72298745	0.179940628	9.57531088	1.02E-21	1.30E-20	Up
JMJD1C	4648.058759	4807.106501	13204.92867	11005.44786	8416.385448	1.356588361	0.167204228	8.113361575	4.92E-16	4.62E-15	Up
BSG	15904.06651	17262.433	36521.61003	39281.29886	27242.3521	1.192498872	0.155322563	7.677563697	1.62E-14	1.37E-13	Up
RPL38	11513.65714	10683.1318	24017.21102	22991.53566	17301.38391	1.082607934	0.154575457	7.003750511	2.49E-12	1.82E-11	Up
SP3	4683.954185	4615.579266	11769.56222	10105.42855	7793.631055	1.234195692	0.163928811	7.528851615	5.12E-14	4.20E-13	Up
DHCR7	3489.833031	3935.771134	25101.7593	20476.17224	13250.88393	2.617803457	0.172333726	15.19031428	4.10E-52	1.84E-50	Up
OXSR1	3738.707981	3481.556346	13180.58166	10949.74634	7837.648083	1.740889563	0.169377126	10.27818577	8.84E-25	1.32E-23	Up
KDM2A	3518.549371	3677.62573	16837.05873	12591.47538	9156.177302	2.032062196	0.182222444	11.15154724	7.04E-29	1.26E-27	Up
TNKS	2109.853341	1990.974819	10372.92963	8242.359262	5679.029264	2.182741716	0.176935872	12.33634365	5.77E-35	1.32E-33	Up
SMARCC1	4119.997167	3751.814145	10387.3165	7795.281265	6513.602269	1.20805937	0.185045922	6.52843013	6.65E-11	4.37E-10	Up
ZNF622	134.8072645	223.3222705	13459.46551	10979.06293	6199.164494	6.091087645	0.223899076	27.2046127	5.73E-163	4.33E-160	Up
CHD2	4565.100443	4629.205709	25790.11546	19524.8489	13627.31763	2.301275932	0.178385711	12.90056202	4.47E-38	1.17E-36	Up
EIF1AX	3795.342985	3907.004197	12835.2969	10631.66134	7792.326357	1.607405565	0.168367485	9.547007065	1.33E-21	1.70E-20	Up
PSMD1	4390.409372	4825.275093	11300.32908	9242.054979	7439.517131	1.156596418	0.172590423	6.701393974	2.06E-11	1.41E-10	Up
TOMM20	1674.322179	1174.90225	14794.1239	11695.85356	7334.800472	3.217098097	0.204564266	15.72658883	9.94E-56	4.98E-54	Up
NET1	1578.601044	1536.760031	17924.92705	13929.77771	8742.51646	3.35418875	0.177270437	18.92130915	7.61E-80	7.61E-78	Up
RGMB	1744.517677	1313.437761	10108.43265	8919.57249	5521.490146	2.637745346	0.187482808	14.06926519	5.87E-45	2.03E-43	Up
PIGG	3504.191201	3237.79441	10552.2121	8816.964425	6527.790535	1.522714768	0.170158291	8.948813218	3.59E-19	4.05E-18	Up
PRPF8	7370.527361	7469.562181	17495.53447	14678.81658	11753.61015	1.116517901	0.164499501	6.787363463	1.14E-11	7.92E-11	Up
EXO1	8511.204215	7032.75896	20562.15007	15999.52896	13026.41055	1.23410899	0.183981752	6.7077793	1.98E-11	1.35E-10	Up
PTDSS2	2884.396855	3189.344833	9912.549955	9066.155439	6263.111771	1.643781844	0.163584346	10.04852775	9.33E-24	1.33E-22	Up

DHX36	4001.941101	3721.533159	17276.41145	13047.34835	9511.808515	1.973295514	0.181412823	10.87737617	1.48E-27	2.50E-26	Up
PSMD2	4661.619253	5545.962555	12962.56533	11572.72388	8685.717753	1.265267936	0.16976747	7.45294689	9.13E-14	7.36E-13	Up
CKAP5	4175.036819	4022.071943	9905.909863	7814.337049	6479.338919	1.112453203	0.175910788	6.32396235	2.55E-10	1.60E-09	Up
EIF3F	2839.726992	2600.379658	13927.59196	12208.89388	7894.148123	2.264495187	0.164772003	13.74320359	5.59E-43	1.76E-41	Up
PPP2R2D	2665.833598	2148.435945	12357.21032	9504.438459	6668.979579	2.183265049	0.191070346	11.42649864	3.08E-30	5.90E-29	Up
UBE2O	1521.168363	1529.946809	10783.50863	8365.48894	5550.028185	2.650079261	0.179899755	14.7308664	4.08E-49	1.62E-47	Up
TMEM39A	3729.933544	3094.716752	7704.719525	6607.959373	5284.332298	1.068702935	0.177571215	6.018446943	1.76E-09	1.02E-08	Up
CLK2	2132.985949	1575.368288	12905.01787	10634.593	6811.991276	2.666488388	0.194012444	13.74390393	5.54E-43	1.74E-41	Up
USF3	3430.007322	3475.500149	7939.336092	6707.635779	5388.119835	1.084998177	0.168254237	6.448563778	1.13E-10	7.28E-10	Up
ANKLE2	6664.583994	6260.593821	14832.85777	13264.29112	10255.58168	1.12033515	0.15992246	7.005489731	2.46E-12	1.79E-11	Up
ZFAS1	11723.44596	13204.7809	33256.89837	30672.48223	22214.40186	1.358714484	0.159009416	8.544868101	1.29E-17	1.32E-16	Up
UBE2N	3325.51175	3281.70184	8801.441307	6984.677554	5598.333113	1.256801601	0.175822325	7.148134341	8.80E-13	6.62E-12	Up
TUFM	3976.415465	3900.190975	14384.65159	13435.79317	8924.262801	1.820559819	0.156481421	11.63435131	2.76E-31	5.54E-30	Up
EIF3K	6376.622914	6705.724313	14653.5753	12985.78351	10180.42651	1.07918554	0.160220851	6.735612354	1.63E-11	1.12E-10	Up
AURKB	3283.234916	2962.994464	8248.100348	7579.804329	5518.533514	1.341556768	0.164351294	8.162739334	3.28E-16	3.10E-15	Up
CLK3	2478.379709	2163.576438	13270.2229	11174.01826	7271.549326	2.396877064	0.172714668	13.87766938	8.65E-44	2.85E-42	Up
EXOC3	3379.753726	3935.014109	12928.25819	11017.1745	7815.05013	1.710945731	0.172208755	9.935300502	2.92E-23	4.07E-22	Up
EMC3-AS1	4610.567982	3657.943089	14915.85891	11861.49229	8761.465569	1.695493779	0.187197088	9.057265794	1.34E-19	1.54E-18	Up
SMG1P3	1715.003661	1989.460769	11647.8272	9077.882075	6107.543428	2.4842053	0.184160256	13.48936711	1.81E-41	5.38E-40	Up
PITPNB	4666.40531	4200.72976	11009.27174	9007.52226	7220.982267	1.174874645	0.173366554	6.77682412	1.23E-11	8.50E-11	Up
SETD2	2667.42895	2439.133409	8573.464832	6455.513105	5033.885074	1.557645679	0.186337573	8.359267834	6.31E-17	6.23E-16	Up
IST1	4885.766243	4247.665288	16409.87951	12487.40149	9507.678131	1.661882594	0.183942299	9.034803871	1.64E-19	1.89E-18	Up
EXT1	3729.933544	3719.262085	14344.81104	11512.62487	8326.657885	1.795578804	0.171867294	10.44747237	1.50E-25	2.34E-24	Up
BACE2	11733.81575	15562.91267	29133.40154	32147.1067	22144.30916	1.166654367	0.180093149	6.478060801	9.29E-11	6.03E-10	Up
KPNA2	675.6316748	615.4610371	18374.23991	15605.22083	8817.638363	4.718236339	0.170690423	27.64206835	3.48E-168	3.38E-165	Up
ACBD3	2594.840423	1941.768217	16396.59932	13145.55893	8519.691723	2.703299494	0.193731409	13.95385243	2.98E-44	1.01E-42	Up
C16orf72	9434.115487	9553.651031	22538.68398	20215.25459	15435.42627	1.17104148	0.156694033	7.47342739	7.81E-14	6.33E-13	Up
RPL35A	6966.903244	7325.727498	16091.15511	15164.00615	11386.948	1.128856608	0.15549216	7.259894047	3.87E-13	2.97E-12	Up
EWSR1	14349.39575	12808.85701	46792.72493	36996.07068	27736.76209	1.62542506	0.173896429	9.347087073	9.01E-21	1.10E-19	Up
PTTG1IP	5275.032191	5214.385761	11272.66203	11014.24284	8194.080707	1.087287949	0.155014572	7.014101549	2.31E-12	1.69E-11	Up
SELENOF	4583.446993	4195.430587	24392.37619	20152.22393	13330.86942	2.343245135	0.168657014	13.8935528	6.93E-44	2.29E-42	Up
BCOR	8429.841251	6939.644929	23793.66127	19691.95346	14713.77523	1.500568971	0.17681156	8.486826133	2.12E-17	2.15E-16	Up
RBM12B	8209.682641	7873.813341	24467.63056	21849.65448	15600.19526	1.526029164	0.157684229	9.677753888	3.75E-22	4.91E-21	Up
RBM33	2622.759087	1994.002917	14020.55324	10511.46332	7287.194642	2.409960107	0.200078525	12.04507136	2.06E-33	4.49E-32	Up
PRPF39	4548.349244	3169.662192	11607.98666	9032.441361	7089.609863	1.419389962	0.207126513	6.852768119	7.24E-12	5.12E-11	Up
MUC1	3576.779728	4881.294916	8089.844833	9583.593252	6532.878182	1.062983822	0.193208694	5.501739077	3.76E-08	1.92E-07	Up
NR2F2	2147.344119	2003.844238	10629.67984	8575.102558	5838.992688	2.210099664	0.175158333	12.61772495	1.69E-36	4.14E-35	Up
AHNAK2	10132.08209	14329.71952	30750.26382	33544.04221	22189.02691	1.394116359	0.189480973	7.357553324	1.87E-13	1.48E-12	Up

UBE2L3	2888.385235	3218.868794	9372.489178	7785.020459	5816.190917	1.490402867	0.173348885	8.597706673	8.13E-18	8.46E-17	Up
YTHDF3	4679.965804	5185.618824	13996.20624	11308.87457	8792.666359	1.359080028	0.173724327	7.823199248	5.15E-15	4.50E-14	Up
KIF18B	3978.808493	3126.511787	7941.549456	6295.73769	5335.651857	1.002998079	0.191463253	5.238593106	1.62E-07	7.78E-07	Up
NKRF	4052.992373	4213.599179	12617.28057	10150.86927	7758.685346	1.461816352	0.172275859	8.485323265	2.15E-17	2.18E-16	Up
KPNA4	1687.882673	1788.849238	11781.73572	8856.541821	6028.752362	2.56970866	0.184643638	13.91712538	4.99E-44	1.66E-42	Up
INSIG1	3787.366224	3013.715115	10564.38561	10348.75625	6928.555799	1.620658767	0.173948878	9.316868165	1.20E-20	1.45E-19	Up
GPATCH8	2449.663369	2461.844148	12245.43544	8994.329794	6537.818188	2.112753209	0.18644467	11.33179732	9.13E-30	1.70E-28	Up
POFUT2	1586.577805	1361.130313	10337.51581	7793.815436	5269.759842	2.621139128	0.190207816	13.78039651	3.34E-43	1.07E-41	Up
RPL14	12762.81794	11774.00432	33656.41054	30591.8616	22196.2736	1.388749942	0.157128858	8.838286984	9.72E-19	1.06E-17	Up
SF3B3	4150.308859	3691.252173	35914.04166	26974.19441	17682.44927	3.003678177	0.182571515	16.45206357	8.11E-61	4.79E-59	Up
PAX8-AS1	3126.09272	4285.51652	9069.258332	9162.900186	6410.94194	1.29854468	0.187111141	6.939964505	3.92E-12	2.82E-11	Up
TSPYL1	5183.299437	4014.501697	14552.86724	13778.79727	9382.366413	1.6231417	0.176864185	9.177334012	4.42E-20	5.23E-19	Up
HMGB1	8538.325203	9004.808162	19469.85501	17223.49659	13559.12124	1.064676027	0.159322242	6.68253229	2.35E-11	1.60E-10	Up
TUBB	478.6056728	430.7470235	12138.0873	10073.1803	5780.155074	4.610671187	0.177756664	25.93810593	2.48E-148	1.44E-145	Up
POM121	4744.57757	4037.212436	13276.86299	10378.07284	8109.181459	1.429755642	0.182695516	7.825893434	5.04E-15	4.40E-14	Up
XRCC6	8169.001159	7512.712585	22219.95958	18621.89793	14130.89281	1.381061393	0.166286454	8.30531508	9.96E-17	9.74E-16	Up
TSC22D2	6383.004323	7049.413502	29373.55151	24002.95801	16702.23184	1.990541135	0.16983267	11.72060203	1.00E-31	2.04E-30	Up
PIK3R4	1555.468437	1543.573253	9358.102313	7554.885228	5003.007308	2.448466429	0.175224043	13.97334744	2.27E-44	7.69E-43	Up
SIAH1	1774.82937	1642.743481	10652.92016	8616.145784	5671.659699	2.49548086	0.175628599	14.20885249	8.07E-46	2.85E-44	Up
PRPF40A	4437.472263	3536.819145	12591.82688	9938.32399	7626.11057	1.49863711	0.188201632	7.96293364	1.68E-15	1.51E-14	Up
HDAC2	2915.506224	2383.113585	9748.761031	7836.324491	5720.926333	1.730926251	0.185267135	9.342867244	9.38E-21	1.14E-19	Up
SNHG17	2685.775501	2536.789588	73031.04656	56987.05336	33810.16625	4.63786639	0.173516738	26.72863974	2.19E-157	1.44E-154	Up
EOLA2	2121.020807	1342.961722	9671.293296	8393.3397	5382.153881	2.382926463	0.215058325	11.08037305	1.56E-28	2.77E-27	Up
SND1	4650.451788	5004.689934	19312.70618	14700.80403	10917.16298	1.816857935	0.179635828	10.11411787	4.78E-24	6.89E-23	Up
HNRNPAB	3440.377111	3237.037386	8689.666433	7563.680205	5732.690284	1.283563056	0.165899754	7.736979851	1.02E-14	8.69E-14	Up
ERO1A	5656.321377	5279.48988	11311.3959	11371.90524	8404.778099	1.052580991	0.15650856	6.725389279	1.75E-11	1.20E-10	Up
NOL8	6281.699456	6231.826885	14234.14285	11546.33895	9573.502035	1.042944129	0.16955909	6.150918403	7.70E-10	4.63E-09	Up
RPS4X	9837.739605	8200.847988	19854.98032	17113.55938	13751.78182	1.035306045	0.17172438	6.028882124	1.65E-09	9.60E-09	Up
ZNF84	5449.723261	4714.749494	11140.96689	9322.675601	7657.028811	1.009712421	0.173413374	5.822575269	5.79E-09	3.21E-08	Up
MIER1	5596.495668	4962.296554	15772.43072	13018.03176	9837.313676	1.447291411	0.171800411	8.42426045	3.63E-17	3.63E-16	Up
QRICH1	2291.723497	2034.882248	10357.43609	8199.850207	5720.973009	2.10093876	0.180292063	11.65297418	2.22E-31	4.47E-30	Up
RPL23A	1140.676854	1146.135314	15476.94665	13954.69681	7929.613907	3.686028315	0.161157231	22.87224906	8.78E-116	2.39E-113	Up
YTHDF2	1539.514914	1353.560067	19996.63561	15937.96412	9706.918677	3.634891311	0.177853835	20.43752004	7.76E-93	1.15E-90	Up
MAFK	2215.146589	3165.120044	8771.560896	8337.638179	5622.366427	1.668960096	0.195052011	8.556487519	1.16E-17	1.20E-16	Up
TOGARAM1	1418.268144	1077.246071	11025.87197	8202.781866	5431.042012	2.946210533	0.203226585	14.49717087	1.26E-47	4.76E-46	Up
CTR9	2791.068749	3308.954727	12019.67233	9299.222329	6854.729534	1.805384759	0.185808504	9.716373126	2.57E-22	3.39E-21	Up
SMOC1	4750.161303	5787.453418	13240.34249	14479.46377	9564.355245	1.395269927	0.170537054	8.181623243	2.80E-16	2.66E-15	Up
RPL10A	1677.512883	1351.288993	9697.853662	9038.304679	5441.240054	2.629211443	0.175719374	14.96255869	1.29E-50	5.46E-49	Up

PNP	658.0828001	455.7288368	12598.46697	9907.541571	5904.955045	4.337313465	0.208520941	20.80037359	4.29E-96	7.01E-94	Up
FOXJ3	3533.705218	2966.022562	10022.11146	7710.263155	6058.0256	1.448206886	0.187654512	7.717410437	1.19E-14	1.01E-13	Up
ARHGAP11A	3686.859033	3358.161329	8743.893848	6863.013705	5662.981979	1.147792561	0.179629282	6.389785394	1.66E-10	1.06E-09	Up
SFMBT2	3217.027797	3080.333284	8146.285611	7326.215826	5442.46563	1.297042785	0.162866203	7.963854767	1.67E-15	1.50E-14	Up
CAPZA2	3558.433177	4143.195887	10901.92359	9098.403688	6925.489086	1.376908156	0.174992697	7.868374942	3.59E-15	3.18E-14	Up
TOP1	2138.569681	2253.662371	25101.7593	18736.23263	12057.556	3.31924247	0.182196767	18.21789999	3.72E-74	3.16E-72	Up
SREBF2	3560.02853	2658.670556	11511.70533	8806.703618	6634.277008	1.708350489	0.200193822	8.53348256	1.42E-17	1.46E-16	Up
BRD2	8001.489173	6085.721128	58825.67744	48359.18094	30318.01717	2.927706034	0.185700524	15.76573926	5.35E-56	2.70E-54	Up
PPP1R10	458.6637698	501.9073402	12027.4191	8651.325692	5409.828976	4.428199662	0.196536721	22.53115674	2.06E-112	4.99E-110	Up
ABCF1	3472.284156	3990.276908	21129.87789	16074.28627	11166.68131	2.317805446	0.183435728	12.63551804	1.35E-36	3.32E-35	Up
RACK1	69373.0946	56415.74768	138148.2107	129104.3988	98260.36295	1.087208804	0.166455843	6.531514795	6.51E-11	4.29E-10	Up
TBC1D8	3315.939637	4234.795869	8805.868035	8150.012004	6126.653886	1.167112438	0.178533263	6.537226843	6.27E-11	4.14E-10	Up
TRIM27	2852.48981	2489.85406	9698.960344	8252.620069	5823.481071	1.748780555	0.172906293	10.11403651	4.79E-24	6.89E-23	Up
ZBTB10	1152.641995	709.3320932	19216.42485	15838.28772	9229.171665	4.235084404	0.219650576	19.28100747	7.76E-83	8.50E-81	Up
IPO7	2682.584796	2723.017651	11887.97718	8951.820739	6561.350092	1.947028913	0.182548592	10.66581171	1.47E-26	2.38E-25	Up
AC004980.	3892.659472	4168.934725	11150.92702	9416.488689	7157.252477	1.351362616	0.167801618	8.053334847	8.06E-16	7.44E-15	Up
RNPS1	14401.2447	11961.74643	39744.26778	33920.76039	25007.00482	1.482519868	0.171553218	8.641749113	5.54E-18	5.80E-17	Up
TSN	2444.877312	2726.802774	11158.6738	9473.65604	6451.002481	1.996305722	0.170464651	11.71096595	1.12E-31	2.28E-30	Up
SCAF8	1288.246936	1251.36174	11678.8143	8742.207121	5740.157524	3.007620967	0.185479303	16.21539936	3.93E-59	2.17E-57	Up
CHUK	1840.238812	1556.442672	12032.95251	9220.067537	6162.425383	2.645746278	0.187945335	14.07721172	5.24E-45	1.82E-43	Up
COG8	4397.588457	4013.744672	10203.6073	8390.408041	6751.337118	1.144645044	0.172351878	6.641326202	3.11E-11	2.10E-10	Up
NDUFS3	2641.105638	3026.584534	7917.202454	6898.193613	5120.77156	1.386385521	0.171626772	8.077909414	6.59E-16	6.13E-15	Up
PHB2	8350.871315	7414.299381	20453.69524	17565.03486	13445.9752	1.270064513	0.166136216	7.644717975	2.09E-14	1.76E-13	Up
PPP2R2A	9831.358196	10722.49708	35864.24097	31537.32163	21988.85447	1.71340317	0.160294073	10.68912366	1.14E-26	1.87E-25	Up
CCNL2	6540.146519	5449.063401	15006.60683	12510.85476	9876.667877	1.198755444	0.176163369	6.804794035	1.01E-11	7.05E-11	Up
LAMTOR5-A	12698.20618	9285.664306	34892.57425	27840.49964	21179.23609	1.512854987	0.194418573	7.781432407	7.17E-15	6.20E-14	Up
AC006001.	3674.893891	3766.197613	15298.77086	12726.33169	8866.548514	1.91325337	0.167163154	11.44542519	2.48E-30	4.79E-29	Up
SNHG15	2082.732353	1806.260805	20714.87217	19180.37897	10946.06108	3.358869777	0.163850324	20.49962237	2.17E-93	3.33E-91	Up
CTC-338M1	6534.562786	5147.010567	13758.26963	12528.44471	9492.071923	1.170201323	0.176101908	6.64502353	3.03E-11	2.05E-10	Up
FAM133B	3824.857002	3150.736576	9154.47284	7510.910343	5910.24419	1.256711095	0.181867997	6.910017793	4.85E-12	3.47E-11	Up
OLMALINC	2921.887633	2723.017651	11092.27288	10668.30708	6851.371311	1.94676439	0.158788077	12.26014209	1.48E-34	3.34E-33	Up
KIFC1	2468.807596	1973.563252	10167.0868	8756.865416	5841.580765	2.091035422	0.180900985	11.55900516	6.65E-31	1.31E-29	Up
NEAT1	18558.73264	24714.58361	53688.45997	56340.62255	38325.59969	1.346315363	0.177940008	7.566119476	3.85E-14	3.18E-13	Up
LUCAT1	4624.128476	6754.17389	17474.50751	18177.75159	11757.64037	1.647639835	0.194897592	8.453874783	2.82E-17	2.84E-16	Up
CDK11B	1723.778098	1814.588076	10872.04318	8614.679954	5756.272327	2.461514616	0.177218391	13.88972447	7.31E-44	2.42E-42	Up
AC139887.	4129.56928	3592.838969	8123.045291	8092.844653	5984.574548	1.070330878	0.164218576	6.517721097	7.14E-11	4.68E-10	Up
OSMR-AS1	7375.313418	10281.90874	22856.30169	24259.47818	16193.25051	1.415900012	0.186868622	7.576981089	3.54E-14	2.93E-13	Up
PVT1	5231.95768	6633.049947	19110.18339	16065.49129	11760.17058	1.56790936	0.180985173	8.663192296	4.59E-18	4.83E-17	Up

LINC02615	3548.861064	4979.70812	12153.58084	13495.89218	8544.510552	1.588428662	0.191813484	8.281110545	1.22E-16	1.18E-15	Up
TUG1	6810.558724	6206.845071	15269.99713	13770.0023	10514.3508	1.157690807	0.160868889	7.196486601	6.18E-13	4.69E-12	Up
LSM14A	5597.293344	5268.13451	12876.24414	11511.15904	8813.207758	1.166500535	0.16062658	7.26218869	3.81E-13	2.92E-12	Up
OTUD7B	1841.834164	1607.920348	13194.96853	10278.39643	6730.779869	2.766702795	0.18281412	15.13396664	9.67E-52	4.28E-50	Up
DCP1A	1905.648254	1363.401387	14262.91658	10816.35586	7087.08052	2.939836414	0.206424599	14.24169613	5.05E-46	1.80E-44	Up
FP671120.	19422.61588	33448.37695	44734.29656	75834.68903	43359.99461	1.189302027	0.533762792	2.228147117	0.025870706	0.053529129	Up
FP236383.	26654.3476	43008.08418	82938.06311	67168.70504	54942.29998	1.107543886	0.216999743	5.103894915	3.33E-07	1.54E-06	Up

Supplementary Table 5: Proteins interacting with KHSRP in HL7702 cells as determined by mass spectrometry analysis of KHSRP or IgG immunoprecipitations

co-immunoprecipitations; p-values and permutation-based false discovery rate (FDR) calculated by one-tailed t-test

Gene_Name	Sq	Score	Spectra M	Q value	Delta Mass (PPM)	Protein AC	Number of PSMs	Unique peptide
SF3B3	NVSEELDR	6.90E-02	961.46	0.00055	4.62	sp_Q15393	69	24
FUBP2	DQGGFGDRNEYGSR	2.26E-02	1557.67	0.00055	4.73	sp_Q92945	69	22
SF3B1	VAIGPCR	2.47E-01	772.42	0.00534	4.73	sp_O75533	33	10
RL8	VGLIAARR	1.92E-01	855.56	0.00163	3.97	sp_P62917	24	8
SURF6	MASLLAKDAYLQSLAK	3.37E-01	1633.92	0.00869	4.02	sp_O75683	20	8
ENOA	IEEELGSKAK	3.00E-02	1103.59	0.00055	-1.17	sp_P06733	20	7
NPM	GPSSVEDIK	3.98E-02	931.48	0.00055	4.73	sp_P06748	18	7
RL4	AAAAAAALQAK	2.86E-03	956.56	0	3.7	sp_P36578	16	7
H4	DAVTYTEHAK	2.04E-02	1134.55	0.00055	4.77	sp_P62805	16	6
G3P	GALQNIIPASTGAAK	1.46E-01	1411.8	0.00163	7.12	sp_P04406	16	5
CH60	SIDLKDKYK	1.11E-01	1109.63	0.00144	5.04	sp_P10809	14	8
DHX9	ILTTEGR	3.87E-02	789.45	0.00055	4.8	sp_Q08211	13	8
TCPD	AVADAIR	7.26E-02	715.41	0.00055	3.72	sp_P50991	12	4
NUCL	ALVATPGK	9.37E-02	756.46	0.00144	3.33	sp_P19338	12	7
LDHB	LKDDEVAQLKK	2.07E-01	1286.74	0.00268	3.68	sp_P07195	12	5
KPYM	QAHLYR	2.90E-01	770.4	0.00632	3.43	sp_P14618	11	3
FETUA	CNLLAEK	8.33E-02	847.44	0.00144	4.41	sp_P02765	11	4
EF2	VNFTVDQIR	7.81E-03	1091.59	0	4.53	sp_P13639	11	4
SMD2	MSLLNKPK	2.13E-01	841.52	0.00268	4.24	sp_P62316	10	1
SERA	GGIVDEGALLR	3.13E-03	1099.61	0	4.04	sp_O43175	10	3
PHF5A	IVNLGSSK	3.14E-02	817.48	0.00055	6.61	sp_Q7RTV0	10	3
RS3	MAVQISKKR	7.04E-02	971.6	0.00055	2.51	sp_P23396	9	3
ROA1	SSGPYGGGGQYFAKPR	1.64E-01	1628.79	0.00163	5.37	sp_P09651	9	3
ROA1	NQGGYGGSSSSSSYGSGR	2.94E-06	1694.71	0	4.14	sp_P09651	9	3
HSP7C	LSKEDIER	9.63E-03	989.53	0	4.06	sp_P11142	9	5
VIME	SSVPGVR	1.76E-01	701.4	0.00163	3.53	sp_P08670	8	6
SSBP	SLNRVHLLGR	1.84E-01	1164.7	0.00163	3.59	sp_Q04837	8	2
SF3A1	NGPEFEAR	7.71E-02	919.43	0.00144	4.36	sp_Q15459	8	2

RL7	IALTDNALIAR	1.41E-03	1170.69	0	3.69	sp_P18124	8	3
RL7	ASINMLR	2.23E-01	820.44	0.00339	6.13	sp_P18124	8	3
RL3	VAFSVAR	3.75E-02	749.43	0.00055	3.67	sp_P39023	8	2
PIMT	VQLVVGDGR	1.82E-01	942.54	0.00163	4.68	sp_P22061	8	1
MATR3	GPGPLQER	9.61E-02	853.45	0.00144	2.37	sp_P43243	8	2
HNRPK	NLPLPPPPPPR	2.03E-01	1194.71	0.00268	4.91	sp_P61978	8	3
RS28	VTKVLGR	3.26E-01	772.51	0.00869	4.28	sp_P62857	7	2
RL12	IGPLGLSPK	1.41E-01	881.55	0.00163	4.9	sp_P30050	7	1
GRP75	VLENAEGAR	1.91E-02	958.5	0.00055	4.57	sp_P38646	7	4
DDX5	LLQLVEDR	2.06E-01	985.57	0.00268	5.34	sp_P17844	7	3
VIGLN	AGLLER	3.06E-01	658.39	0.00757	4.29	sp_Q00341	6	1
RL7A	VAPAPAVVK	1.35E-01	851.54	0.00163	4.66	sp_P62424	6	3
RL28	ATLSSIR	7.23E-02	747.44	0.00055	6.06	sp_P46779	6	2
RL24	IVKPVKVSAPR	1.72E-01	1193.78	0.00163	2	sp_P83731	6	2
PRDX1	IGHPAPNFK	1.60E-01	980.54	0.00163	4.55	sp_Q06830	6	3
PARP1	ILTLGKLSR	3.71E-01	1000.66	0.00976	4.33	sp_P09874	6	4
HNRPU	ISKEVLAGR	2.41E-01	972.59	0.00534	3.8	sp_Q00839	6	5
FLNB	AYGPGLEK	6.49E-02	834.44	0.00055	4.26	sp_O75369	6	3
FLNA	KRAEFTVETR	2.90E-01	1236.67	0.00632	3.86	sp_P21333	6	4
TKT	LILDSAR	3.34E-01	787.47	0.00869	4.32	sp_P29401	5	2
TCPQ	FAEAFEAIPR	7.29E-03	1150.59	0	5.07	sp_P50990	5	2
SND1	QINLSNIR	1.31E-01	957.55	0.00163	5.38	sp_Q7KZF4	5	2
RL14	NRIIKNEVK	2.66E-01	1113.68	0.00632	3.09	sp_P50914	5	2
PAIRB	SAAQAAAQTNSNAAGK	9.05E-03	1460.72	0	5.87	sp_Q8NC51	5	4
GLYM	LIDYAR	3.59E-01	750.42	0.00976	5.4	sp_P34897	5	3
XRCC6	AIVEKLR	1.28E-01	828.53	0.00163	5.27	sp_P12956	4	1
TPIS	VVFEQTK	4.16E-02	850.47	0.00055	5.94	sp_P60174	4	3
STMN1	ASGQAFELILSPR	5.29E-04	1388.76	0	4.32	sp_P16949	4	3
RS4X	LIYDTK	3.14E-01	752.42	0.00807	4.03	sp_P62701	4	2
RS3A	IASDGLK	1.20E-01	703.4	0.00163	2.92	sp_P61247	4	3
RS20	MAFKDTGK	1.13E-01	808.42	0.00144	4.85	sp_P60866	4	2
RS16	PSKGPLQSVQVFGR	3.13E-01	1499.84	0.00807	4.84	sp_P62249	4	2
RS14	TPGPGAQSALR	3.20E-04	1054.57	0	4.64	sp_P62263	4	2
RL31	IDTRLNK	6.88E-02	859.5	0.00055	4.24	sp_P62899	4	2
RL29	AQAAAPASVPAQAPK	1.44E-01	1377.76	0.00163	5.13	sp_P47914	4	2
RL19	SMLRLQKR	3.50E-01	1089.62	0.00869	3.71	sp_P84098	4	3

RL15	SLQSVAEER	3.20E-02	1018.52	0.00055	6.05	sp_P61313	4	3
RL13A	GQAALDRLK	4.21E-02	971.57	0.00055	5.6	sp_P40429	4	3
RBMX	GLPPSMER	3.68E-01	902.44	0.00976	3.07	sp_P38159	4	2
PUR6	MATAEVLNIGKK	3.96E-01	1185.69	0.00985	5.72	sp_P22234	4	1
PGK1	ELNYFAK	8.48E-02	884.45	0.00144	4.11	sp_P00558	4	2
PESC	GSATNYITR	9.52E-02	982.5	0.00144	5.15	sp_O00541	4	1
PEBP1	LYEQLSGK	4.51E-02	937.5	0.00055	5.22	sp_P30086	4	2
PA2G4	ALLQSSASR	4.19E-02	932.52	0.00055	6.17	sp_Q9UQ80	4	1
KHDR1	ILGPQGNTIKR	7.34E-03	1196.72	0	5.01	sp_Q07666	4	3
IF4B	SILPTAPR	4.46E-02	854.51	0.00055	4.43	sp_P23588	4	2
HNRPM	KACQIFVR	3.83E-02	1021.57	0.00055	4.88	sp_P52272	4	3
FUBP3	GVPQQIEVAR	5.39E-02	1096.62	0.00055	5.09	sp_Q96I24	4	1
CNBP	TSEVNCYR	1.44E-02	1028.45	0	3.74	sp_P62633	4	3
CBX3	DAADKPRGFAR	2.81E-01	1203.63	0.00632	4.87	sp_Q13185	4	2
SF3A3	TYEDLKR	5.30E-02	924.48	0.00055	5.13	sp_Q12874	3	1
SF3A3	TYEDLKR	6.12E-02	924.48	0.00055	5.29	sp_Q12874	3	1
RS7	ELNITAAK	4.87E-02	859.49	0.00055	3.87	sp_P62081	3	1
RS25	LITPAVVSER	2.46E-01	1084.64	0.00534	7.32	sp_P62851	3	1
RS21	LAKADGIVSK	2.88E-01	1001.6	0.00632	5.45	sp_P63220	3	2
RMXL1	SAPSGLVR	1.05E-01	786.45	0.00144	5.23	sp_Q96E39	3	1
RM38	TQQLLER	1.40E-01	887.5	0.00163	4.85	sp_Q96DV4	3	1
RL32	AAQLAIR	1.37E-01	742.46	0.00163	2.69	sp_P62910	3	1
PPIA	KITIADCGQLE	5.05E-03	1247.64	0	5.04	sp_P62937	3	1
NU205	TGPVAVR	3.70E-01	699.42	0.00976	1.55	sp_Q92621	3	1
KAIN	ADLSGITK	3.44E-02	804.45	0.00055	4.22	sp_P29622	3	1
HBAZ/HBA	VDPVNFK	8.06E-02	818.45	0.00144	5.46	sp_P02008/sp_P6	3	1
CASP5	LVDSLR	2.47E-01	702.42	0.00534	5.63	sp_P51878	3	1
ASSY	IKQGLGLK	2.01E-01	856.56	0.00235	3.57	sp_P00966	3	1
1433G	MVDREQLVQKAR	9.93E-02	1383.78	0.00144	4.28	sp_P61981	3	1
SRSF1	YGPPSR	1.50E-01	676.34	0.00163	5.19	sp_Q07955	2	2
RS18	VLNTNIDGR	5.06E-02	1001.54	0.00055	4.83	sp_P62269	2	2
RL18	GCGTVLLSGPR	3.51E-02	1116.59	0.00055	4.42	sp_Q07020	2	2
PDIA3	LAPEYEAAATR	8.89E-02	1191.61	0.00144	5.76	sp_P30101	2	2
MCM2	YIIYAK	2.67E-01	770.45	0.00632	6.45	sp_P49736	2	2
IMDH2	VRDVFEAKAR	1.23E-01	1190.67	0.00163	3.97	sp_P12268	2	2
IMDH2	VRDVFEAK	3.93E-01	963.53	0.00985	5.85	sp_P12268	2	2

HBA	YGAEALER	2.70E-01	908.45	0.00632	4.33	sp_P69905	2	2
HBA	VGAHAGEYGAEALER	3.38E-02	1529.74	0.00055	2.52	sp_P69905	2	2
EFTU	EHILLAR	1.41E-01	851.51	0.00163	4.72	sp_P49411	2	2
DDX54	TKQQILK	3.45E-01	886.55	0.00869	4.8	sp_Q8TDD1	2	2
